# Supplementary material for: Photo-induced intramolecular dearomative [5 + 4] cycloaddition of arenes for the construction of highly strained medium-sized-rings
Source: Nat Commun. 2024 Mar 19;15:2462. doi: 10.1038/s41467-024-46647-4 (PMC10951311; doi:10.1038/s41467-024-46647-4)
Supplement: Supplementary file 1 — Supplementary Information [file 41467_2024_46647_MOESM1_ESM.pdf]

Supplementary Information

Photo-Induced Intramolecular Dearomative [5 + 4] Cycloaddition of Arenes for the Construction of Highly Strained Medium-Sized-Rings

Min Zhu,[a,b],† Yuan-Jun Gao,[a],† Xu-Lun Huang,[a,b],† Muzi Li[a],Chao Zheng,[a],* and Shu-Li You[a,b],*

[a] New Cornerstone Science Laboratory, State Key Laboratory of Organometallic Chemistry, Shanghai Institute of Organic Chemistry, Chinese Academy of Sciences, 345 Lingling Lu, Shanghai 200032, China

[b] School of Physical Science and Technology, ShanghaiTech University, 100 Haike Road, Shanghai 201210, China

† These authors contributed equally.

Email: zhengchao@sioc.ac.cn or slyou@sioc.ac.cn

Table of Contents

1. General Methods............2

2. General Procedures and Characterization Data............3

3. Transformations of Products............32

4. Mechanistic Studies............37

5. Data of X-Ray Crystalloraphic Analyses............43

6. Copies of NMR Spectra............57

7. Unsuccessful Substrates............163

8. References............164

1. General Methods

Unless stated otherwise, all reactions were carried out in flame-dried glasswares under a dry argon atmosphere. All solvents were purified and dried according to standard methods prior to use.

^{1}H and ^{13}C NMR spectra were recorded on an Agilent instrument (400 MHz and 100 MHz, respectively) or an Agilent instrument (600 MHz and 150 MHz, respectively), and internally referenced to tetramethylsilane signal or residual protio solvent signals. ^{19}F NMR spectra were recorded on an Agilent instrument (376 MHz) and referenced relative to $CFCl_3$. Data for ^{1}H NMR are recorded as follows: chemical shift (δ, ppm), multiplicity (s = singlet, d = doublet, t = triplet, m = multiplet or unresolved, br = broad singlet, coupling constant(s) in Hz, integration). Data for ^{13}C NMR are reported in terms of chemical shift (δ, ppm).

Substrates **1** and **9** were synthesized according to the literature procedures.[1] Reagents were purchased from Alfa Aesar, and used without further purification.

2. General Procedures and Characterization Data

2.1 General procedure for the synthesis of the alkyl bromide

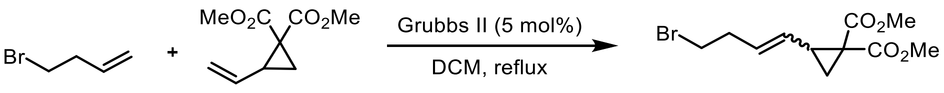

S1

To a solution of 4-bromo-1-butene (1.3 mL, 13 mmol) in anhydrous DCM (30 mL) were added substituted vinylcyclopropanes (1.6 mL, 10 mmol) and Grubbs II catalyst (300 mg, 0.5 mmol) under argon. Then, the mixture was refluxed for 6 h at 60 °C. After completion (monitored by TLC), the reaction was quenched with water. The aqueous layer was extracted with EtOAc (30 mL × 3). The combined organic layers were washed with brine, dried over Na_2SO_4, filtered and concentrated by rotary evaporation. Then, the residue was purified by silica gel column chromatography (petroleum ether/EtOAc = 9/1) to afford the desired product **S1** (1.5 g, 51% yield).

2.2 General procedure for the synthesis of substrates 1a-1j, 1l, 1s, 9a and 9b[2]

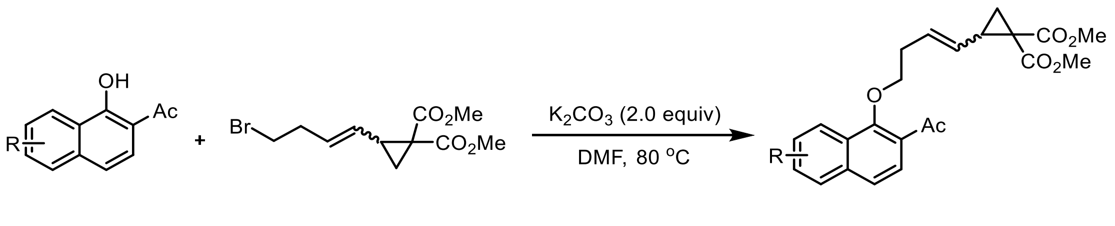

A **S1** **1**

In a round bottom flask, naphthol derivative **A** (4.7 mmol) was dissolved in DMF (reagent grade, 30.0 mL). After the addition of **S1** (1.5 g, 5.2 mmol), K_2CO_3 (1.3 g, 9.4 mmol) was added in sequence under argon. Then, the mixture was stirred for 24 h at 80 °C. After completion (monitored by TLC), the reaction was quenched with water. The aqueous layer was extracted with EtOAc (30 mL × 3). The combined organic layers were washed with brine, dried over Na_2SO_4, filtered and concentrated by rotary evaporation. Then the residue was purified by silica gel column chromatography (petroleum ether/EtOAc = 6/1) to afford the desired substrates **1a-1j**, **1l**, **1s**, **9a** and **9b** as mixtures

of *E*/*Z* isomers. The analytical data of the products are summarized below, and the peaks of minor isomers are indicated by an asterisk (*).

2.3 General procedure for the synthesis of substrate 1k

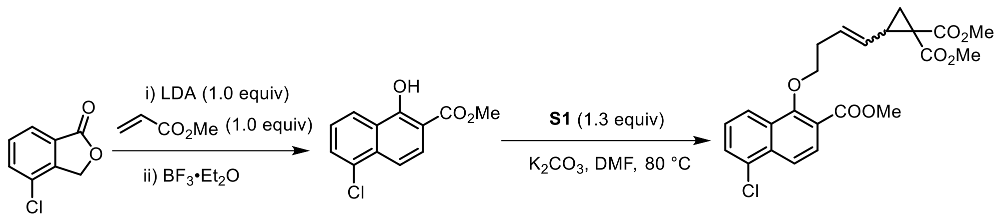

To a stirred solution of DIPEA (1.58 mL, 11.3 mmol) in THF (60 mL) was added *n*-BuLi (4.5 mL, 11.3 mmol, 2.5 M in hexanes) dropwise at −40 °C. The solution was stirred for 15 min at −40 °C, before a solution of 4-chloroisobenzofuran-1(3*H*)-one (1.82 g, 10.8 mmol) in THF (30 mL) was added dropwise at −40 °C. The reaction mixture was stirred for another 15 min, before the addition of a solution of methyl acrylate (974 μL, 10.8 mmol) in THF (5 mL) at −40 °C. The resulting reaction mixture was stirred for 30 min at −40 °C and allowed to warm to room temperature. The reaction was quenched with HCl (aq, 2 M, 100 mL) and extracted with Et_2O (3 × 100 mL). The combined organics were dried by Na_2SO_4, concentrated under reduced pressure and used in the next step without further purification. The crude mixture was then dissolved in CH_2Cl_2 (80 mL) and $BF_3·OEt_2$ (3 mL) was added dropwise. The mixture was stirred at room temperature for 15 min. The reaction was then quenched with H_2O (50 mL), extracted with CH_2Cl_2 (3 × 50 mL), dried (Na_2SO_4) and concentrated under reduced pressure to afford a crude naphthol product without further purification.

The naphthol derivative obtained above (389 mg, 1.9 mmol) was dissolved in DMF (reagent grade, 30.0 mL). After the addition of **S1** (609 mg, 2.1 mmol), K_2CO_3 (524 mg, 3.8 mmol) was added in sequence under argon. Then, the mixture was stirred for 24 h at 80 °C. After completion (monitored by TLC), the reaction was quenched with water. The aqueous layer was extracted with EtOAc (30 mL × 3). The combined organic layers were washed with brine, dried over Na_2SO_4, filtered and concentrated by rotary evaporation. Then the residue was purified by silica gel column chromatography (petroleum ether/EtOAc = 5/1) to afford the desired substrates **1k** as mixtures of *E*/*Z* isomers. The analytical data of the products are summarized below, and the peaks of minor

isomers are indicated by an asterisk (*).

2.4 General procedure for the synthesis of substrates 1m-1p

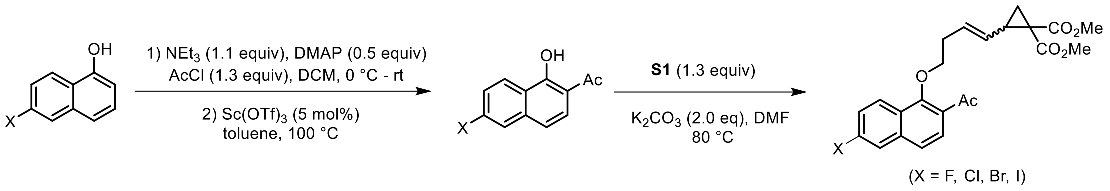

To a stirred solution of the respective naphthol (1 equiv), DMAP (0.5 equiv), Et_3N (1.1 equiv) and CH_2Cl_2 (0.3 M) at 0 °C was added the respective acid chloride (6.5 mmol, 1.3 equiv) dropwise. The reaction mixture was warmed to room temperature and stirred for 1–2 h. The reaction was then quenched with HCl (aq, 1 M, 10 mL) and extracted with CH_2Cl_2 (3 × 10 mL). The combined organics were washed with $NaHCO_3$ (20 mL), dried over $MgSO_4$, concentrated under reduced pressure to afford the acylated naphthol product. To an oven-dried 100 mL round bottom flask was charged $Sc(OTf)_3$ (5 mol%), the respective acylated naphthol (4.0 mmol, 1 equiv) and toluene (8 mL, anhydrous). the reaction mixture was stirred at 100 °C for 16 h. The reaction was then quenched with water (10 mL) and extracted with CH_2Cl_2 (3 × 10 mL). The combined organics were dried over Na_2SO_4, concentrated under reduced pressure a to afford the crude naphthol without further purification.

The naphthol derivatives obtained above were dissolved in DMF (reagent grade, 30.0 mL). After the addition of **S1** (1.3 equiv), K_2CO_3 (2.0 equiv) was added in sequence under argon. Then, the mixture was stirred for 24 h at 80 °C. After completion (monitored by TLC), the reaction was quenched with water. The aqueous layer was extracted with EtOAc (30 mL × 3). The combined organic layers were washed with brine, dried over Na_2SO_4, filtered and concentrated by rotary evaporation. Then the residue was purified by silica gel column chromatography (petroleum ether/EtOAc = 3/1) to afford the desired substrates **1m-1p** as mixtures of *E*/*Z* isomers. The analytical data of the products are summarized below, and the peaks of minor isomers are indicated by an asterisk (*).

2.5 General procedure for the synthesis of substrate 1q

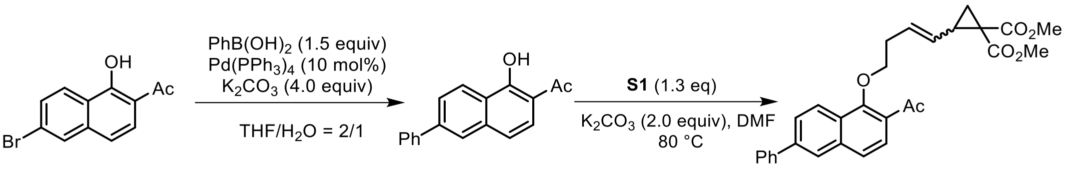

In a round bottom flask, 6-Br naphthol derivative (396 mg, 1.5 mmol) was dissolved in THF/H_2O = 2/1 (4.8 mL). After the addition of $PhB(OH)_2$ (274 mg, 2.25 mmol), $Pd(PPh_3)_4$ (173 mg, 0.15 mmol), K_2CO_3 (828 g, 6 mmol) was added in sequence under argon. Then, the mixture was stirred for 24 h at 80 °C. After completion (monitored by TLC), the reaction was quenched with water. The aqueous layer was extracted with EtOAc (30 mL × 3). The combined organic layers were washed with brine, dried over Na_2SO_4, filtered and concentrated by rotary evaporation to afford the naphthol product without further purification.

The naphthol derivative obtained above (299 mg, 1.1 mmol) was dissolved in DMF (reagent grade, 10.0 mL). After the addition of **S1** (0.63 g, 6.1 mmol), K_2CO_3 (320 mg, 2.3 mmol) was added in sequence under argon. Then, the mixture was stirred for 24 h at 80 °C. After completion (monitored by TLC), the reaction was quenched with water. The aqueous layer was extracted with EtOAc (30 mL × 3). The combined organic layers were washed with brine, dried over Na_2SO_4, filtered and concentrated by rotary evaporation. Then the residue was purified by silica gel column chromatography (petroleum ether/EtOAc = 3/1) to afford the desired substrates **1q** as mixtures of *E*/*Z* isomers. The analytical data of the products are summarized below, and the peaks of minor isomers are indicated by an asterisk (*).

2.6 General procedure for the synthesis of substrate 1r

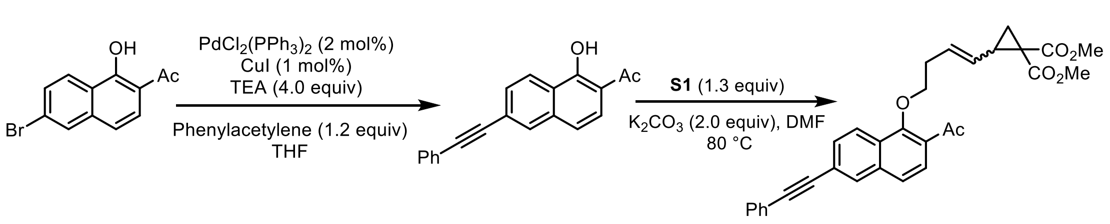

In a round bottom flask, 6-Br naphthol derivative (1.06 g, 4 mmol) was dissolved in THF (20 mL). After the addition of $PdCl_2(PPh_3)_2$ (56.2 mg, 0.08 mmol), CuI (7.6 mg, 0.04 mmol), TEA (2.2 mL, 16.0 mmol) was added in sequence under argon. Then, the

mixture was stirred for 18 h at 60 °C. After completion (monitored by TLC), the reaction was quenched with water. The aqueous layer was extracted with EtOAc (30 mL × 3). The combined organic layers were washed with brine, dried over Na_2SO_4, filtered and concentrated by rotary evaporation to afford the crude naphthol without further purification.

The naphthol derivative obtained above (330 mg, 1.2 mmol) was dissolved in DMF (reagent grade, 10.0 mL). After the addition of **S1** (368 mg, 1.3 mmol), K_2CO_3 (317 mg, 2.3 mmol) was added in sequence under argon. Then, the mixture was stirred for 24 h at 80 °C. After completion (monitored by TLC), the reaction was quenched with water. The aqueous layer was extracted with EtOAc (30 mL × 3). The combined organic layers were washed with brine, dried over Na_2SO_4, filtered and concentrated by rotary evaporation. Then the residue was purified by silica gel column chromatography (petroleum ether/EtOAc = 3/1) to afford the desired substrates **1r** as mixtures of *E*/*Z* isomers. The analytical data of the products are summarized below, and the peaks of minor isomers are indicated by an asterisk (*).

2.7 General procedure for the synthesis of substrate 1t

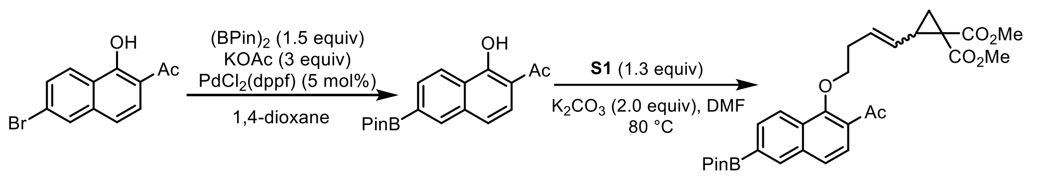

In a round bottom flask, 6-Br naphthol derivative (1.06 g, 4 mmol) was dissolved in dioxane (30 mL). After the addition of $(Bpin)_2$ (1.5 equiv), KOAc (3 equiv), $PdCl_2$(dppf) (5 mol%) was added in sequence under argon. Then, the mixture was stirred for 18 h at 80 °C. After completion (monitored by TLC), the reaction was quenched with water. The aqueous layer was extracted with EtOAc (30 mL × 3). The combined organic layers were washed with brine, dried over Na_2SO_4, filtered and concentrated by rotary evaporation to afford the crude naphthol without further purification.

The naphthol derivative obtained above (204 mg, 0.7 mmol) was dissolved in DMF (reagent grade, 10.0 mL). After the addition of **S1** (210 mg, 0.7 mmol), K_2CO_3 (180 mg, 1.3 mmol) was added in sequence under argon. Then, the mixture was stirred for

24 h at 80 °C. After completion (monitored by TLC), the reaction was quenched with water. The aqueous layer was extracted with EtOAc (30 mL × 3). The combined organic layers were washed with brine, dried over Na_2SO_4, filtered and concentrated by rotary evaporation. Then the residue was purified by silica gel column chromatography (petroleum ether/EtOAc = 4/1) to afford the desired substrates **1t** as mixtures of *E*/*Z* isomers. The analytical data of the products are summarized below, and the peaks of minor isomers are indicated by an asterisk (*).

2.8 Characterization data of substrates

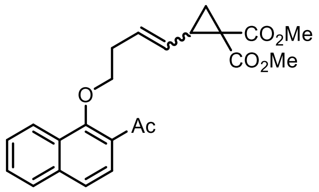

1a, brown oil, 0.34 g, 43% yield (2.0 mmol scale), *E*/*Z* = 4/1. ^{1}H NMR (400 MHz, $CDCl_3$) δ 8.22-8.17 (m, 1H), 7.84-7.82 (m, 1H), 7.70-7.66 (m, 1H), 7.61-7.55 (m, 3H), 5.92-5.72 (m, 1H), 5.29 (dd, *J* = 15.2, 8.4 Hz, 1H), 5.20-5.14 (m, 1H*), 4.13-3.99 (m, 2H), 3.74 (s, 3H), 3.67 (s, 3H), 2.89-2.28 (m, 3H), 2.75 (s, 3H*), 2.73 (s, 3H), 1.74-1.55 (m, 2H). ^{13}C NMR (100 MHz, $CDCl_3$) δ 200.4, 200.3, 169.8, 167.73, 167.68, 155.8, 155.7, 136.53, 136.49, 130.2, 129.9, 128.18, 128.15, 128.04, 128.01, 127.82, 127.75, 127.4, 126.4, 125.21, 125.18, 123.9, 123.1, 75.9, 75.7, 52.6, 52.5, 52.42, 52.40, 35.5, 35.4, 33.3, 30.64, 30.61, 28.8, 26.4, 21.7, 20.8, 20.6. IR (thin film) ν_{max} (cm^{-1}) = 3000, 2951, 1723, 1673, 1622, 1595, 1566, 1502, 1434, 1398, 1358, 1207, 1126, 1072, 964, 899, 874, 817, 751, 730, 688. HRMS (ESI) calcd for $C_{23}H_{24}NaO_6$ [M+Na]$^+$: 419.1465. Found: 419.1471.

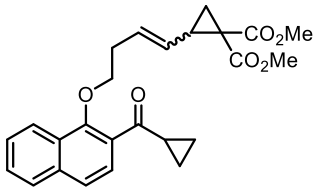

1b, yellow oil, 1.10 g, 56% yield (4.6 mmol scale), *E*/*Z* = 4.6/1. ^{1}H NMR (400 MHz, $CDCl_3$) δ 8.27-8.22 (m, 1H), 7.84-7.81 (m, 1H), 7.64-7.53 (m, 4H), 5.90-5.74 (m, 1H), 5.26 (dd, *J* = 15.2, 8.4 Hz, 1H), 5.18-5.10 (m, 1H*), 4.12-4.01 (m, 2H), 3.74 (s, 3H), 3.69 (s, 3H), 3.02-2.95 (m, 1H), 2.85-2.53 (m, 3H), 1.73-1.55 (m, 2H), 1.33-1.30 (m,

2H), 1.09-1.04 (m, 2H). ^{13}C NMR (100 MHz, $CDCl_3$) δ 203.6, 169.8, 167.7, 155.6, 136.3, 130.3, 130.0, 128.6, 128.2, 127.9, 127.7, 127.5, 127.1, 126.4, 125.3, 123.7, 123.0, 76.0, 75.8, 52.5, 52.4, 35.4, 33.4, 30.7, 28.9, 26.4, 21.7, 21.1, 20.6, 12.6, 12.5. IR (thin film) ν_{max} (cm^{-1}) = 3005, 2951, 1723, 1660, 1622, 1594, 1502, 1433, 1383, 1359, 1264, 1206, 1126, 1089, 1001, 964, 820, 758, 728, 704, 675. HRMS (ESI) calcd for $C_{25}H_{26}NaO_6$ $[M+Na]^+$: 445.1622. Found: 445.1625.

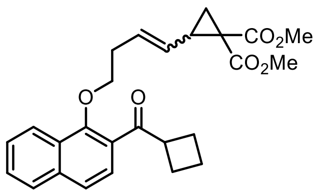

1c, yellow oil, 0.63 g, 30% yield (4.9 mmol scale), *E*/*Z* = 5.4/1. ^{1}H NMR (400 MHz, $CDCl_3$) δ 8.23-8.16 (m, 1H), 7.84-7.81 (m, 1H), 7.63-7.53 (m, 4H), 5.92-5.76 (m, 1H), 5.28 (dd, *J* = 15.6, 8.4 Hz, 1H), 5.19-5.12 (m, 1H*), 4.21-4.11 (m, 1H), 4.07-3.89 (m, 2H), 3.76-3.68 (m, 6H), 2.88-2.57 (m, 3H), 2.41-2.20 (m, 4H), 2.09-1.82 (m, 2H), 1.75-1.57 (m, 2H). ^{13}C NMR (100 MHz, $CDCl_3$) δ 204.9, 169.8, 167.6, 154.8, 136.1, 130.3, 130.0, 128.0, 127.72, 127.70, 127.5, 127.2, 127.1, 126.3, 125.2, 123.8, 123.6, 123.0, 75.9, 75.7, 52.52, 52.46, 52.4, 52.3, 45.02, 44.98, 35.4, 33.3, 30.7, 28.8, 26.4, 25.1, 21.6, 20.5, 17.6. IR (thin film) ν_{max} (cm^{-1}) = 2949, 2868, 1723, 1669, 1621, 1594, 1565, 1397, 1330, 1265, 1207, 1125, 1068, 965, 900, 873, 822, 780, 755, 703, 572. HRMS (ESI) calcd for $C_{26}H_{28}NaO_6$ $[M+Na]^+$: 459.1778. Found: 459.1774.

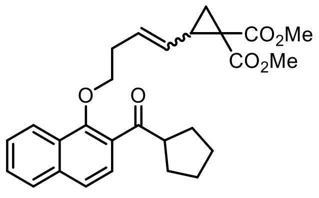

1d, yellow oil, 1.00 g, 47% yield (4.7 mmol scale), *E*/*Z* = 4.6/1. ^{1}H NMR (400 MHz, $CDCl_3$) δ 8.19-8.17 (m, 1H), 7.85-7.82 (m, 1H), 7.63-7.48 (m, 4H), 5.89-5.68 (m, 1H), 5.25 (dd, *J* = 15.6, 8.4 Hz, 1H), 5.18-5.09 (m, 1H*), 4.07-3.97 (m, 2H), 3.89-3.82 (m, 1H), 3.74 (s, 3H), 3.70 (s, 3H), 2.83-2.54 (m, 3H), 1.90-1.85 (m, 4H), 1.75-1.57 (m, 6H). ^{13}C NMR (100 MHz, $CDCl_3$) δ 207.7, 170.0, 167.9, 154.1, 136.0, 130.5, 129.3, 128.2, 127.9, 127.7, 126.5, 125.3, 124.0, 123.1, 76.1, 52.7, 52.6, 50.8, 35.6, 33.5, 30.9, 29.7, 26.1, 21.8, 20.8. IR (thin film) ν_{max} (cm^{-1}) = 2951, 2868, 1723, 1679, 1565, 1435,

1396, 1356, 1330, 1265, 1207, 1126, 1092, 1004, 964, 900, 873, 820, 759, 705, 571. HRMS (ESI) calcd for $C_{27}H_{30}NaO_6$ $[M+Na]^+$: 473.1935. Found: 473.1937.

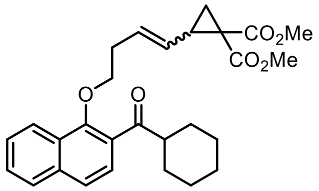

1e, yellow oil, 0.57 g, 31% yield (4.1 mmol scale), *E*/*Z* = 4.4/1. ^{1}H NMR (400 MHz, $CDCl_3$) δ 8.21-8.16 (m, 1H), 7.84-7.82 (m, 1H), 7.61 (d, *J* = 8.4 Hz, 1H), 7.57-7.44 (m, 3H), 5.91-5.74 (m, 1H), 5.26 (dd, *J* = 15.6, 8.4 Hz, 1H), 5.19-5.11 (m, 1H*), 4.07-3.96 (m, 2H), 3.74 (s, 3H), 3.70 (s, 3H), 3.41-3.31 (m, 1H), 2.85-2.55 (m, 3H), 1.92-1.88 (m, 2H), 1.82-1.64 (m, 4H), 1.61-1.58 (m, 1H), 1.50-1.20 (m, 5H). ^{13}C NMR (100 MHz, $CDCl_3$) δ 208.2, 169.9, 167.7, 153.8, 135.9, 130.4, 130.1, 128.8, 128.0, 127.8, 127.62, 127.55, 127.1, 126.4, 125.2, 123.9, 122.9, 76.0, 75.8, 52.6, 52.4, 49.7, 35.5, 33.4, 30.8, 28.9, 28.9, 26.5, 25.9, 25.69, 25.68, 21.7, 20.7. IR (thin film) ν_{max} (cm^{-1}) = 2927, 2852, 1724, 1678, 1621, 1594, 1566, 1435, 1358, 1333, 1207, 1126, 1087, 985, 964, 896, 845, 821, 798, 766, 705. HRMS (ESI) calcd for $C_{28}H_{32}NaO_6$ $[M+Na]^+$: 487.2091. Found: 487.2101.

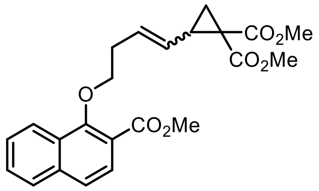

1f, yellow oil, 1.11 g, 57% yield (4.7 mmol scale), *E*/*Z* = 4.7/1. ^{1}H NMR (400 MHz, $CDCl_3$) δ 8.29-8.23 (m, 1H), 7.87-7.83 (m, 2H), 7.61-7.53 (m, 3H), 5.96-5.81 (m, 1H), 5.29 (dd, *J* = 15.2, 8.4 Hz, 1H), 5.20-5.09 (m, 1H*), 4.19-4.08 (m, 2H), 3.96 (s, 3H), 3.74 (s, 3H), 3.70 (s, 3H), 2.92-2.57 (m, 3H), 1.75-1.58 (m, 2H). ^{13}C NMR (100 MHz, $CDCl_3$) δ 170.1, 167.9, 166.7, 157.1, 136.6, 130.9, 128.7, 128.3, 127.7, 127.3, 126.6, 126.5, 123.7, 123.5, 119.1, 116.5, 75.3, 52.7, 52.5, 52.2, 35.5, 33.5, 31.0, 28.1, 25.8, 21.5, 20.8. IR (thin film) ν_{max} (cm^{-1}) = 2998, 2951, 1719, 1624, 1503, 1400, 1360, 1334, 1237, 1207, 1151, 1127, 1081, 965, 899, 873, 826, 800, 767, 724, 431. HRMS (ESI) calcd for $C_{23}H_{24}NaO_7$ $[M+Na]^+$: 435.1414. Found: 435.1416.

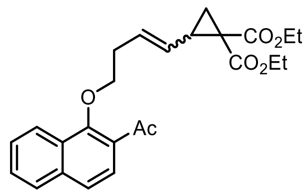

1g, brown oil, 0.67 g, 39% yield (4.2 mmol scale), E/Z = 2.9/1. ^{1}H NMR (400 MHz, $CDCl_3$) δ 8.23-8.17 (m, 1H), 7.85-7.80 (m, 1H), 7.70-7.66 (m, 1H), 7.61-7.51 (m, 3H), 5.91-5.75 (m, 1H), 5.30 (dd, J = 15.2, 8.4 Hz, 1H), 5.21-5.09 (m, 1H*), 4.28-3.98 (m, 6H), 2.89-2.53 (m, 6H), 1.70-1.51 (m, 2H), 1.29-1.19 (m, 6H). ^{13}C NMR (100 MHz, $CDCl_3$) δ 200.3, 169.4, 167.3, 155.7, 136.5, 129.80, 129.77, 128.2, 128.1, 128.04, 128.01, 127.8, 126.4, 125.2, 123.9, 123.2, 76.0, 75.8, 61.4, 61.2, 35.8, 35.7, 35.6, 33.3, 30.62, 30.57, 30.2, 29.7, 28.8, 21.4, 20.3, 14.02, 14.00, 13.90, 13.88. IR (thin film) ν_{max} (cm^{-1}) = 2981, 2936, 1719, 1674, 1595, 1427, 1394, 1316, 1269, 1244, 1127, 1111, 1072, 1019, 965, 912, 868, 817, 751, 728, 688, 664. HRMS (ESI) calcd for $C_{25}H_{28}NaO_6$ $[M+Na]^+$: 447.1778. Found: 447.1784.

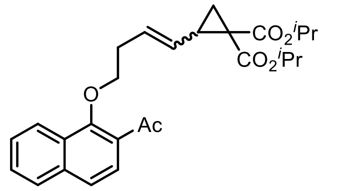

1h, yellow oil, 2.20 g, 78% yield (6.2 mmol scale), E/Z = 4.3/1. ^{1}H NMR (400 MHz, $CDCl_3$) δ 8.16-8.08 (m, 1H), 7.77-7.73 (m, 1H), 7.62-7.58 (m, 1H), 7.53-7.45 (m, 3H), 5.82-5.63 (m, 1H), 5.20 (dd, J = 15.2, 8.4 Hz, 1H), 5.12-5.05 (m, 1H*), 5.01-4.94 (m, 2H), 4.04-3.91 (m, 2H), 2.83-2.43 (m, 3H), 2.68 (s, 3H*), 2.65 (s, 3H), 1.60-1.38 (m, 2H), 1.19-1.14 (m, 9H), 1.11-1.07 (m, 3H). ^{13}C NMR (100 MHz, $CDCl_3$) δ 200.4, 169.2, 167.0, 155.8, 136.6, 129.5, 129.2, 128.31, 128.27, 128.11, 128.09, 127.9, 126.5, 125.3, 124.0, 123.2, 76.1, 69.0, 68.7, 36.0, 33.4, 30.7, 29.8, 21.8, 21.6, 21.6, 21.4, 20.1. IR (thin film): ν_{max} (cm^{-1}) = 2980, 2936, 1714, 1675, 1596, 1458, 1427, 1358, 1335, 1311, 1270, 1244, 1146, 1097, 1072, 966, 900, 817, 751, 729, 688, 664. HRMS (ESI) calcd for $C_{27}H_{32}NaO_6$ $[M+Na]^+$: 475.2091. Found: 475.2092.

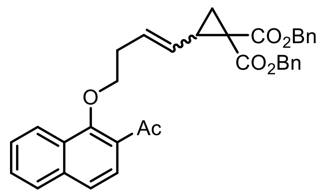

1i, yellow oil, 0.90 g, 36% yield (4.5 mmol scale), *E*/*Z* = 4.6/1. ^{1}H NMR (400 MHz, $CDCl_3$) δ 8.18-8.15 (m, 1H), 7.84-7.81 (m, 1H), 7.70-7.66 (m, 1H), 7.60 (d, *J* = 8.4 Hz, 1H), 7.57-7.51 (m, 2H), 7.34-7.26 (m, 5H), 7.22-7.17 (m, 5H), 5.87-5.69 (m, 1H), 5.23-5.17 (m, 2H), 5.13-5.09 (m, 3H), 3.98-3.90 (m, 2H), 2.89-2.69 (m, 3H), 2.67-2.48 (m, 3H), 1.76-1.58 (m, 2H). ^{13}C NMR (100 MHz, $CDCl_3$) δ 200.5, 169.3, 167.2, 155.8, 136.6, 135.39, 135.35, 130.3, 128.4, 128.32, 128.29, 128.23, 128.15, 127.9, 127.7, 126.5, 125.3, 124.0, 123.3, 76.0, 67.3, 67.2, 35.7, 33.3, 31.0, 30.7, 20.9. IR (thin film) ν_{max} (cm^{-1}) = 3032, 2942, 1720, 1672, 1622, 1595, 1566, 1455, 1427, 1314, 1268, 1244, 1190, 1072, 964, 908, 817, 733, 694, 601, 583, 507. HRMS (ESI) calcd for $C_{35}H_{32}NaO_6$ $[M+Na]^+$: 571.2091. Found: 571.2097.

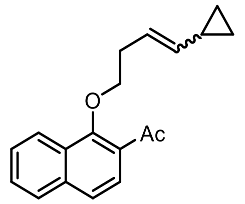

1j, colorless oil, 0.51 g, 43% yield (4.2 mmol scale), *E*/*Z* = 8/1. ^{1}H NMR (400 MHz, $CDCl_3$) δ 8.26-8.19 (m, 1H), 7.81-7.78 (m, 1H), 7.70-7.66 (m, 1H), 7.58-7.50 (m, 3H), 5.63-5.55 (m, 1H), 5.48-5.41 (m, 1H*), 5.15 (dd, *J* = 15.2, 8.4 Hz, 1H), 4.96-4.91 (m, 1H*), 4.05 (t, *J* = 6.8 Hz, 2H*), 3.99 (t, *J* = 6.8 Hz, 2H), 2.79-2.72 (m, 3H), 2.57 (q, *J* = 6.8 Hz, 2H), 1.61-1.56 (m, 1H*), 1.42-1.34 (m, 1H), 0.78-0.61 (m, 2H), 0.36-0.32 (m, 2H). ^{13}C NMR (100 MHz, $CDCl_3$) δ 200.5, 156.0, 137.2, 136.6, 128.3, 128.2, 128.0, 127.8, 126.3, 125.3, 123.8, 123.3, 122.7, 122.4, 76.7, 33.3, 30.7, 30.7, 28.6, 13.5, 9.7, 6.9, 6.3. IR (thin film) ν_{max} (cm^{-1}) = 3002, 2930, 1671, 1622, 1595, 1566, 1458, 1398, 1242, 1191, 1151, 1112, 1046, 961, 870, 814, 749, 727, 687, 663, 616, 565. HRMS (ESI) calcd for $C_{19}H_{20}NaO_2$ $[M+Na]^+$: 303.1356. Found: 303.1351.

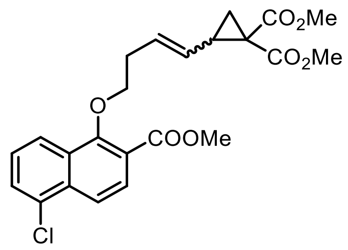

1k, yellow oil, 0.31 g, 37% yield (1.9 mmol scale), *E*/*Z* = 5.9/1. ^{1}H NMR (400 MHz, $CDCl_3$) δ 8.15-8.13 (m, 1H), 7.95-7.93 (m, 1H), 7.88-7.86 (m, 1H), 7.60-7.58 (m, 1H), 7.41-7.37 (m, 1H), 5.86-5.82 (m, 1H), 5.81-5.74 (m, 1H*), 5.29-5.23 (m, 1H), 5.11-5.06 (m, 1H*), 4.04 (*t*, *J* = 6.4 Hz, 2H), 3.93 (s, 3H*), 3.92 (s, 3H), 3.69 (s, 3H), 3.65 (s, 3H), 2.62-2.54 (m, 3H), 1.69-1.65 (m, 1H), 1.62-1.59 (m, 1H*), 1.57-1.53 (m, 1H), ^{13}C NMR (100 MHz, $CDCl_3$) δ 170.0, 167.8, 166.2, 156.9, 133.8, 131.9, 130.7, 130.1, 128.5, 127.7, 127.4, 126.3, 122.8, 119.8, 119.8, 75.5, 75.3, 52.7, 52.6, 52.5, 52.5, 52.3, 35.6, 35.6, 33.4, 30.9, 28.9, 26.7, 21.8, 20.7. IR (thin film) ν_{max} (cm^{-1}) = 2953, 1726, 1592, 1495, 1437, 1401, 1359, 1328, 1262, 1204, 1132, 1103, 1062, 1002, 970, 882, 824, 779. HRMS (ESI) calcd for $C_{23}H_{23}O_7NaCl$ $[M+Na]^+$: 469.1025. Found: 469.1026.

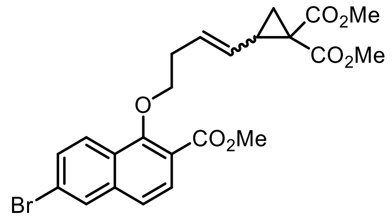

1l, yellow oil, 0.42 g, 39% yield (2.2 mmol scale), *E*/*Z* = 5.3/1. ^{1}H NMR (400 MHz, $CDCl_3$) δ 8.10-8.08 (m, 1H), 7.951-7.946 (m, 1H), 7.84-7.81 (m, 1H), 7.59-7.57 (m, 1H), 7.46-7.44 (m, 1H), 5.91-5.83 (m, 1H), 5.81-5.75 (m, 1H*), 5.30-5.24 (m, 1H), 5.12-5.06 (m, 1H*), 4.34 (t, *J* = 5.2 Hz, 2H), 3.72 (s, 3H), 3.70 (s, 3H*), 3.67 (s, 3H), 2.86-2.73 (m, 3H), 2.63-2.55 (m, 3H), 1.71-1.68 (m, 1H), 1.64-1.60 (m, 1H*), 1.59-1.55 (m, 1H). ^{13}C NMR (100 MHz, $CDCl_3$) δ 170.1, 167.9, 166.4, 157.2, 137.6, 130.8, 130.4*, 123.0, 129.8, 128.1, 127.5, 127.3, 127.2*, 125.7, 123.0, 122.5, 119.4, 75.5, 75.3*, 53.5*, 52.7, 52.6, 52.3. 35.7*, 35.6, 33.5, 31.0, 29.0*, 26.7*, 21.9*, 20.8. IR (thin film) ν_{max} (cm^{-1}) = 3028, 1725, 1619, 1437, 1333, 1273, 1237, 1212, 1185, 1132, 1089, 1004, 968, 880, 832, 793, 733. HRMS (ESI) calcd for $C_{23}H_{23}O_7Na^{79}Br$ $[M+Na]^+$: 513.0519. Found: 513.0514.

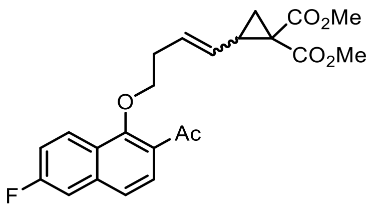

1m, brown oil, 0.33 g, 66% yield (1.2 mmol scale), *E*/*Z* = 4.6/1. ^{1}H NMR (400 MHz, $CDCl_3$) δ 8.20-8.16 (m, 1H), 7.69-7.67 (m, 1H), 7.53-7.51 (m, 1H), 7.44-7.09 (m, 1H), 7.33-7.28 (m, 1H), 6.09-6.03 (m, 1H*), 5.89-5.82 (m, 1H), 5.57-5.51 (m, 1H*), 5.30-5.24 (m, 1H), 4.04-4.01 (m, 2H*), 3.99-3.96 (m, 2H), 3.73 (s, 3H), 3.68 (s, 3H), 2.70 (s, 3H), 2.62-2.56 (s, 3H), 1.72-1.68 (m, 1H), 1.60-1.57 (m, 1H). ^{13}C NMR (100 MHz, $CDCl_3$) δ 200.3, 170.1, 168.0, 163.5, 161.0, 156.2, 138.0, 137.9, 130.3, 128.2, 127.8, 127.0, 126.5, 126.4, 125.4, 123.5, 123.4, 117.1, 116.9, 111.5, 111.3, 52.8, 52.7, 35.6, 33.5, 30.9, 30.7, 20.9. ^{19}F NMR (376 MHz, $CDCl_3$) δ -111.10 ~ -119.16 (m, 1F*), -111.22 ~ -119.29 (m, 1F). IR (thin film) ν_{max} (cm^{-1}) = 2991, 1727, 1677, 1619, 1563, 1437, 1405, 1354, 1332, 1266, 1242, 1211, 1182, 1125, 1088, 1066, 967, 894, 829, 736, 701. HRMS (ESI) calcd for $C_{23}H_{23}O_6FNa$ $[M+Na]^+$: 437.1371. Found: 437.1367.

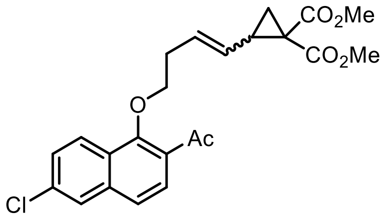

1n, yellow oil, 0.63 g, 49% yield (2.4 mmol scale), *E*/*Z* = 6.2/1. ^{1}H NMR (400 MHz, $CDCl_3$) δ 8.02-7.99 (m, 1H), 7.67-7.66 (m, 1H), 7.58-7.56 (m, 1H), 7.38-7.35 (m, 2H), 5.81-5.73 (m, 1H), 5.70-5.67 (m, 1H*), 5.50-5.44 (m, 1H*), 5.23-5.18 (m, 1H), 3.89-3.85 (m, 2H), 3.65 (s, 3H), 3.61 (s, 3H), 2.61 (s, 3H), 2.53-2.50 (m, 3H), 1.64-1.61 (m, 1H), 1.52-1.48 (m, 1H). ^{13}C NMR (100 MHz, $CDCl_3$) δ 199.7, 169.7, 167.6, 155.7, 137.0, 134.1, 130.1, 128.3, 127.9, 127.3, 126.7, 126.5, 126.4, 125.0, 122.9, 76.2, 52.5, 52.4, 35.4, 33.2, 30.6, 30.5, 20.5. IR (thin film) ν_{max} (cm^{-1}) = 2995, 1728, 1667, 1618, 1437, 1400, 135, 1332, 1267, 1242, 1212, 1182, 1126, 1078, 1066, 967, 894, 829, 736, 702. HRMS (ESI) calcd for $C_{23}H_{23}O_6Na^{35}Cl$ $[M+Na]^+$: 453.1075. Found: 453.1081.

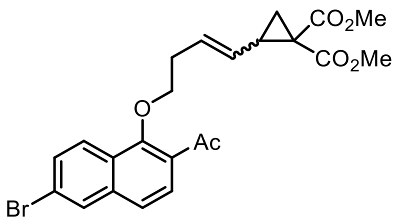

1o, yellow oil, 0.50 g, 56% yield (1.9 mmol scale), *E*/*Z* = 5.3/1. ^{1}H NMR (400 MHz, $CDCl_3$) δ 8.02-7.99 (m, 1H), 7.95 (s, 1H), 7.65-7.63 (m, 1H), 7.59-7.56 (m, 1H), 7.47-7.45 (m, 1H), 5.87-5.79 (m, 1H), 5.77-5.70 (m, 1H*), 5.50-5.44 (m, 1H), 5.15-5.09 (m, 1H*), 3.96-3.93 (m, 2H), 3.71 (s, 3H), 3.67 (s, 3H), 2.68 (s, 3H), 2.60-2.54 (m, 3H), 1.70-1.67 (m, 1H), 1.58-1.55 (m, 1H). ^{13}C NMR (100 MHz, $CDCl_3$) δ 200.2, 170.0, 167.9, 155.9, 137.6, 130.2, 130.05, 130.03, 128.6, 128.1, 126.8, 126.8, 125.2, 123.1, 122.8, 76.4, 52.8, 52.6, 35.6, 33.4, 30.8, 30.7, 20.8. IR (thin film) ν_{max} (cm^{-1}) = 3040, 1729, 1675, 1616, 1437, 1405, 1354, 1332, 1266, 1242, 1211, 1182, 1125, 1088, 1066, 967, 894, 829, 736, 702. HRMS (ESI) calcd for $C_{23}H_{23}O_6Na^{79}Br$ $[M+Na]^+$: 498.3248. Found: 498.3254.

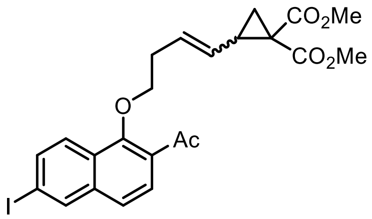

1p, yellow oil, 0.44 g, 53% yield (1.6 mmol scale), *E*/*Z* = 5.8/1. ^{1}H NMR (400 MHz, $CDCl_3$) δ 8.19 (s, 1H), 7.89-7.87 (m, 1H), 7.78-7.75 (m, 1H), 7.66-7.64 (m, 1H), 7.45-7.43 (m, 1H), 5.91-5.84 (m, 1H), 5.82-5.75 (m, 1H*), 5.33-5.28 (m, 1H), 5.19-5.15 (m, 1H*), 3.98-3.95 (m, 2H), 3.75 (s, 3H), 3.71 (s, 3H), 2.71 (s, 3H), 2.62-2.58 (m, 3H), 1.74-1.70 (m, 1H), 1.62-1.58 (m, 1H). ^{13}C NMR (100 MHz, $CDCl_3$) δ 199.6, 169.6, 167.5, 155.6, 137.5, 136.3, 134.9, 130.0, 128.4, 127.8, 126.8, 126.3, 124.7, 122.6, 94.7, 76.1, 52.4, 52.3, 35.3, 33.1, 30.43, 30.41, 20.4. IR (thin film) ν_{max} (cm^{-1}) = 3021, 1721, 1676, 1614, 1436, 1329, 1272, 1240, 1210, 1181, 1124, 1077, 967, 879, 827, 743, 681. HRMS (ESI) calcd for $C_{23}H_{23}O_6NaI$ $[M+Na]^+$: 545.0432. Found: 545.0428.

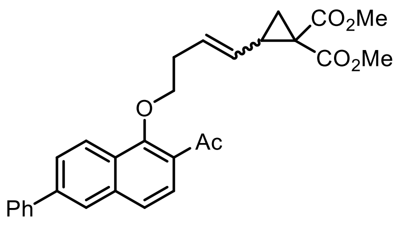

1q, yellow oil, 0.33 g, 63% yield (1.1 mmol scale), *E*/*Z* = 5/1. ^{1}H NMR (400 MHz, $CDCl_3$) δ 8.27-8.25 (m, 1H), 8.031-8.026 (m, 1H), 7.84-7.81 (m, 1H), 7.74-7.70 (m, 3H), 7.67-7.65 (m, 1H), 7.52-7.48 (m, 2H), 7.43-7.38 (m, 1H), 5.94-5.86 (m, 1H), 5.85-5.78 (m, 1H*), 5.34-5.28 (m, 1H), 5.20-5.14 (m, 1H*), 4.06-4.02 (m, 2H), 3.74 (s, 3H), 3.71 (s, 3H), 2.75 (s, 3H), 2.67-2.59 (m, 3H), 1.75-1.72 (m, 1H), 1.62-1.59 (s, 1H). ^{13}C NMR (100 MHz, $CDCl_3$) δ 200.6, 170.1, 168.0, 156.1, 141.1, 140.5, 137.2, 130.5, 129.1, 128.4, 128.1, 128.0, 127.51, 127.45, 126.4, 126.1, 125.9, 124.4, 124.2, 76.3, 52.8, 52.7, 35.7, 33.6, 31.0, 30.9, 20.9. IR (thin film) ν_{max} (cm^{-1}) = 3050, 1727, 1674, 1625, 1463, 1438, 1408, 1341, 1282, 1246, 1210, 1124, 1079, 1040, 1014, 967, 895, 838, 810, 760, 733, 697, 654. HRMS (ESI) calcd for $C_{29}H_{28}O_6Na$ $[M+Na]^+$: 495.1886. Found: 495.1808.

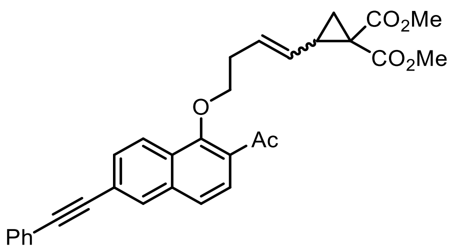

1r, yellow oil, 0.37 g, 65% yield (1.0 mmol scale), *E*/*Z* = 5.7/1. ^{1}H NMR (400 MHz, $CDCl_3$) δ 8.17-8.14 (m, 1H), 8.030-8.026 (m, 1H), 7.74-7.70 (m, 2H), 7.59-7.57 (m, 3H), 7.38-7.36 (m, 3H), 5.91-5.84 (m, 1H), 5.82-5.76 (m, 1H*), 5.32-5.26 (m, 1H), 5.19-5.13 (m, 1H*), 4.02-3.99 (m, 2H), 3.75 (s, 3H), 3.71 (s, 3H), 2.73 (s, 3H), 2.64-2.58 (m, 3H), 1.74-1.71 (m, 1H), 1.62-1.59 (s, 1H). ^{13}C NMR (100 MHz, $CDCl_3$) δ 200.5, 170.1, 168.0, 155.8, 136.4, 131.8, 131.4, 130.4, 129.3, 129.1, 128.7, 128.6, 128.2, 127.7, 126.4, 124.0, 123.6, 123.3, 123.0, 91.4, 89.3, 76.4, 52.8, 52.7, 35.7, 33.6, 30.9, 30.84, 20.9. IR (thin film) ν_{max} (cm^{-1}) = 3080, 1723, 1676, 1653, 1653, 1621, 1559, 1559, 1541, 1457, 1438, 1339, 1211, 1130, 1071, 968, 897, 837, 758, 733, 691. HRMS (ESI) calcd for $C_{31}H_{28}O_6Na$ $[M+Na]^+$: 519.1884. Found: 519.1806.

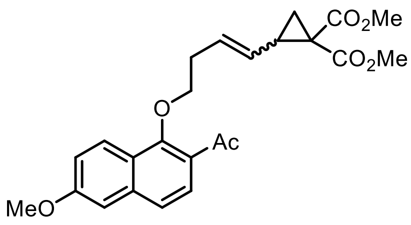

1s, yellow oil, 0.63 g, 37% yield (4.0 mmol scale), *E*/*Z* = 5.6/1. ^{1}H NMR (400 MHz, $CDCl_3$) δ 8.05-8.03 (m, 1H), 7.65-762 (m, 1H), 7.44-7.41 (m, 1H), 7.16-7.13 (m, 1H), 7.07-7.06 (m, 1H), 5.87-5.79 (m, 1H), 5.77-5.72 (m, 1H*), 5.57-5.50 (m, 1H*), 5.27-5.21 (m, 1H), 3.96-3.92 (m, 2H), 3.87 (s, 3H), 3.70 (s, 3H), 3.66 (s, 3H), 2.67 (s, 3H), 2.60-2.53 (m, 3H), 1.69-1.66 (m, 1H), 1.57-1.54 (s, 1H). ^{13}C NMR (100 MHz, $CDCl_3$) δ 199.8, 170.0, 167.8, 159.6, 156.4, 138.6, 130.4, 127.9, 126.4, 126.2, 125.2, 123.3, 122.8, 119.3, 106.0, 76.1, 55.3, 52.7, 52.5, 35.5, 33.4, 30.8, 30.6, 20.7. IR (thin film) ν_{max} (cm^{-1}) = 2996, 1725, 1670, 1622, 1474, 1437, 1416, 1340, 1272, 1243, 1210, 1129, 1078, 1025, 967, 856, 831, 801, 689. HRMS (ESI) calcd for $C_{24}H_{26}O_7Na$ [M+Na]$^+$: 449.1571. Found: 449.1570.

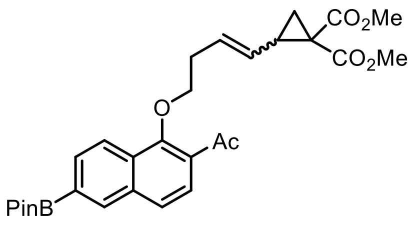

1t, yellow oil, 0.42 g, 52% yield (1.6 mmol scale), *E*/*Z* = 5.8/1. ^{1}H NMR (400 MHz, $CDCl_3$) δ 8.34 (s, 1H), 8.18-8.13 (m, 1H), 7.91-7.89 (m, 1H), 7.74-7.53 (m, 2H), 5.89-5.82 (m, 1H), 5.78-5.76 (m, 1H*), 5.30-5.24 (m, 1H), 4.12-3.97 (m, 2H), 3.73 (s, 3H), 3.69 (s, 3H), 2.72 (s, 3H), 2.62-2.55 (m, 3H), 1.72-1.69 (m, 1H), 1.60-1.57 (m, 1H), 1.38 (s, 12H). ^{13}C NMR (100 MHz, $CDCl_3$) δ 200.8, 169.9, 167.8, 155.5, 136.0, 135.9, 131.2, 130.3, 129.7, 129.3, 127.9, 125.1, 124.7, 122.2, 84.1, 76.1, 52.6, 52.5, 35.5, 33.4, 30.8, 24.8, 20.7. IR (thin film) ν_{max} (cm^{-1}) = 3080, 1729, 1669, 1629, 1475, 1430, 1410, 1340, 1273, 1244, 1217, 1120, 1070, 1021, 966, 856, 832, 801, 688. HRMS (ESI) calcd for $C_{29}H_{35}BO_8Na$ [M+Na]$^+$: 545.2425. Found: 545.2419.

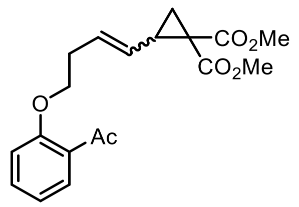

9a (prepared from the corresponding phenol derivatives), yellow oil, 0.34 g, 21% yield (4.7 mmol scale), *E/Z* = 7.8/1. ^{1}H NMR (400 MHz, $CDCl_3$) δ 7.73 (d, *J* = 7.6 Hz, 1H), 7.43 (t, *J* = 6.8 Hz, 1H), 6.97 (t, *J* = 7.6 Hz, 1H), 6.92 (d, *J* = 8.4 Hz, 1H), 5.87-5.80 (m, 1H), 5.74-5.68 (m, 1H*), 5.26 (dd, *J* = 15.6, 8.8 Hz, 1H), 5.10-5.05 (m, 1H*), 4.15-4.02 (m, 2H), 3.73 (s, 3H), 3.71 (s, 3H), 2.79-2.55 (m, 3H), 2.61 (s, 3H), 1.71-1.56 (m, 2H). ^{13}C NMR (100 MHz, $CDCl_3$) δ 199.4, 169.7, 167.6, 157.9, 133.5, 130.4, 130.2, 127.9, 127.4, 120.4, 112.0, 67.6, 52.5, 52.3, 35.3, 32.2, 31.9, 30.6, 20.5. IR (thin film) ν_{max} (cm^{-1}) = 3089, 1727, 1663, 1620, 1470, 1431, 1418, 1347, 1273, 1244, 1215, 1126, 1077, 1027, 962, 856, 837, 805, 667. HRMS (ESI) calcd for $C_{19}H_{22}O_6Na$ $[M+Na]^+$: 369.1416. Found: 369.1512.

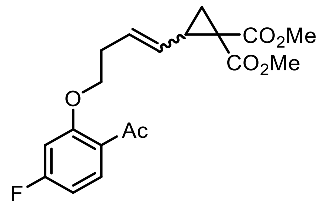

9b (prepared from the corresponding phenol derivatives), yellow oil, 0.32 g, 18% yield (5.0 mmol scale), *E/Z* = 8.3/1. ^{1}H NMR (400 MHz, $CDCl_3$) δ 7.80-7.76 (m, 1H), 6.67-6.57 (m, 2H), 5.82-5.75 (m, 1H), 5.69-5.63 (m, 1H*), 5.26-5.20 (m, 1H), 5.08-5.03 (m, 1H*), 4.02-3.99 (m, 2H), 3.70 (s, 3H), 3.69 (s, 3H), 2.75-2.30 (m, 6H), 1.66-1.63 (m, 1H), 1.57-1.53 (m, 1H). δ ^{13}C NMR (100 MHz, $CDCl_3$) δ 197.7, 169.9, 167.9, 167.5, 165.0, 160.1, 160.0, 132.9, 132.8, 130.2, 129.9, 128.1, 127.6, 124.4, 124.3, 107.8, 107.6, 100.3, 100.3, 100.1, 100.0, 68.31, 68.1, 52.9, 52.8, 52.7, 52.6, 35.7, 35.6, 32.3, 32.2, 32.1, 30.8, 27.8, 26.5, 21.8, 20.8. ^{19}F NMR (376 MHz, $CDCl_3$) δ -103.7 ~ -103.8 (m, 1F). IR (thin film) ν_{max} (cm^{-1}) = 3100, 1740, 1669, 1621, 1479, 1439, 1418, 1346, 1273, 1241, 1211, 1122, 1073, 1024, 958, 840, 830, 801, 651. HRMS (ESI) calcd for $C_{19}H_{21}FO_6Na$ $[M+Na]^+$: 387.1322. Found: 387.1316.

2.2 Procedure for the dearomative [5 + 4] cycloadditions of naphthalene derivatives (using 1a as an example)

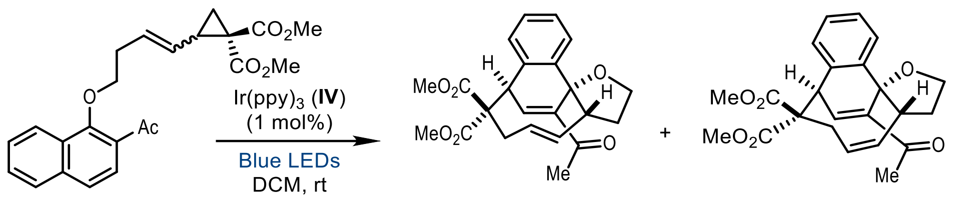

To a flame-dried sealed tube were added **1a** (79 mg, 0.2 mmol), photosensitizer **IV** (1.3 mg, 0.002 mmol) and anhydrous DCM (2.0 mL). The reaction mixture was degassed *via* freeze-pump-thaw for 3 cycles. After the reaction mixture was thoroughly degassed, the vial was sealed and positioned approximately 10 cm from blue LEDs (24 W, λ_{max} = 455 nm). Then the reaction mixture was stirred at room temperature for 24 h (monitored by TLC) under argon atmosphere. Afterwards, the reaction mixture was concentrated by rotary evaporation. Then, the residue was purified by silica gel column chromatography (petroleum ether/acetone = 6/1) to afford the desired major product *E*-**2a** and minor product *Z*-**2a**.

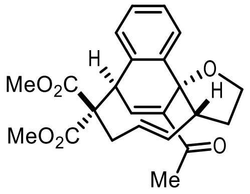

E-**2a**, white solid, 68.1 mg, 86% yield, m.p. = 124.9-126.6 °C, *E*/*Z* = 7.7/1. ^{1}H NMR (400 MHz, $CDCl_3$) δ 7.54 (d, *J* = 8.0 Hz, 1H), 7.33 (t, *J* = 7.2 Hz, 1H), 7.16 (t, *J* = 7.2 Hz, 1H), 6.94 (d, *J* = 8.0 Hz, 1H), 6.79 (d, *J* = 5.6 Hz, 1H), 5.29 (dd, *J* = 16.0, 8.4 Hz, 1H), 5.13-5.05 (m, 1H), 4.54 (d, *J* = 5.6 Hz, 1H), 4.39 (t, *J* = 8.0 Hz, 1H), 4.31-4.24 (m, 1H), 3.84 (s, 3H), 3.79 (s, 3H), 3.07 (dd, *J* = 12.8, 2.8 Hz, 1H), 2.60-2.48 (m, 2H), 2.33 (s, 3H), 2.22 (t, *J* = 12.4 Hz, 1H), 2.13-2.09 (m, 1H). ^{13}C NMR (100 MHz, $CDCl_3$) δ 202.3, 171.3, 171.0, 147.9, 143.3, 135.3, 134.2, 133.3, 127.9, 126.6, 126.4, 126.3, 124.4, 89.1, 72.3, 68.5, 55.4, 52.8, 52.5, 44.3, 39.0, 30.5, 29.8. IR (thin film) ν_{max} (cm^{-1}) = 2957, 2922, 2855, 1729, 1694, 1482, 1434, 1358, 1259, 1228, 1179, 1131, 1041, 999,

899, 845, 789, 764. HRMS (ESI) calcd for $C_{23}H_{24}O_6Na$ [M+Na]$^+$: 419.1465. Found: 419.1467.

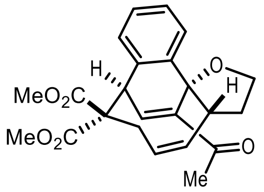

Z-**2a**, colorless oil, 7.7 mg, 10% yield. ^{1}H NMR (400 MHz, $CDCl_3$) δ 7.58 (dd, *J* = 8.0, 1.4 Hz, 1H), 7.35 (td, *J* = 7.6, 1.3 Hz, 1H), 7.26 (d, *J* = 6.4 Hz, 1H), 7.15 (td, *J* = 7.6, 1.3 Hz, 1H), 6.78-6.70 (m, 1H), 5.50-5.46 (m, 1H), 5.43-5.36 (m, 1H), 4.53 (d, *J* = 6.4 Hz, 1H), 4.50-4.33 (m, 2H), 3.83 (s, 3H), 3.80 (s, 3H), 3.27-3.14 (m, 1H), 2.74-2.62 (m, 1H), 2.55-2.40 (m, 2H), 2.25 (s, 3H), 1.85-1.73 (m, 1H). ^{13}C NMR (100 MHz, $CDCl_3$) δ 199.5, 170.6, 169.7, 147.3, 147.0, 138.8, 135.4, 130.8, 128.3, 127.2, 126.4, 126.0, 124.5, 88.2, 71.9, 65.0, 54.7, 53.0, 52.8, 44.6, 34.6, 30.2, 28.8. IR (thin film) ν_{max} (cm^{-1}) = 2991, 1722, 1599, 1551, 1390, 1292, 1241, 1166, 913, 783, 720. HRMS (ESI) calcd for $C_{23}H_{24}O_6Na$ [M+Na]$^+$: 419.1573. Found: 419.1565.

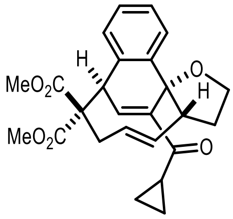

E-**2b**, yellow oil, 61.9 mg, 74% yield, *E*/*Z* = 7.2/1. ^{1}H NMR (400 MHz, $CDCl_3$) δ 7.56 (d, *J* = 8.0 Hz, 1H), 7.34 (t, *J* = 7.2 Hz, 1H), 7.17 (t, *J* = 7.2 Hz, 1H), 6.96 (d, *J* = 7.6 Hz, 1H), 6.78 (d, *J* = 5.2 Hz, 1H), 5.34 (dd, *J* = 16.0, 9.2 Hz, 1H), 5.14-5.06 (m, 1H), 4.57 (d, *J* = 5.2 Hz, 1H), 4.37-4.24 (m, 2H), 3.84 (s, 3H), 3.80 (s, 3H), 3.07 (dd, *J* = 12.8, 2.8 Hz, 1H), 2.57-2.46 (m, 2H), 2.31-2.23 (m, 2H), 2.16-2.08 (m, 1H), 1.13-1.05 (m, 2H), 0.97-0.86 (m, 2H). ^{13}C NMR (100 MHz, $CDCl_3$) δ 205.3, 171.6, 171.1, 148.9, 143.3, 135.6, 134.6, 132.1, 128.0, 126.7, 126.6, 126.3, 124.5, 89.2, 72.2, 68.7, 55.3, 52.9, 52.6, 44.4, 39.1, 29.9, 21.1, 12.1, 12.0. IR (thin film) ν_{max} (cm^{-1}) = 3005, 2929, 2859, 1727, 1671, 1481, 1433, 1339, 1228, 1201, 1177, 1127, 1042, 998, 895, 820, 786, 763. HRMS (ESI) calcd for $C_{25}H_{26}O_6Na$ [M+Na]$^+$: 445.1622. Found: 445.1621.

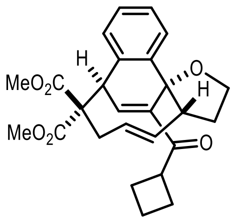

E-**2c**, yellow solid, 70.2 mg, 74% yield, m.p. = 147.5-149.1 °C, *E*/*Z* = 6.7/1. ^{1}H NMR (400 MHz, $CDCl_3$) δ 7.54 (d, *J* = 7.6 Hz, 1H), 7.33 (t, *J* = 7.2 Hz, 1H), 7.16 (t, *J* = 7.2 Hz, 1H), 6.93 (d, *J* = 7.6 Hz, 1H), 6.66 (d, *J* = 5.6 Hz, 1H), 5.31 (dd, *J* = 16.0, 9.6 Hz, 1H), 5.12-5.04 (m, 1H), 4.50 (d, *J* = 5.6 Hz, 1H), 4.40 (t, *J* = 8.4 Hz, 1H), 4.31-4.25 (m, 1H), 3.84 (s, 3H), 3.79 (s, 3H), 3.68-3.59 (m, 1H), 3.07 (dd, *J* = 12.4, 2.8 Hz, 1H), 2.71-2.60 (m, 1H), 2.56-2.48 (m, 1H), 2.33-2.26 (m, 1H), 2.24-2.00 (m, 5H), 1.97-1.86 (m, 1H), 1.81-1.72 (m, 1H). ^{13}C NMR (100 MHz, $CDCl_3$) δ 205.4, 171.5, 171.2, 146.9, 143.5, 135.6, 134.4, 132.7, 128.0, 126.6, 126.5, 126.3, 124.6, 89.2, 72.5, 68.6, 55.6, 52.9, 52.6, 44.9, 44.4, 39.2, 29.8, 25.1, 24.8, 17.5. IR (thin film) ν_{max} (cm^{-1}) = 2950, 2857, 1728, 1677, 1433, 1233, 1210, 1182, 1131, 1064, 1042, 1002, 959, 931, 896, 824, 790, 763. HRMS (ESI) calcd for $C_{26}H_{28}O_6Na$ $[M+Na]^+$: 459.1778. Found: 459.1779.

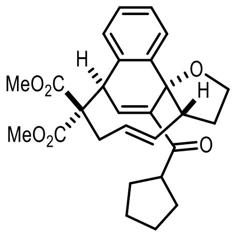

E-**2d**, white solid, 64.0 mg, 71% yield, m.p. = 156.2-157.3 °C, *E*/*Z* = 7.0/1. ^{1}H NMR (400 MHz, $CDCl_3$) δ 7.53 (d, *J* = 8.0 Hz, 1H), 7.33 (t, *J* = 7.6 Hz, 1H), 7.17 (t, *J* = 7.6 Hz, 1H), 6.95 (d, *J* = 8.0 Hz, 1H), 6.61 (d, *J* = 5.2 Hz, 1H), 5.37 (dd, *J* = 16.0, 9.6 Hz, 1H), 5.13-5.05 (m, 1H), 4.52 (d, *J* = 5.6 Hz, 1H), 4.33-4.23 (m, 2H), 3.84 (s, 3H), 3.79 (s, 3H), 3.33-3.25 (m, 1H), 3.06 (dd, *J* = 12.8, 2.8 Hz, 1H), 2.72-2.61 (m, 1H), 2.56-2.49 (m, 1H), 2.23 (t, *J* = 12.4 Hz, 1H), 2.12-2.06 (m, 1H), 1.94-1.85 (m, 1H), 1.76-1.62 (m, 4H), 1.61-1.47 (m, 3H). ^{13}C NMR (100 MHz, $CDCl_3$) δ 208.0, 171.6, 171.2, 148.7, 143.5, 135.6, 134.5, 131.0, 128.0, 126.6, 126.5, 126.3, 124.5, 89.4, 72.4, 68.6, 55.7, 52.9, 52.6, 50.7, 44.3, 39.1, 29.9, 29.7, 28.2, 26.0, 25.7. IR (thin film) ν_{max} (cm^{-1}) = 2952, 2852, 1731, 1681, 1429, 1229, 1207, 1182, 1043, 990, 950, 899, 820, 788, 762, 713, 680, 646. HRMS (ESI) calcd for $C_{27}H_{30}O_6Na$ $[M+Na]^+$: 473.1935. Found: 473.1929.

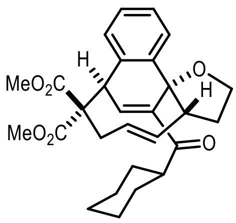

E-**2e**, yellow oil, 35.6 mg, 38% yield, *E*/*Z* = 8.6/1. ^{1}H NMR (400 MHz, $CDCl_3$) δ 7.52 (d, *J* = 8.0 Hz, 1H), 7.33 (t, *J* = 7.2 Hz, 1H), 7.17 (t, *J* = 7.6 Hz, 1H), 6.95 (d, *J* = 8.0 Hz, 1H), 6.48 (d, *J* = 5.2 Hz, 1H), 5.42 (dd, *J* = 16.0, 9.6 Hz, 1H), 5.12-5.05 (m, 1H), 4.52 (d, *J* = 5.2 Hz, 1H), 4.30-4.21 (m, 2H), 3.84 (s, 3H), 3.79 (s, 3H), 3.06 (dd, *J* = 12.8, 2.8 Hz, 1H), 2.74-2.58 (m, 2H), 2.55-2.48 (m, 1H), 2.24 (t, *J* = 12.4 Hz, 1H), 2.11-2.05 (m, 1H), 1.83-1.61 (m, 6H), 1.43-1.34 (m, 1H), 1.23-1.10 (m, 3H). ^{13}C NMR (100 MHz, $CDCl_3$) δ 209.6, 171.6, 171.3, 148.3, 143.4, 135.7, 134.6, 129.4, 128.0, 126.6, 126.5, 126.3, 124.6, 89.4, 72.3, 68.7, 55.7, 52.9, 52.6, 50.1, 44.3, 39.1, 29.7, 29.0, 27.9, 25.9, 25.9, 25.3. IR (thin film) ν_{max} (cm^{-1}) = 2925, 2850, 1733, 1687, 1446, 1433, 1231, 1205, 1183, 1069, 998, 945, 821, 788, 755, 732, 678, 645. HRMS (ESI) calcd for $C_{28}H_{32}O_6Na$ $[M+Na]^+$: 487.2091. Found: 487.2088.

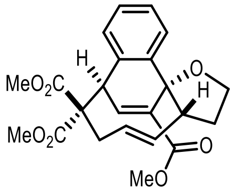

E-**2f**, yellow oil, 66.5 mg, 80% yield, *E*/*Z* = 7.1/1. ^{1}H NMR (400 MHz, $CDCl_3$) δ 7.55 (d, *J* = 8.4 Hz, 1H), 7.32 (t, *J* = 8.0 Hz, 1H), 7.15 (t, *J* = 7.6 Hz, 1H), 7.07 (d, *J* = 5.6 Hz, 1H), 6.91 (d, *J* = 8.0 Hz, 1H), 5.31 (dd, *J* = 16.0, 9.2 Hz, 1H), 5.16-5.08 (m, 1H), 4.53-4.47 (m, 2H), 4.34-4.28 (m, 1H), 3.84 (s, 3H), 3.80 (s, 3H), 3.75 (s, 3H), 3.09 (dd, *J* = 12.8, 2.8 Hz, 1H), 2.69-2.53 (m, 2H), 2.24 (t, *J* = 12.4 Hz, 1H), 2.17-2.13 (m, 1H). ^{13}C NMR (100 MHz, $CDCl_3$) δ 171.5, 171.0, 167.0, 143.4, 139.2, 136.8, 135.4, 134.3, 128.0, 127.0, 126.41, 126.39, 124.5, 89.0, 72.6, 68.6, 55.4, 52.9, 52.6, 51.8, 44.5, 39.2, 30.0. IR (thin film) ν_{max} (cm^{-1}) = 2952, 2857, 1729, 1708, 1435, 1340, 1272, 1231, 1205, 1132, 1066, 1040, 996, 968, 817, 788, 763, 699, 677, 644. HRMS (ESI) calcd for $C_{23}H_{24}O_7Na$ $[M+Na]^+$: 435.1414. Found: 435.1415.

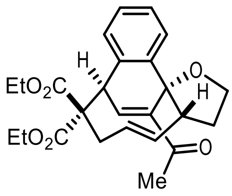

E-**2g**, yellow oil, 61.2 mg, 72% yield, *E*/*Z* = 6.3/1. ^{1}H NMR (400 MHz, $CDCl_3$) δ 7.54 (d, *J* = 8.0 Hz, 1H), 7.33 (t, *J* = 8.0 Hz, 1H), 7.15 (t, *J* = 8.0 Hz, 1H), 7.01 (d, *J* = 8.0 Hz, 1H), 6.82 (d, *J* = 5.2 Hz, 1H), 5.28 (dd, *J* = 16.0, 8.8 Hz, 1H), 5.13-5.06 (m, 1H), 4.54 (d, *J* = 5.2 Hz, 1H), 4.42-4.36 (m, 2H), 4.32-4.18 (m, 4H), 3.08 (dd, *J* = 12.8, 2.8 Hz, 1H), 2.59-2.49 (m, 2H), 2.34 (s, 3H), 2.21 (t, *J* = 12.4 Hz, 1H), 2.14-2.08 (m, 1H), 1.30 (q, *J* = 7.2 Hz, 6H). ^{13}C NMR (100 MHz, $CDCl_3$) δ 202.5, 171.1, 170.7, 147.9, 143.4, 135.3, 134.5, 133.8, 128.0, 126.9, 126.6, 126.3, 124.4, 89.3, 72.4, 68.7, 61.8, 55.6, 44.2, 39.2, 30.6, 30.0, 29.6, 14.0, 13.8. IR (thin film) ν_{max} (cm^{-1}) = 2920, 2852, 1726, 1685, 1446, 1363, 1245, 1225, 1131, 1095, 938, 905, 888, 870, 780, 757, 710, 675. HRMS (ESI) calcd for $C_{25}H_{28}O_6Na$ $[M+Na]^+$: 447.1778. Found: 447.1774.

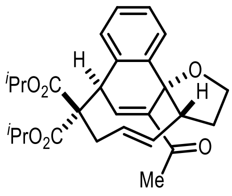

E-**2h**, yellow oil, 63.9 mg, 70% yield, *E*/*Z* = 8/1. ^{1}H NMR (400 MHz, $CDCl_3$) δ 7.54 (d, *J* = 8.0 Hz, 1H), 7.33 (t, *J* = 8.4 Hz, 1H), 7.17-7.10 (m, 2H), 6.84 (d, *J* = 5.2 Hz, 1H), 5.27 (dd, *J* = 16.0, 8.8 Hz, 1H), 5.19-5.05 (m, 3H), 4.52 (d, *J* = 5.6 Hz, 1H), 4.42-4.38 (m, 1H), 4.31-4.24 (m, 1H), 3.07 (dd, *J* = 12.8, 2.8 Hz, 1H), 2.63-2.48 (m, 2H), 2.33 (s, 3H), 2.19-2.09 (m, 2H), 1.40 (d, *J* = 6.4 Hz, 3H), 1.28 (d, *J* = 6.4 Hz, 6H), 1.24 (d, *J* = 6.4 Hz, 3H). ^{13}C NMR (100 MHz, $CDCl_3$) δ 202.5, 170.7, 170.2, 147.7, 143.4, 135.1, 134.6, 134.2, 127.9, 127.1, 126.8, 126.2, 124.4, 72.4, 70.0, 69.9, 69.3, 68.9, 55.6, 44.1, 39.4, 30.6, 30.0, 21.6, 21.5, 21.4. IR (thin film) ν_{max} (cm^{-1}) = 2980, 2936, 2861, 1724, 1682, 1447, 1372, 1232, 1208, 1181, 1047, 941, 911, 833, 789, 757, 707, 682. HRMS (ESI) calcd for $C_{27}H_{32}O_6Na$ $[M+Na]^+$: 475.2091. Found: 475.2083.

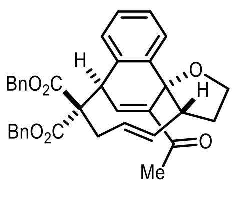

E-**2i**, yellow oil, 87.5 mg, 80% yield, *E*/*Z* = 8.8/1. ^{1}H NMR (400 MHz, $CDCl_3$) δ 7.52 (d, *J* = 8.0 Hz, 1H), 7.34-7.24 (m, 10H), 7.00 (t, *J* = 8.0 Hz, 1H), 6.92 (d, *J* = 7.2 Hz, 1H), 6.80 (d, *J* = 5.6 Hz, 1H), 5.31-5.25 (m, 2H), 5.15-5.06 (m, 4H), 4.57 (d, *J* = 5.6 Hz, 1H), 4.41-4.37 (m, 1H), 4.29-4.23 (m, 1H), 3.13 (dd, *J* = 12.8, 2.8 Hz, 1H), 2.60-2.46 (m, 2H), 2.30 (s, 3H), 2.25-2.16 (m, 1H), 2.15-2.02 (m, 1H). ^{13}C NMR (100 MHz, $CDCl_3$) δ 202.4, 170.7, 170.3, 148.1, 143.3, 135.4, 135.0, 134.5, 134.2, 133.4, 128.6, 128.54, 128.47, 128.4, 128.1, 128.0, 126.8, 126.6, 126.3, 124.4, 89.2, 72.4, 68.9, 67.5, 67.4, 55.6, 44.2, 39.3, 30.6, 29.9. IR (thin film) ν_{max} (cm^{-1}) = 2928, 2882, 1730, 1688, 1497, 1453, 1361, 1284, 1219, 1181, 1041, 997, 910, 844, 754, 733, 692, 620. HRMS (ESI) calcd for $C_{35}H_{32}O_6Na$ [M+Na]$^+$: 571.2091. Found: 571.2085.

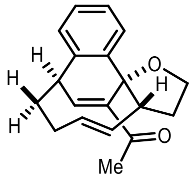

E-**2j**, colorless oil, 39.1 mg, 70% yield, *E*/*Z* = 2.6/1. ^{1}H NMR (400 MHz, $CDCl_3$) δ 7.56-7.49 (m, 1H), 7.35-7.11 (m, 3H), 6.74 (d, *J* = 6.4 Hz, 1H*), 6.51 (d, *J* = 5.2 Hz, 1H), 5.63-5.56 (m, 1H*), 5.29 (dd, *J* = 10.8, 6.4 Hz, 1H*), 5.09 (dd, *J* = 16.0, 9.6 Hz, 1H), 4.73-4.65 (m, 1H), 4.51-4.24 (m, 2H), 3.91-3.59 (m, 1H), 3.09-3.02 (m, 2H*), 2.68-2.54 (m, 2H), 2.44-2.23 (m, 2H), 2.34 (s, 3H), 2.25 (s, 3H*), 2.19-2.03 (m, 2H), 1.96-1.88 (m, 1H*), 1.79-1.72 (m, 1H). ^{13}C NMR (100 MHz, $CDCl_3$) δ 201.6, 198.9, 146.2, 144.9, 144.5, 141.1, 140.1, 139.2, 137.6, 135.1, 134.8, 132.5, 132.0, 130.8, 127.3, 127.0, 126.5, 126.1, 125.6, 123.69, 123.67, 89.9, 89.0, 72.2, 72.0, 57.0, 55.5, 40.6, 40.3, 39.0, 34.7, 34.3, 32.1, 30.11, 30.09, 28.5, 24.4. IR (thin film) ν_{max} (cm^{-1}) = 2923, 2844, 1676, 1478, 1451, 1373, 1254, 1219, 1183, 1123, 1045, 999, 937, 854, 753, 708, 669, 615. HRMS (ESI) calcd for $C_{19}H_{20}O_2Na$ [M+Na]$^+$: 303.1356. Found: 303.1356.

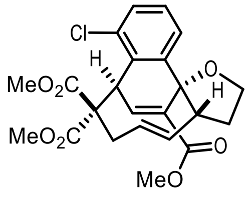

E-**2k**, white foam, 72.4 mg, 81% yield, *E*/*Z* = 5.3/1. ^{1}H NMR (400 MHz, $CDCl_3$) δ 7.50-7.48 (m, 1H), 7.32-7.24 (m, 2H), 6.69 (d, *J* = 5.6 Hz, 1H), 5.54-5.46 (m, 1H), 5.38-5.32 (m, 1H), 5.14 (d, *J* = 6.0 Hz, 1H), 4.48 (t, *J* = 8.4 Hz, 1H), 4.36-4.30 (m, 1H), 3.90

(s, 3H), 3.74 (s, 3H), 3.72 (s, 3H), 2.90-2.83 (m, 1H), 2.79-2.74 (m, 1H), 2.72-2.63 (m, 1H), 2.59-2.53 (m, 1H), 2.23-2.17 (m, 1H). ^{13}C NMR (100 MHz, $CDCl_3$) δ 171.8, 171.5, 166.8, 147.2, 139.6, 136.4, 136.2, 134.8, 133.0, 129.0, 127.7, 127.1, 123.9, 89.8, 72.9, 66.5, 55.4, 53.4, 53.1, 52.1, 42.7, 40.9, 30.2. IR (thin film) ν_{max} (cm^{-1}) = 1727, 1435, 1249, 1211, 1183, 1049, 1005, 912, 778, 732, 701, 646. HRMS (ESI) calcd for $C_{23}H_{23}O_7Na^{35}Cl$ $[M+Na]^+$: 469.1025. Found: 469.1027.

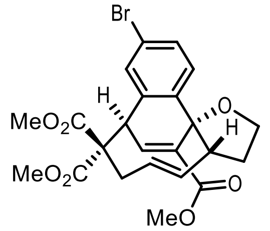

E-**2l**, white foam, 79.6 mg, 81% yield, *E*/*Z* = 5.6/1. ^{1}H NMR (400 MHz, $CDCl_3$) δ 7.422-7.417 (m, 2H), 7.08 (s, 1H), 7.03 (d, *J* = 5.2 Hz, 1H), 5.30-5.23 (m, 1H), 5.14-5.07 (m, 1H), 4.49-4.43 (m, 2H), 4.30-4.24 (m, 1H), 3.88 (s, 3H), 3.79 (s, 3H), 3.73 (s, 3H), 3.08 (d, *J* = 15.2 Hz, 1H), 2.56-2.46 (m, 2H), 2.25-2.10 (m, 2H). ^{13}C NMR (100 MHz, $CDCl_3$) δ 171.3, 170.8, 166.8, 142.7, 139.0, 136.6, 135.5, 131.1, 129.7, 127.2, 126.5, 120.6, 89.0, 72.8, 68.7, 55.5, 53.2, 53.0, 52.1, 44.4, 39.3, 30.1. IR (thin film) ν_{max} (cm^{-1}) = 1734, 1688, 1435, 1235, 1212, 1182, 1047, 1003, 911, 822, 733, 650. HRMS (ESI) calcd for $C_{23}H_{23}O_7Na^{79}Br$ $[M+H]^+$: 513.0519. Found: 513.0518.

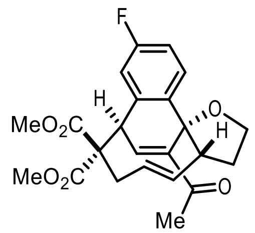

E-**2m**, white solid, 58.7 mg, 71% yield, m.p. = 153.5-155.1 °C, *E*/*Z* = 6.1/1. ^{1}H NMR (400 MHz, $CDCl_3$) δ 7.52-7.48 (m, 1H), 7.05-7.00 (m, 1H), 6.75 (d, *J* = 5.6 Hz, 1H), 6.64 (dd, *J* = 10.8, 2.8 Hz, 1H), 5.29-5.22 (m, 1H), 5.10-5.02 (m, 1H), 4.47 (d, *J* = 5.6 Hz, 1H), 4.37 (t, *J* = 7.6 Hz, 1H), 4.27-4.21 (m, 1H), 3.86 (s, 3H), 3.78 (s, 3H), 3.07 (dd, *J* = 12.8, 2.8 Hz, 1H), 2.53-2.41 (m, 2H), 2.32 (s, 3H), 2.26-2.17 (m, 1H), 2.12-2.06 (m, 1H). ^{13}C NMR (100 MHz, $CDCl_3$) δ 202.4, 171.3, 171.1, 161.4 (d, *J* = 243.5 Hz), 148.2, 139.4 (d, *J* = 2.9 Hz), 136.5 (d, *J* = 7.3 Hz), 135.6, 133.2, 126.7, 126.5 (d, *J* = 8.2 Hz), 115.4 (d, *J* = 21.1 Hz), 113.2 (d, *J* = 22.3 Hz), 89.3, 72.5, 68.7, 55.8, 53.1,

52.9, 44.5 (d, J = 1.8 Hz) , 39.2, 30.7, 30.0. ^{19}F NMR (376 MHz, $CDCl_3$) δ -116.15 ~ -116.21 (m, 1F), IR (thin film) ν_{max} (cm^{-1}) = 1735, 1691, 1491, 1437, 1233, 1047, 914, 823, 732, 652. HRMS (ESI) calcd for $C_{23}H_{23}O_6FNa$ $[M+Na]^+$: 437.1371. Found: 437.1377.

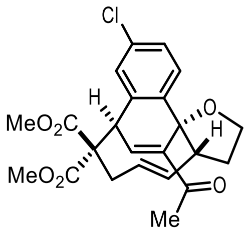

E-**2n**, white solid, 56.7 mg, 66% yield, m.p. = 139.2-140.4 °C, *E*/*Z* = 5.0/1. ^{1}H NMR (400 MHz, $CDCl_3$) δ 7.48-7.46 (m, 1H), 7.31-7.29 (m, 1H), 6.95-6.94 (m, 1H), 6.76-6.75 (m, 1H), 5.29-5.23 (m, 1H), 5.12-5.04 (m, 1H), 4.47 (d, J = 5.2 Hz, 1H), 4.38 (t, J = 8.0 Hz, 1H), 4.28-4.18 (m, 1H), 3.88 (s, 3H), 3.79 (s, 3H), 3.07 (dd, J = 13.2, 2.8 Hz, 1H), 2.56-2.44 (m, 2H), 2.33 (s, 3H), 2.23-2.17 (m, 1H), 2.14-2.08 (m, 1H). ^{13}C NMR (100 MHz, $CDCl_3$) δ 202.3, 171.3, 171.1, 148.0, 142.2, 136.3, 135.6, 133.3, 132.5, 128.3, 126.9, 126.8, 126.3, 89.2, 72.7, 68.8, 55.7, 53.2, 53.0, 44.4, 39.2, 30.8, 30.0. IR (thin film) ν_{max} (cm^{-1}) = 1733, 1683, 1438, 1237, 1215, 1047, 1005, 894, 851, 823, 732, 681, 648, 625. HRMS (ESI) calcd for $C_{23}H_{23}O_6Na^{35}Cl$ $[M+Na]^+$: 453.1075. Found: 453.1072.

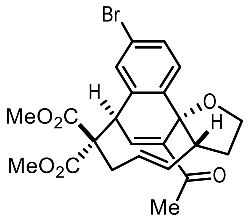

E-**2o**, white solid, 77.8 mg, 82% yield, m.p. = 144.6-144.9 °C, *E*/*Z* = 8.2/1. ^{1}H NMR (400 MHz, $CDCl_3$) δ 7.45-7.38 (m, 2H), 7.10-7.09 (m, 1H), 6.75-6.73 (m, 1H), 5.28-5.21 (m, 1H), 5.11-5.04 (m, 1H), 4.46 (d, J = 5.2 Hz, 1H), 4.37 (t, J = 8.0 Hz, 1H), 4.27-4.20 (m, 1H), 3.88 (s, 3H), 3.78 (s, 3H), 3.06 (dd, J = 12.8, 2.4 Hz, 1H), 2.54-2.44 (m, 2H), 2.32 (s, 3H), 2.22-2.16 (m, 1H), 2.12-2.07 (m, 1H). ^{13}C NMR (100 MHz, $CDCl_3$) δ 202.2, 171.3, 171.0, 147.9, 142.7, 136.6, 135.6, 133.3, 131.2, 129.7, 126.9, 126.5, 120.6, 89.2, 72.6, 68.8, 55.6, 53.2, 53.0, 44.3, 39.2, 30.7, 30.0. IR (thin film) ν_{max} (cm^-

^{1}) = 1734, 1688, 1435, 1235, 1212, 1182, 1047, 1003, 911, 822, 733, 650. HRMS (ESI) calcd for $C_{23}H_{23}O_6Na^{79}Br$ [M+Na]$^+$: 497.0570. Found: 497.0577.

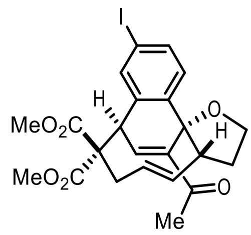

E-**2p**, white foam, 77.3 mg, 74% yield, *E*/*Z* = 7.7/1. ^{1}H NMR (400 MHz, $CDCl_3$) δ 7.62-7.60 (m, 1H), 7.31-7.30 (m, 1H), 7.24 (s, 1H), 6.70 (d, *J* = 5.2 Hz, 1H), 5.27-5.21 (m, 1H), 5.12-5.04 (m, 1H), 4.44 (d, *J* = 5.6 Hz, 1H), 4.36 (t, *J* = 8.0 Hz, 1H), 4.25-4.19 (m, 1H), 3.89 (s, 3H), 3.77 (s, 3H), 3.05 (dd, *J* = 12.8, 2.4 Hz, 1H), 2.54-2.44 (m, 2H), 2.32 (s, 3H), 2.21-2.14 (m, 1H), 2.12-2.06 (m, 1H). ^{13}C NMR (100 MHz, $CDCl_3$) δ 202.2, 171.2, 170.9, 147.9, 143.4, 136.9, 136.8, 135.8, 135.5, 133.0, 126.9, 126.6, 92.2, 89.1, 72.6, 68.7, 55.5, 53.1, 52.9, 44.1, 39.2, 30.6, 29.9. IR (thin film) ν_{max} (cm^{-1}) = 1734, 1688, 1437, 1234, 1214, 1182, 1047, 1004, 912, 822, 732, 648. HRMS (ESI) calcd for $C_{23}H_{23}O_6NaI$ [M+Na]$^+$: 545.0432. Found: 545.0437.

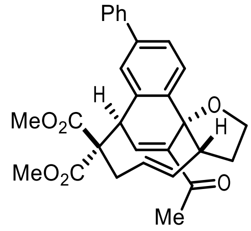

E-**2q**, yellow oil, 65.2 mg, 69% yield, *E*/*Z* = 6.4/1. ^{1}H NMR (400 MHz, $CDCl_3$) δ 7.62-7.60 (m, 1H), 7.57-7.52 (m, 3H), 7.44-7.40 (m, 2H), 7.35-7.31 (m, 1H), 7.17 (d, *J* = 2.0 Hz, 1H), 6.81 (d, *J* = 5.2 Hz, 1H), 5.34-5.28 (m, 1H), 5.20-5.14 (m, 1H), 4.60 (d, *J* = 4.0 Hz, 1H), 4.44-4.40 (m, 1H), 4.34-4.28 (m, 1H), 3.80 (s, 3H), 3.78 (s, 3H), 3.11 (dd, *J* = 12.8, 2.0 Hz, 1H), 2.60-2.51 (m, 2H), 2.36 (s, 3H), 2.27-2.21 (m, 1H), 2.15-2.12 (m, 1H). ^{13}C NMR (100 MHz, $CDCl_3$) δ 202.7, 171.6, 171.3, 148.3, 142.6, 141.0, 139.6, 135.6, 134.9, 133.5, 128.9, 127.5, 127.2, 127.2, 127.0, 125.8, 125.1, 89.4, 72.6, 68.9, 55.8, 53.1, 52.9, 44.7, 39.4, 30.8, 30.1. IR (thin film) ν_{max} (cm^{-1}) = 1737, 1689, 1437, 1236, 1217, 1181, 1041, 1002, 912, 825, 723, 641. HRMS (ESI) calcd for $C_{29}H_{28}O_6Na$ [M+Na]$^+$: 495.1884. Found: 495.1816.

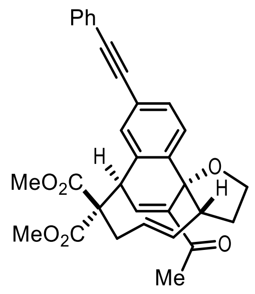

E-**2r**, yellow oil, 50.3 mg, 54% yield, *E*/*Z* = 6.4/1. ^{1}H NMR (400 MHz, $CDCl_3$) δ 7.53-7.48 (m, 4H), 7.36-7.33 (m, 3H), 7.14-7.13 (m, 1H), 6.79 (d, *J* = 5.2 Hz, 1H), 5.32-5.26 (m, 1H), 5.15-5.08 (m, 1H), 4.52 (d, *J* = 5.6 Hz, 1H), 4.41 (t, *J* = 8.0 Hz, 1H), 4.31-4.25 (m, 1H), 3.91 (s, 3H), 3.81 (s, 3H), 3.09 (dd, *J* = 12.8, 2.4 Hz, 1H), 2.58-2.48 (m, 2H), 2.35 (s, 3H), 2.25-2.19 (m, 1H), 2.15-2.11 (m, 1H). ^{13}C NMR (100 MHz, $CDCl_3$) δ 202.4, 171.5, 171.2, 148.0, 143.8, 135.6, 134.7, 133.5, 131.7, 131.1, 130.2, 128.5, 128.5, 127.1, 124.9, 123.3, 121.6, 89.5, 89.3, 89.1, 72.7, 68.9, 55.8, 53.1, 52.9, 44.4, 39.3, 30.8, 30.1. IR (thin film) ν_{max} (cm^{-1}) = 1739, 1689, 1437, 1238, 1217, 1171, 1041, 1002, 913, 822, 724, 651. HRMS (ESI) calcd for $C_{31}H_{28}O_6Na$ $[M+Na]^+$: 519.1886. Found: 519.1854.

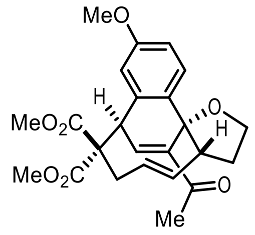

E-**2s**, white solid, 63.7 mg, 74% yield, m.p. = 162.9-164.5 °C, *E*/*Z* = 4.8/1. ^{1}H NMR (400 MHz, $CDCl_3$) δ 7.46-7.43 (m, 1H), 6.91-6.88 (m, 1H), 6.76-6.74 (m, 1H), 6.51-6.50 (m, 1H), 5.29-5.23 (m, 1H), 5.15-5.06 (m, 1H), 4.52 (d, *J* = 5.2 Hz, 1H), 4.37 (t, *J* = 7.6 Hz, 1H), 4.26-4.22 (m, 1H), 3.87 (s, 3H), 3.79 (s, 3H), 3.76 (s, 3H), 3.09-3.06 (m, 1H), 2.56-2.48 (m, 2H), 2.33 (s, 3H), 2.23-2.17 (m, 1H), 2.12-2.08 (m, 1H). ^{13}C NMR (100 MHz, $CDCl_3$) δ 202.8, 171.6, 171.3, 158.2, 148.6, 135.8, 135.8, 135.7, 133.5, 126.6, 125.8, 113.8, 112.5, 89.5, 72.4, 68.7, 55.8, 55.4, 53.1, 52.9, 44.5, 39.3, 30.8, 30.1. IR (thin film) ν_{max} (cm^{-1}) = 2953, 1733, 1685, 1611, 1497, 1436 1237, 1130, 1046, 1003, 913, 880, 817, 732, 685, 649. HRMS (ESI) calcd for $C_{24}H_{26}O_7Na$ $[M+Na]^+$: 449.1571. Found: 449.1565.

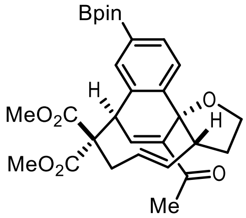

E-**2t**, white foam, 71.0 mg, 68% yield, *E*/*Z* = 8/1. ^{1}H NMR (400 MHz, $CDCl_3$) δ 7.73 (d, *J* = 7.6 Hz, 1H), 7.52 (d, *J* = 7.6 Hz, 1H), 7.43 (s, 1H), 6.77 (d, *J* = 5.2 Hz, 1H), 5.30-5.24 (m, 1H), 5.12-5.05 (m, 1H), 4.56 (d, *J* = 5.2 Hz, 1H), 4.41-4.37 (m, 1H), 4.31-4.25 (m, 1H), 3.95 (s, 3H), 3.79 (s, 3H), 3.09-3.06 (m, 1H), 2.56-2.46 (m, 2H), 2.33 (s, 3H), 2.23-2.09 (m, 2H), 1.32 (s, 12H). ^{13}C NMR (100 MHz, $CDCl_3$) δ 202.5, 171.3, 171.2, 147.9, 146.4, 135.4, 134.0, 133.61, 133.60, 133.5, 127.0, 123.7, 89.3, 83.7, 72.5, 68.6, 55.5, 52.9, 52.7, 44.3, 39.4, 30.6, 30.0, 25.0, 24.9. IR (thin film) ν_{max} (cm^{-1}) = 2962, 1734, 1685, 1612, 1499, 1439 1231, 1131, 1041, 1002, 912, 881, 814, 733, 686, 642. HRMS (ESI) calcd for $C_{29}H_{35}BO_8Na$ $[M+Na]^+$: 545.2425. Found: 545.2436.

2.3 Procedure for the cascade dearomative [5 + 4]/[2 + 2] cycloadditions of benzene derived VCPs (using 9a as an example)

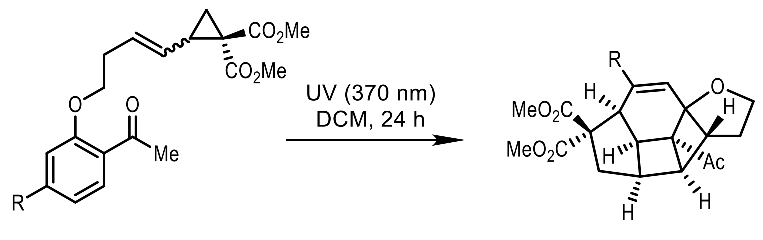

9a **10a**

To a flame-dried sealed tube were added benzene derivative **9a** (70.0 mg, 0.2 mmol) and anhydrous DCM (20 mL). The reaction mixture was degassed *via* freeze-pump-thaw for 3 cycles. After the reaction mixture was thoroughly degassed, the vial was sealed and positioned approximately 10 cm from UV LEDs (24 W, λ_{max} = 370 nm). Then the reaction mixture was stirred at room temperature for 24 h (monitored by TLC) under argon atmosphere. Afterwards, the reaction mixture was filtered and concentrated by rotary evaporation. Then, the residue was purified by silica gel column chromatography (petroleum ether/EtOAc = 3/1) to afford the desired product **10a**.

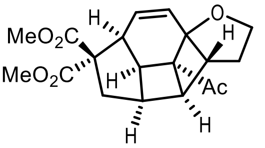

10a, yellow oil, 33.2 mg, 48% yield. ^{1}H NMR (400 MHz, $CDCl_3$) δ 6.08 (d, *J* = 10.4 Hz, 1H), 5.68 (dd, *J* = 10.8, 5.2 Hz, 1H), 4.00 (t, *J* = 8.8 Hz, 1H), 3.81-3.73 (m, 7H), 3.47-3.44 (m, 1H), 3.20-3.15 (m, 1H), 2.99-2.85 (m, 2H), 2.67-2.59 (m, 2H), 2.18-2.06 (m, 2H), 1.93 (s, 3H), 1.81-1.76 (m, 1H). ^{13}C NMR (100 MHz, $CDCl_3$) δ 207.5, 171.9, 169.5, 132.2, 126.5, 83.7, 69.2, 67.5, 55.1, 53.0, 52.6, 46.6, 41.0, 40.3, 33.7, 31.4, 30.7, 29.7, 25.5. IR (thin film) ν_{max} (cm^{-1}) = 2954, 2923, 2852, 1723, 1694, 1452, 1434, 1355, 1229, 1171, 1107, 1065, 982, 854, 749, 732, 681, 604. HRMS (ESI) calcd for $C_{19}H_{22}O_6Na$ $[M+Na]^+$: 369.1309. Found: 369.1302.

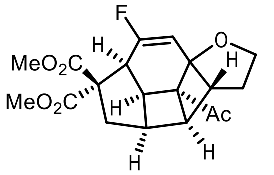

10b, yellow solid, 21.9 mg, 30% yield, m.p. = 127.3-127.9 °C. ^{1}H NMR (400 MHz, $CDCl_3$) δ 5.67 (d, J = 17.6 Hz, 1H), 3.99-3.95 (m, 1H), 3.76-3.66 (m, 7H), 3.27-3.21 (m, 1H), 3.01-2.99 (m, 1H), 2.96-2.84 (m, 2H), 2.78-2.72 (m, 1H), 2.64-2.63 (m, 1H), 2.26-2.22 (s, 1H), 2.15-2.05 (m, 1H), 1.94 (s, 3H), 1.82-1.76 (m, 1H). ^{13}C NMR (100 MHz, $CDCl_3$) δ 206.6, 171.4, 169.2, 159.0, 156.5, 109.8, 109.6, 85.7, 85.6, 69.4, 66.2, 66.1, 55.0, 53.3, 53.0, 46.6, 46.6, 44.0, 43.8, 41.7, 41.6, 33.20, 33.17, 33.1, 31.30, 31.27, 29.8, 25.7. ^{19}F NMR (376 MHz, $CDCl_3$) δ -99.55 ~ -99.58 (m, 1F). IR (thin film) ν_{max} (cm^{-1}) = 2989, 2928, 2851, 1720, 1693, 1451, 1434, 1355, 1228, 1170, 1108, 1066, 983, 854, 746, 733, 680, 602. HRMS (ESI) calcd for $C_{19}H_{21}FO_6Na$ $[M+Na]^+$: 387.1322. Found: 387.1312.

3. Transformations of Products

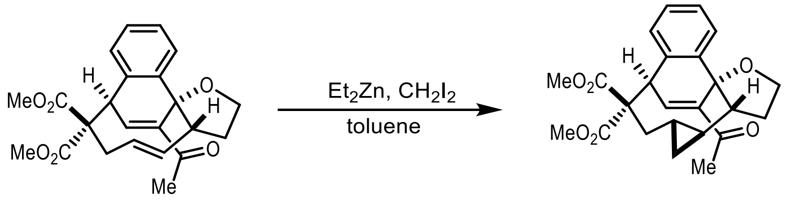

E-**2a** **3**

To a solution of *E*-**2a** (78.0 mg, 0.2 mmol) in toluene was added diethylzinc (0.8 mL, 1.6 mmol) and diiodomethane (0.26 mL, 3.2 mmol) under argon atmosphere. The reaction mixture was stirred at room temperature overnight. After completion (monitored by TLC), the reaction was quenched with water. The aqueous layer was extracted with EtOAc (10 mL × 3). The combined organic layers were washed with brine, dried over Na_2SO_4, filtered and concentrated by rotary evaporation and the residue was purified by silica gel column chromatography (petroleum ether/EtOAc = 6/1) to afford the desired product **3**.

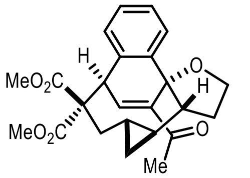

3, colorless oil, 56.0 mg, 70% yield. ^{1}H NMR (400 MHz, $CDCl_3$) δ 7.46 (d, *J* = 8.0 Hz, 1H), 7.28 (t, *J* = 7.6 Hz, 1H), 7.14 (t, *J* = 7.6 Hz, 1H), 7.02 (d, *J* = 8.0 Hz, 1H), 6.83 (d, *J* = 5.6 Hz, 1H), 4.79 (d, *J* = 5.6 Hz, 1H), 4.31 (t, *J* = 8.4 Hz, 1H), 4.23-4.17 (m, 1H), 3.82 (s, 3H), 3.77 (s, 3H), 2.71 (d, *J* = 14.0 Hz, 1H), 2.51-2.42 (m, 1H), 2.38 (s, 3H), 2.10-2.04 (m, 1H), 1.46-1.39 (m, 1H), 1.01-0.95 (m, 1H), 0.34-0.20 (m, 4H). ^{13}C NMR (100 MHz, $CDCl_3$) δ 203.8, 171.8, 171.5, 148.4, 146.8, 133.1, 132.9, 127.7, 126.7, 126.3, 124.6, 85.6, 71.3, 63.3, 56.8, 52.8, 51.7, 43.7, 37.4, 31.6, 30.9, 24.2, 11.3, 8.8. IR (thin film) ν_{max} (cm^{-1}) = 2946, 2845, 1729, 1695, 1435, 1356, 1235, 1209, 1149, 1097, 1071, 1042, 1001, 942, 894, 790, 762, 733, 679, 530, 478, 428. HRMS (ESI) calcd for $C_{24}H_{26}O_6Na$ $[M+Na]^+$: 433.1622. Found: 433.1628.

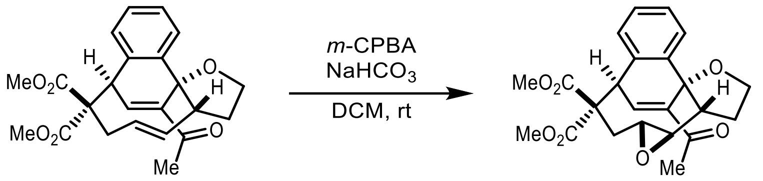

E-**2a** **4**

To a solution of *E*-**2a** (78.0 mg, 0.2 mmol) in DCM was added sodium bicarbonate (34.0 mg, 0.4 mmol) and *m*-chloroperbenzoic acid (52.0 mg, 0.3 mmol). The reaction mixture was stirred at room temperature overnight. After the completion of the reaction (monitored by TLC), the suspension was filtered and washed with dichloromethane. Then, the filtrate was concentrated by rotary evaporation and the residue was purified by silica gel column chromatography (petroleum ether /EtOAc = 3/1) to afford the desired product **4**.

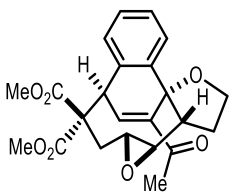

4, viscous solid, 61.1 mg, 96% yield. ^{1}H NMR (400 MHz, $CDCl_3$) δ 7.50 (d, *J* = 8.0 Hz, 1H), 7.32 (t, *J* = 7.6 Hz, 1H), 7.19 (t, *J* = 7.6 Hz, 1H), 7.02 (d, *J* = 8.0 Hz, 1H), 6.75 (d, *J* = 5.6 Hz, 1H), 4.82 (d, *J* = 5.6 Hz, 1H), 4.38 (t, *J* = 8.4 Hz, 1H), 4.31-4.25 (m, 1H), 3.84 (s, 3H), 3.80 (s, 3H), 2.93 (d, *J* = 13.2 Hz, 1H), 2.69-2.56 (m, 2H), 2.50-2.47 (m, 1H), 2.37 (s, 3H), 2.28-2.21 (m, 1H), 1.81-1.74 (m, 1H), 1.33-1.25 (m, 1H). ^{13}C NMR (100 MHz, $CDCl_3$) δ 203.5, 170.73, 170.71, 148.1, 145.5, 132.6, 132.0, 128.2, 127.0, 126.8, 124.8, 84.4, 72.3, 61.8, 61.4, 54.6, 53.2, 52.7, 51.9, 43.8, 35.2, 31.0, 29.8. IR (thin film) ν_{max} (cm^{-1}) = 2959, 2917, 1731, 1693, 1435, 1357, 1258, 1212, 1137, 1086, 1068, 1041, 970, 948, 902, 888, 840, 823, 791, 777, 740. HRMS (ESI) calcd for $C_{23}H_{24}O_7Na$ $[M+Na]^+$: 435.1414. Found: 435.1411.

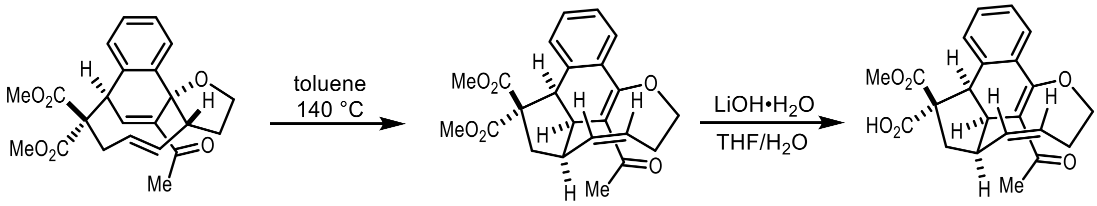

E-**2a** **5** **6**

To a solution of *E*-**2a** (79.0 mg, 0.2 mmol) in toluene was heated at 140 °C for 1.5 h under argon. After the completion of the reaction (monitored by TLC), the suspension was filtered and washed with dichloromethane. Then the filtrate was concentrated by rotary evaporation and the residue was purified by silica gel column chromatography (petroleum ether/EtOAc = 4/1) to afford the desired product **5**.

To a solution of **5** (79.2 mg, 0.2 mmol) in THF/H_2O (v/v = 1/1) was added LiOH·H_2O (34.0 mg, 0.8 mmol). The reaction mixture was stirred at room temperature overnight. After the completion of the reaction (monitored by TLC), the resulted solution was acidified with HCl (2 N) until pH < 2. The aqueous layer was extracted with EtOAc (10 mL × 3). The combined organic layers were washed with brine, dried over Na_2SO_4, filtered and concentrated by rotary evaporation and the residue was purified by silica gel column chromatography (petroleum ether/EtOAc = 1/5) to afford the desired product **6**.

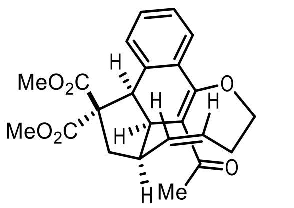

5, brown oil,70.7 mg, 89% yield. ^{1}H NMR (400 MHz, $CDCl_3$) δ 7.55-7.53 (m, 1H), 7.15-7.13 (m, 2H), 7.01-6.98 (m, 1H), 5.58 (dd, *J* = 11.6, 6.0 Hz, 1H), 5.10-5.03 (m, 1H), 4.75 (d, *J* = 8.8 Hz, 1H), 4.71-4.67 (m, 1H), 4.58-4.51 (m, 1H), 3.80 (s, 3H), 3.67 (s, 3H), 3.39-3.36 (m, 1H), 3.08-3.03 (m, 1H), 2.60-2.57 (m, 1H), 2.42 (s, 3H), 2.37-2.31 (m, 1H), 2.16-2.04 (m, 2H). ^{13}C NMR (100 MHz, $CDCl_3$) δ 199.8, 173.2, 172.5, 162.5, 136.9, 136.1, 134.9, 129.4, 129.3, 127.7, 126.9, 122.2, 76.8, 69.2, 60.4, 53.2, 52.4, 48.7, 47.7, 40.1, 29.5, 29.1, 14.3. IR (thin film) ν_{max} (cm^{-1}) = 1730, 1642, 1610, 1564, 1434, 1389, 1364, 1295, 1246, 1205, 1163, 1114, 1067, 1023, 911, 784, 729, 646. HRMS (ESI) calcd for $C_{23}H_{24}O_6Na$ [M+Na]$^+$: 419.1465. Found: 419.1465.

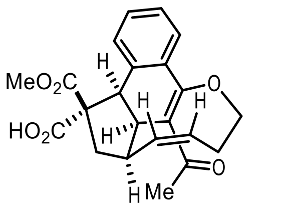

6, white solid, 46.6 mg, 61% yield, m.p. = 189.0-194.5 °C. ^{1}H NMR (400 MHz, $CDCl_3$) δ 7.52-7.50 (m, 1H), 7.11-7.09 (m, 2H), 7.01-6.99 (m, 1H), 6.79 (br, 1H), 5.54 (dd, *J* = 11.6, 6.0 Hz, 1H), 5.08-5.00 (m, 1H), 4.72-4.68 (m, 2H), 4.57-4.51 (m, 1H), 3.64 (s, 3H), 3.47-3.44 (m, 1H), 3.07-3.02 (m, 1H), 2.60-2.57 (m, 1H), 2.50-2.46 (m, 1H), 2.41 (s, 3H) , 2.13-1.99 (m, 2H). ^{13}C NMR (100 MHz, $CDCl_3$) δ 200.2, 177.6, 173.7, 163.2, 137.5, 135.9, 135.4, 129.3, 129.0, 127.7, 126.6, 123.5, 122.1, 69.8, 46.5, 40.4, 52.3, 49.0, 47.6, 29.7, 29.4, 28.9. IR (thin film) ν_{max} (cm^{-1}) = 1726, 1593, 1559, 1392, 1299, 1249, 1165, 910, 780, 723. HRMS (ESI) calcd for $C_{22}H_{22}O_6Na$ $[M+Na]^+$: 405.1309. Found: 405.1310.

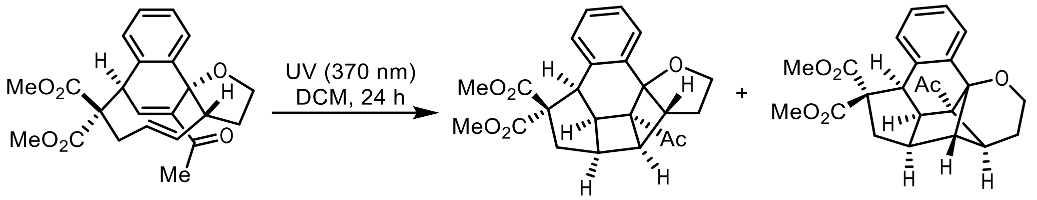

E-**2a** **7** **8**

To a flame-dried sealed tube were added *E*-**2a** (79.0 mg, 0.2 mmol) and anhydrous DCM (2.0 mL). The reaction mixture was degassed *via* freeze-pump-thaw for 3 cycles. After the reaction mixture was thoroughly degassed, the vial was sealed and positioned approximately 10 cm from UV LEDs (24 W, λ_{max} = 370 nm). Then the reaction mixture was stirred at room temperature for the indicated time (monitored by TLC) under argon atmosphere. Afterwards, the reaction mixture was concentrated by rotary evaporation. Then the residue was purified by silica gel column chromatography (petroleum ether/EtOAc = 4/1) to afford the desired products **7** and **8**.

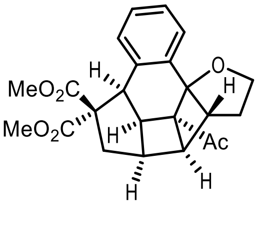

7, white solid, 33.3 mg, 42% yield, m.p. = 171.6-172.5 °C. ^{1}H NMR (400 MHz, $CDCl_3$) δ 7.61-7.60 (m, 1H), 7.28-7.26 (m, 1H), 7.22-7.20 (m, 1H), 7.12-7.11 (m, 1H), 4.42 (d, *J* = 6.4 Hz, 1H), 4.24 (t, *J* = 5.6 Hz, 1H), 3.96-3.92 (m, 1H), 3.78 (s, 3H), 3.56 (s, 3H), 3.35-3.32 (m, 1H), 3.12-3.07 (m, 2H), 2.80-2.77 (m, 1H), 2.73-2.72 (m, 1H), 2.26-2.15

(m, 2H), 1.86 (dd, J = 8.4, 3.6 Hz, 1H), 1.72 (s, 3H). ^{13}C NMR (100 MHz, $CDCl_3$) δ 207.2, 172.9, 169.5, 136.6, 133.0, 130.0, 127.7, 127.2, 125.2, 85.5, 71.2, 69.2, 55.4, 53.2, 52.3, 46.0, 45.5, 41.5, 35.2, 34.4, 32.2, 31.8, 25.5. IR (thin film) ν_{max} (cm^{-1}) = 2989, 1728, 1592, 1558, 1391, 1298, 1247, 1166, 911, 781, 720. HRMS (ESI) calcd for $C_{23}H_{24}O_6Na$ $[M+Na]^+$: 419.1465. Found: 419.1464.

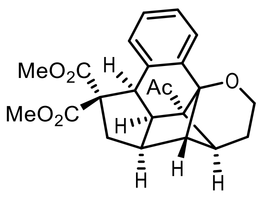

8, white solid, 32.5 mg, 41% yield, m.p. = 163.3.0-164.4 °C. ^{1}H NMR (400 MHz, $CDCl_3$) δ 7.65-7.64 (m, 1H), 7.27-7.25 (m, 1H), 7.17-7.14 (m, 1H), 6.98-6.96 (m, 1H), 4.20-4.15 (m, 1H), 4.06 (d, J = 4.0 Hz, 1H), 3.85-3.81 (m, 1H), 3.77 (s, 3H), 3.61 (s, 3H), 3.25 (dd, J = 6.4, 4.0 Hz, 1H), 2.95 (s, 1H), 2.57-2.52 (m, 2H), 2.19-2.14 (m, 2H), 2.00-1.95 (m, 1H), 1.77 (dd, J = 9.2, 5.6 Hz, 1H), 1.53 (s, 3H). ^{13}C NMR (100 MHz, $CDCl_3$) δ 209.7, 171.3, 169.1, 138.9, 133.9, 127.5, 127.4, 127.1, 126.4, 86.7, 70.9, 63.1, 61.3, 53.2, 52.8, 52.5, 51.9, 48.2, 46.6, 41.1, 30.5, 29.5, 24.2. IR (thin film) ν_{max} (cm^{-1}) = 2989, 1727, 1590, 1558, 1391, 1298, 1248, 1167, 912, 782, 721. HRMS (ESI) calcd for $C_{23}H_{24}O_6Na$ $[M+Na]^+$: 419.1465. Found: 419.1464.

4. Mechanistic Studies

4.1 Control experiment with triplet quencher

2,5-Dimethylhexa-2,4-diene is known as a triplet quencher.[3] The target dearomative [5 + 4] cycloaddition reaction of **1a** to *E*-**2a** was significantly inhibited in the presence of 2,5-dimethylhexa-2,4-diene (1 equiv) and the ^{1}H NMR yield of *E*-**2a** was reduced to 13%. This result suggested that a triplet diradical intermediate might be involved in the reaction.

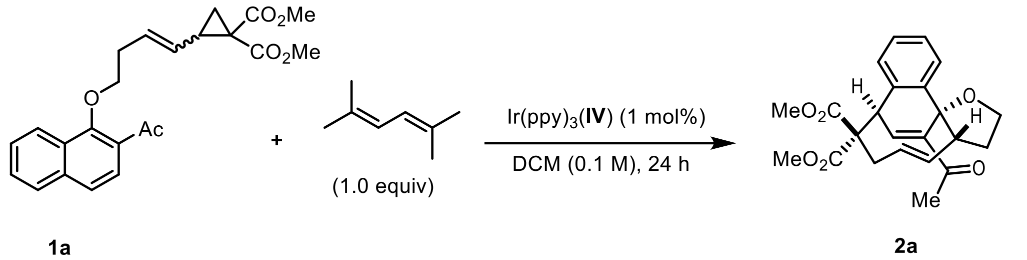

4.2 Transformation of *E*-2a under photosensitizer V

Compound *E*-**2a** (0.2 mmol) were subjected to following conditions: photosensitizer **V** (1 mol%) in CH_2Cl_2 (2.0 mL) in the presence of 24 W blue LEDs at room temperature under argon. At each time interval, 0.2 mL of the reaction mixture was taken out and quenched. The ratios of *E*-**2a**, *Z*-**2a**, and combined **7** and **8** in each crude mixture were determined by ^{1}H NMR analysis (Supplementary Table 1 and Supplementary Figure 1).

Supplementary Table 1. Transformation of *E*-**2a** under photosensitizer **V**.

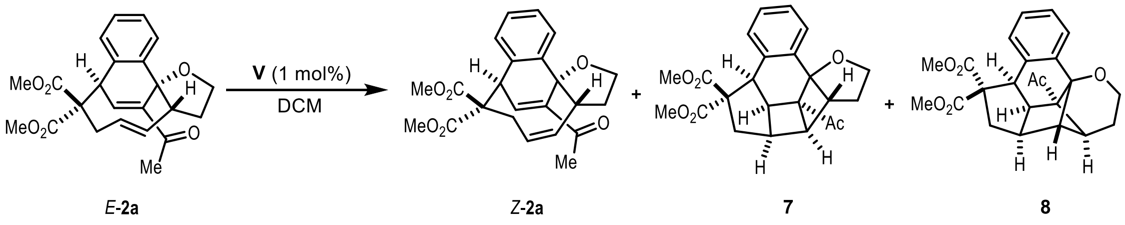

time (min)	0	2	4	6	12	22

E-**2a**	100	46	38	30	17	0
Combined **7** and **8**	0	28	28	28	30	41
Z-**2a**	0	26	34	42	53	59

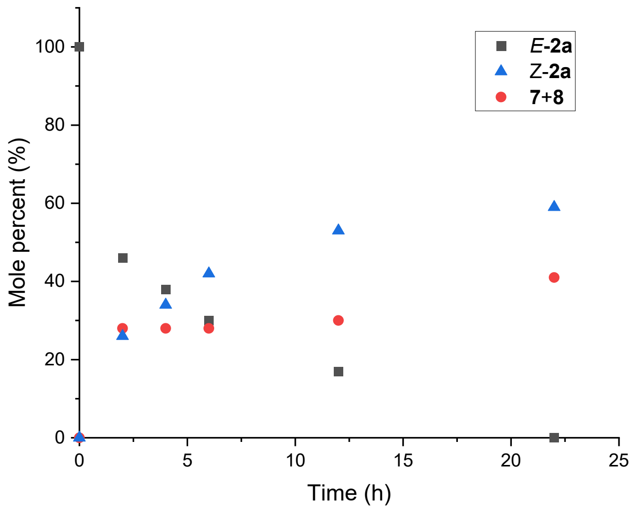

Supplementary Figure 1. Time course of mole percent of *E*-**2a**, *Z*-**2a**, **7** and **8**.

4.3 DFT calculations

Computational methods

All the calculations in this study were performed with Gaussian16.[4] DFT studies were carried out with the (U)B3LYP functional.[5–7] The Grimme dispersion correction scheme D3 with Becke–Johnson damping[8] was added to consider weak dispersion interaction.[9] The def2-SVP basis sets[10–11] of Weigend and Ahlrichs were employed for all atoms. Optimizations were conducted without any constraint in dichloromethane (SMD model,[12] ε = 8.93). Frequency analyses at the same computational level were carried out to confirm that each structure is a local minimum (no imaginary frequency) or a transition state (only one imaginary frequency). Single-point calculations were performed at the (U)M062X-D3/def2-TZVPP (SMD, DCM) level of theory in order to obtain more accurate estimation of the energies. The searching for minimal energy crossing points was conducted using a modified version of Harvey's code[13] (sob-MECP[14]) interfaced with Gaussian16.

Cartesian coordinates and energies for all the optimized structures

The coordinates of all the optimized structures are included in the Source Data file of this manuscript.

1a

```
Opt @ B3LYP-D3(BJ)/def2-SVP in dichloromethane (SMD model)
SCF Done: E(RB3LYP) = -1341.38963154 a.u.
Sum of electronic and thermal Free Energies = -1341.020292 a.u.
SP @ M062X-D3/def2-TZVPP in dichloromethane (SMD model)
SCF Done: E(RM062X) = -1342.23592769 a.u.
```

1a-T_1

```
Opt @ B3LYP-D3(BJ)/def2-SVP in dichloromethane (SMD model)
SCF Done: E(UB3LYP) = -1341.30216962 a.u.
Sum of electronic and thermal Free Energies = -1340.937981 a.u.
SP @ M062X-D3/def2-TZVPP in dichloromethane (SMD model)
```

SCF Done: E(UM062X) = -1342.13569702 a.u.

TS1-T$_1$

Opt @ B3LYP-D3(BJ)/def2-SVP in dichloromethane (SMD model)

SCF Done: E(UB3LYP) = -1341.29748593 a.u.

Sum of electronic and thermal Free Energies = -1340.931687 a.u.

SP @ M062X-D3/def2-TZVPP in dichloromethane (SMD model)

SCF Done: E(UM062X) = -1342.12794740 a.u.

INT1-T$_1$

Opt @ B3LYP-D3(BJ)/def2-SVP in dichloromethane (SMD model)

SCF Done: E(UB3LYP) = -1341.31478088 a.u.

Sum of electronic and thermal Free Energies = -1340.946167 a.u.

SP @ M062X-D3/def2-TZVPP in dichloromethane (SMD model)

SCF Done: E(UM062X) = -1342.15554547 a.u.

TS2-*Z*-T$_1$

Opt @ B3LYP-D3(BJ)/def2-SVP in dichloromethane (SMD model)

SCF Done: E(UB3LYP) = -1341.30558950 a.u.

Sum of electronic and thermal Free Energies = -1340.940485 a.u.

SP @ M062X-D3/def2-TZVPP in dichloromethane (SMD model)

SCF Done: E(UM062X) = -1342.14512613 a.u.

TS2-*E*-T$_1$

Opt @ B3LYP-D3(BJ)/def2-SVP in dichloromethane (SMD model)

SCF Done: E(UB3LYP) = -1341.31202575 a.u.

Sum of electronic and thermal Free Energies = -1340.944144 a.u.

SP @ M062X-D3/def2-TZVPP in dichloromethane (SMD model)

SCF Done: E(UM062X) = -1342.14936041 a.u.

INT2-*Z*-T$_1$

Opt @ B3LYP-D3(BJ)/def2-SVP in dichloromethane (SMD model)

SCF Done: E(UB3LYP) = -1341.32619376 a.u.

Sum of electronic and thermal Free Energies = -1340.959135 a.u.

SP @ M062X-D3/def2-TZVPP in dichloromethane (SMD model)

SCF Done: E(UM062X) = -1342.16185361 a.u.

INT2-*E*-T$_1$

Opt @ B3LYP-D3(BJ)/def2-SVP in dichloromethane (SMD model)

SCF Done: E(UB3LYP) = -1341.32954461 a.u.

Sum of electronic and thermal Free Energies = -1340.961183 a.u.

SP @ M062X-D3/def2-TZVPP in dichloromethane (SMD model)

SCF Done: E(UM062X) = -1342.16333040 a.u.

MECP1

Opt @ B3LYP-D3(BJ)/def2-SVP in dichloromethane (SMD model)

For open-shell singlet state

SCF Done: E(UB3LYP) = -1341.3294481500 a.u.

For triplet state

SCF Done: E(UB3LYP) = -1341.3294981100 a.u.

MECP2

Opt @ B3LYP-D3(BJ)/def2-SVP in dichloromethane (SMD model)

For open-shell singlet state

SCF Done: E(UB3LYP) = -1341.32568463 a.u.

For triplet state

SCF Done: E(UB3LYP) = -1341.32572861 a.u.

INT2-*Z*-OSS

Opt @ B3LYP-D3(BJ)/def2-SVP in dichloromethane (SMD model)

SCF Done: E(UB3LYP) = -1341.32600178 a.u.

Sum of electronic and thermal Free Energies = -1340.958052 a.u.

SP @ M062X-D3/def2-TZVPP in dichloromethane (SMD model)

SCF Done: E(UM062X) = -1342.16151159 a.u.

INT2-*E*-OSS

Opt @ B3LYP-D3(BJ)/def2-SVP in dichloromethane (SMD model)

SCF Done: E(UB3LYP) = -1341.32967525 a.u.

Sum of electronic and thermal Free Energies = -1340.960444 a.u.

SP @ M062X-D3/def2-TZVPP in dichloromethane (SMD model)

SCF Done: E(UM062X) = -1342.16407885 a.u.

TS3-*Z*-OSS

Opt @ B3LYP-D3(BJ)/def2-SVP in dichloromethane (SMD model)

SCF Done: E(UB3LYP) = -1341.32472239 a.u.

Sum of electronic and thermal Free Energies = -1340.954182 a.u.

SP @ M062X-D3/def2-TZVPP in dichloromethane (SMD model)

SCF Done: E(UM062X) = -1342.15891634 a.u.

TS3-*E*-OSS

Opt @ B3LYP-D3(BJ)/def2-SVP in dichloromethane (SMD model)

SCF Done: E(UB3LYP) = -1341.32963312 a.u.

Sum of electronic and thermal Free Energies = -1340.958276 a.u.

SP @ M062X-D3/def2-TZVPP in dichloromethane (SMD model)

SCF Done: E(UM062X) = -1342.16450125 a.u.

***Z*-2a**

Opt @ B3LYP-D3(BJ)/def2-SVP in dichloromethane (SMD model)

SCF Done: E(RB3LYP) = -1341.38836858 a.u.

Sum of electronic and thermal Free Energies = -1341.011828 a.u.

SP @ M062X-D3/def2-TZVPP in dichloromethane (SMD model)

SCF Done: E(RM062X) = -1342.23449745 a.u.

***E*-2a**

Opt @ B3LYP-D3(BJ)/def2-SVP in dichloromethane (SMD model)

SCF Done: E(RB3LYP) = -1341.37383766 a.u.

Sum of electronic and thermal Free Energies = -1340.998230 a.u.

SP @ M062X-D3/def2-TZVPP in dichloromethane (SMD model)

SCF Done: E(RM062X) = -1342.22170537 a.u.

5. Data of X-Ray Crystalloraphic Analyses

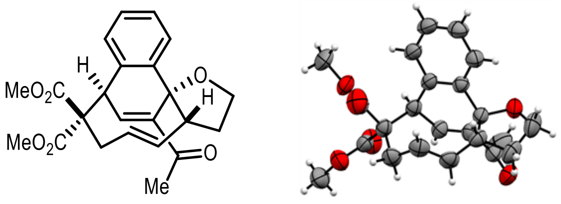

E-**2a**

Supplementary Figure 2. X-Ray crystal structure of *E*-**2a** (CCDC 2306009) (The crystal was obtained by slow evaporation of its solution of DCM and PE).

Supplementary Table 2. Crystal data and structure refinement for mo_d8v21450_0m.

Identification code	mo_d8v21450_0m	
Empirical formula	C23 H24 O6	
Formula weight	396.42	
Temperature	293(2) K	
Wavelength	0.71073 Å	
Crystal system	Monoclinic	
Space group	C 2/c	
Unit cell dimensions	a = 21.3487(8) Å	a = 90°
	b = 12.0164(4) Å	b = 109.1310(10)°
	c = 16.6320(6) Å	g = 90°
Volume	4031.0(3) $Å^3$	
Z	8	
Density (calculated)	1.306 Mg/m^3	
Absorption coefficient	0.094 mm^{-1}	
F(000)	1680	
Crystal size	0.160 x 0.140 x 0.100 mm^3	
Theta range for data collection	2.714 to 25.999°	
Index ranges	-23<=h<=26, -14<=k<=14, -20<=l<=20	
Reflections collected	29785	
Independent reflections	3944 [R(int) = 0.0490]	
Completeness to theta = 25.242°	99.4 %	
Absorption correction	Semi-empirical from equivalents	
Max. and min. transmission	0.7456 and 0.5501	

Refinement method	Full-matrix least-squares on F^2
Data / restraints / parameters	3944 / 0 / 266
Goodness-of-fit on F^2	1.079
Final R indices [I>2sigma(I)]	R1 = 0.0706, wR2 = 0.2074
R indices (all data)	R1 = 0.0871, wR2 = 0.2258
Extinction coefficient	0.0045(15)
Largest diff. peak and hole	1.342 and -0.280 e.Å^{-3}

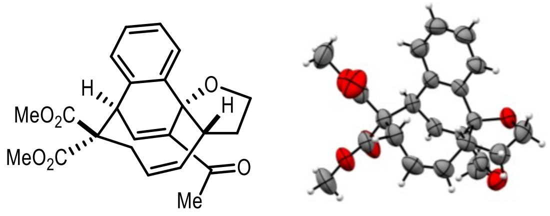

Z-**2a**

Supplementary Figure 3. X-Ray crystal structure of *Z*-**2a** (CCDC 2306010) (The crystal was obtained by slow evaporation of its solution of DCM and PE).

Supplementary Table 3. Crystal data and structure refinement for d8v21441.

Identification code	d8v21441	
Empirical formula	C23 H24 O6	
Formula weight	396.42	
Temperature	293(2) K	
Wavelength	0.71073 Å	
Crystal system	Monoclinic	
Space group	P 21/c	
Unit cell dimensions	a = 10.4581(10) Å	a = 90°
	b = 21.448(2) Å	b = 97.906(3)°
	c = 8.9823(10) Å	g = 90°
Volume	1995.6(4) $Å^3$	
Z	4	
Density (calculated)	1.319 Mg/m^3	
Absorption coefficient	0.095 mm^{-1}	
F(000)	840	
Crystal size	0.110 x 0.100 x 0.050 mm^3	
Theta range for data collection	2.734 to 25.500°	
Index ranges	-12<=h<=12, -25<=k<=25, -10<=l<=8	
Reflections collected	15680	
Independent reflections	3707 [R(int) = 0.0628]	
Completeness to theta = 25.242°	99.8 %	
Absorption correction	Semi-empirical from equivalents	
Max. and min. transmission	0.7456 and 0.5202	
Refinement method	Full-matrix least-squares on F^2	
Data / restraints / parameters	3707 / 0 / 266	
Goodness-of-fit on F^2	1.058	
Final R indices [I>2sigma(I)]	R1 = 0.0579, wR2 = 0.1305	

R indices (all data)	R1 = 0.1062, wR2 = 0.1545
Extinction coefficient	0.013(3)
Largest diff. peak and hole	0.179 and -0.118 e.Å^{-3}

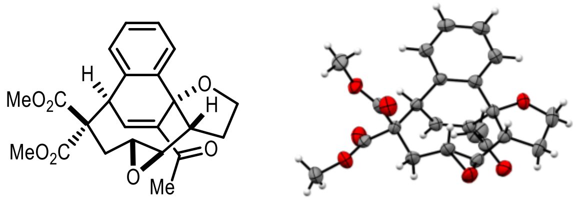

4

Supplementary Figure 4. X-Ray crystal structure of **4** (CCDC 2306011) (The crystal was obtained by slow evaporation of the solution of DCM and PE).

Supplementary Table 4. Crystal data and structure refinement for mo_d8v21712_0m.

Identification code	mo_d8v21712_0m	
Empirical formula	C23 H24 O7	
Formula weight	412.42	
Temperature	213(2) K	
Wavelength	0.71073 Å	
Crystal system	Trigonal	
Space group	P 32 2 1	
Unit cell dimensions	a = 12.1993(4) Å	a = 90°
	b = 12.1993(4) Å	b = 90°
	c = 23.4620(8) Å	g = 120°
Volume	3023.9(2) $Å^3$	
Z	6	
Density (calculated)	1.359 Mg/m^3	
Absorption coefficient	0.101 mm^{-1}	
F(000)	1308	
Crystal size	0.170 x 0.150 x 0.120 mm^3	
Theta range for data collection	2.114 to 25.997°.	
Index ranges	-15<=h<=13, -13<=k<=15, -28<=l<=28	
Reflections collected	26834	
Independent reflections	3950 [R(int) = 0.0333]	
Completeness to theta = 25.242°	99.5 %	
Absorption correction	Semi-empirical from equivalents	
Max. and min. transmission	0.7456 and 0.6556	
Refinement method	Full-matrix least-squares on F^2	
Data / restraints / parameters	3950 / 0 / 275	
Goodness-of-fit on F^2	1.063	

Final R indices [I>2sigma(I)]	R1 = 0.0321, wR2 = 0.0799
R indices (all data)	R1 = 0.0358, wR2 = 0.0831
Absolute structure parameter	0.2(3)
Extinction coefficient	0.014(2)
Largest diff. peak and hole	0.171 and -0.160 e.Å^{-3}

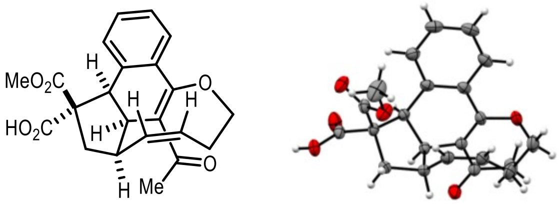

6

Supplementary Figure 5. X-Ray crystal structure of **6** (CCDC 2306012) (The crystal was obtained by slow evaporation of the solution of DCM and PE).

Supplementary Table 5. Crystal data and structure refinement for mj22448_0m.

Identification code	mj22448_0m	
Empirical formula	C23 H24 Cl2 O6	
Formula weight	467.32	
Temperature	222.00 K	
Wavelength	1.34139 Å	
Crystal system	Monoclinic	
Space group	C 1 2/c 1	
Unit cell dimensions	a = 16.0500(2) Å	a = 90°
	b = 12.2364(2) Å	b = 94.7710(10)°
	c = 22.2958(3) Å	g = 90°
Volume	4363.59(11) $Å^3$	
Z	8	
Density (calculated)	1.423 Mg/m^3	
Absorption coefficient	1.982 mm^{-1}	
F(000)	1952	
Crystal size	0.07 x 0.07 x 0.05 mm^3	
Theta range for data collection	3.957 to 54.955°.	
Index ranges	-19<=h<=19, -14<=k<=14, -27<=l<=27	
Reflections collected	34975	
Independent reflections	4159 [R(int) = 0.0435]	
Completeness to theta = 53.594°	99.8 %	
Absorption correction	Semi-empirical from equivalents	
Max. and min. transmission	0.7508 and 0.5805	
Refinement method	Full-matrix least-squares on F^2	
Data / restraints / parameters	4159 / 0 / 283	
Goodness-of-fit on F^2	1.062	
Final R indices [I>2sigma(I)]	R1 = 0.0735, wR2 = 0.2007	

R indices (all data)	R1 = 0.0849, wR2 = 0.2114
Extinction coefficient	n/a
Largest diff. peak and hole	1.688 and -1.268 e.Å^{-3}

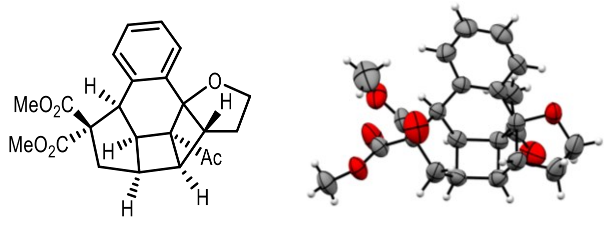

7

Supplementary Figure 6. X-Ray crystal structure of **7** (CCDC 2306013) (The crystal was obtained by slow evaporation of its solution of DCM and PE).

Supplementary Table 6. Crystal data and structure refinement for mo_d8v20517_0m.

Identification code	mo_d8v20517_0m	
Empirical formula	C23 H24 O6	
Formula weight	396.42	
Temperature	293(2) K	
Wavelength	0.71073 Å	
Crystal system	Monoclinic	
Space group	P 21/n	
Unit cell dimensions	a = 7.6087(2) Å	a = 90°
	b = 24.2039(9) Å	b = 96.5780(10)°
	c = 10.9288(4) Å	g = 90°
Volume	1999.40(12) $Å^3$	
Z	4	
Density (calculated)	1.317 Mg/m^3	
Absorption coefficient	0.095 mm^{-1}	
F(000)	840	
Crystal size	0.150 x 0.120 x 0.070 mm^3	
Theta range for data collection	2.520 to 26.000°.	
Index ranges	-9<=h<=9, -26<=k<=29, -13<=l<=13	
Reflections collected	20044	
Independent reflections	3932 [R(int) = 0.0409]	
Completeness to theta = 25.242°	99.8 %	
Absorption correction	Semi-empirical from equivalents	
Max. and min. transmission	0.7456 and 0.6700	
Refinement method	Full-matrix least-squares on F^2	
Data / restraints / parameters	3932 / 0 / 266	
Goodness-of-fit on F^2	1.045	

Final R indices [I>2sigma(I)]	R1 = 0.0489, wR2 = 0.1114
R indices (all data)	R1 = 0.0735, wR2 = 0.1282
Extinction coefficient	0.028(3)
Largest diff. peak and hole	0.194 and -0.143 e.Å^{-3}

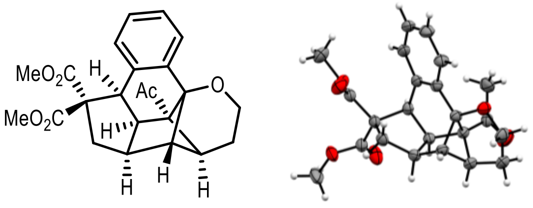

8

Supplementary Figure 7. X-Ray crystal structure of **8** (CCDC 2306014) (The crystal was obtained by slow evaporation of its solution of DCM and PE).

Supplementary Table 7. Crystal data and structure refinement for mj22259_0m.

Identification code	mj22259_0m	
Empirical formula	C23 H24 O6	
Formula weight	396.42	
Temperature	213 K	
Wavelength	1.34139 Å	
Crystal system	Triclinic	
Space group	P-1	
Unit cell dimensions	a = 9.2876(2) Å	a = 96.6560(10)°
	b = 9.8998(2) Å	b = 107.6530(10)°
	c = 11.7254(2) Å	g = 105.5850(10)°.
Volume	966.41(3) $Å^3$	
Z	2	
Density (calculated)	1.362 Mg/m^3	
Absorption coefficient	0.515 mm^{-1}	
F(000)	420	
Crystal size	0.07 x 0.07 x 0.05 mm^3	
Theta range for data collection	3.524 to 55.046°.	
Index ranges	-11<=h<=11, -12<=k<=11, -14<=l<=14	
Reflections collected	12960	
Independent reflections	3600 [R(int) = 0.0380]	
Completeness to theta = 53.594°	97.4 %	
Absorption correction	Semi-empirical from equivalents	
Max. and min. transmission	0.7508 and 0.6660	
Refinement method	Full-matrix least-squares on F^2	
Data / restraints / parameters	3600 / 0 / 265	
Goodness-of-fit on F^2	1.052	
Final R indices [I>2sigma(I)]	R1 = 0.0414, wR2 = 0.1083	
R indices (all data)	R1 = 0.0447, wR2 = 0.1110	

Extinction coefficient	n/a
Largest diff. peak and hole	0.236 and -0.189 e.$Å^{-3}$

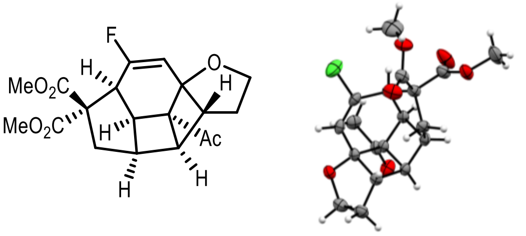

10b

Supplementary Figure 8. X-Ray crystal structure of **10b** (CCDC 2306015) (The crystal was obtained by slow evaporation of the solution of DCM and PE).

Supplementary Table 8. Crystal data and structure refinement for mj22522_0m.

Identification code	mj22522_0m	
Empirical formula	C19 H21 Cl O6	
Formula weight	380.81	
Temperature	213.00 K	
Wavelength	1.34139 Å	
Crystal system	Triclinic	
Space group	P-1	
Unit cell dimensions	a = 7.36980(10) Å	a = 109.4910(10)°
	b = 11.0888(2) Å	b = 107.1150(10)°
	c = 12.4624(2) Å	g = 95.8060(10)°
Volume	894.83(3) $Å^3$	
Z	2	
Density (calculated)	1.413 Mg/m^3	
Absorption coefficient	1.435 mm^{-1}	
F(000)	400	
Crystal size	0.07 x 0.07 x 0.05 mm^3	
Theta range for data collection	3.772 to 54.944°	
Index ranges	-8<=h<=8, -13<=k<=13, -15<=l<=15	
Reflections collected	15581	
Independent reflections	3354 [R(int) = 0.0379]	
Completeness to theta = 53.594°	98.7 %	
Absorption correction	Semi-empirical from equivalents	
Max. and min. transmission	0.7508 and 0.5846	
Refinement method	Full-matrix least-squares on F^2	
Data / restraints / parameters	3354 / 0 / 238	
Goodness-of-fit on F^2	1.103	
Final R indices [I>2sigma(I)]	R1 = 0.0505, wR2 = 0.1491	

R indices (all data)	R1 = 0.0551, wR2 = 0.1527
Extinction coefficient	n/a
Largest diff. peak and hole	0.280 and -0.684 e.$Å^{-3}$

6. Copies of NMR Spectra

^{1}H NMR Spectrum (400 MHz, $CDCl_3$) of **1a**

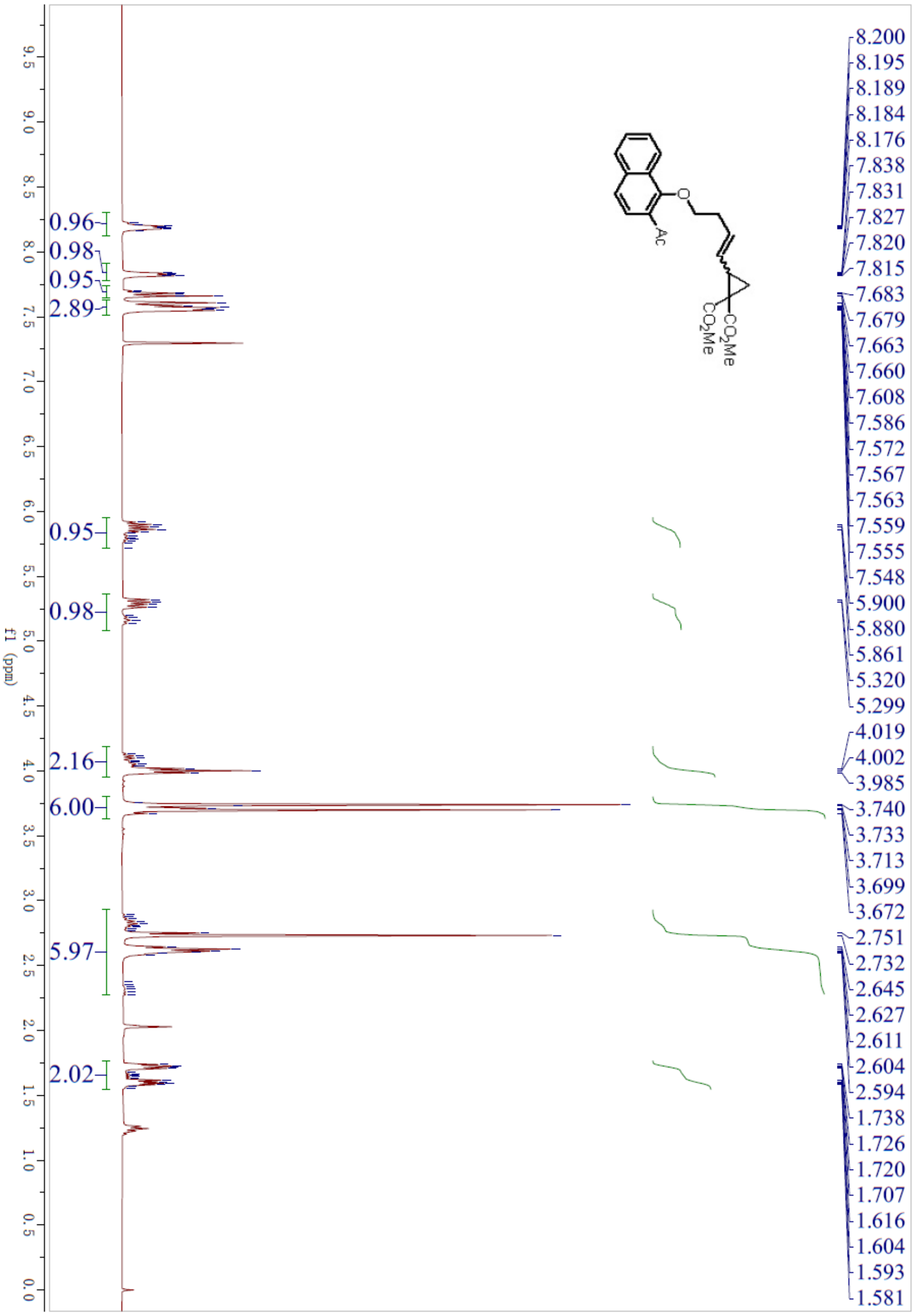

^{13}C NMR Spectrum (100 MHz, $CDCl_3$) of **1a**

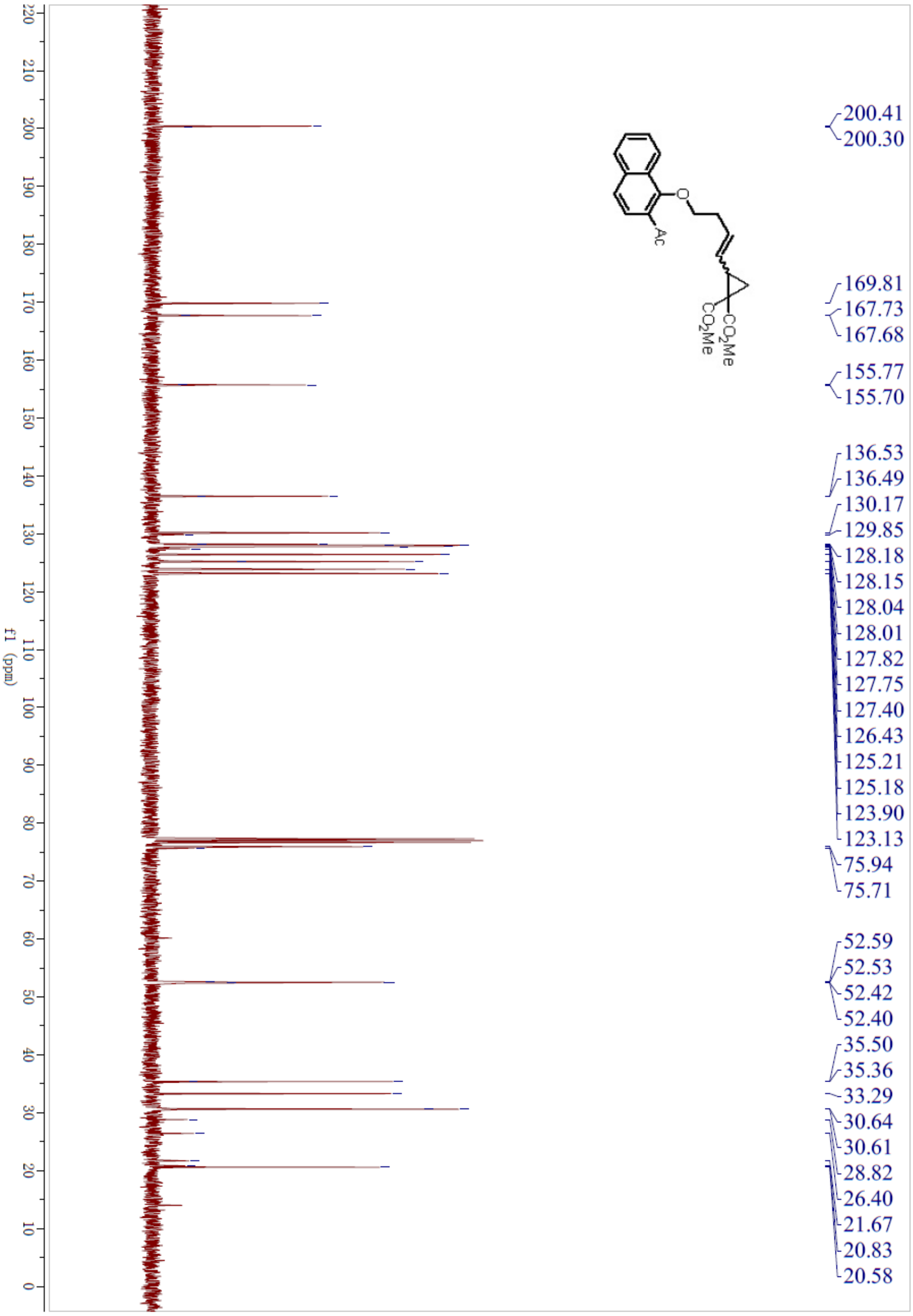

^{1}H NMR Spectrum (400 MHz, $CDCl_3$) of **1b**

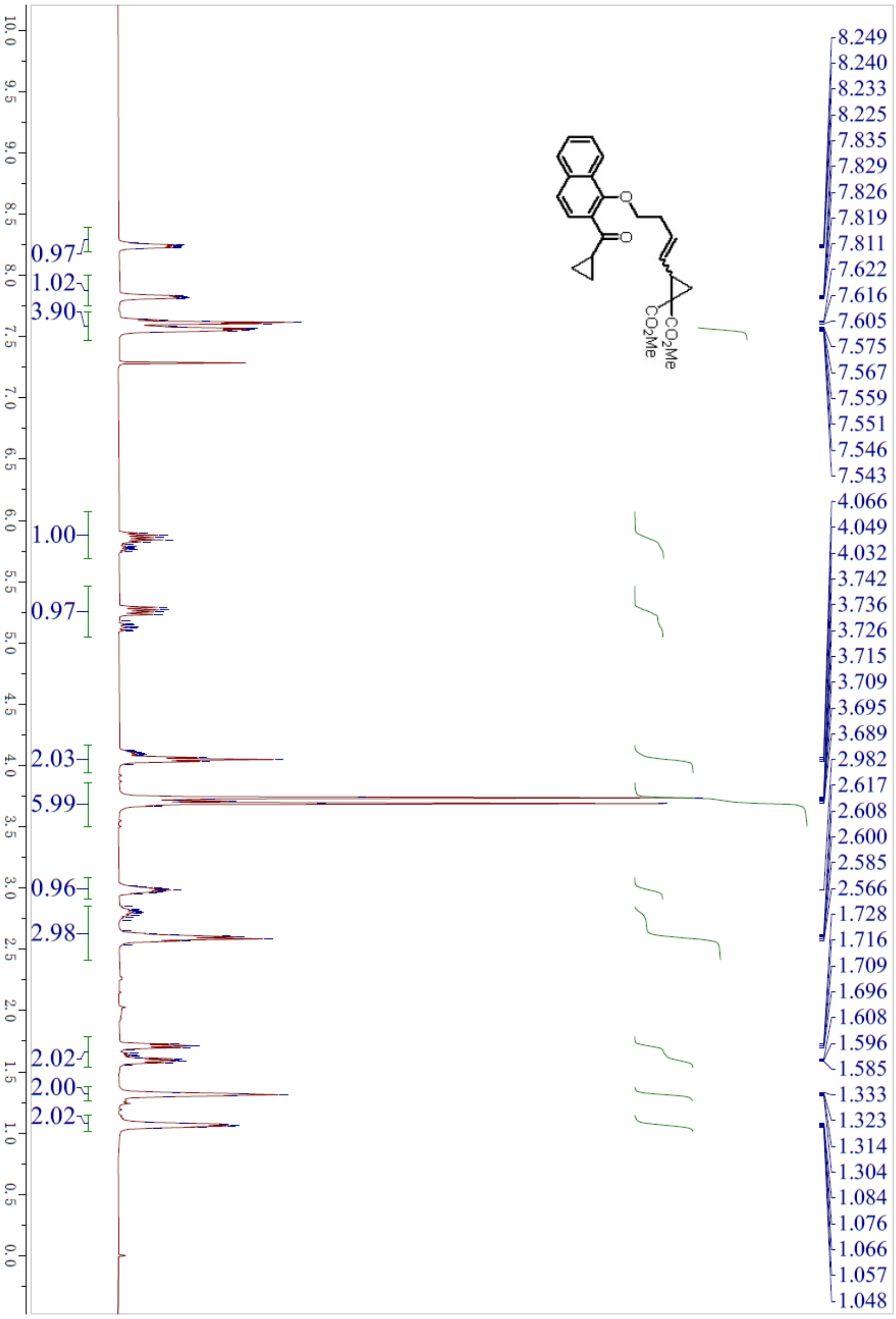

^{13}C NMR Spectrum (100 MHz, CDCl3) of **1b**

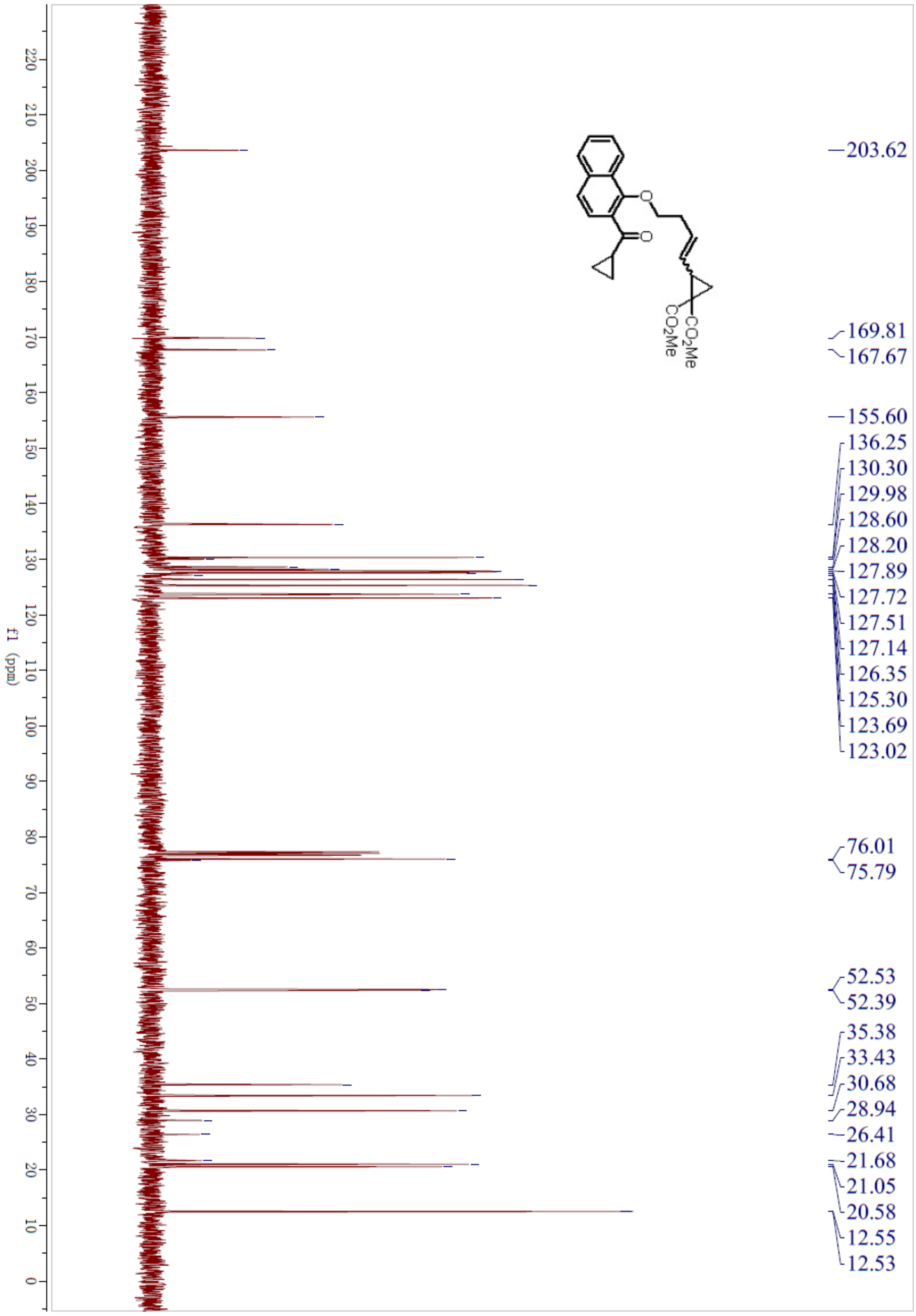

^{1}H NMR Spectrum (400 MHz, $CDCl_3$) of **1c**

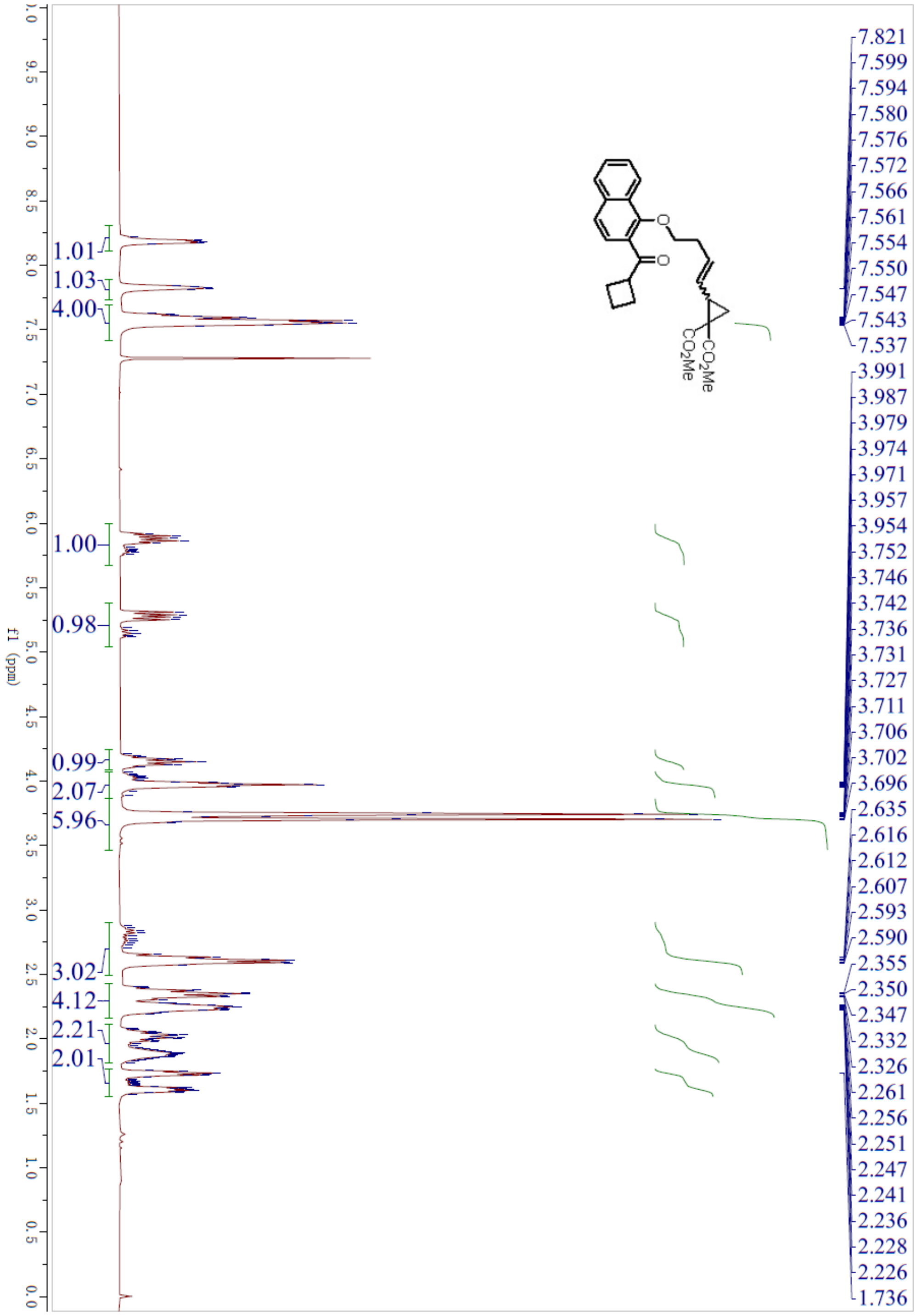

^{13}C NMR Spectrum (100 MHz, $CDCl_3$) of **1c**

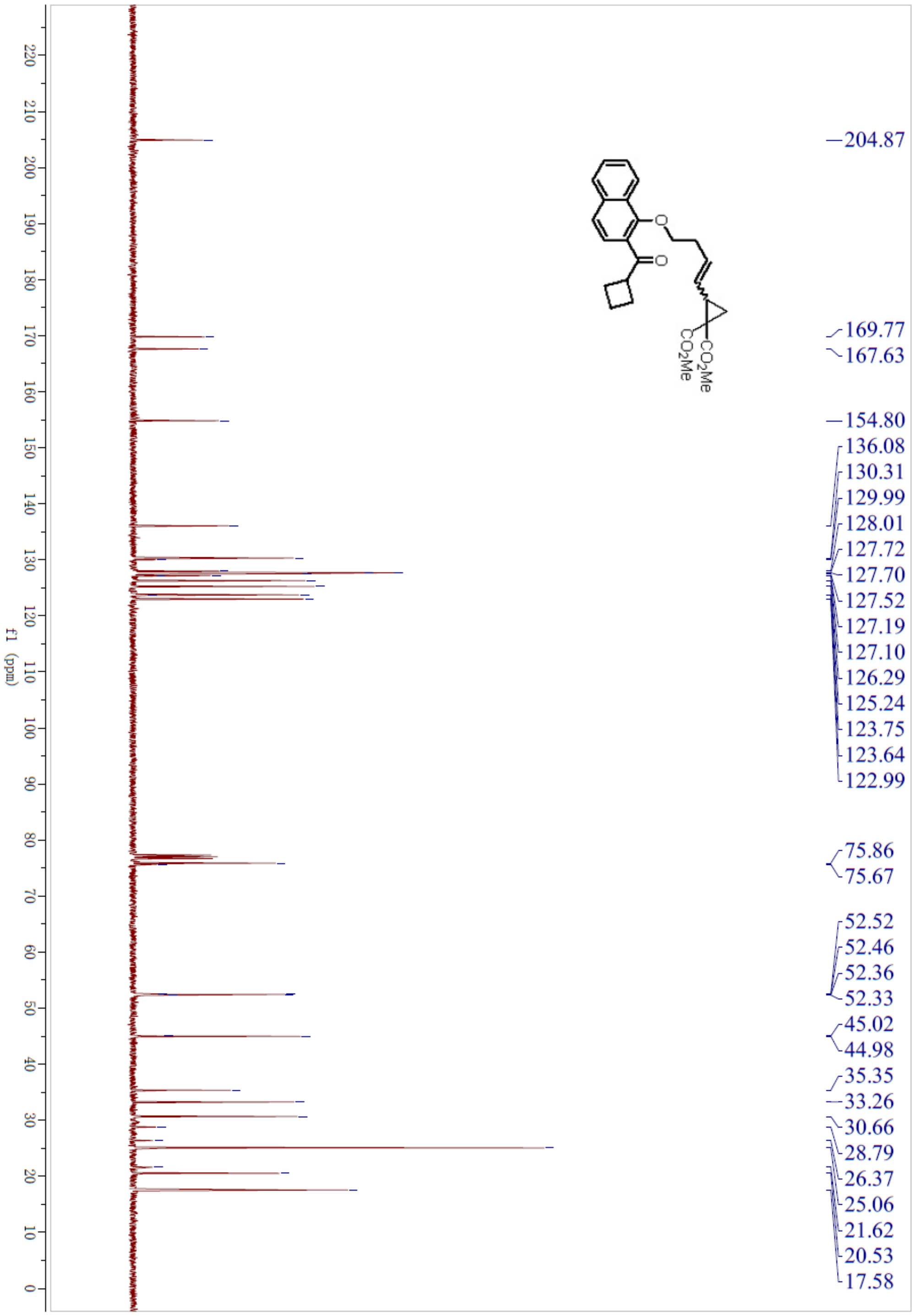

^{1}H NMR Spectrum (400 MHz, CDCl3) of **1d**

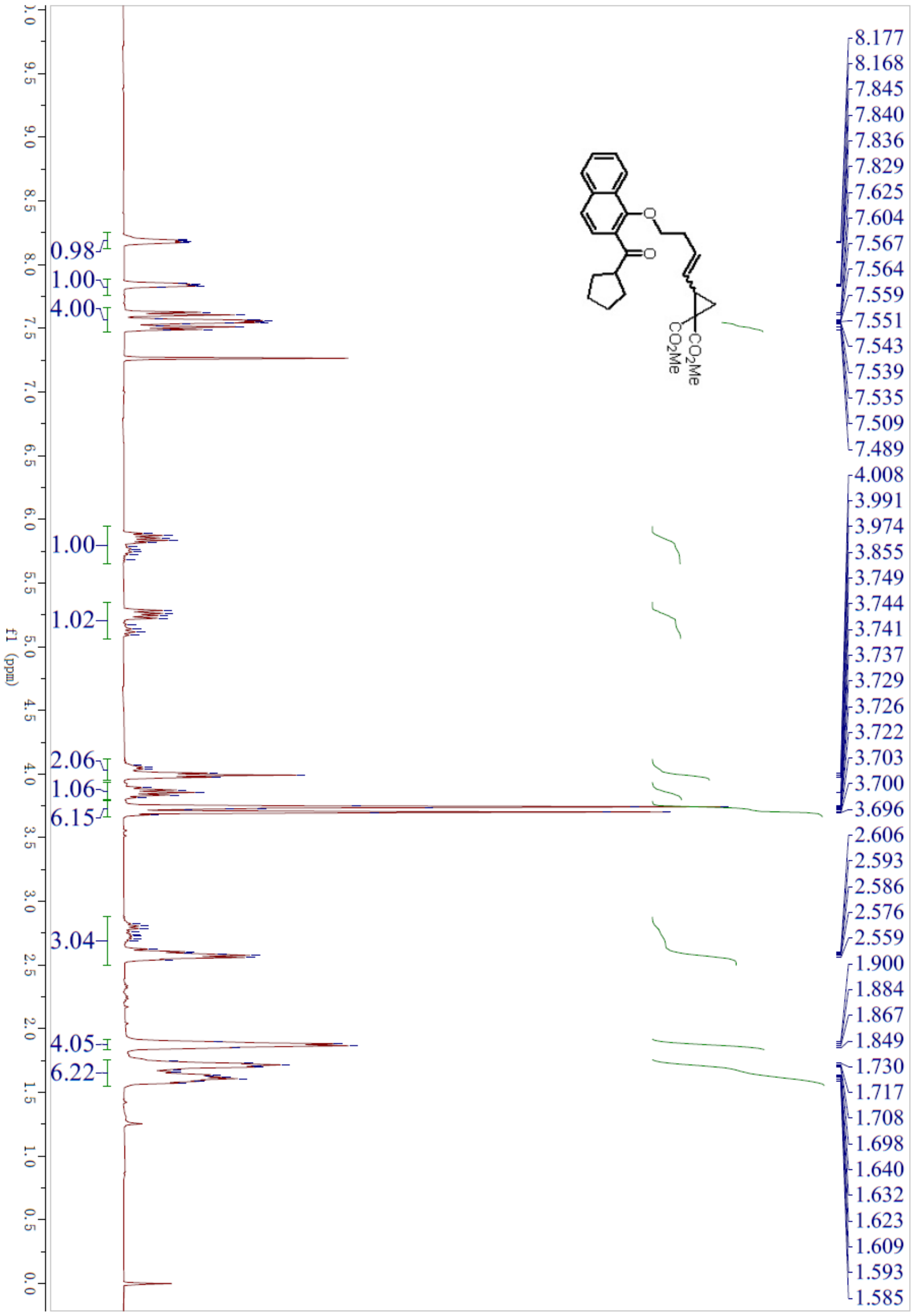

^{13}C NMR Spectrum (100 MHz, $CDCl_3$) of **1d**

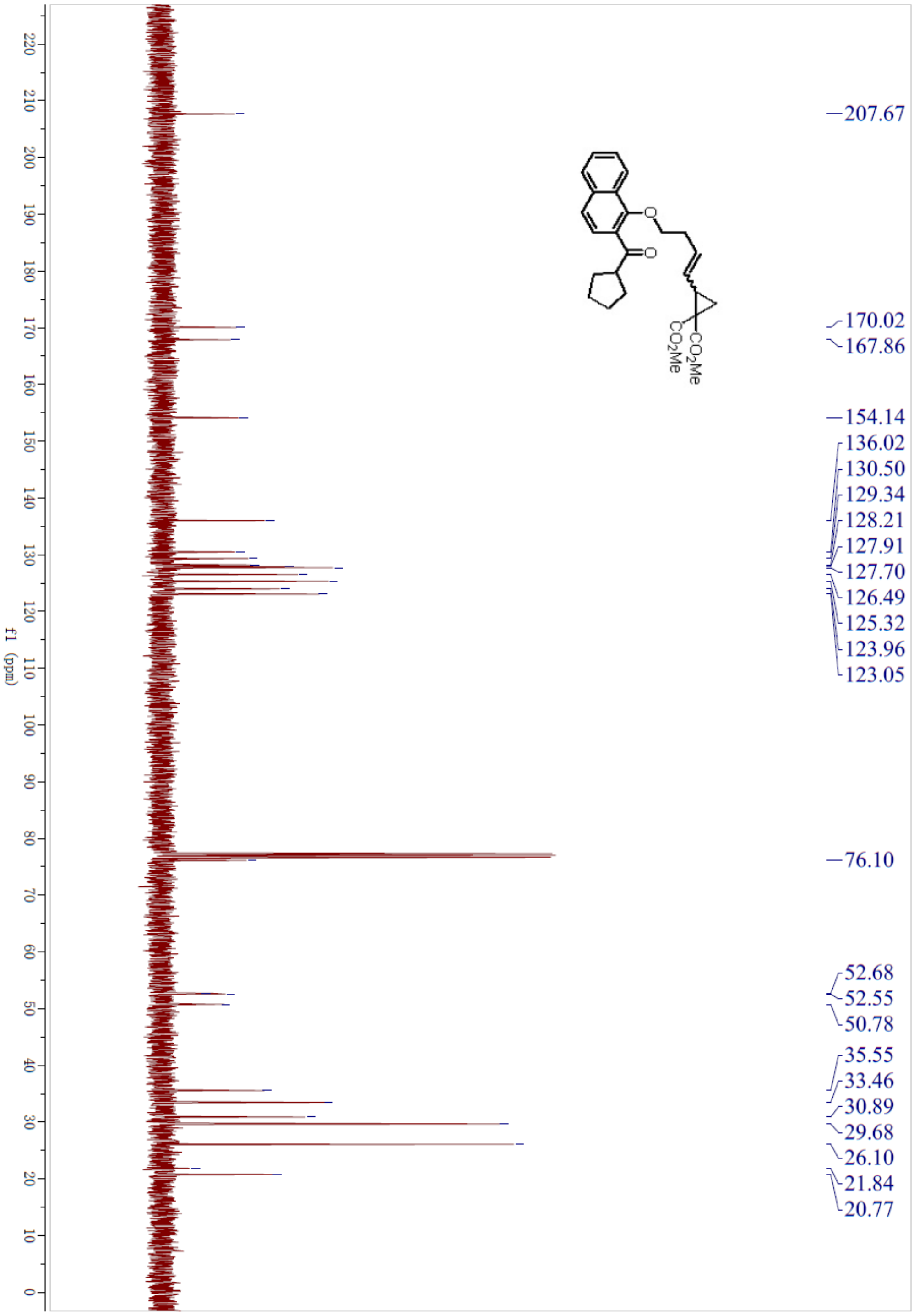

^{1}H NMR Spectrum (400 MHz, $CDCl_3$) of **1e**

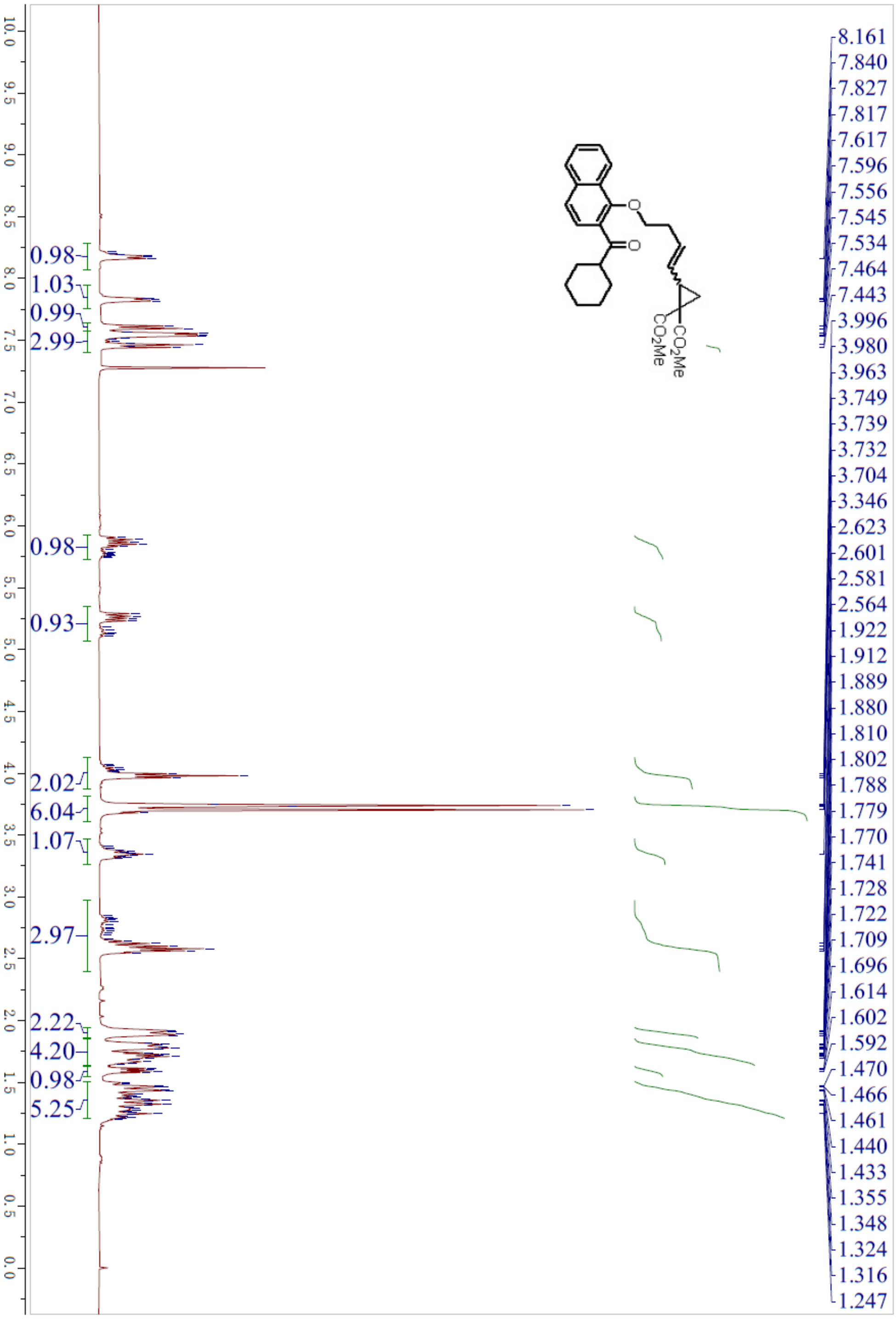

^{13}C NMR Spectrum (100 MHz, $CDCl_3$) of **1e**

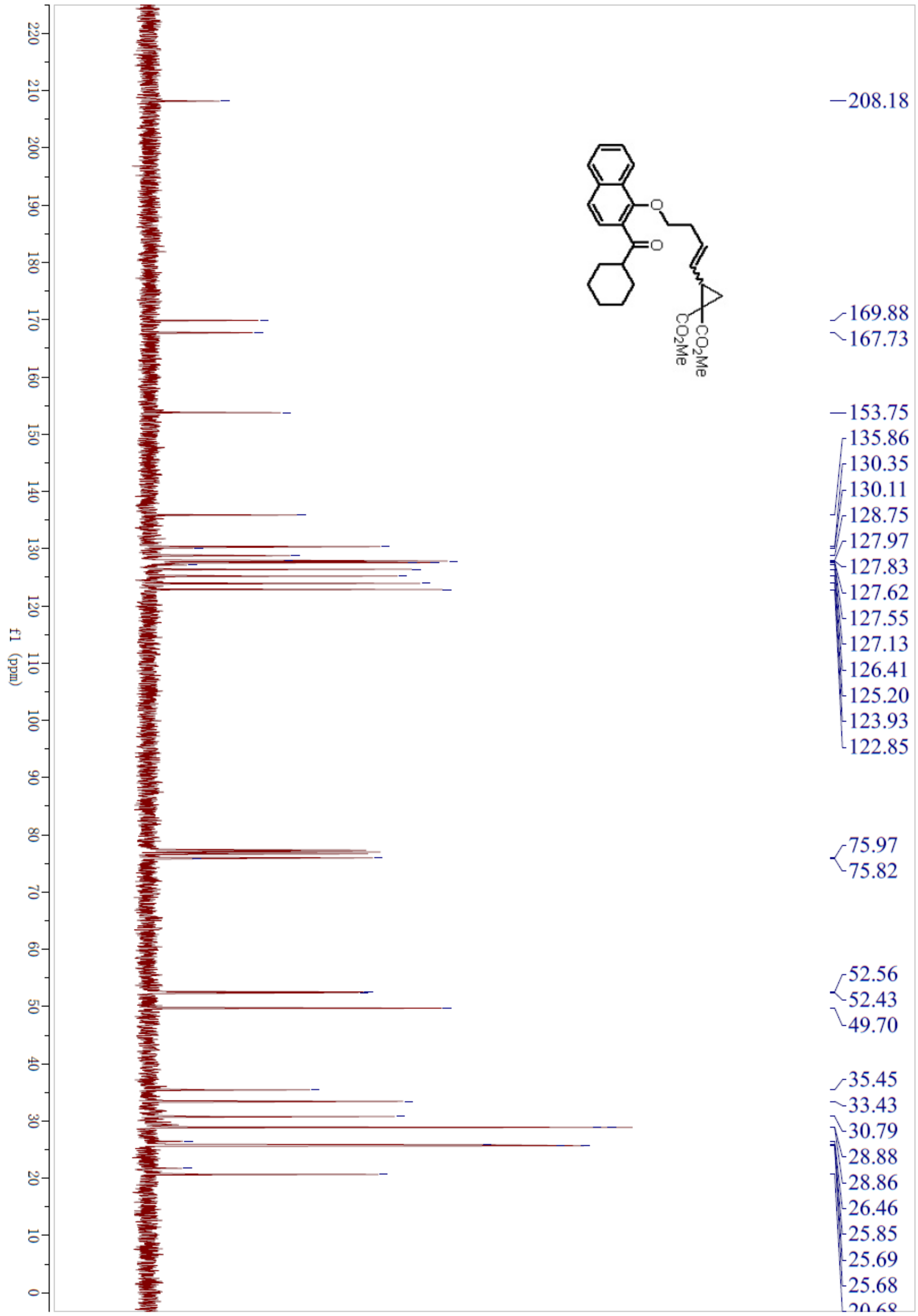

^{1}H NMR Spectrum (400 MHz, $CDCl_3$) of **1f**

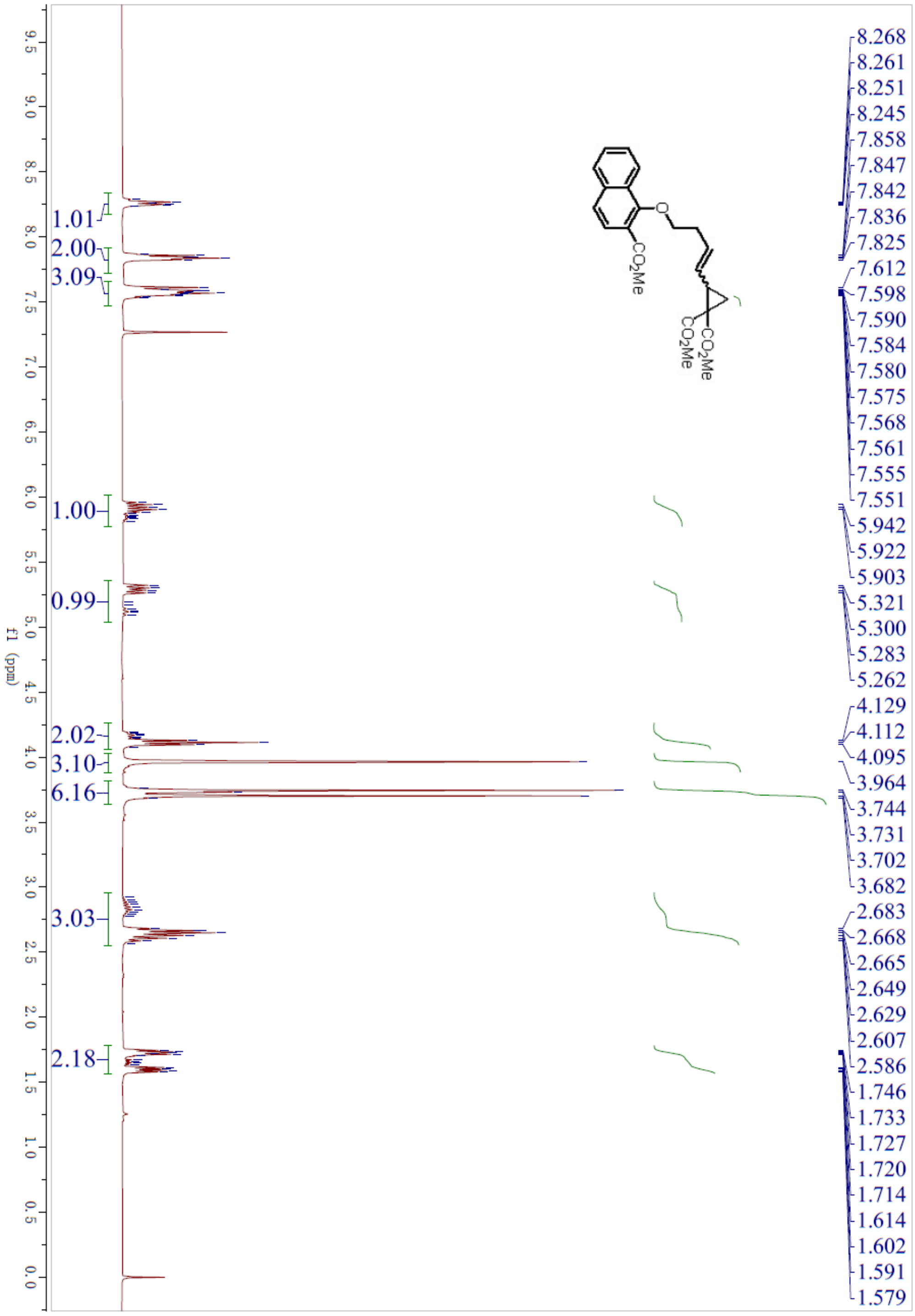

^{13}C NMR Spectrum (100 MHz, $CDCl_3$) of **1f**

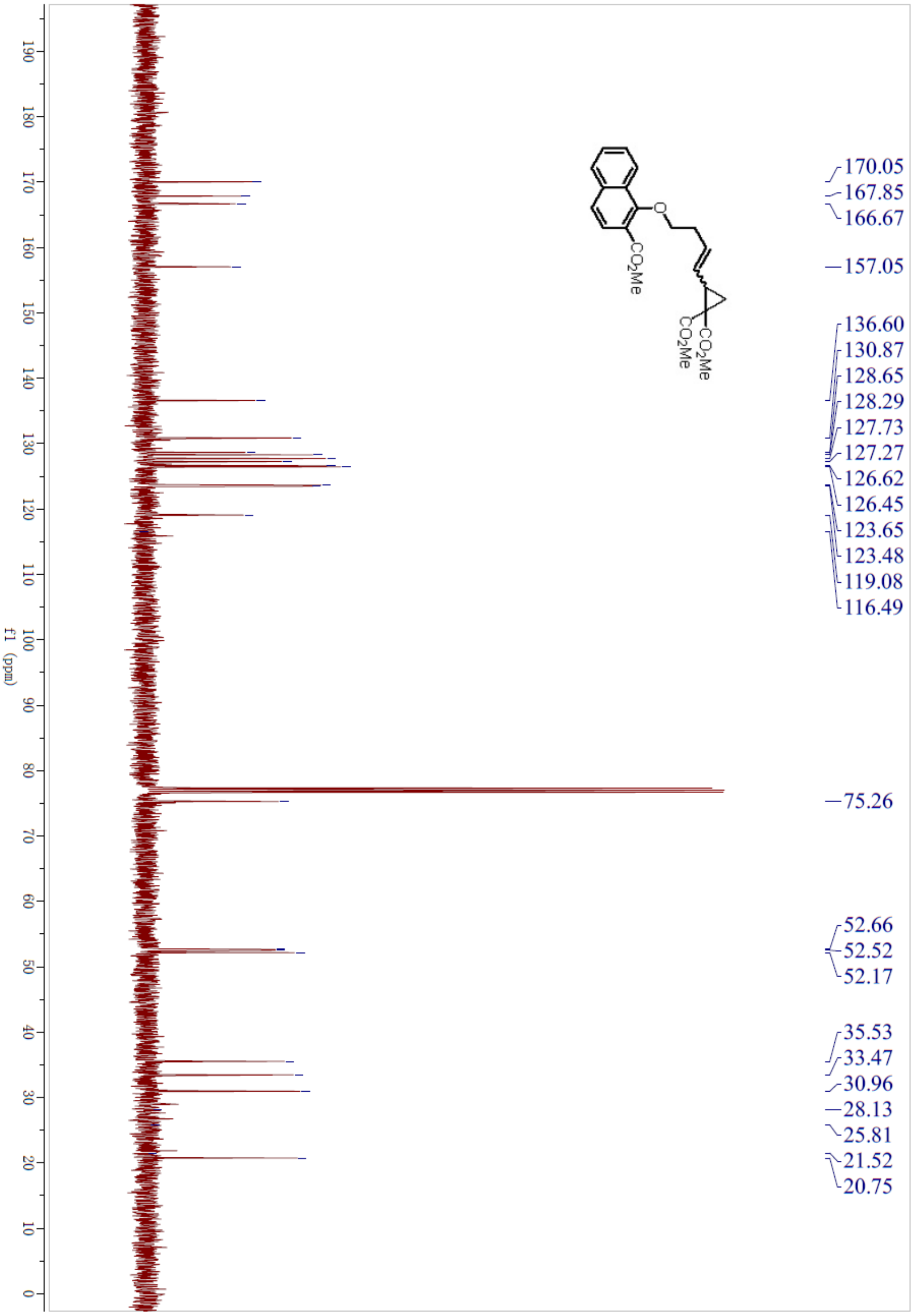

^{1}H NMR Spectrum (400 MHz, CDCl3) of **1g**

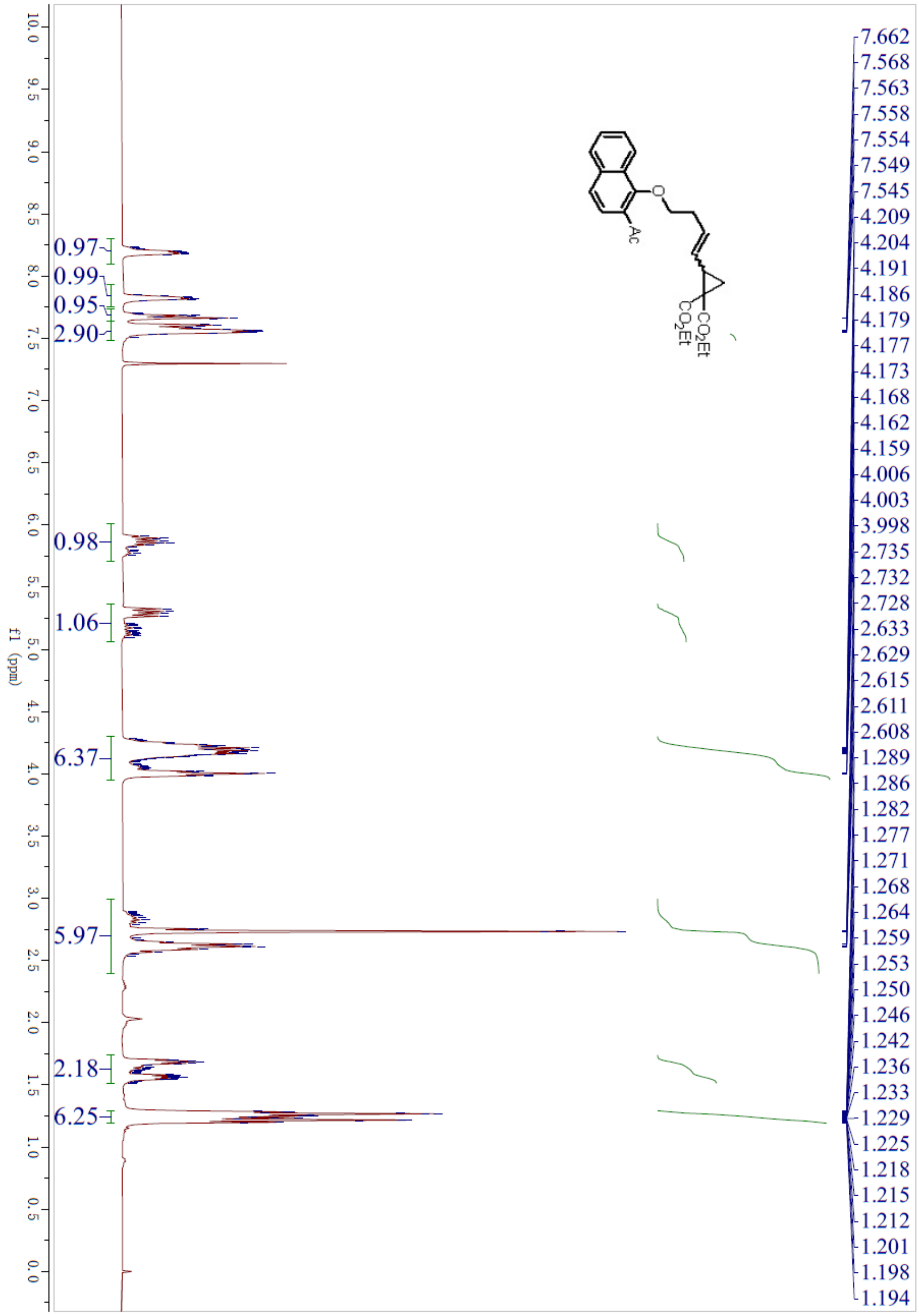

^{13}C NMR Spectrum (100 MHz, $CDCl_3$) of **1g**

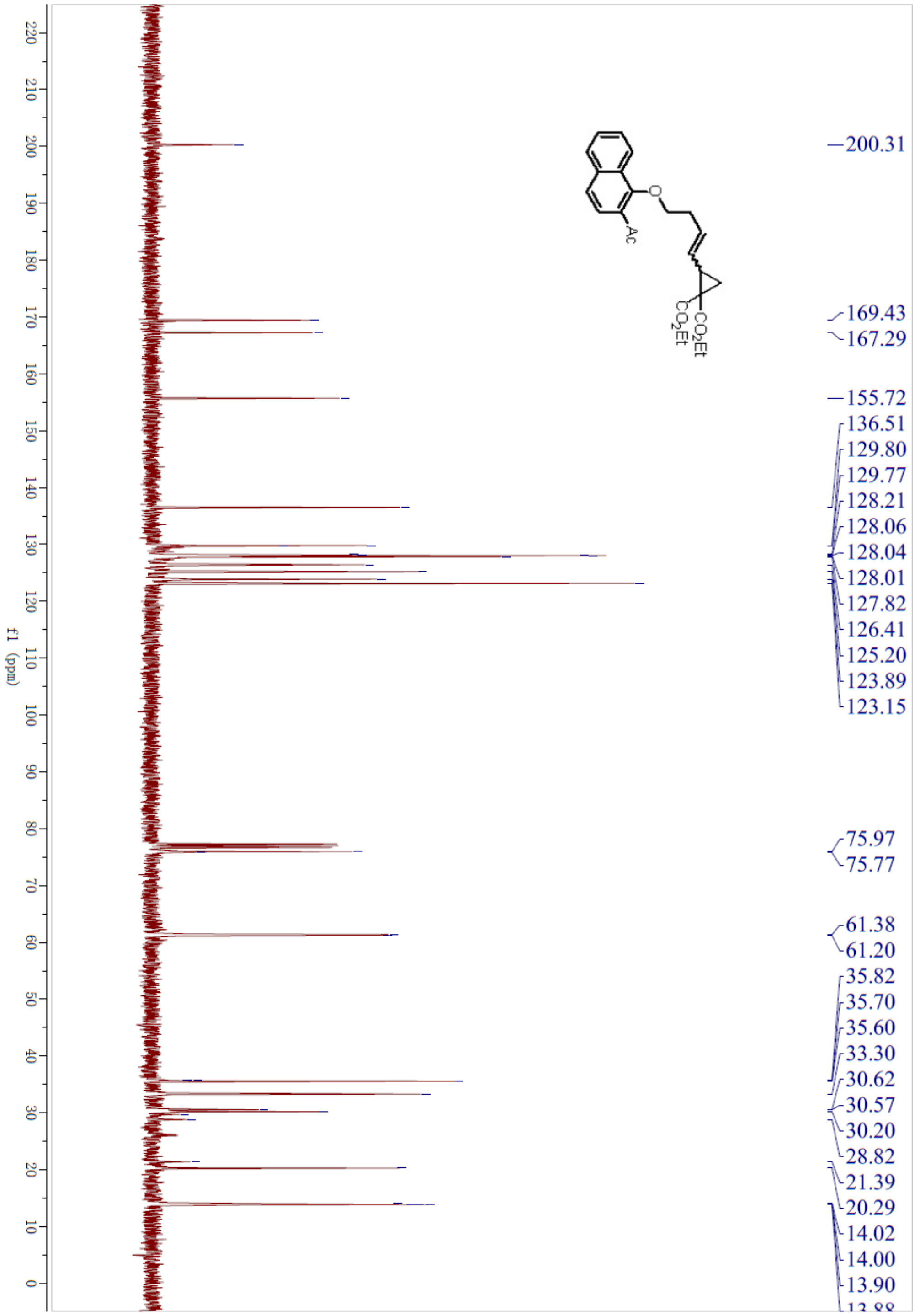

^{1}H NMR Spectrum (400 MHz, $CDCl_3$) of **1h**

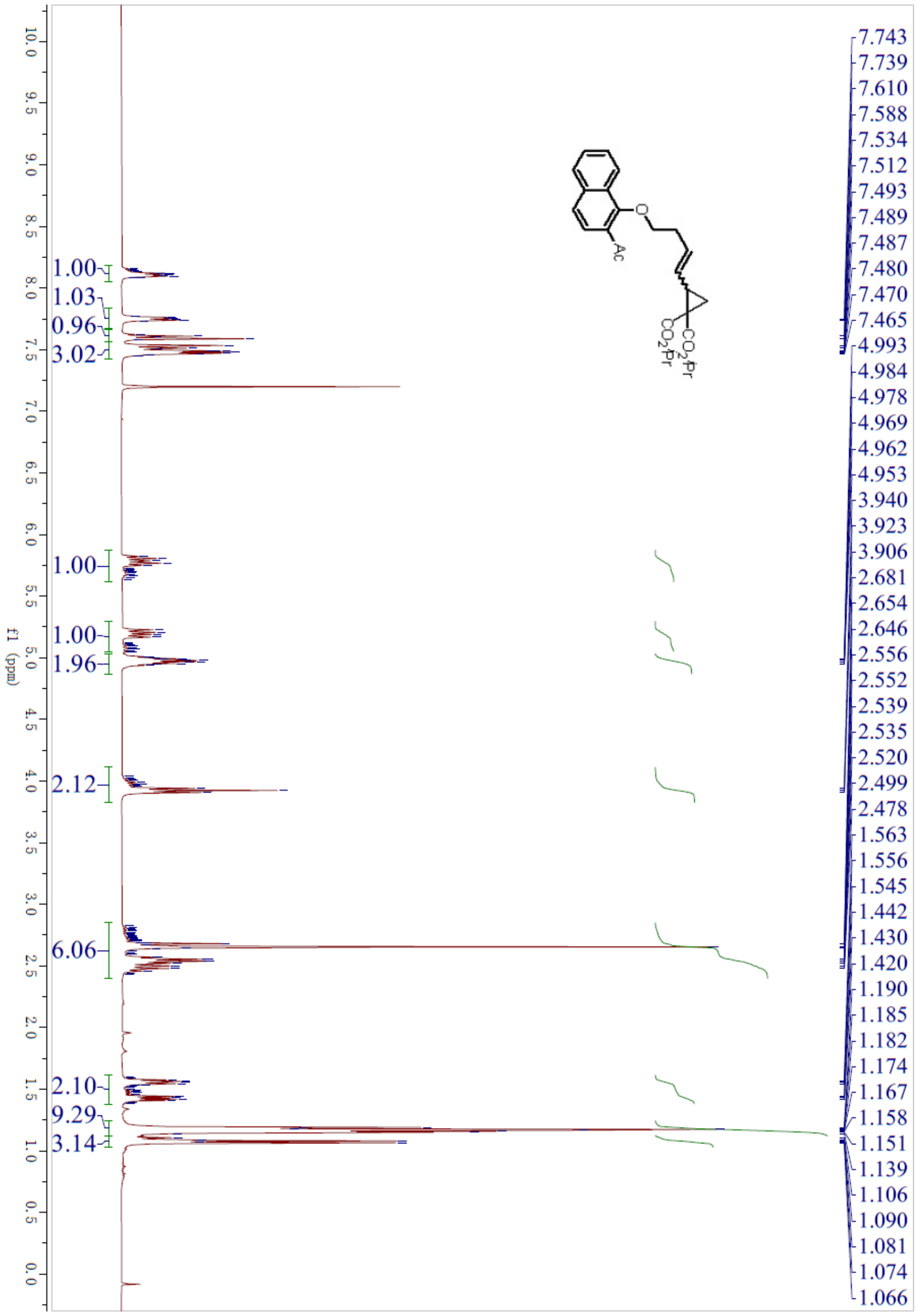

^{13}C NMR Spectrum (100 MHz, $CDCl_3$) of **1h**

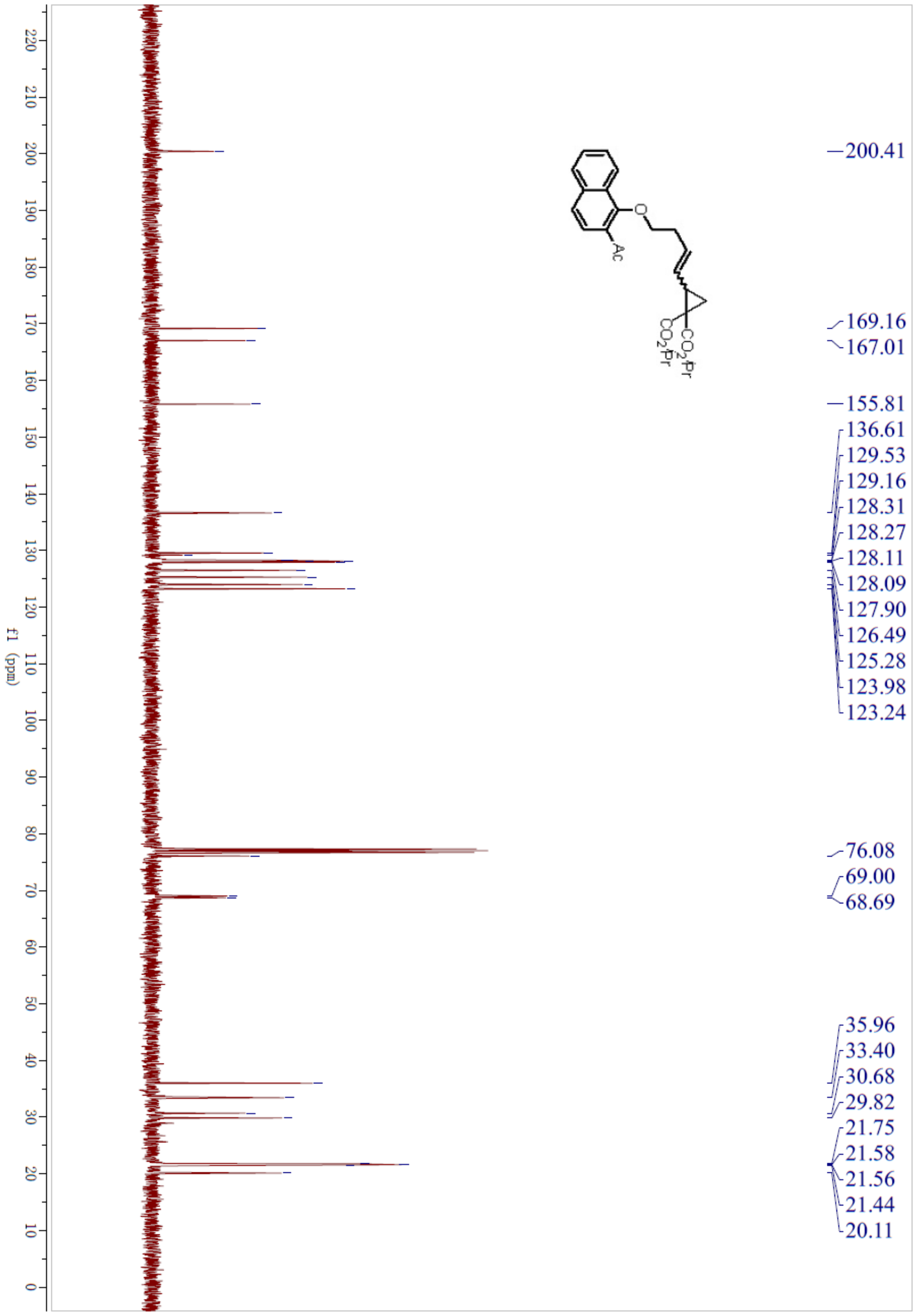

^{1}H NMR Spectrum (400 MHz, $CDCl_3$) of **1i**

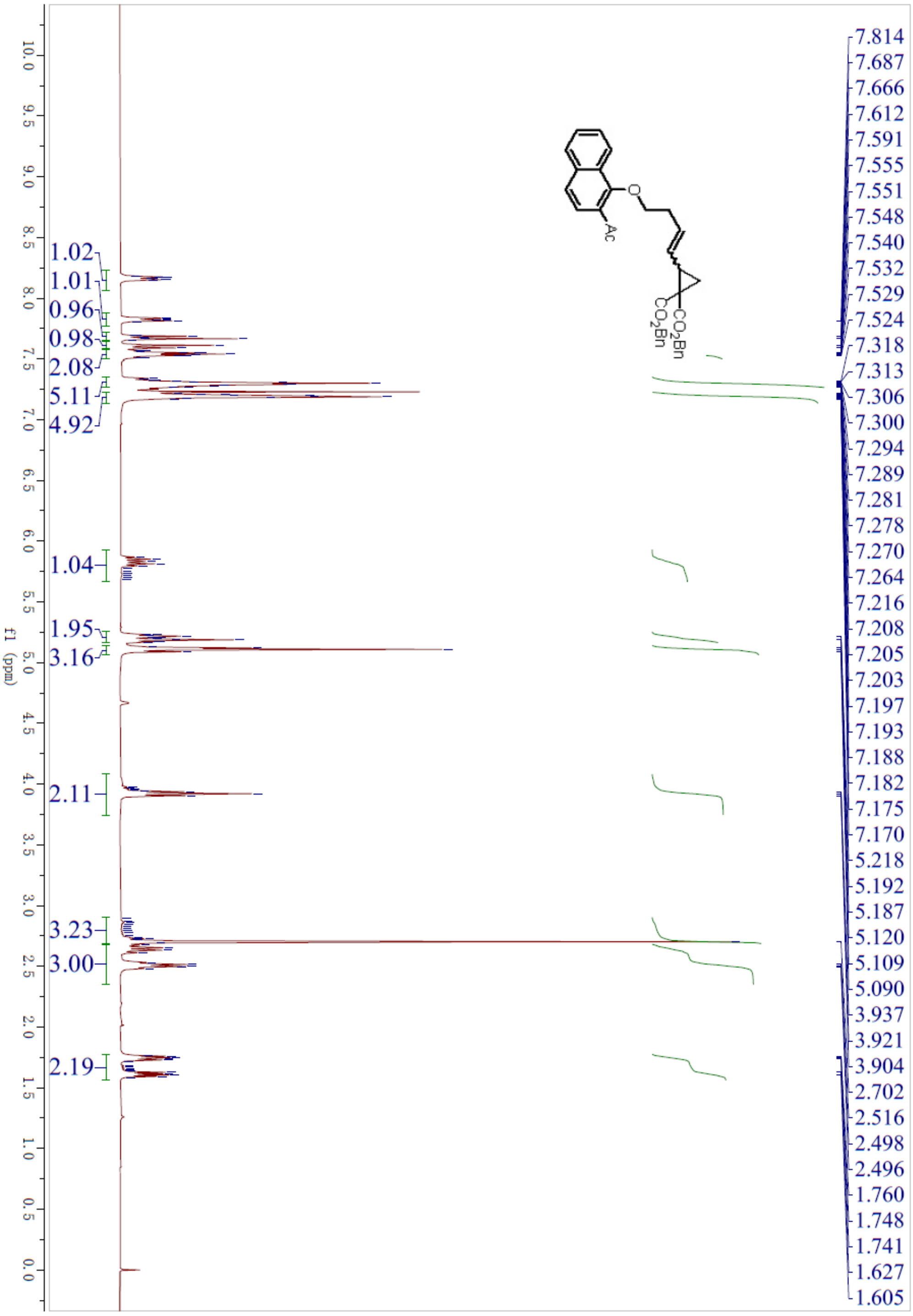

^{13}C NMR Spectrum (100 MHz, $CDCl_3$) of **1i**

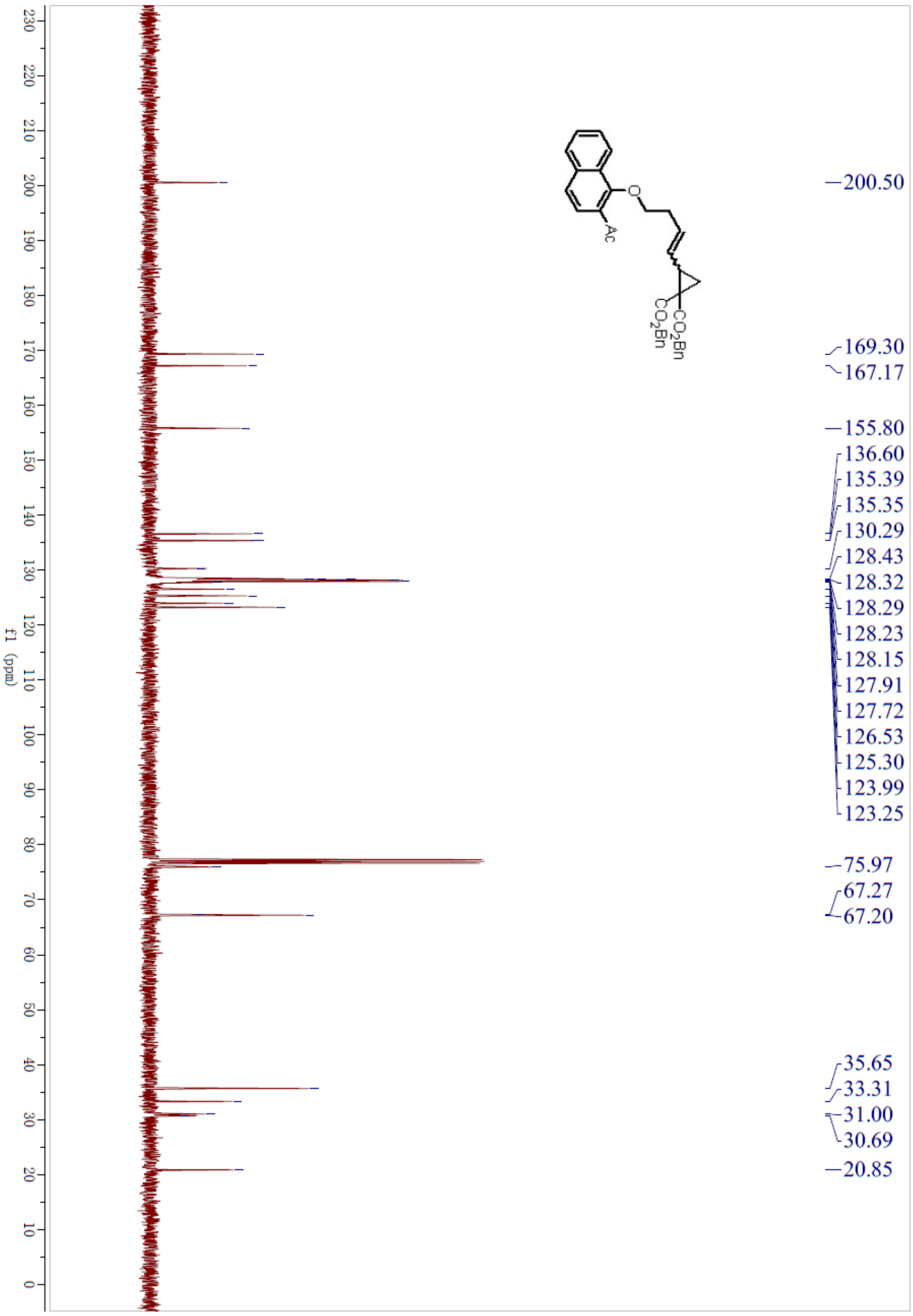

^{1}H NMR Spectrum (400 MHz, $CDCl_3$) of **1j**

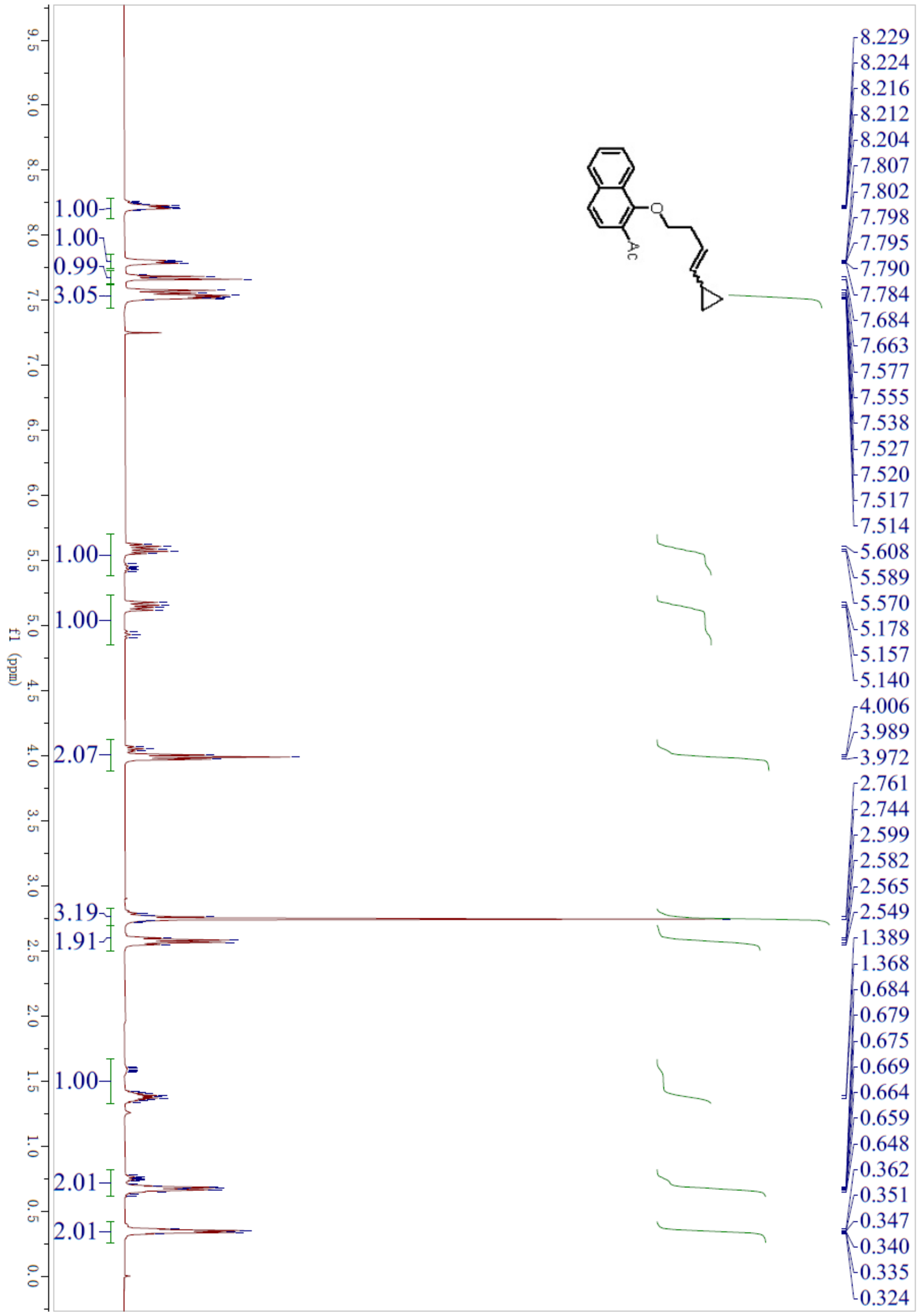

^{13}C NMR Spectrum (100 MHz, CDCl3) of **1j**

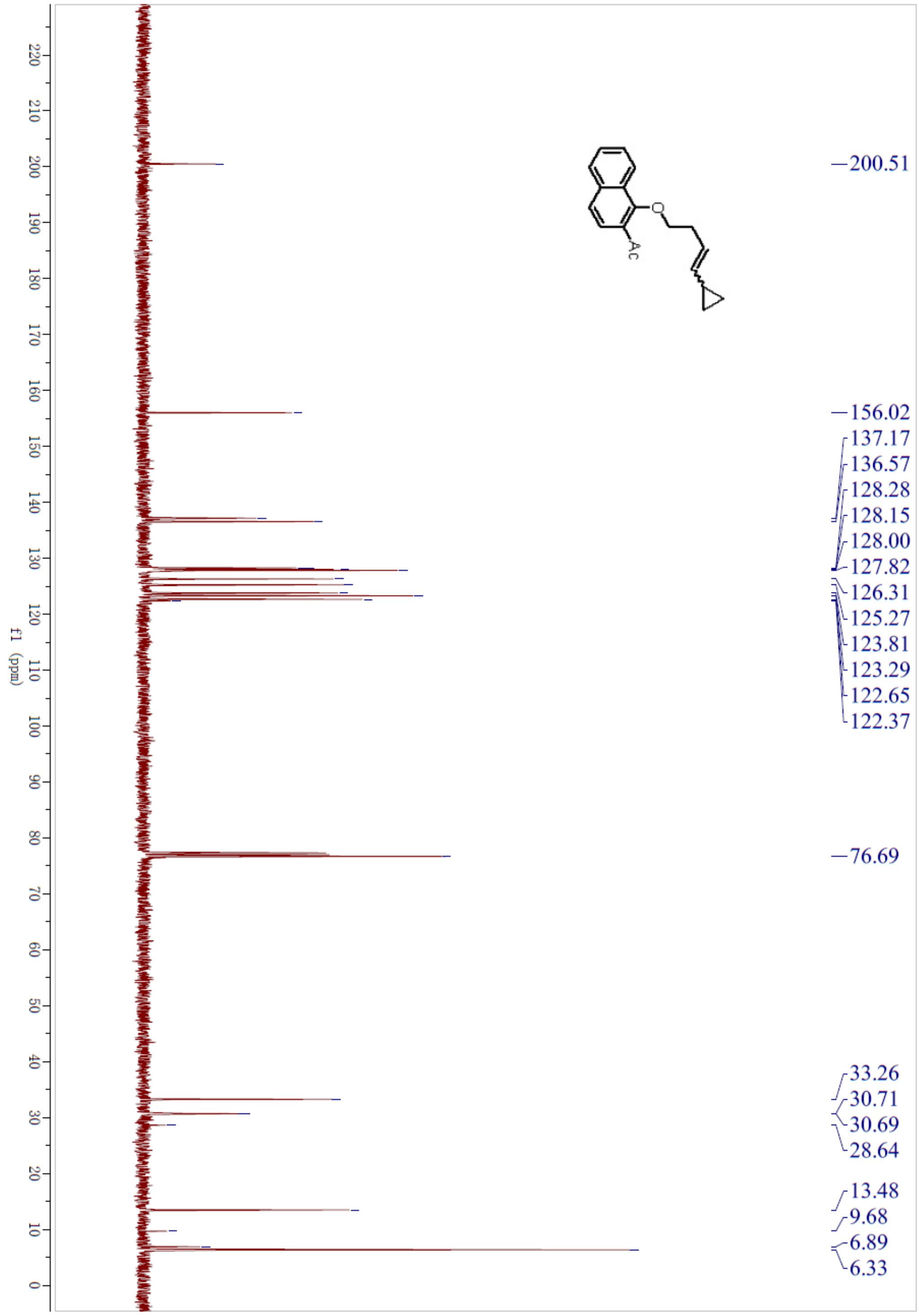

^{1}H NMR Spectrum (400 MHz, $CDCl_3$) of **1k**

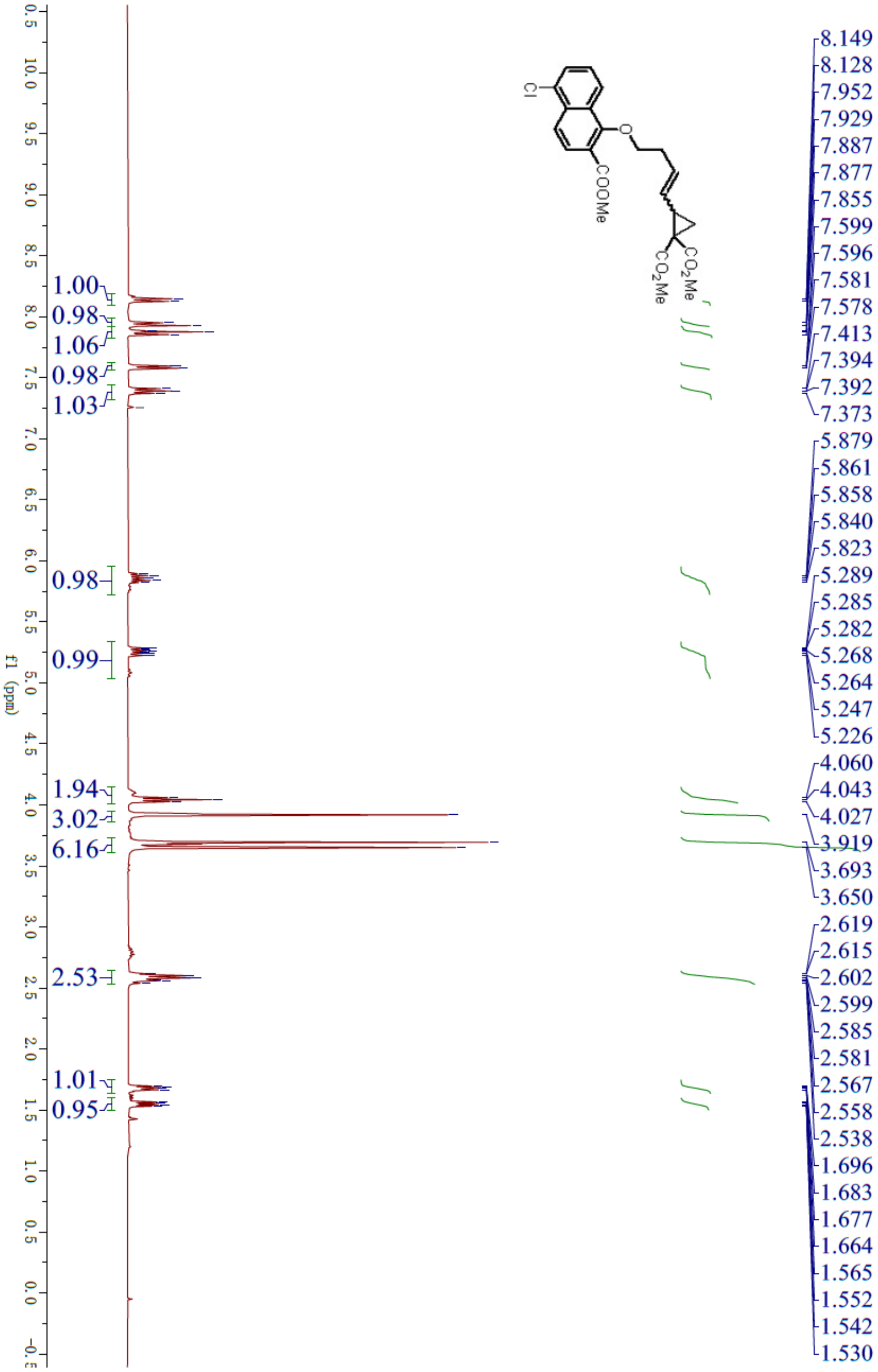

^{13}C NMR Spectrum (100 MHz, $CDCl_3$) of **1k**

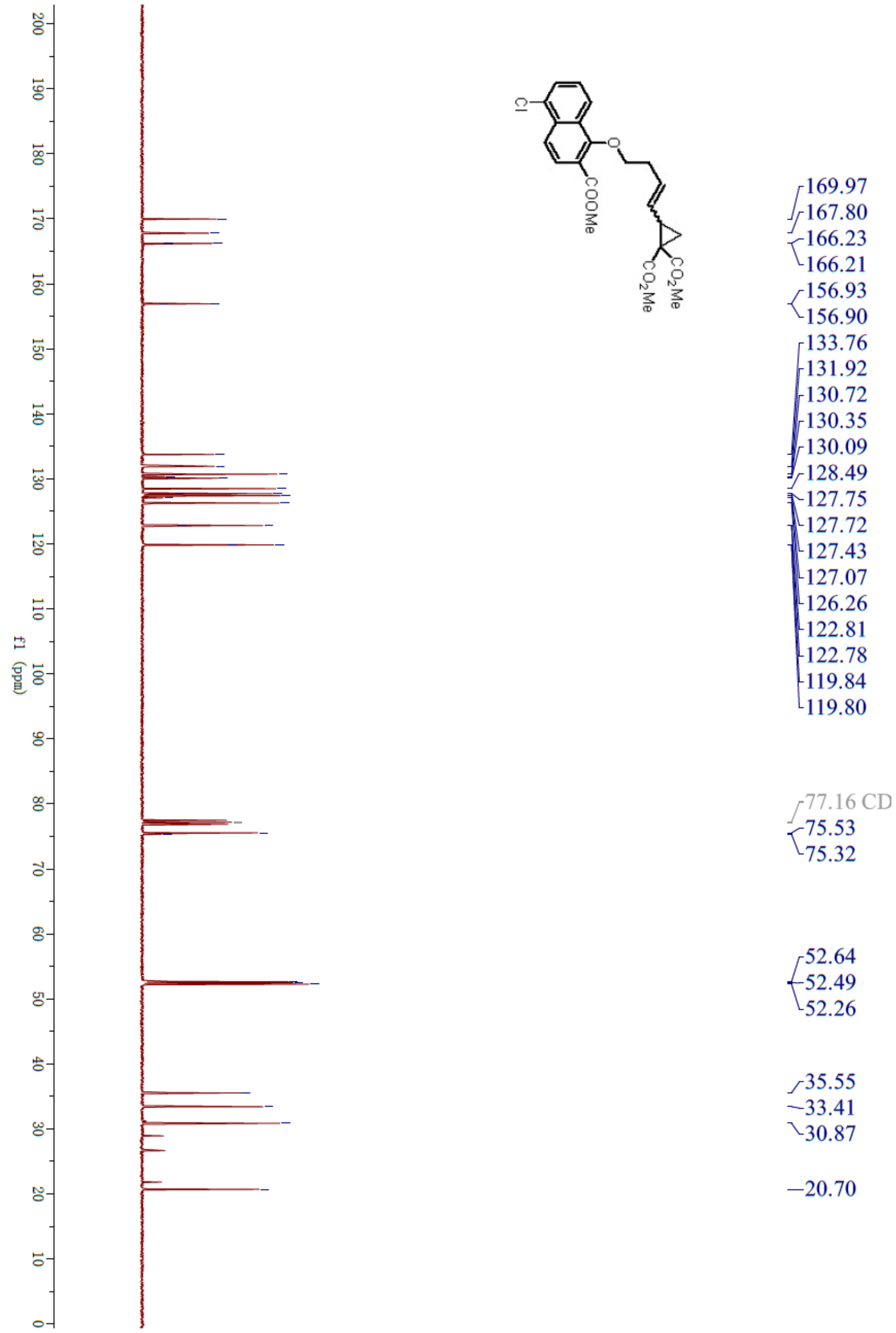

^{1}H NMR Spectrum (400 MHz, CDCl3) of **1l**

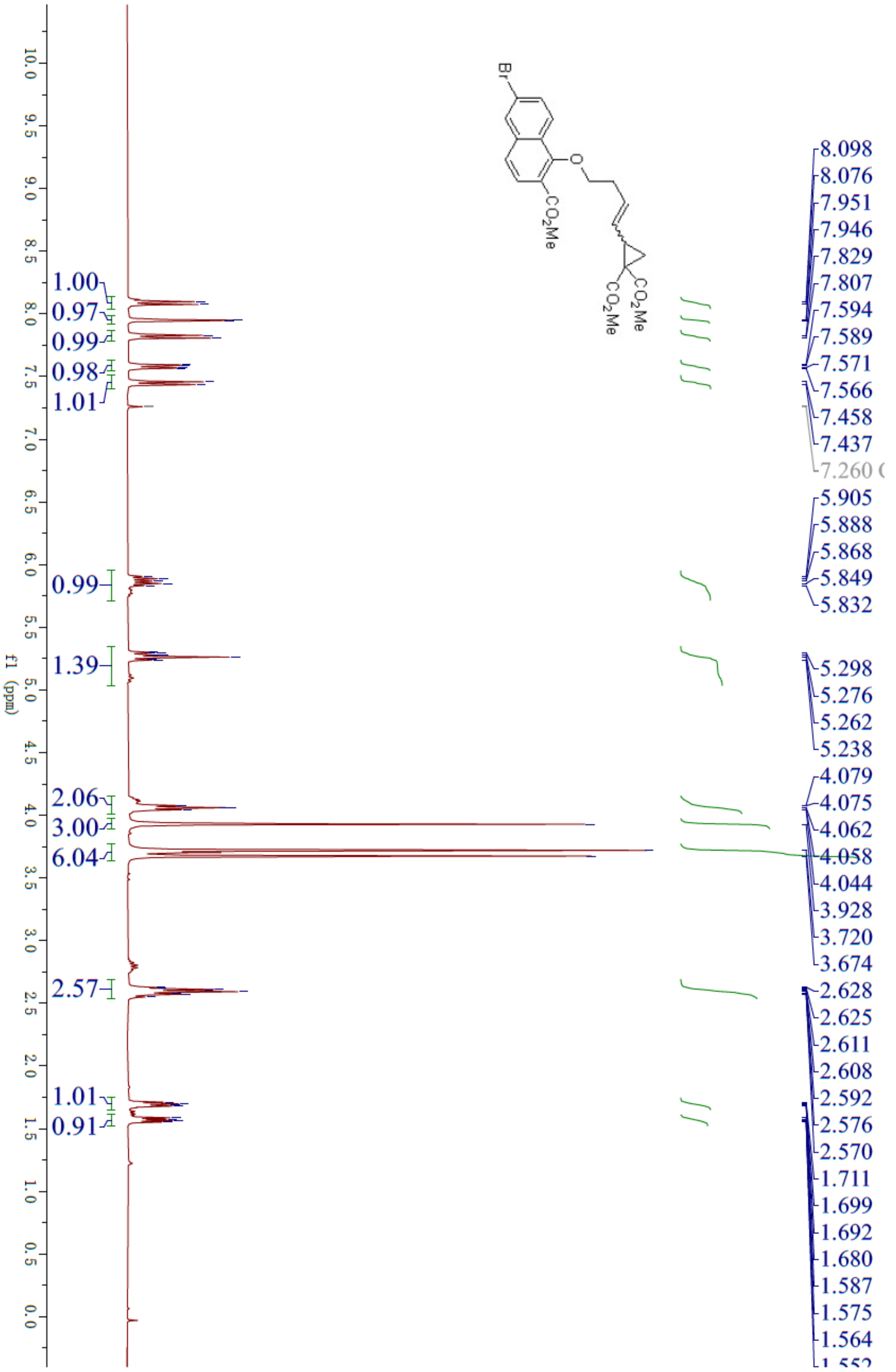

^{13}C NMR Spectrum (100 MHz, $CDCl_3$) of **1l**

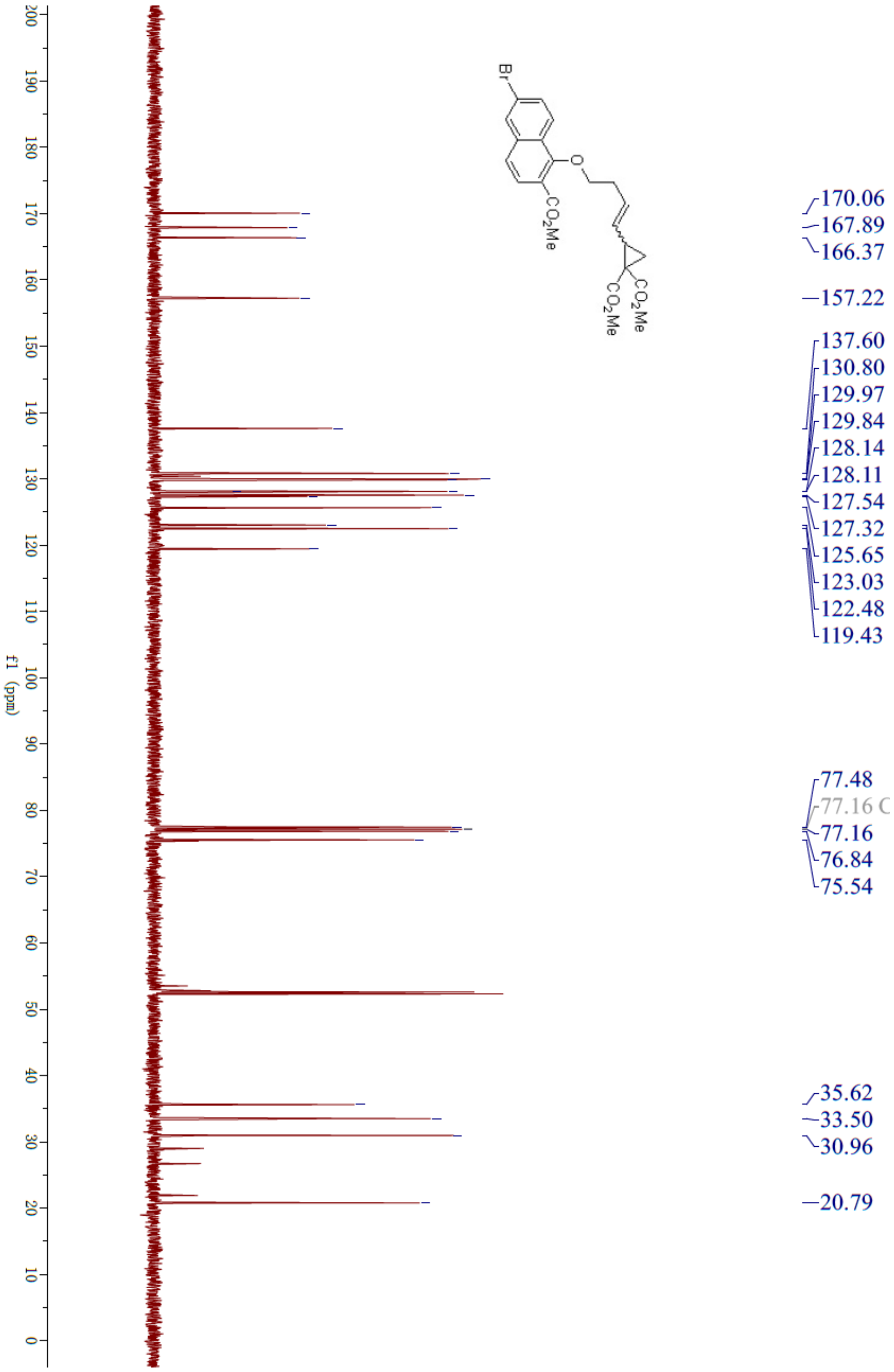

^{1}H NMR Spectrum (400 MHz, $CDCl_3$) of **1m**

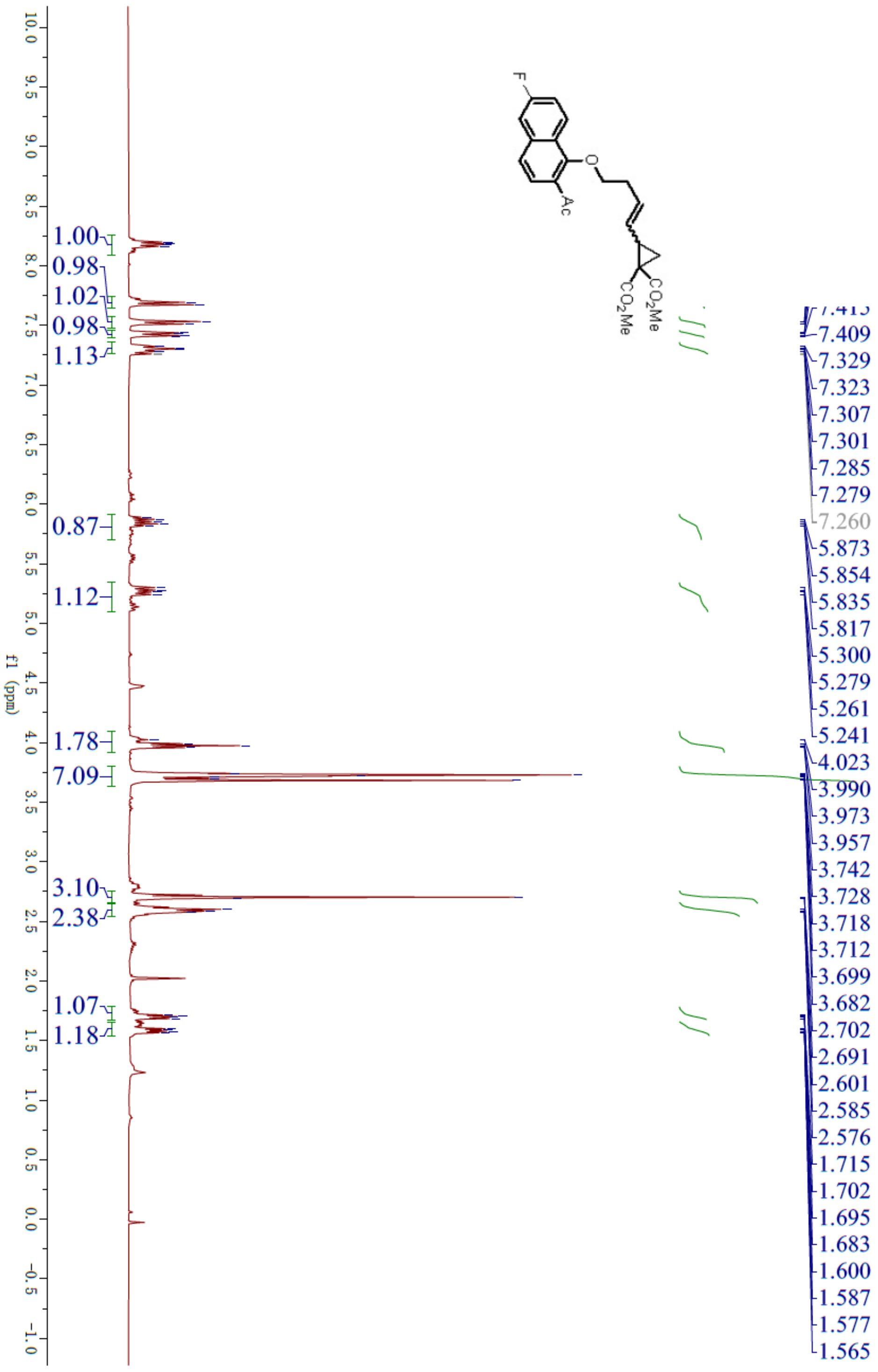

^{13}C NMR Spectrum (100 MHz, CDCl3) of **1m**

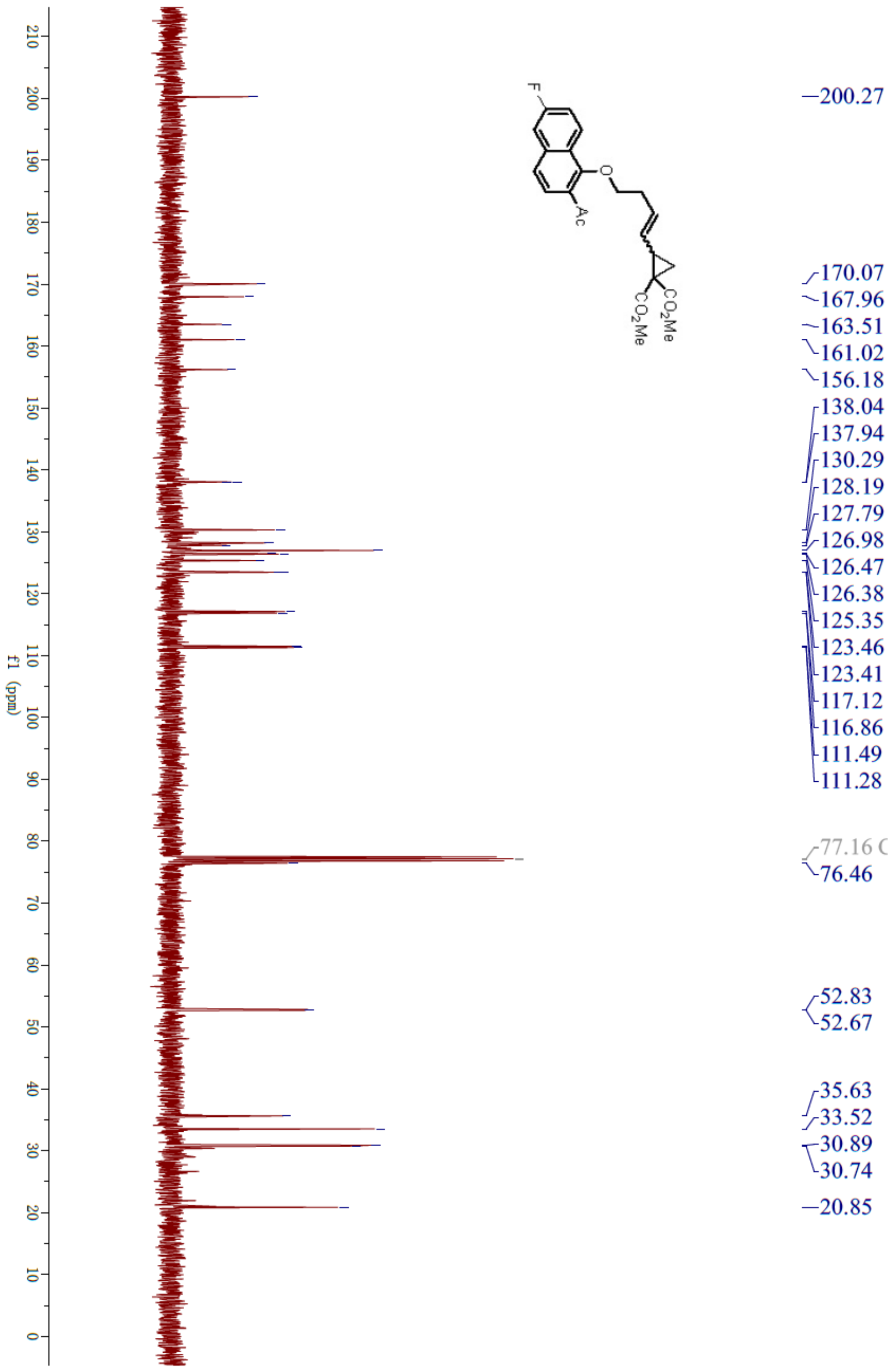

^{19}F NMR Spectrum (376 MHz, $CDCl_3$) of **1m**

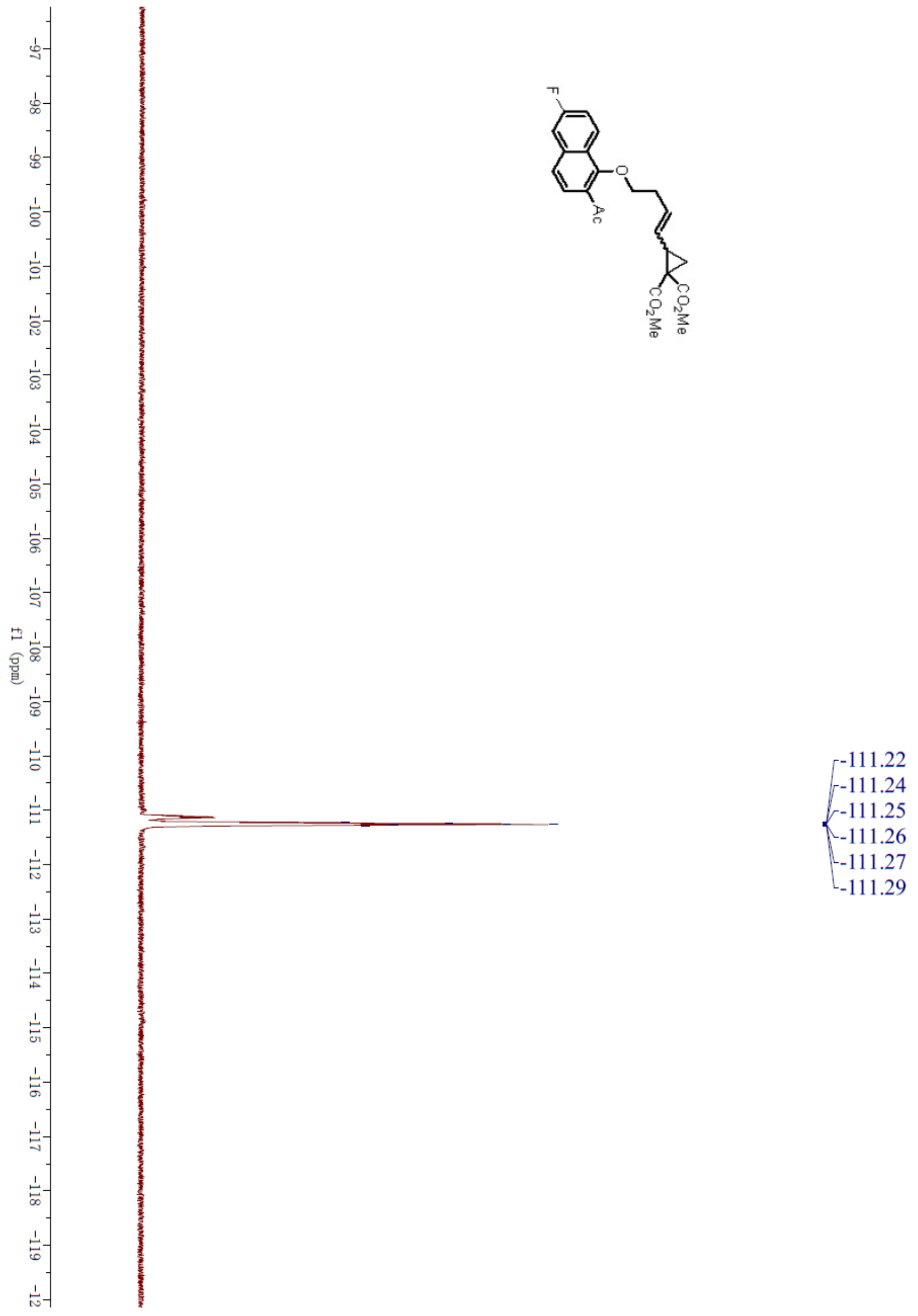

^{1}H NMR Spectrum (400 MHz, $CDCl_3$) of **1n**

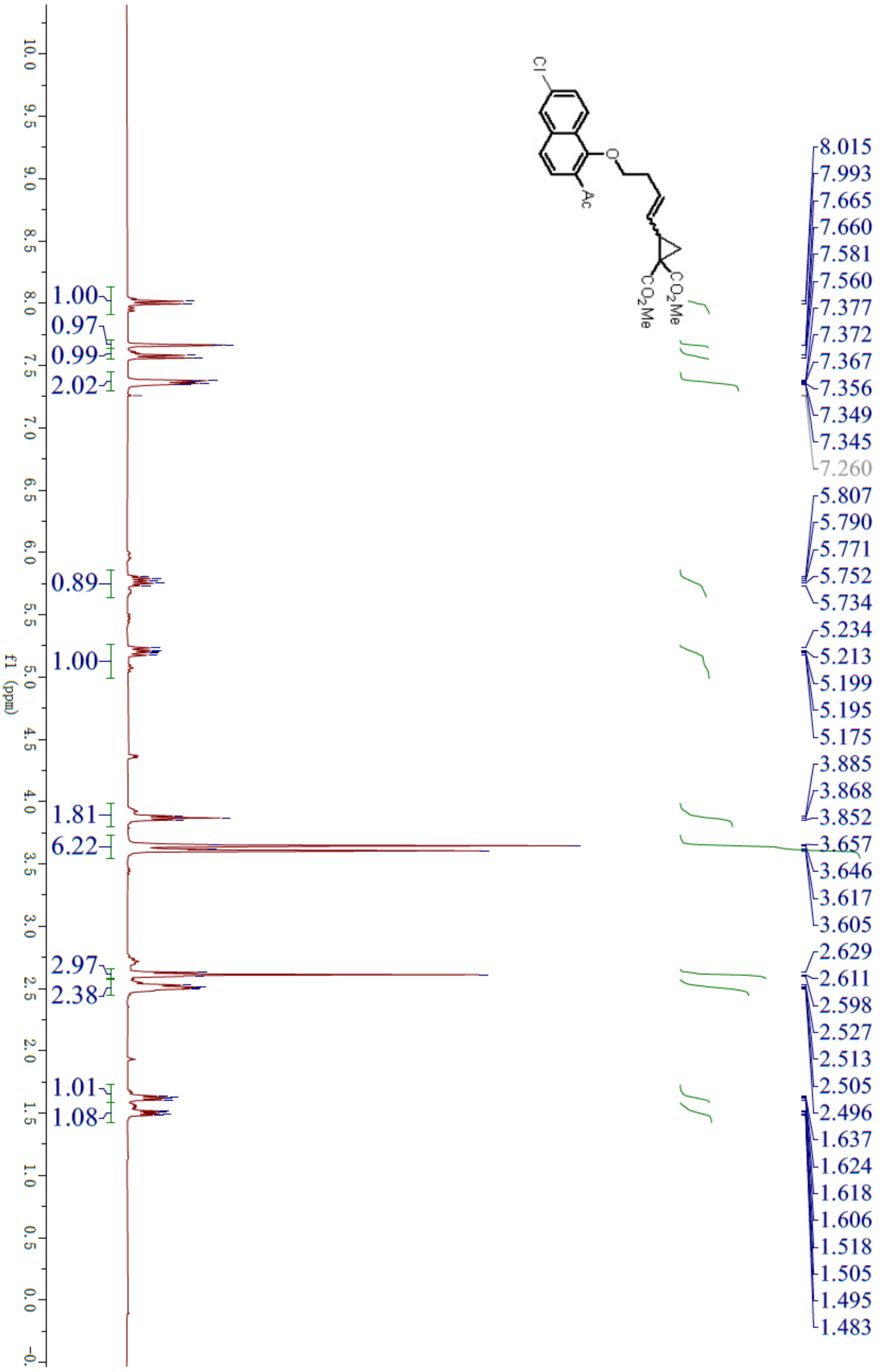

^{13}C NMR Spectrum (100 MHz, $CDCl_3$) of **1n**

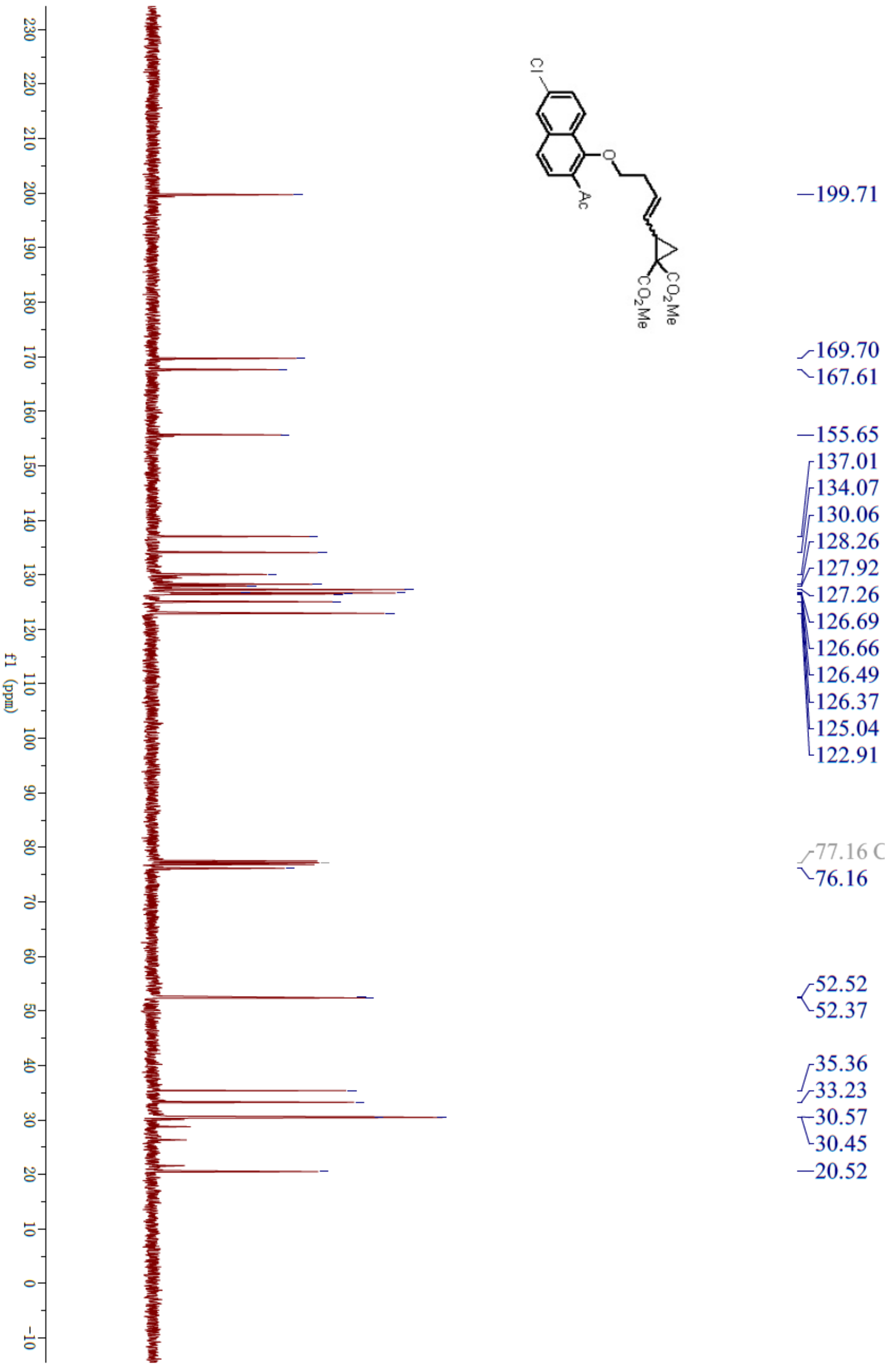

^{1}H NMR Spectrum (400 MHz, $CDCl_3$) of **1o**

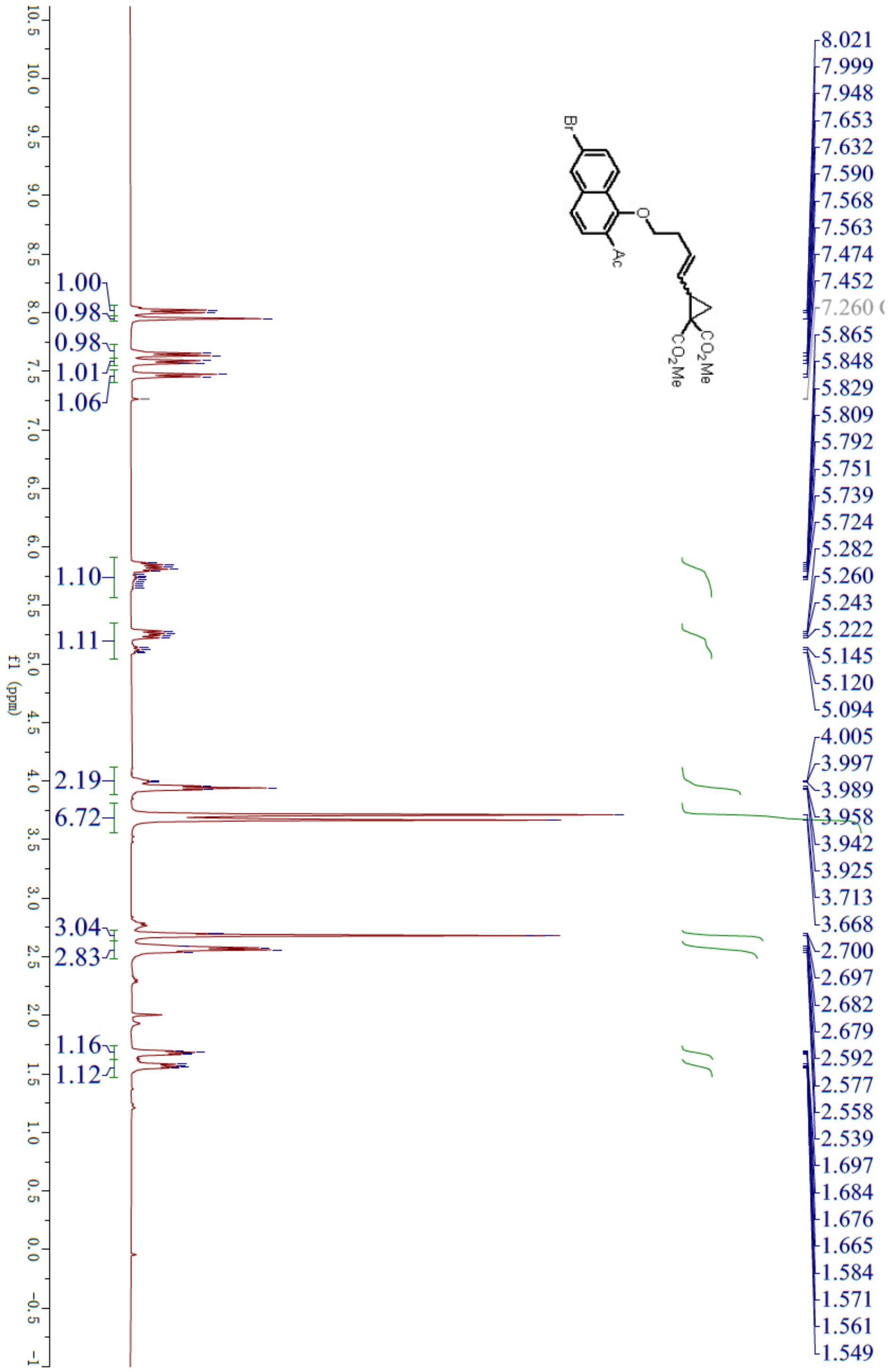

^{13}C NMR Spectrum (100 MHz, $CDCl_3$) of **1o**

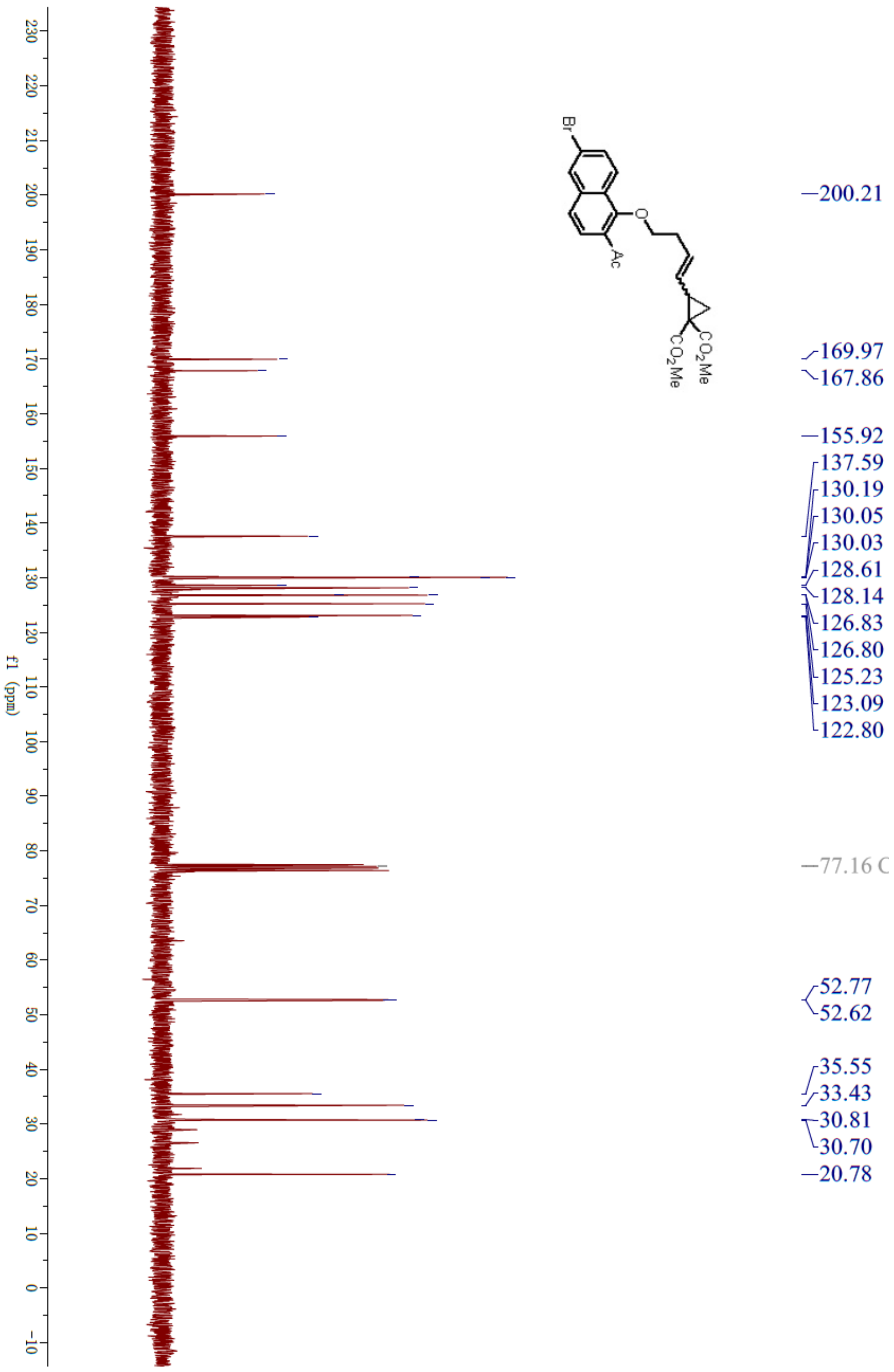

^{1}H NMR Spectrum (400 MHz, $CDCl_3$) of **1p**

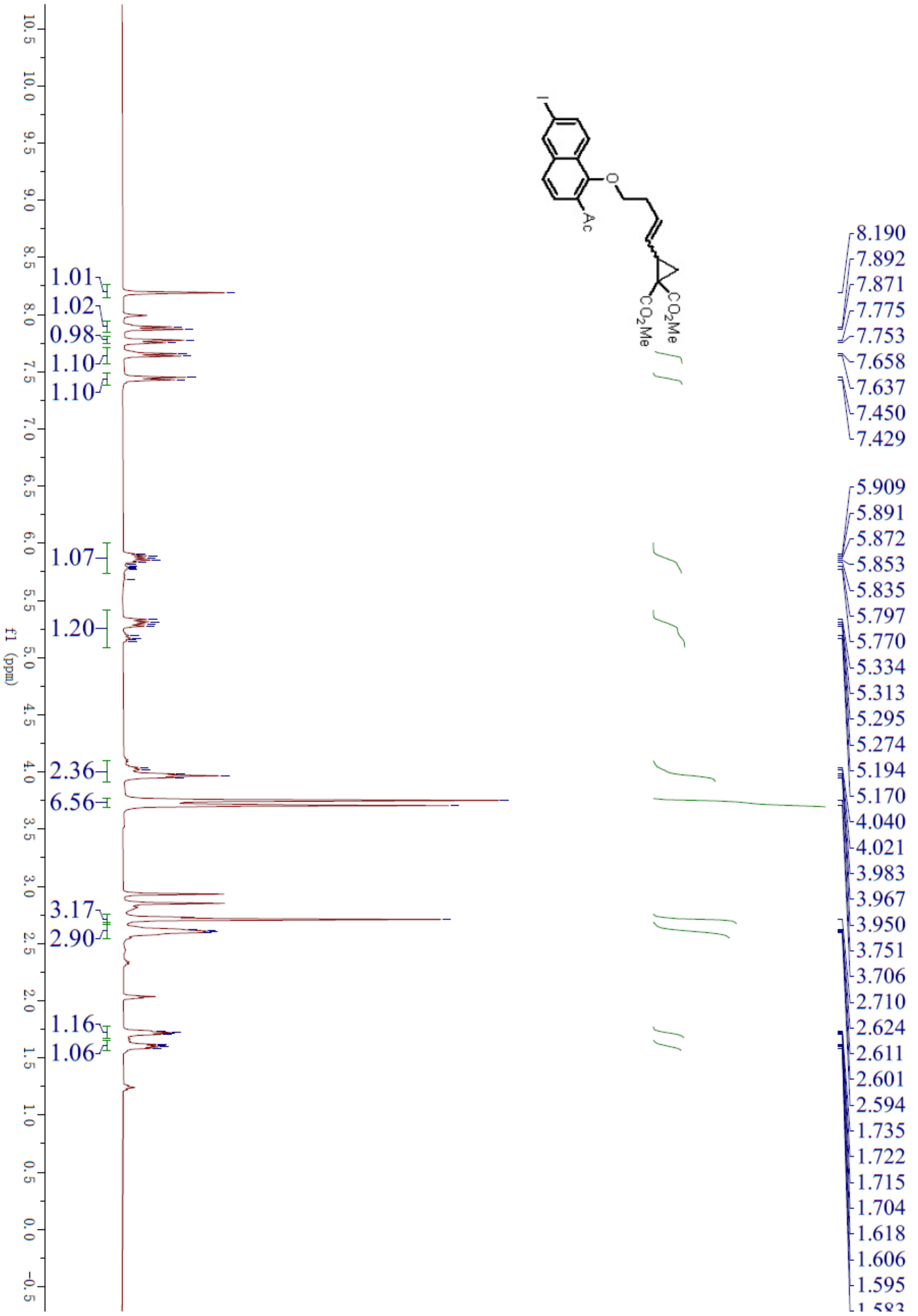

^{13}C NMR Spectrum (100 MHz, $CDCl_3$) of **1p**

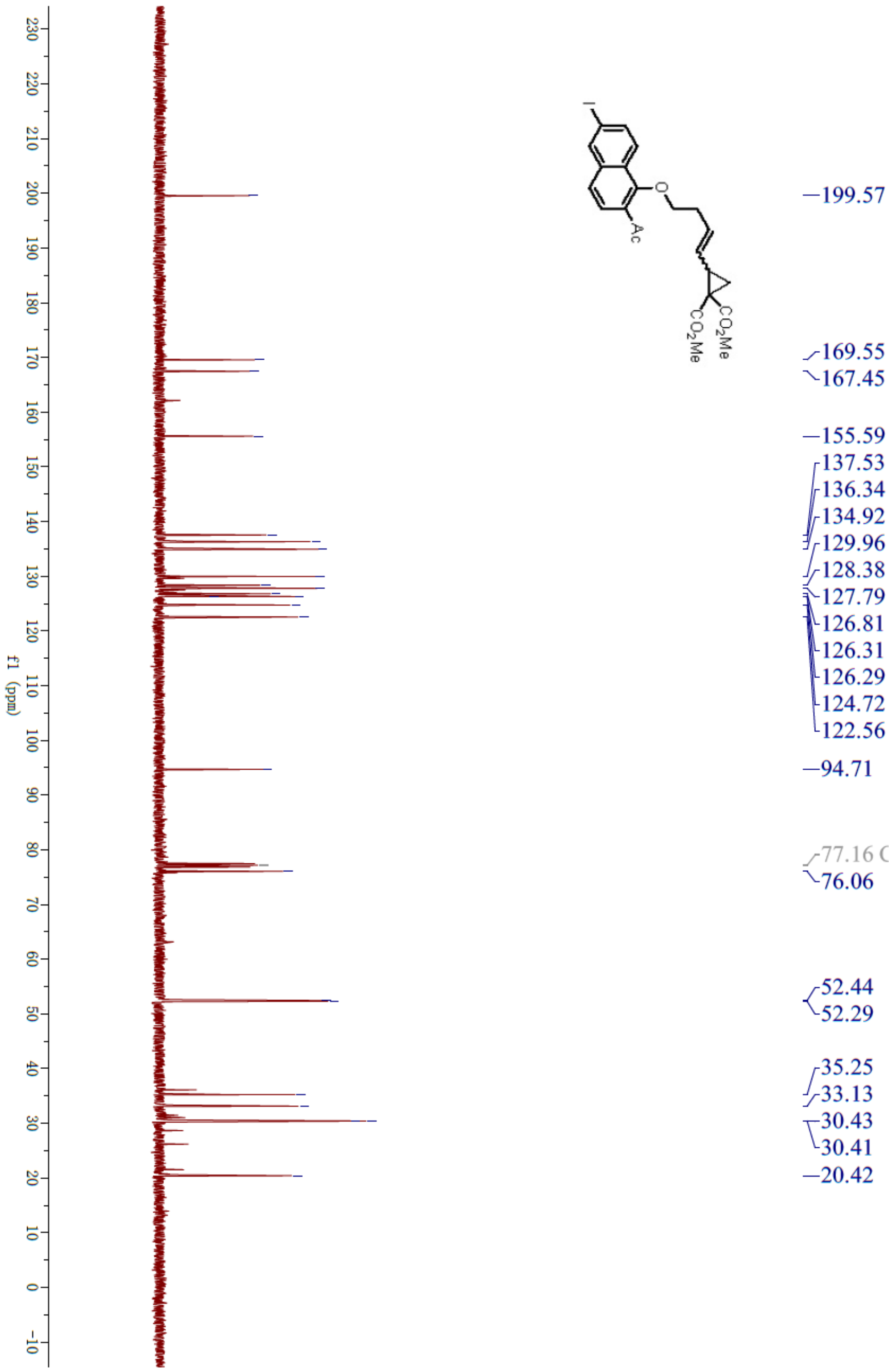

^{1}H NMR Spectrum (400 MHz, $CDCl_3$) of **1q**

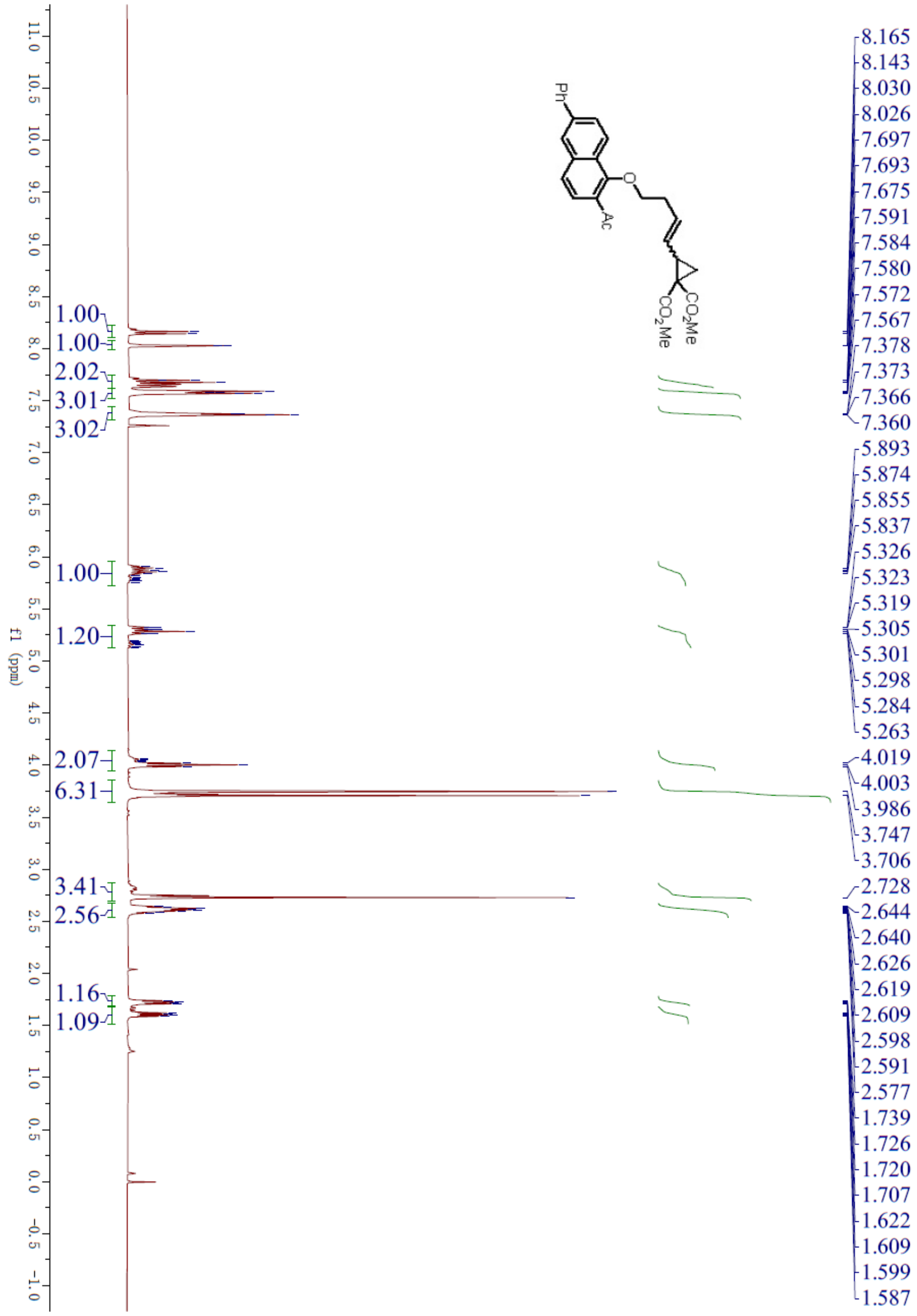

^{13}C NMR Spectrum (100 MHz, $CDCl_3$) of **1q**

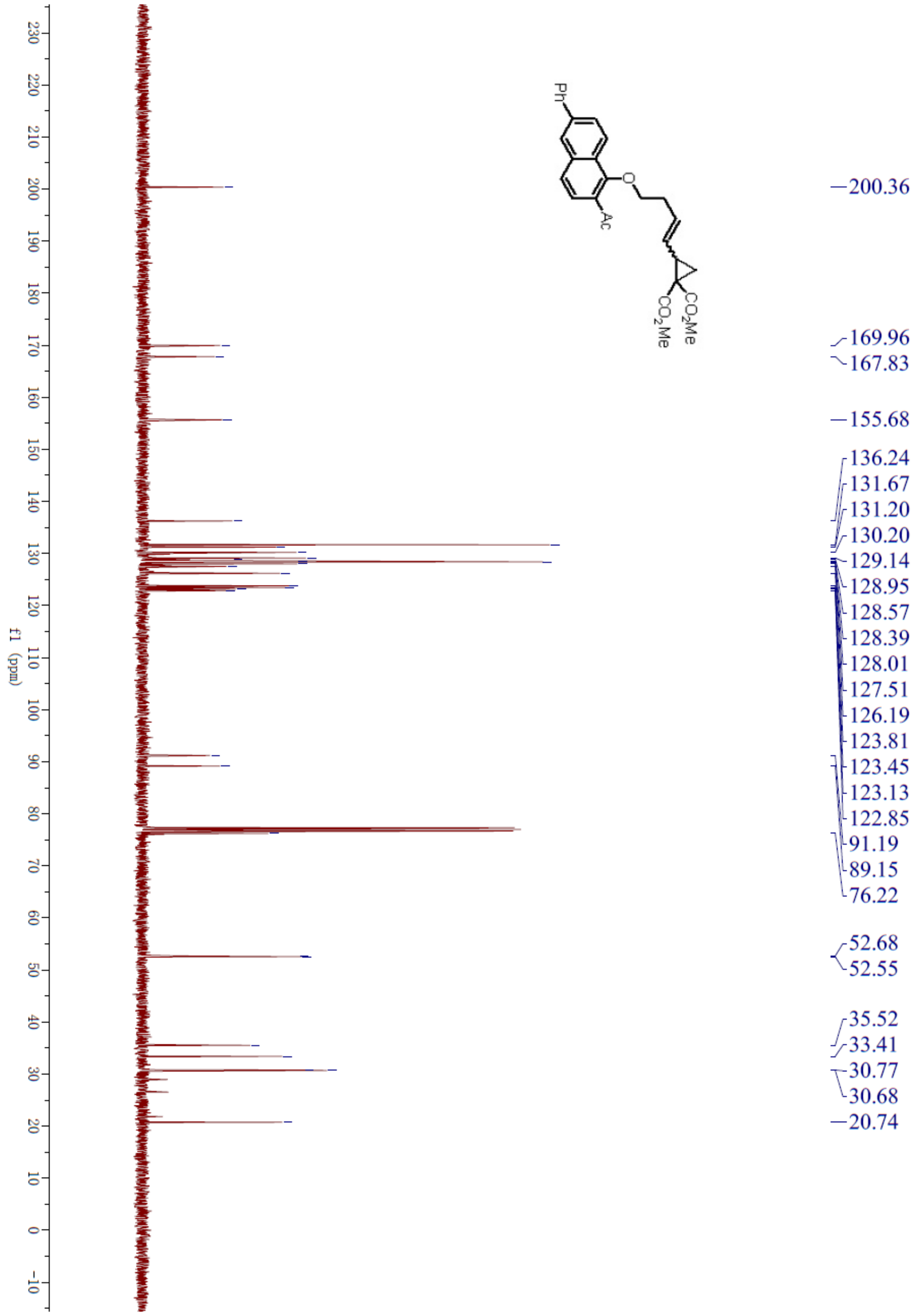

^{1}H NMR Spectrum (400 MHz, $CDCl_3$) of **1r**

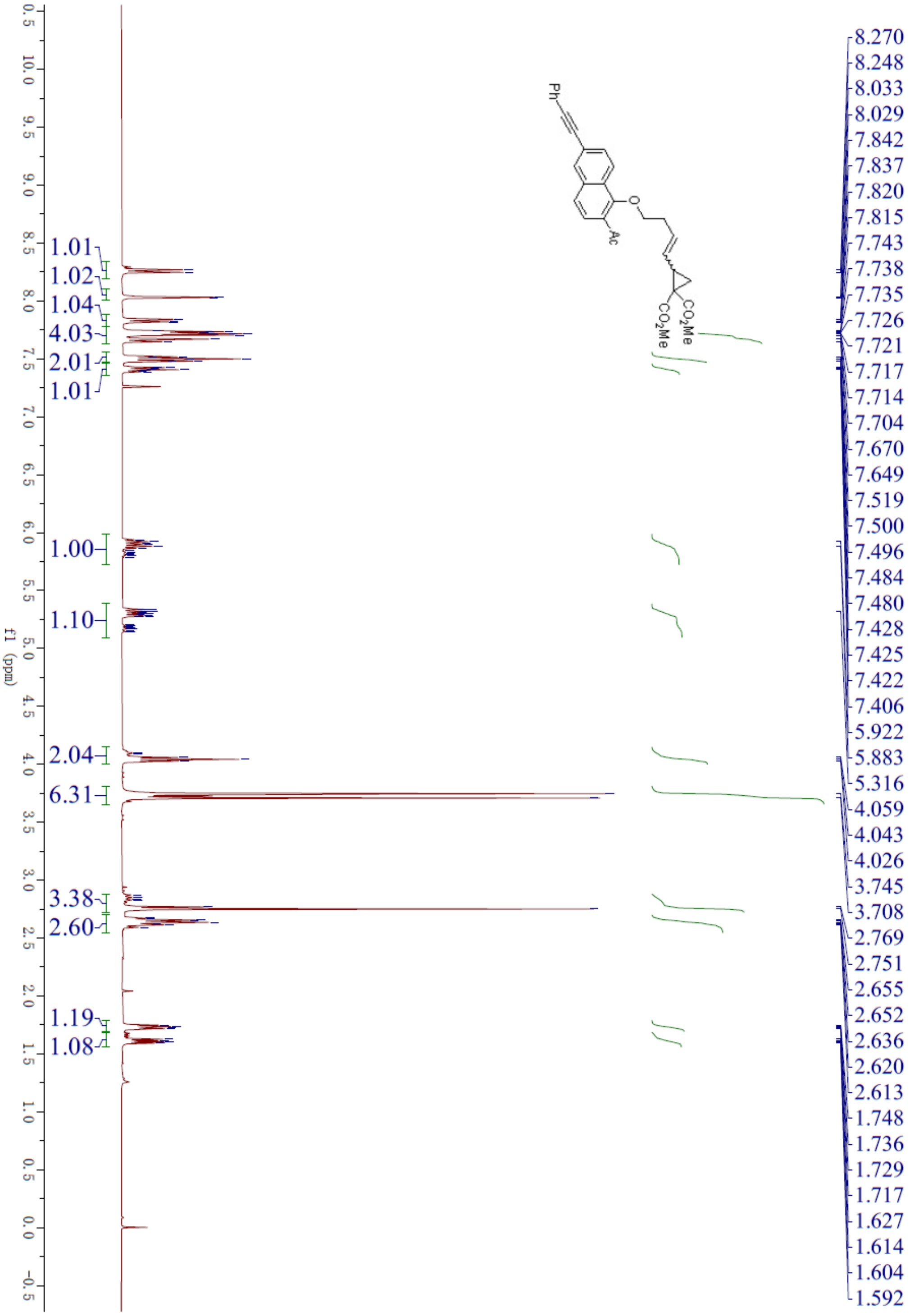

^{13}C NMR Spectrum (100 MHz, $CDCl_3$) of **1r**

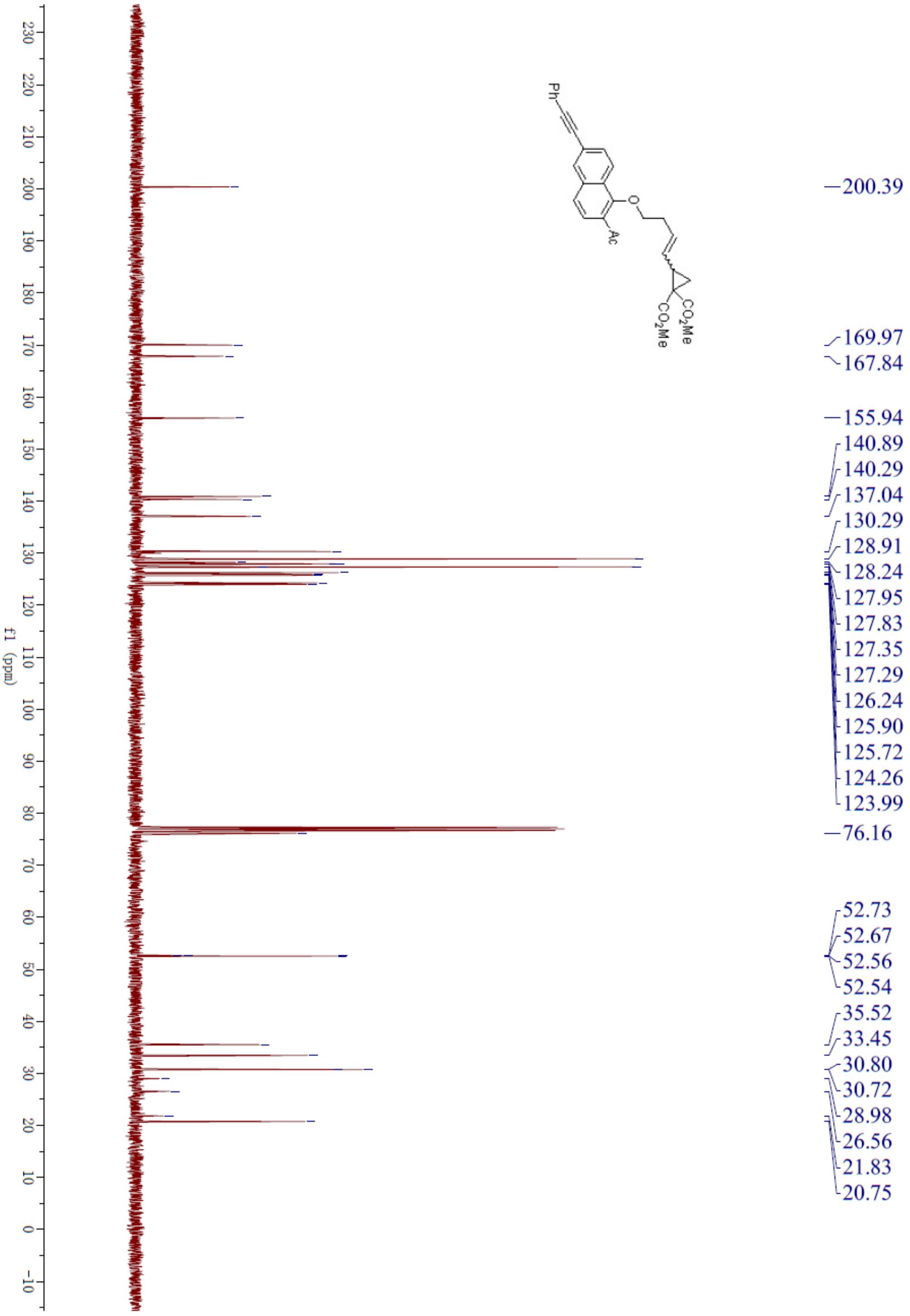

^{1}H NMR Spectrum (400 MHz, $CDCl_3$) of **1s**

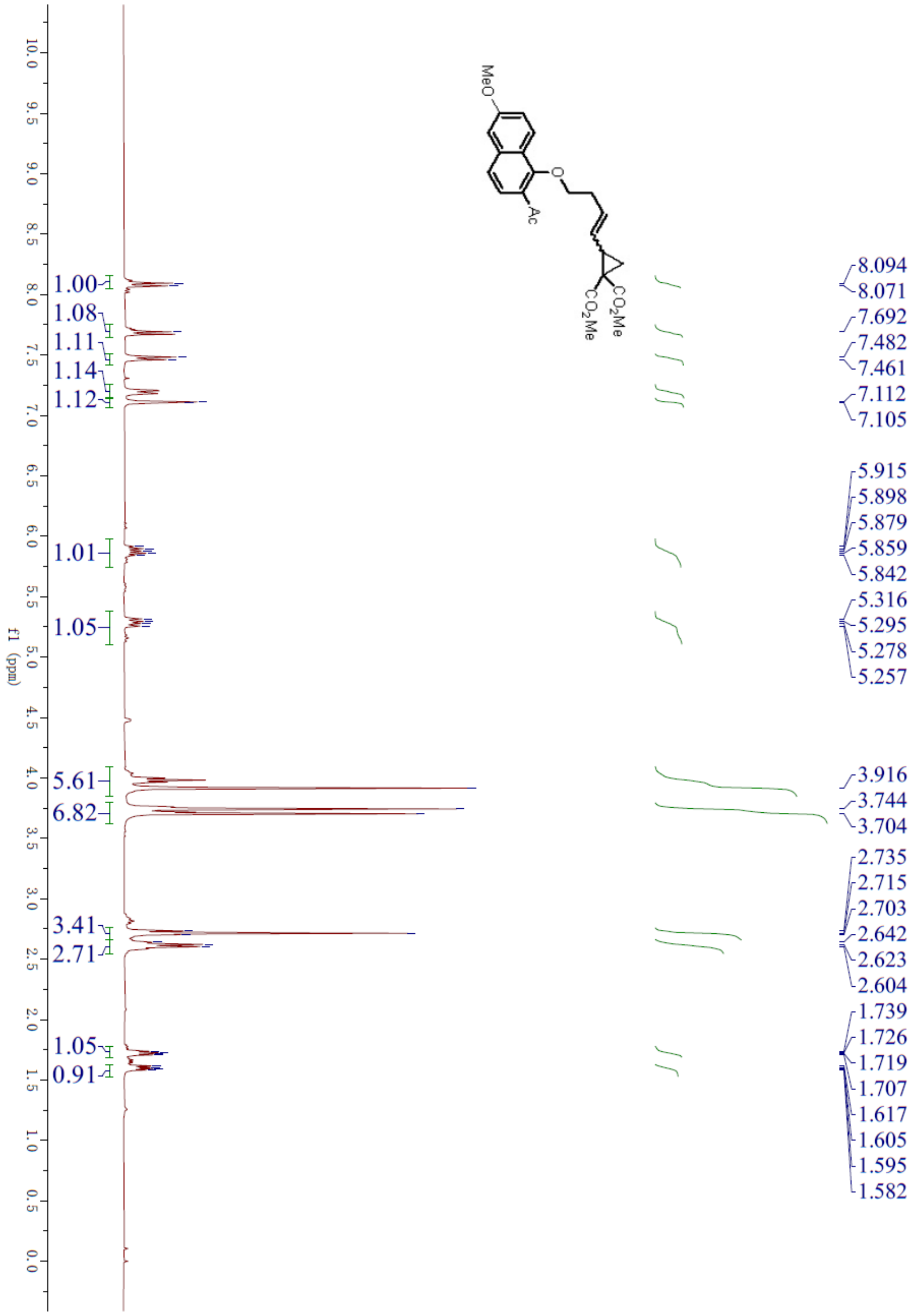

^{13}C NMR Spectrum (100 MHz, $CDCl_3$) of **1s**

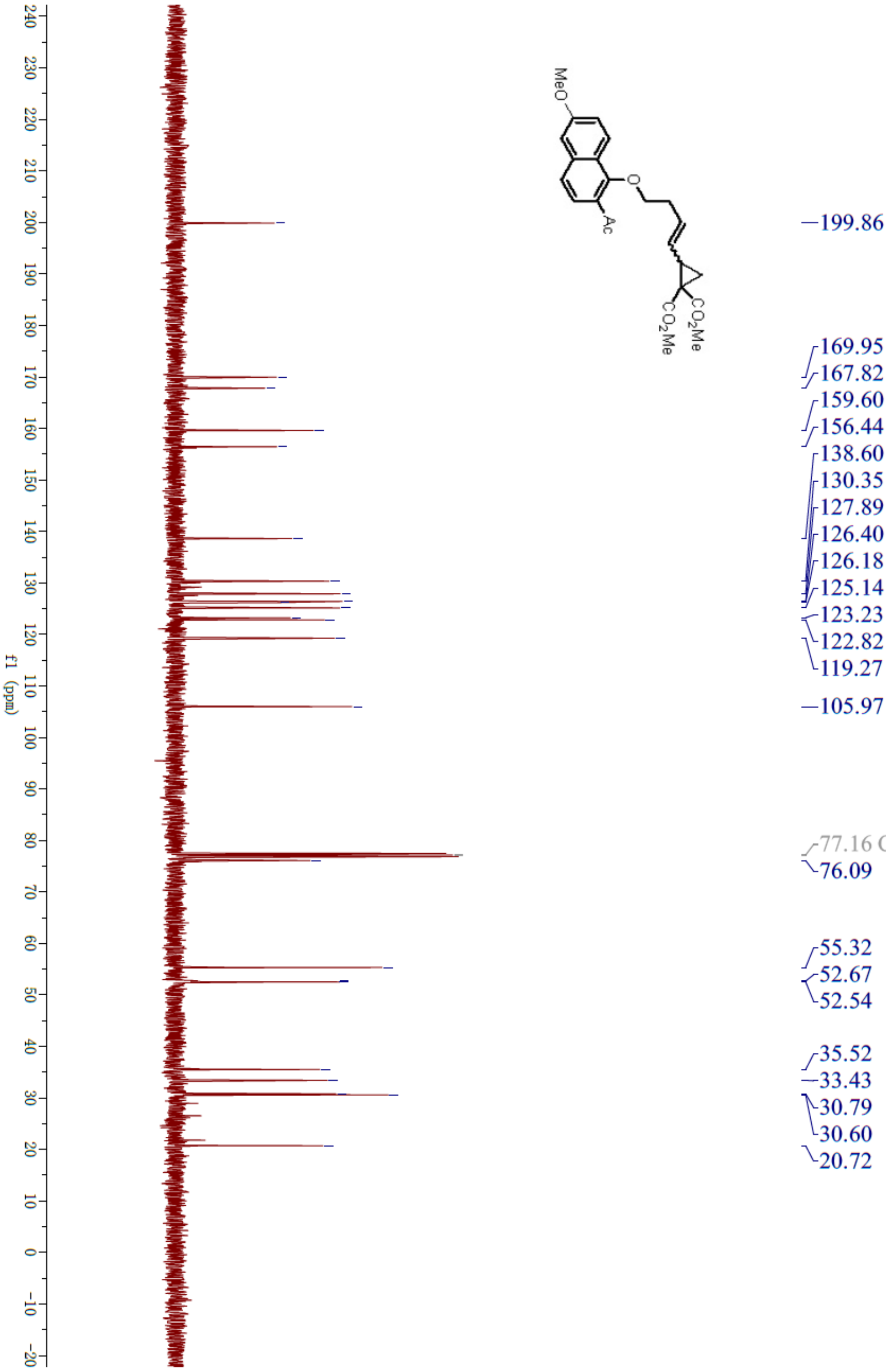

^{1}H NMR Spectrum (400 MHz, CDCl3) of **1t**

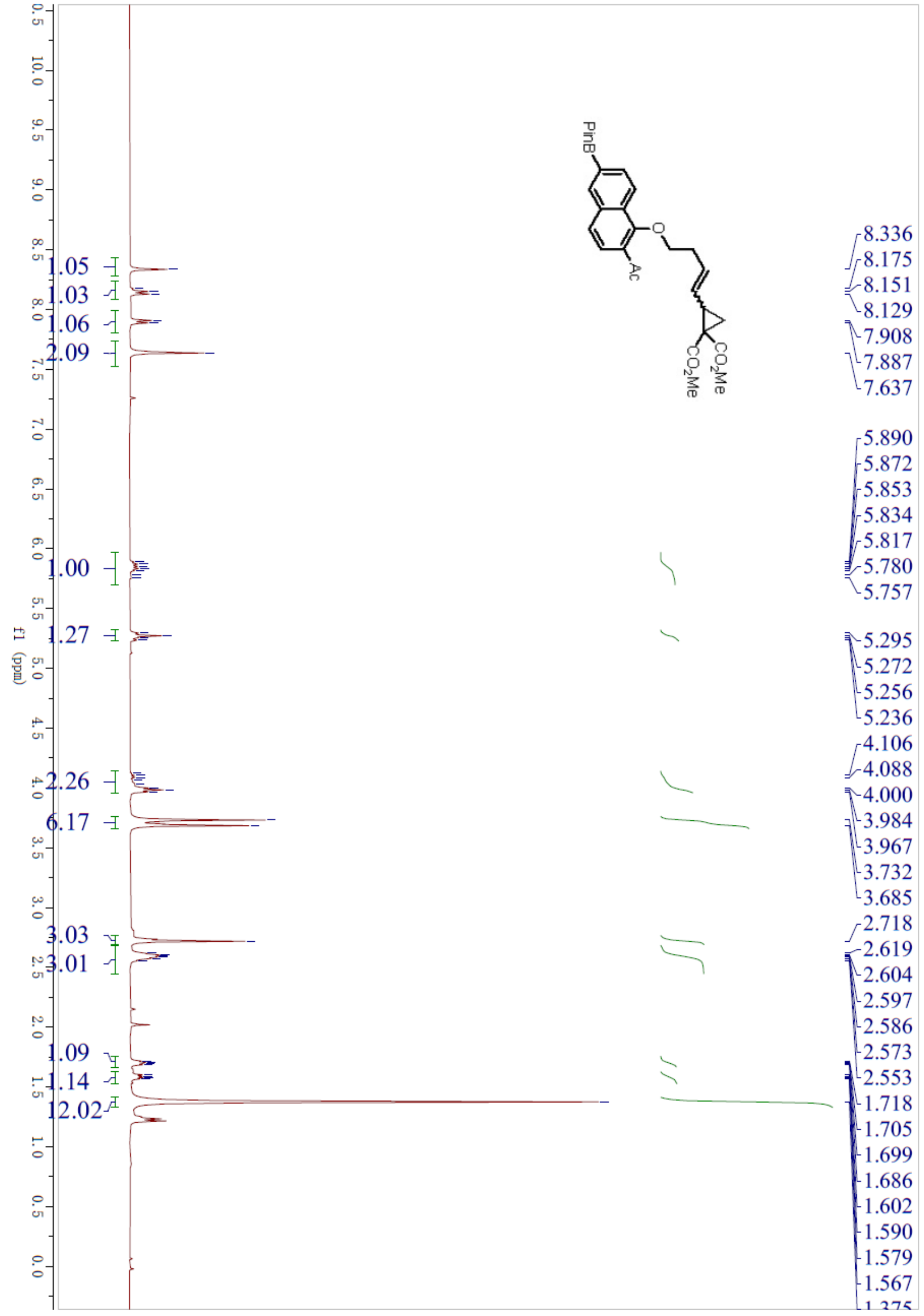

^{13}C NMR Spectrum (100 MHz, $CDCl_3$) of **1t**

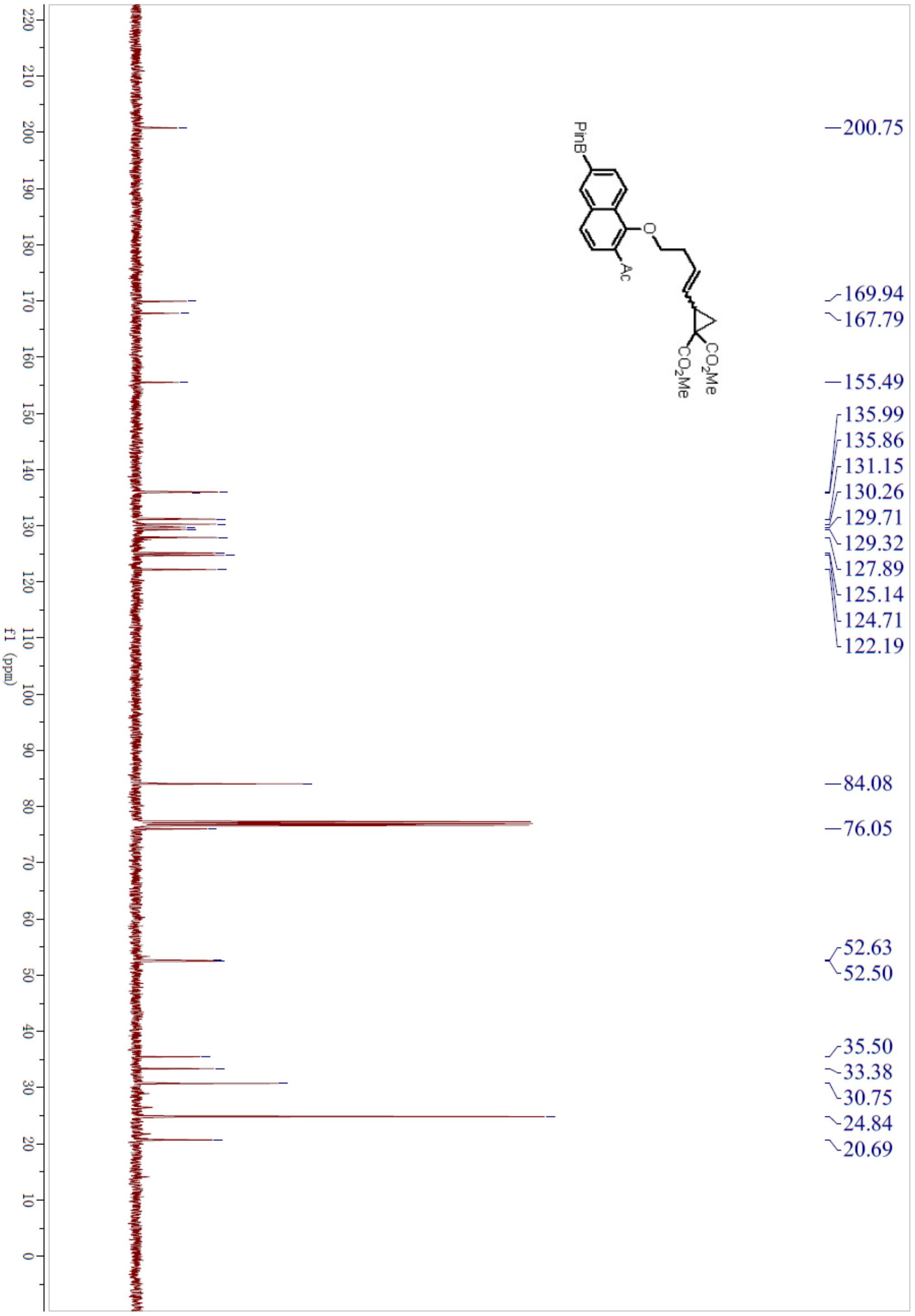

^{1}H NMR Spectrum (400 MHz, CDCl3) of **9a**

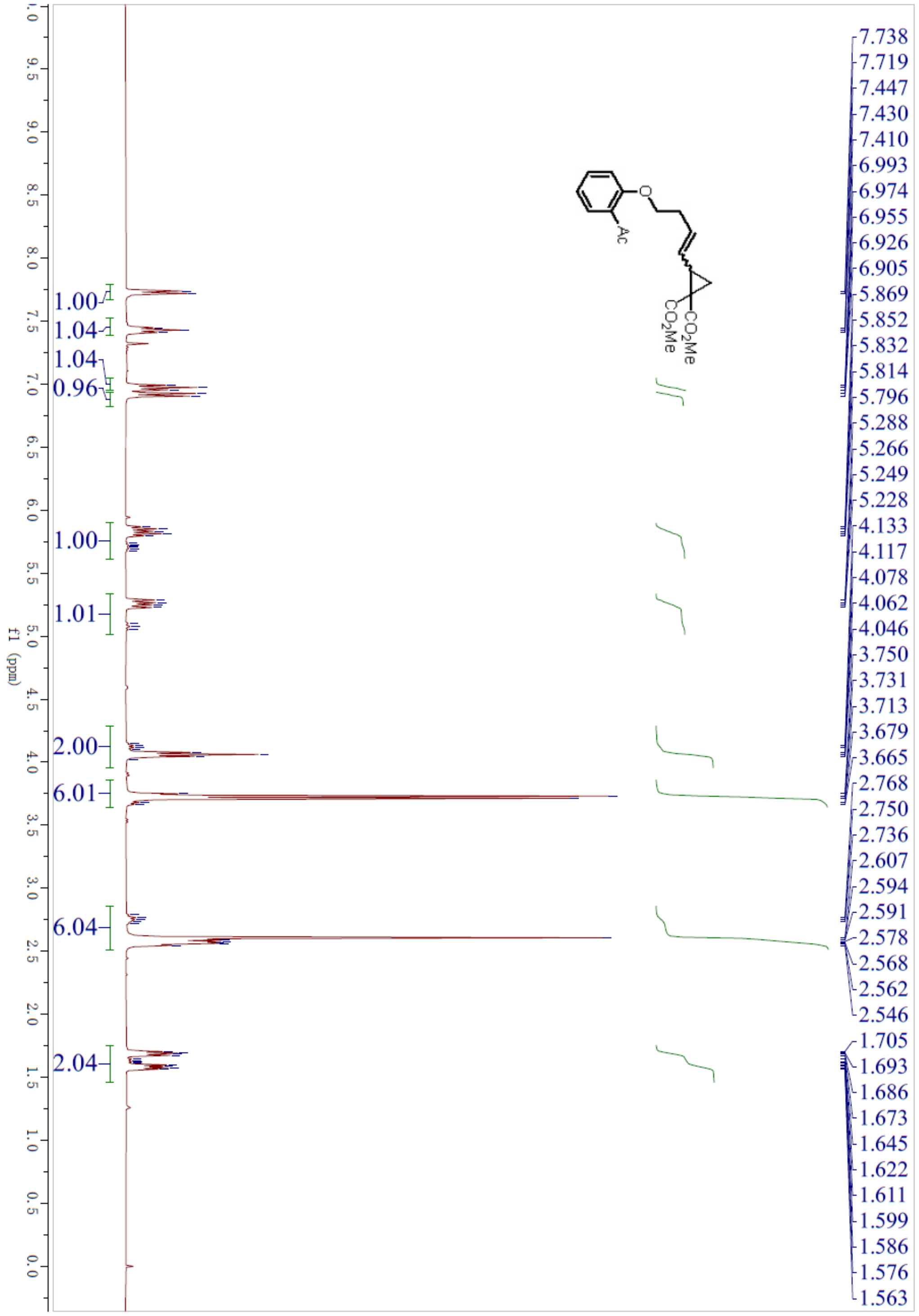

^{13}C NMR Spectrum (100 MHz, $CDCl_3$) of **9a**

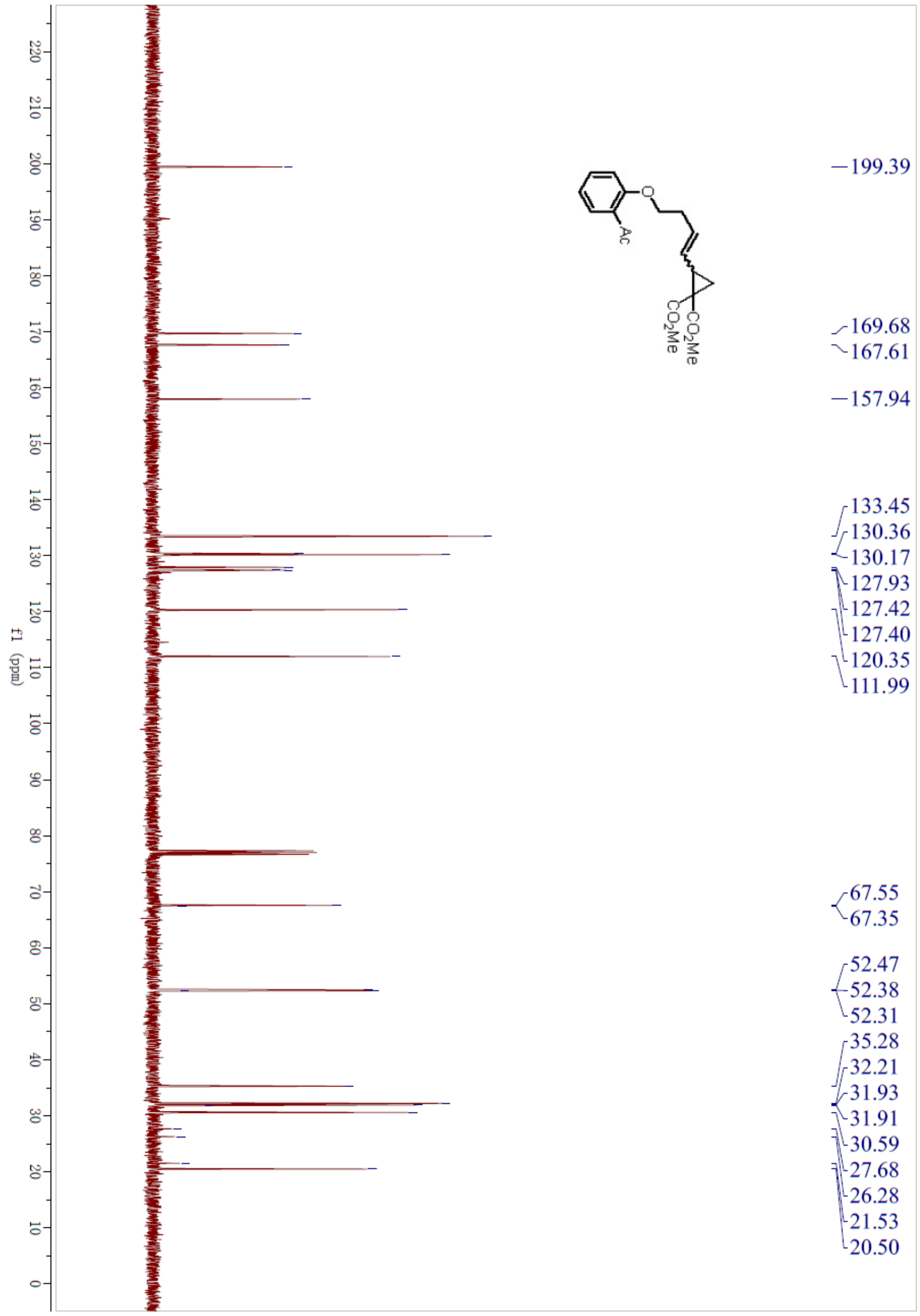

^{1}H NMR Spectrum (400 MHz, $CDCl_3$) of **9b**

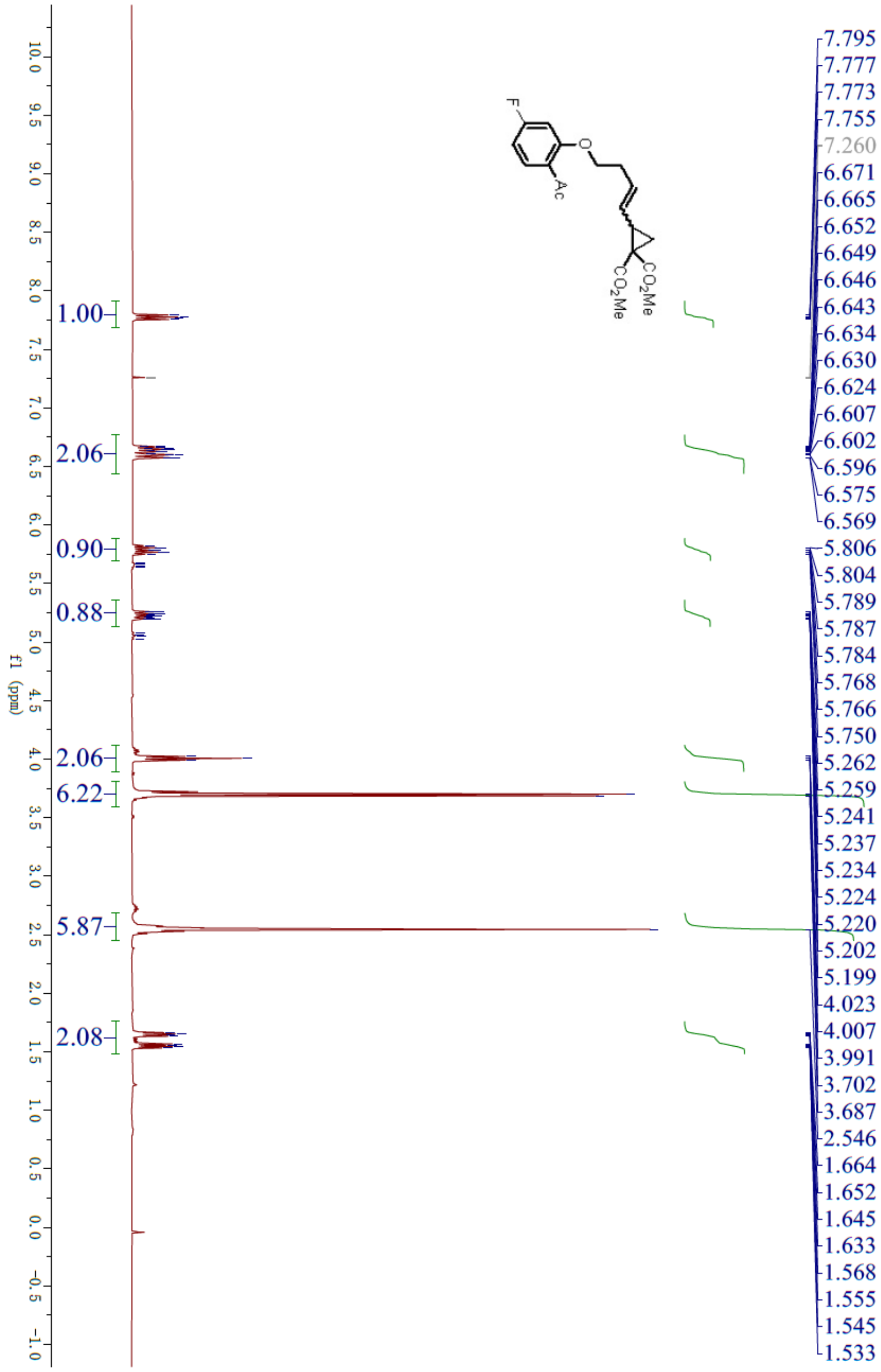

^{13}C NMR Spectrum (100 MHz, $CDCl_3$) of **9b**

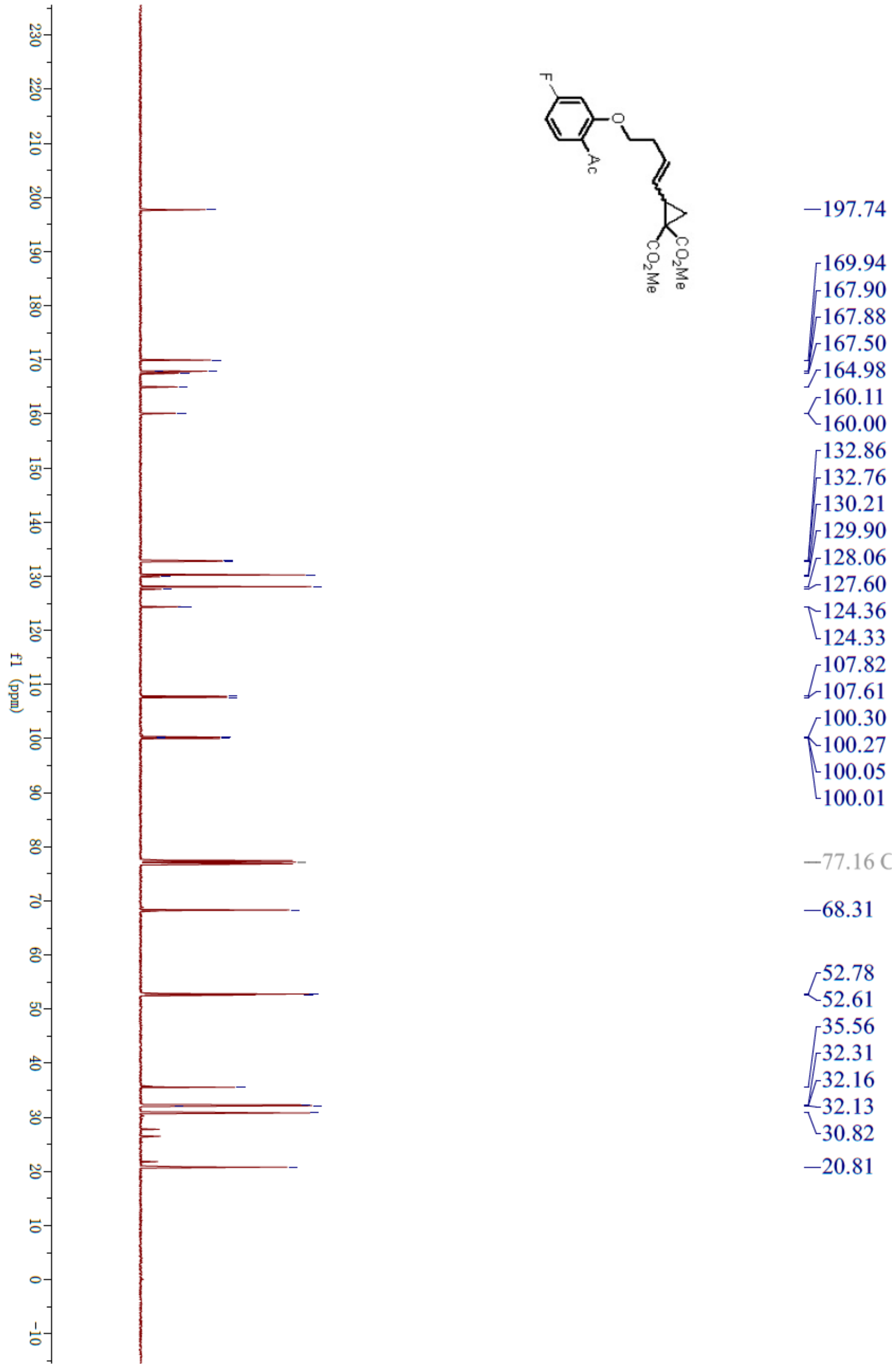

^{19}F NMR Spectrum (376 MHz, $CDCl_3$) of **9b**

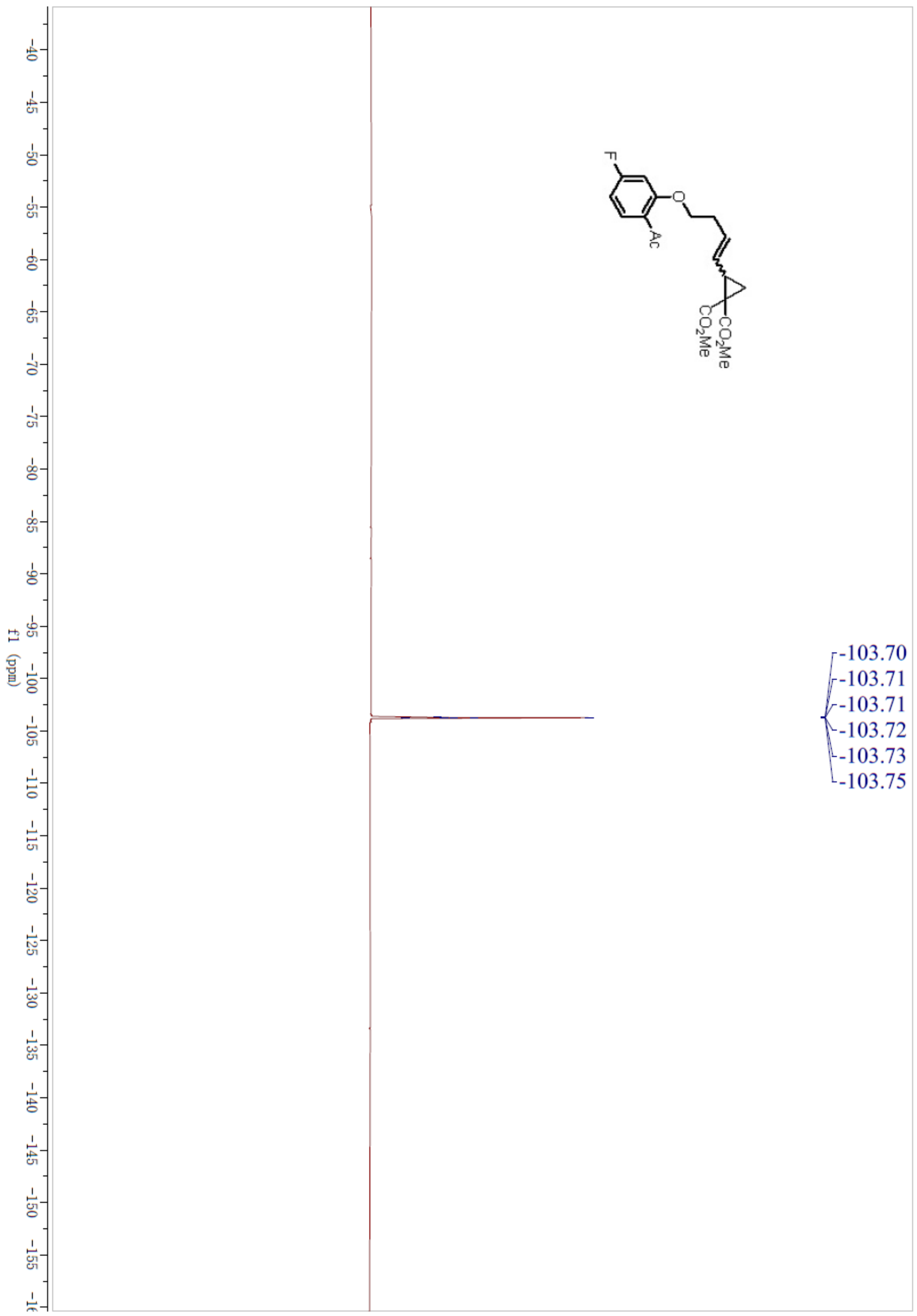

^{1}H NMR Spectrum (400 MHz, $CDCl_3$) of *E*-**2a**

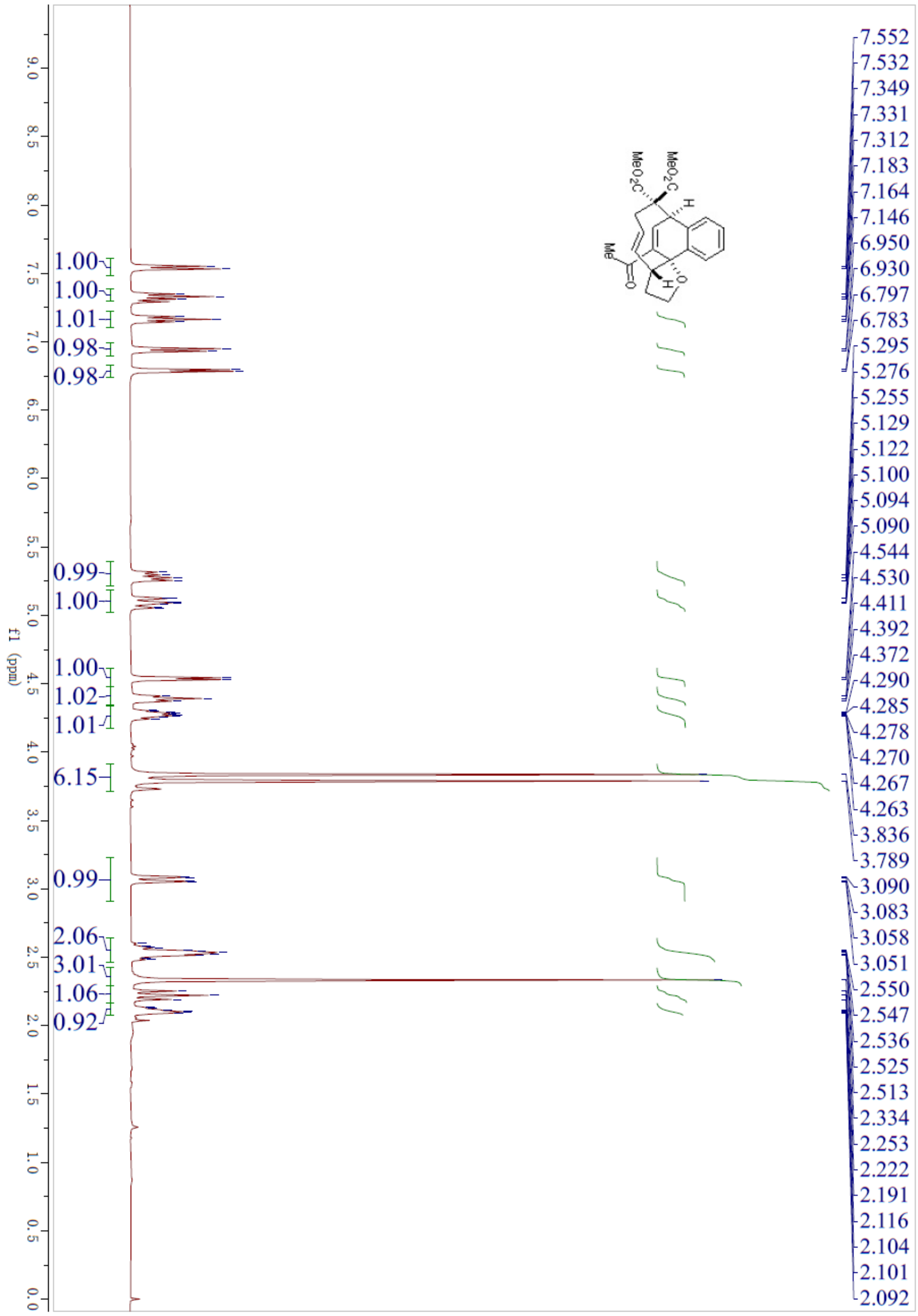

^{13}C NMR Spectrum (100 MHz, $CDCl_3$) of *E*-**2a**

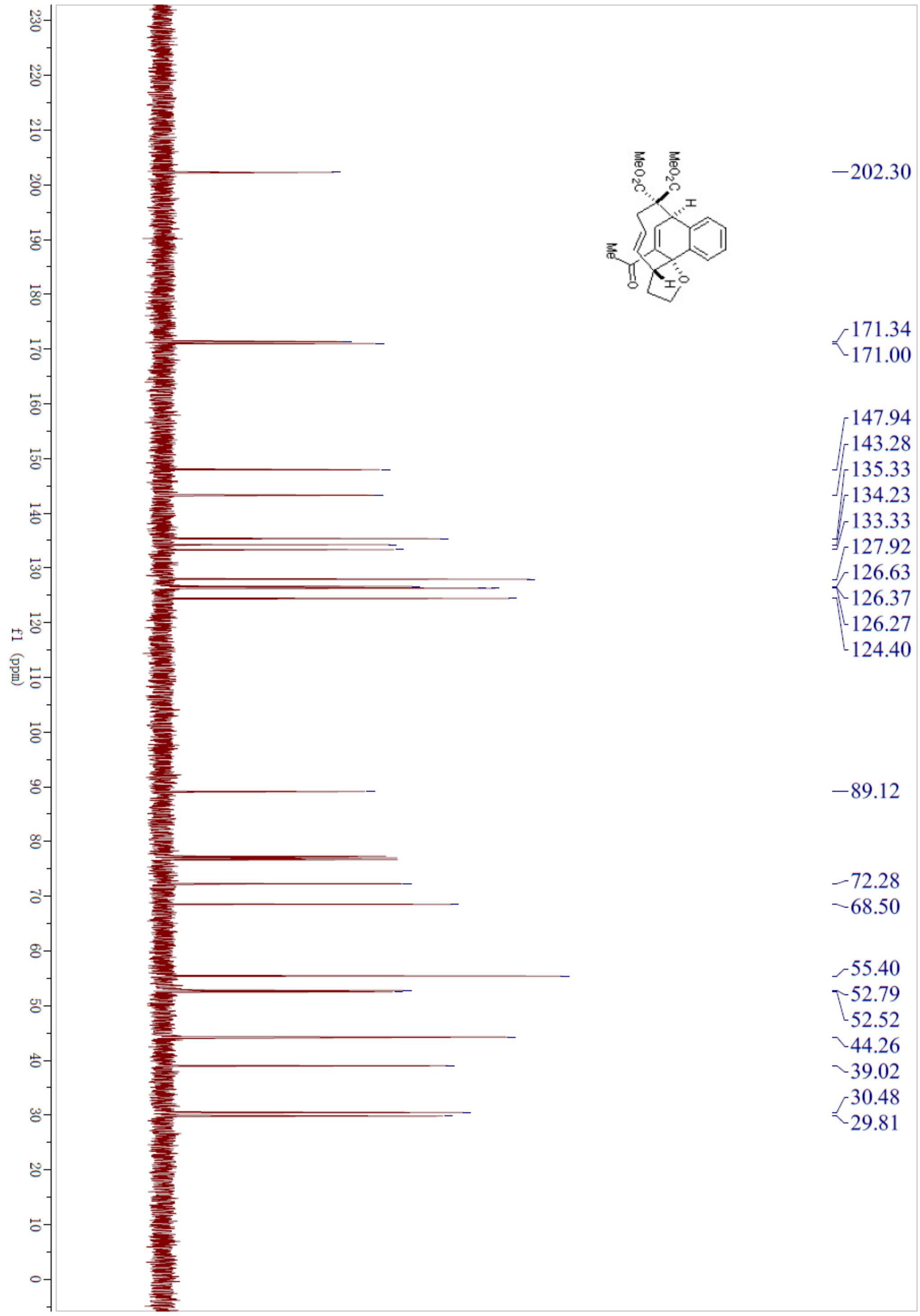

^{1}H NMR Spectrum (400 MHz, CDCl3) of *Z*-**2a**

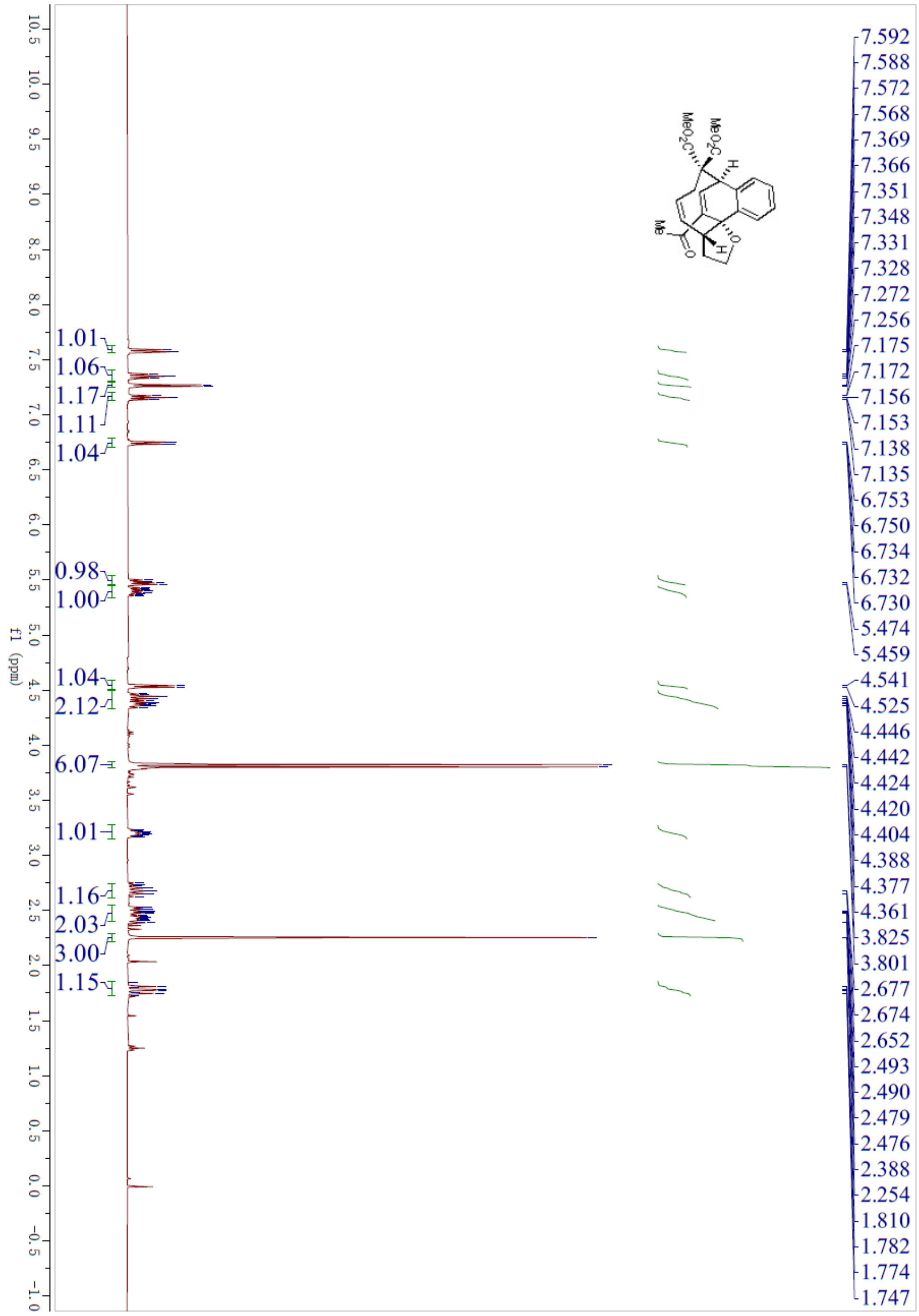

^{13}C NMR Spectrum (100 MHz, $CDCl_3$) of *Z*-**2a**

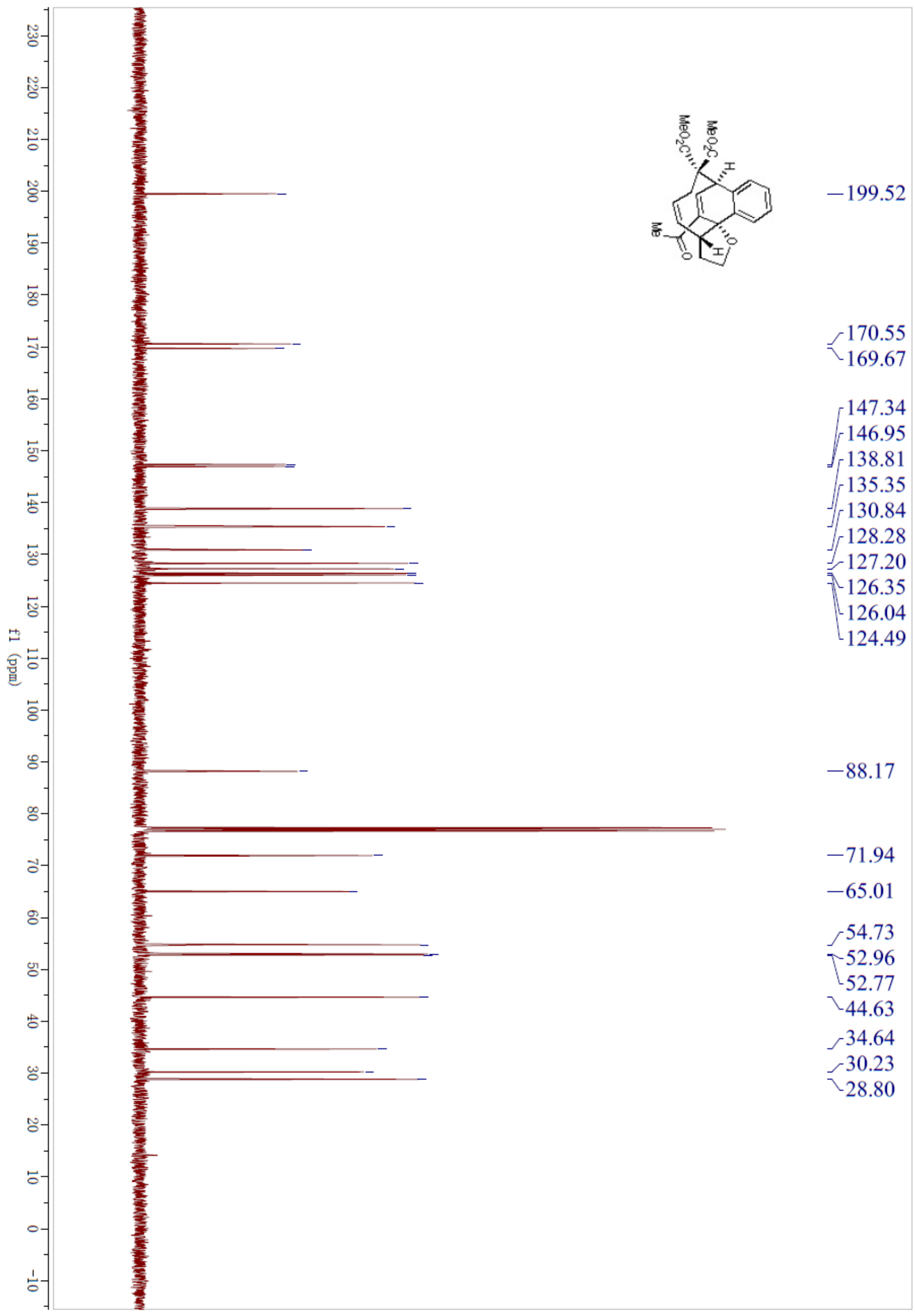

^{1}H NMR Spectrum (400 MHz, $CDCl_3$) of *E*-**2b**

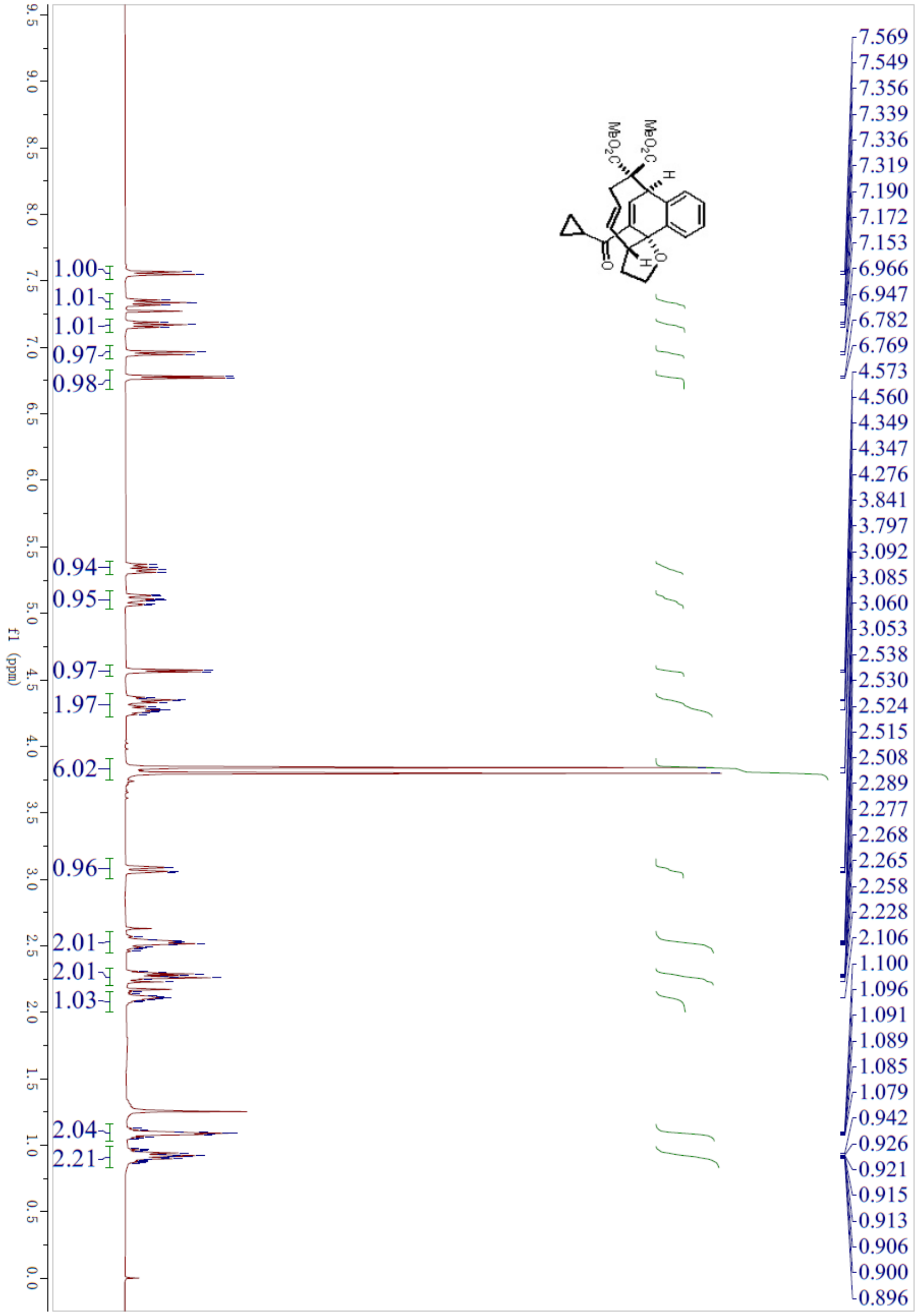

^{13}C NMR Spectrum (100 MHz, $CDCl_3$) of *E*-**2b**

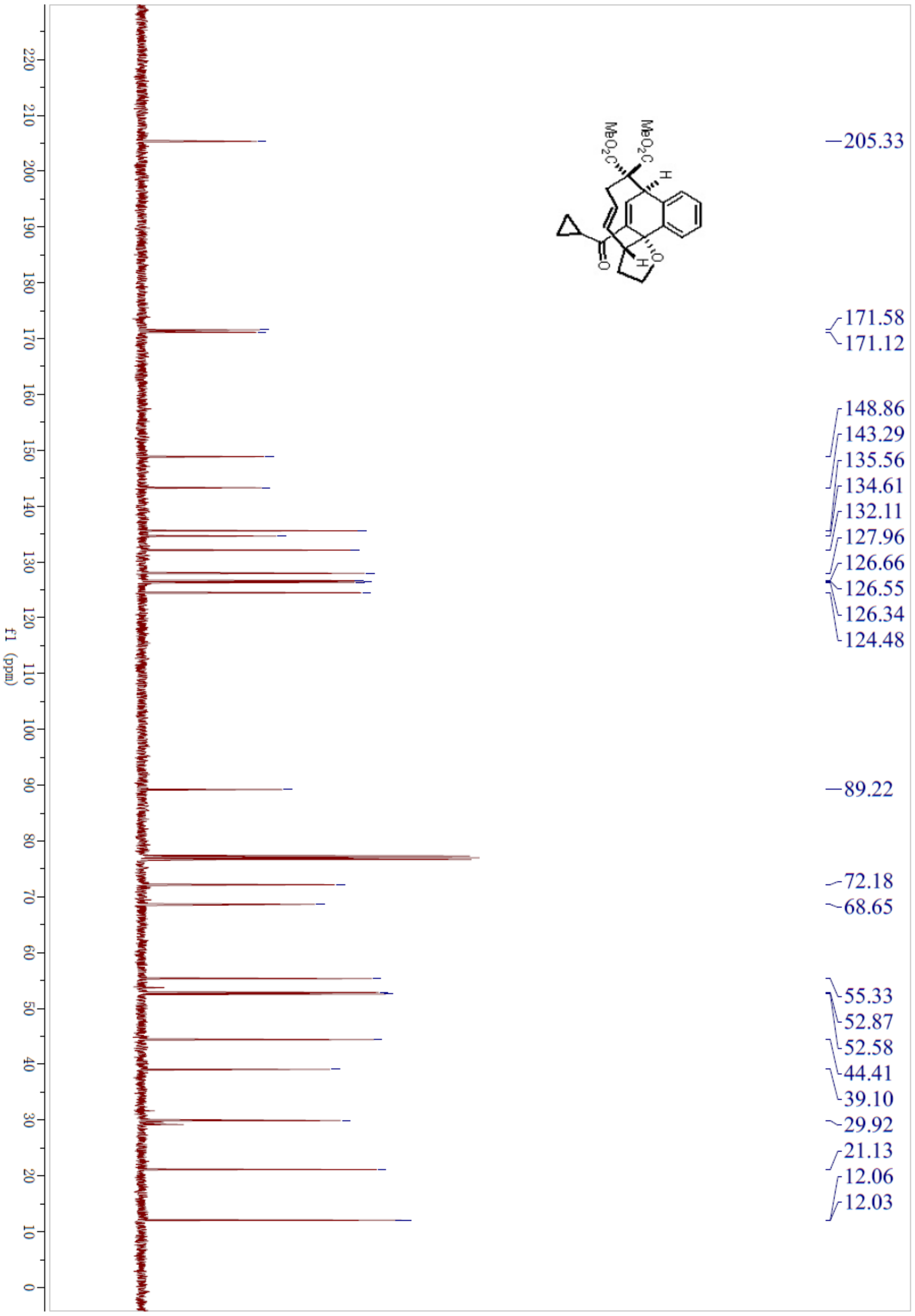

^{1}H NMR Spectrum (400 MHz, $CDCl_3$) of *E*-**2c**

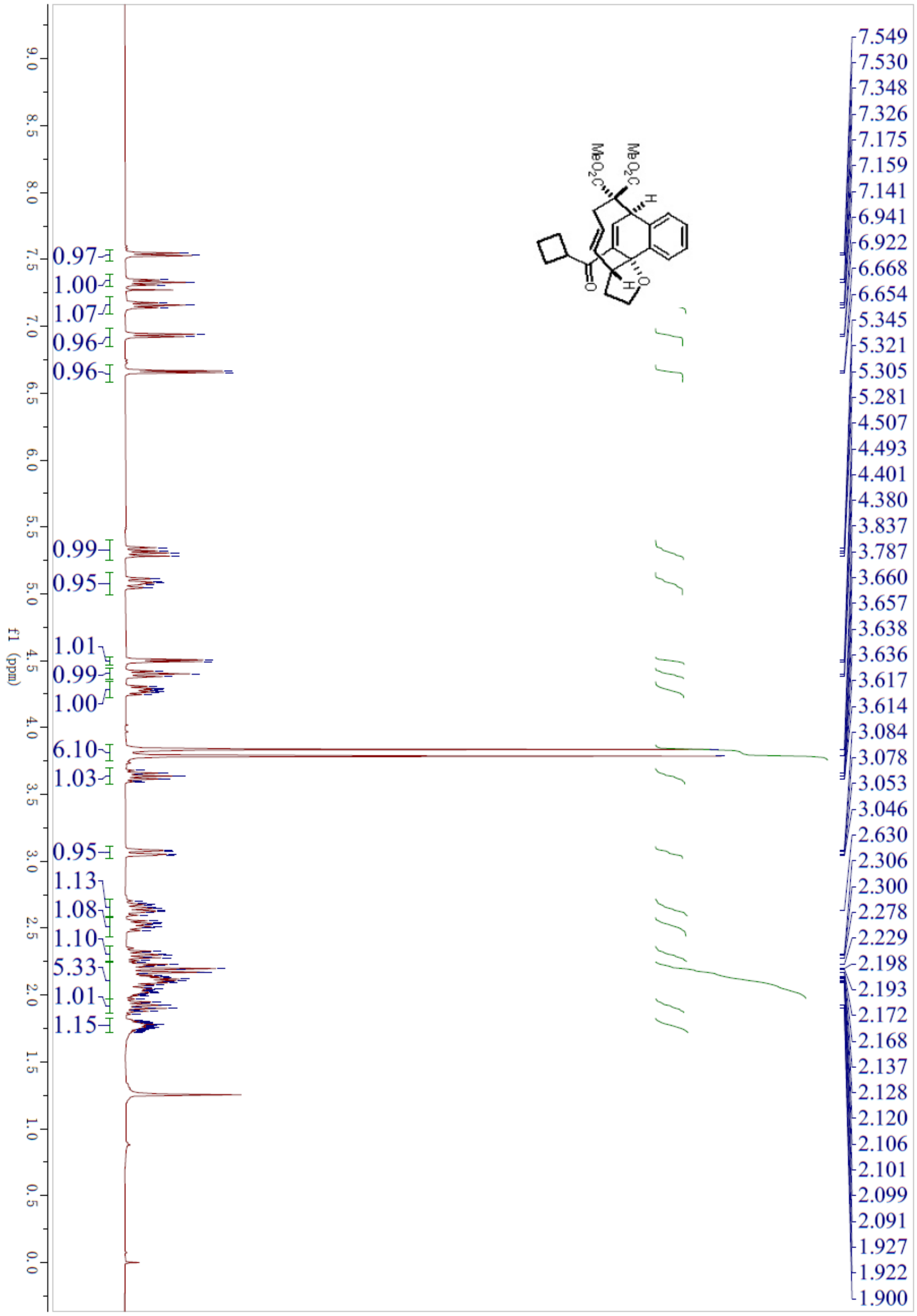

^{13}C NMR Spectrum (100 MHz, $CDCl_3$) of *E*-**2c**

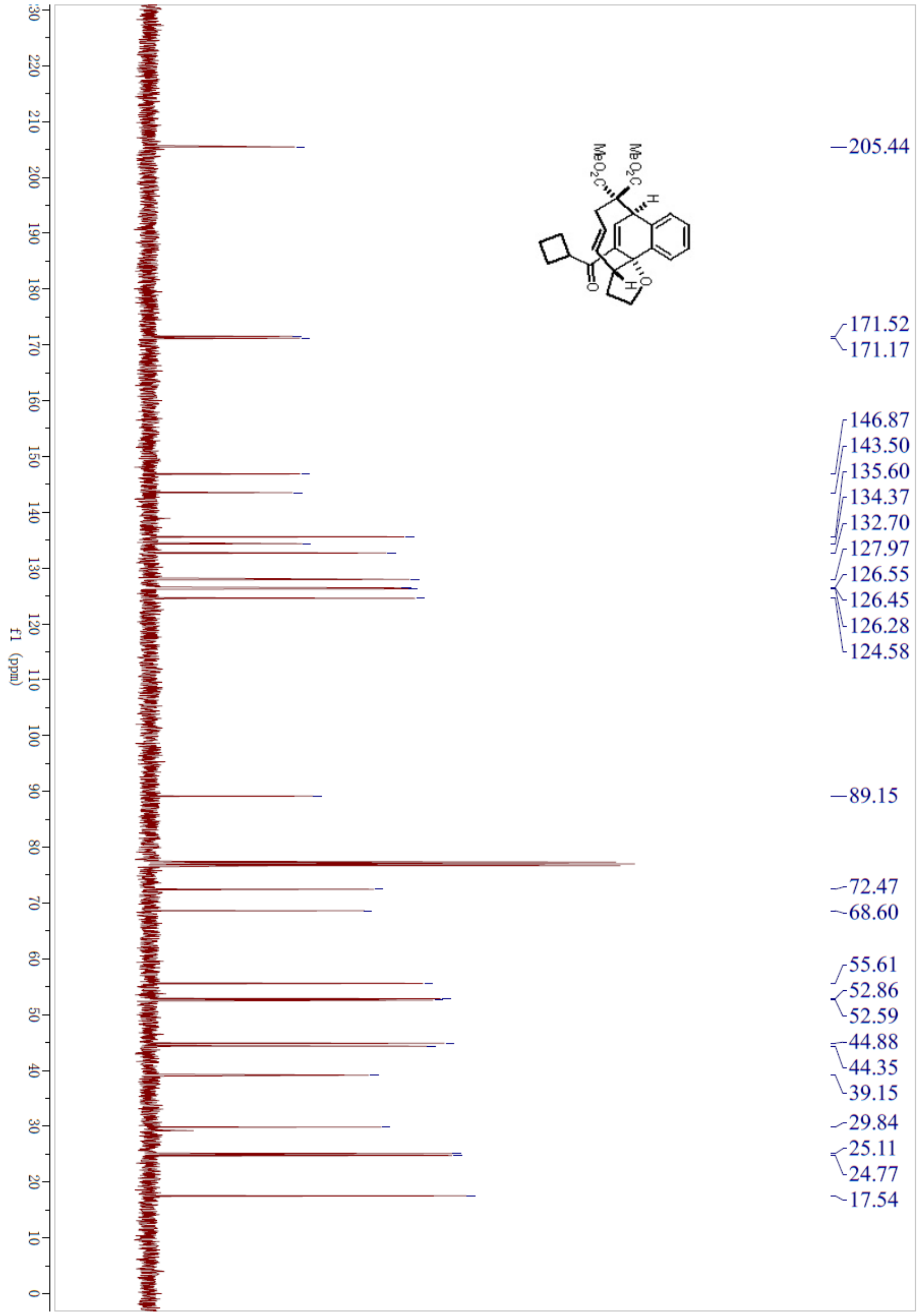

^{1}H NMR Spectrum (400 MHz, $CDCl_3$) of *E*-**2d**

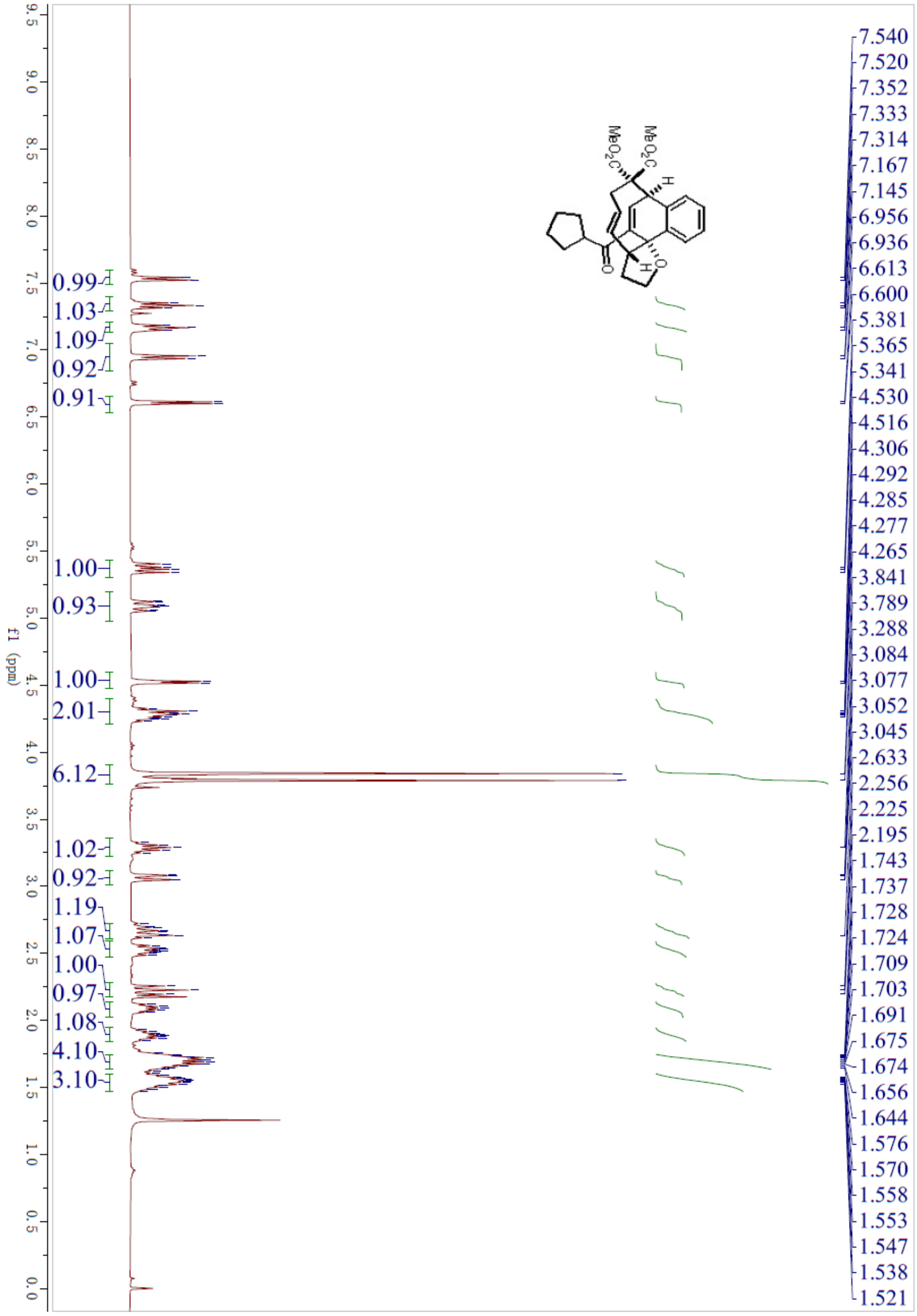

^{13}C NMR Spectrum (100 MHz, $CDCl_3$) of *E*-**2d**

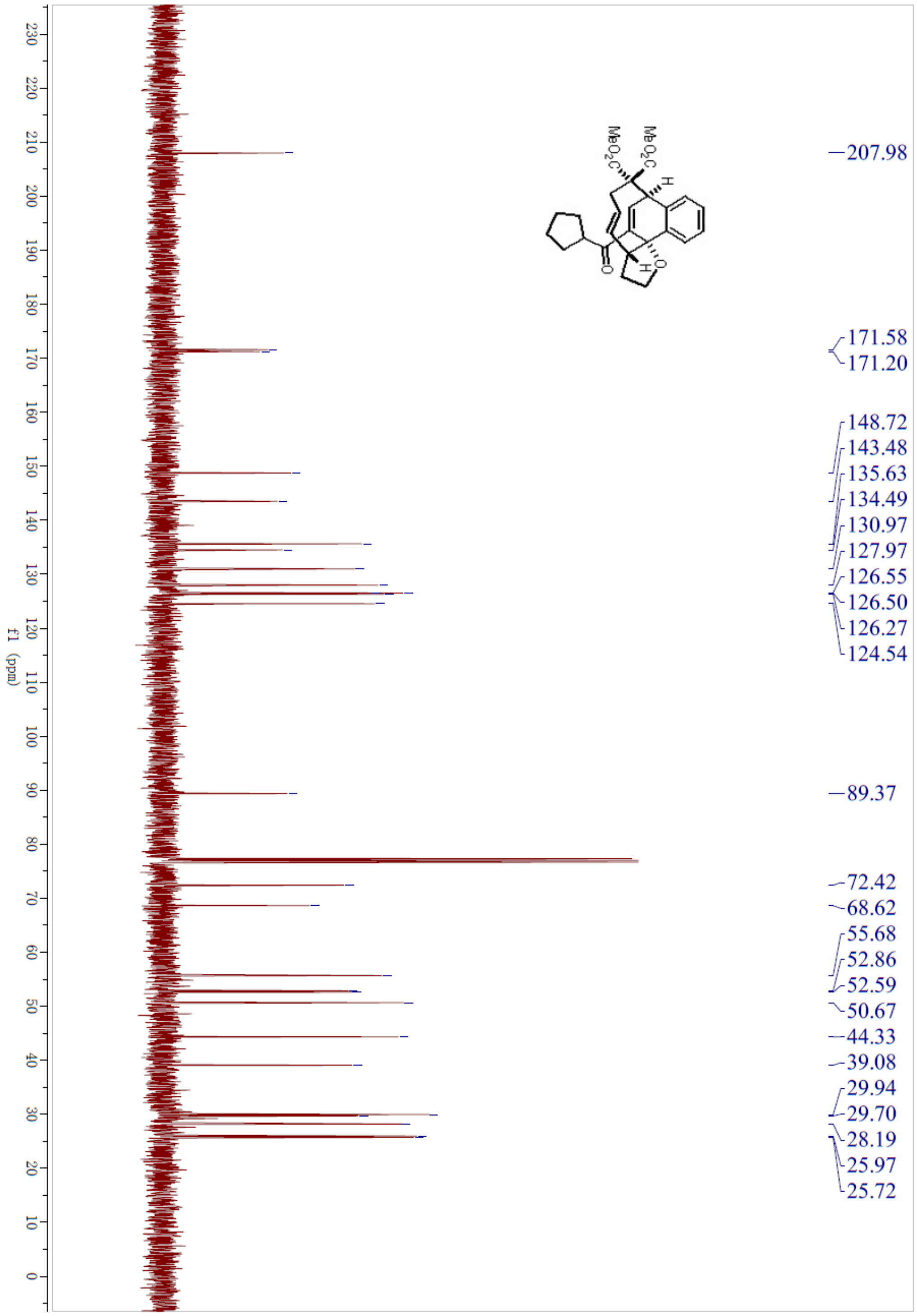

^{1}H NMR Spectrum (400 MHz, $CDCl_3$) of *E*-**2e**

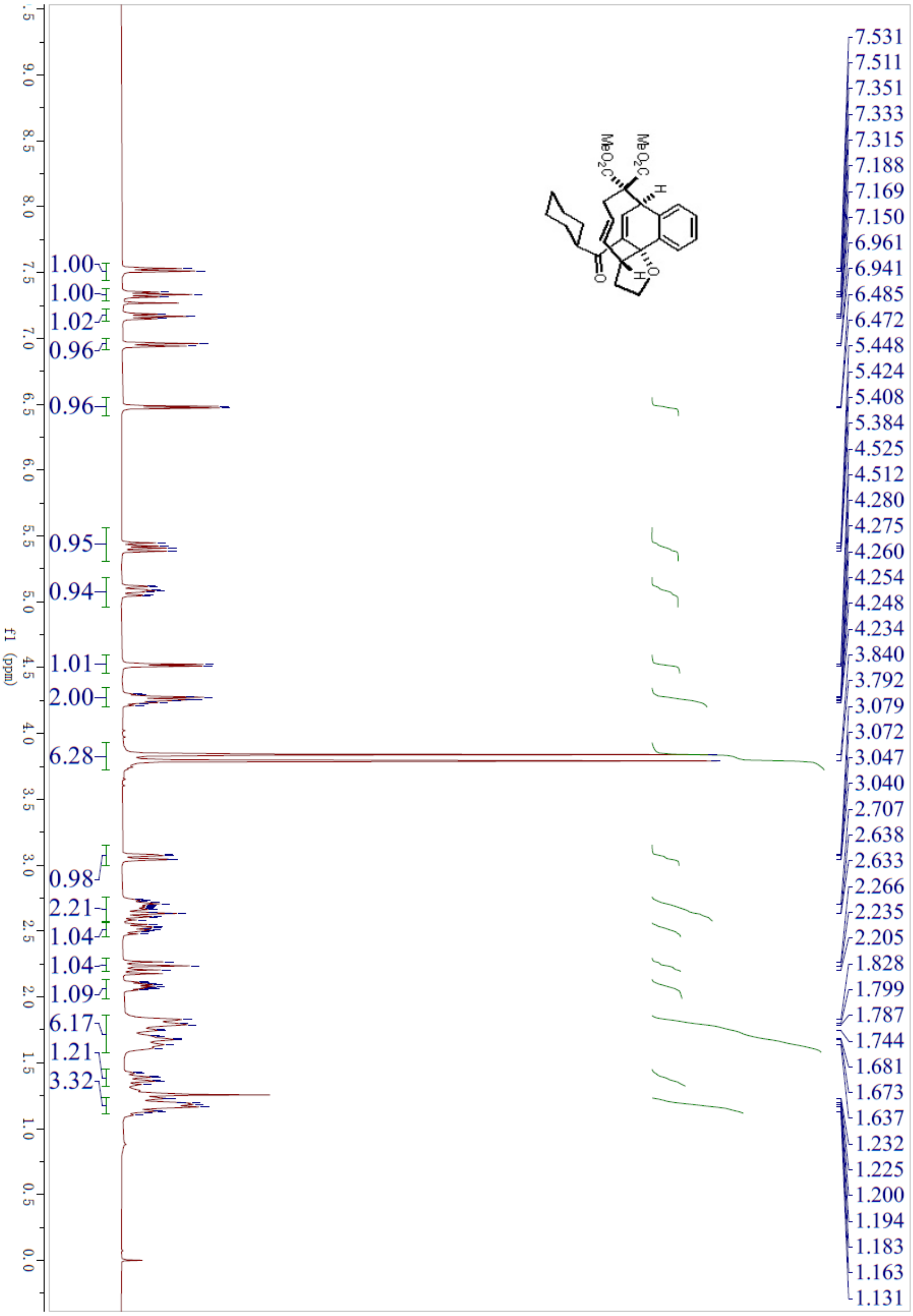

^{13}C NMR Spectrum (100 MHz, $CDCl_3$) of *E*-**2e**

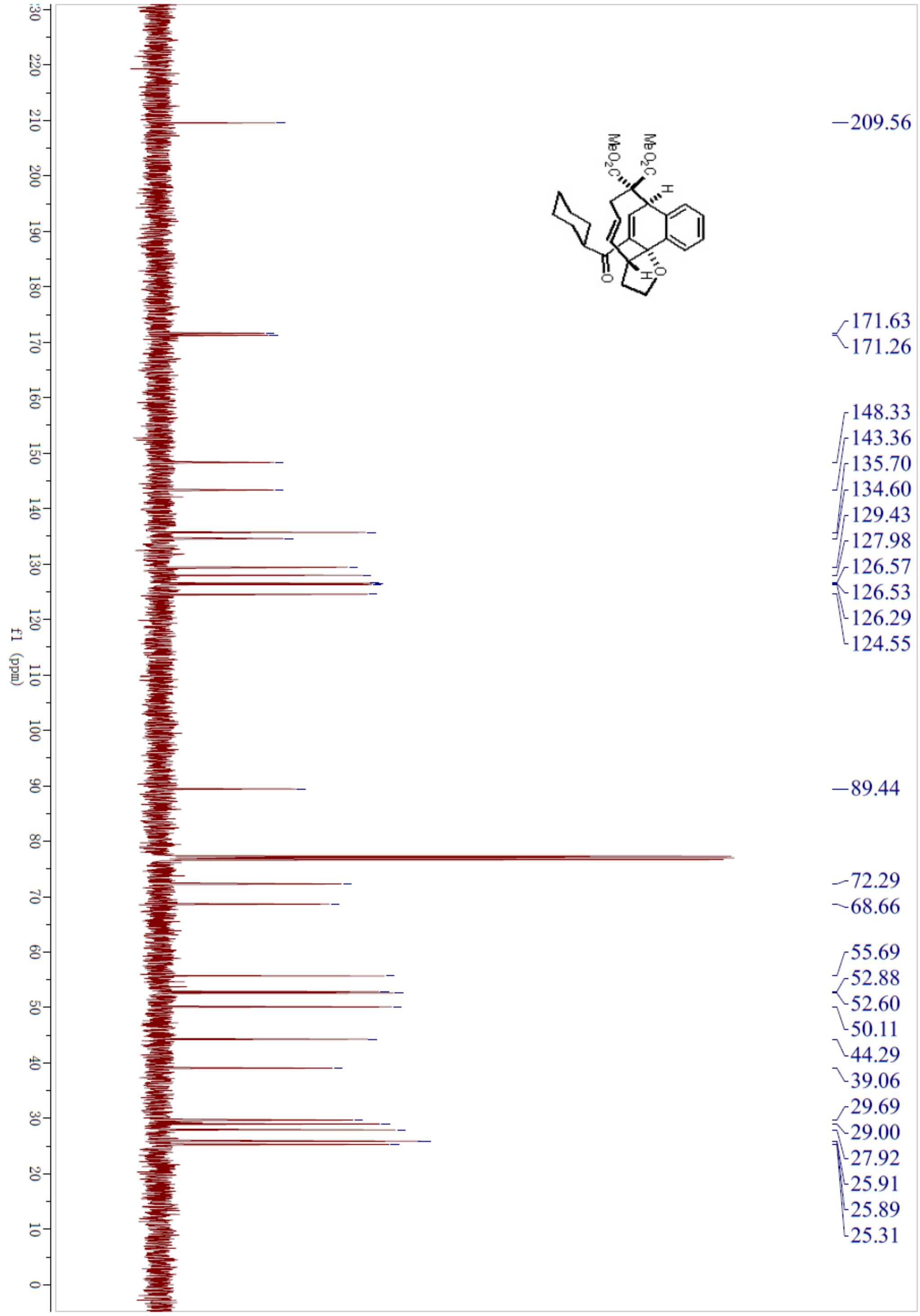

^{1}H NMR Spectrum (400 MHz, $CDCl_3$) of *E*-**2f**

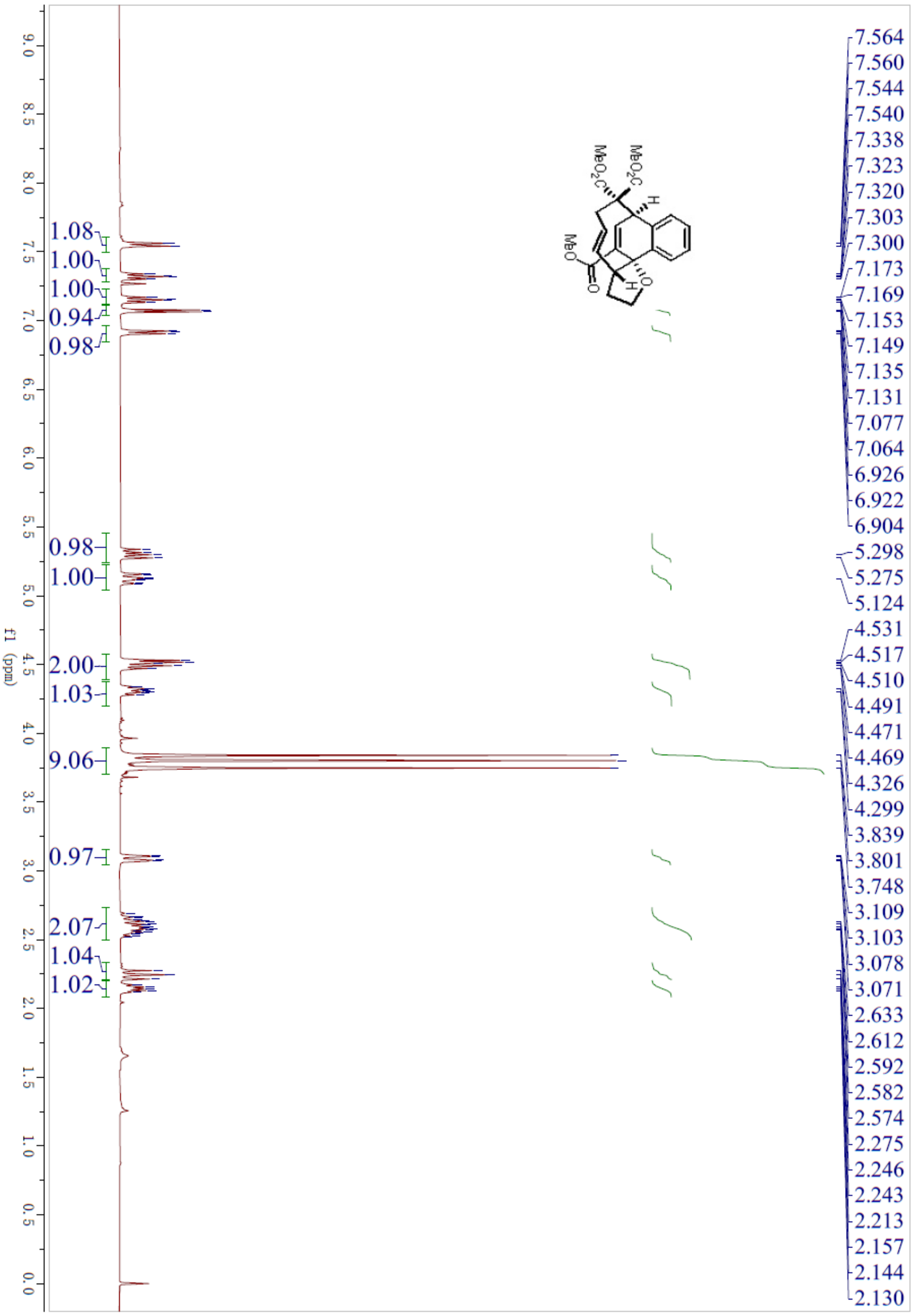

^{13}C NMR Spectrum (100 MHz, $CDCl_3$) of *E*-**2f**

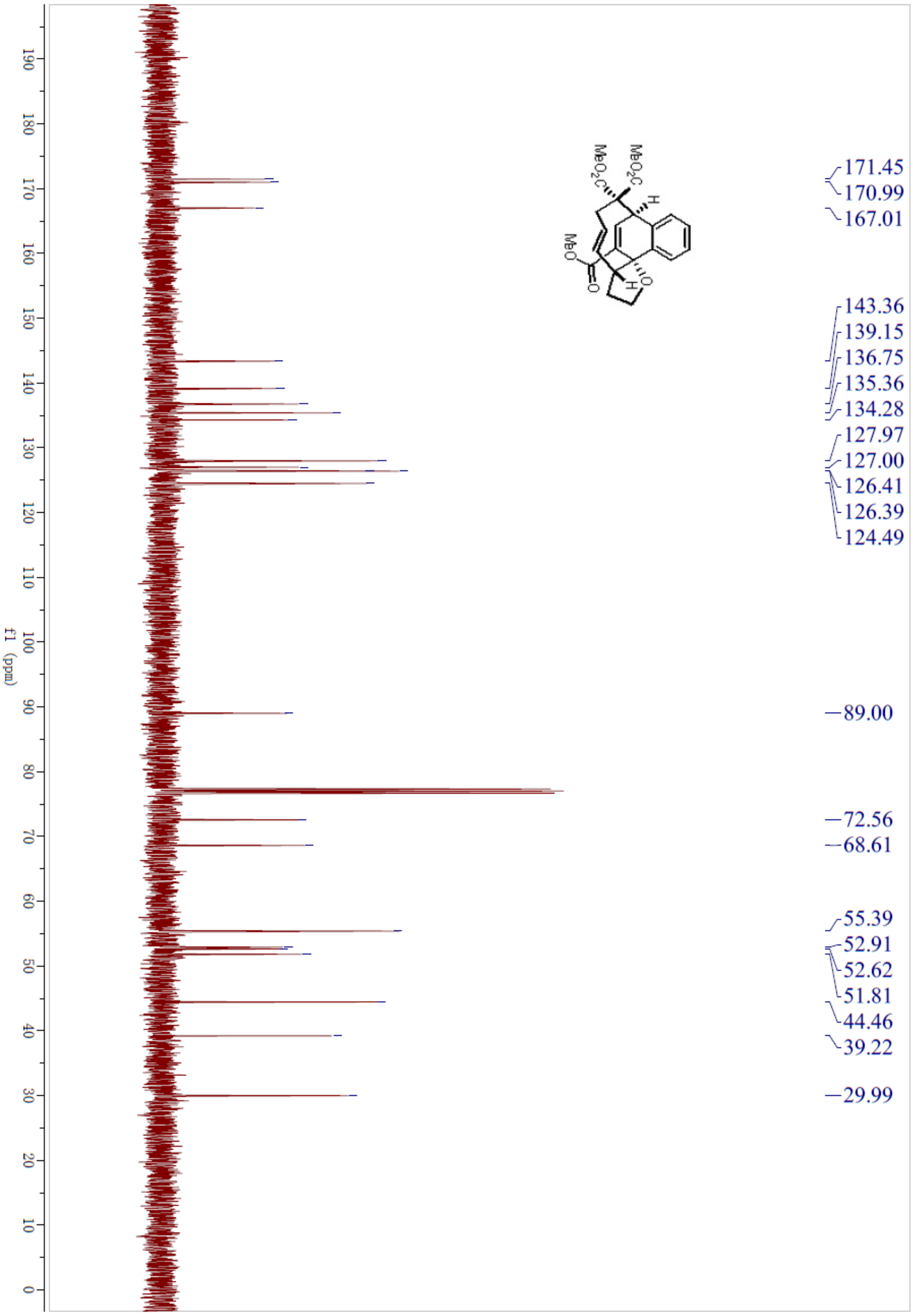

^{1}H NMR Spectrum (400 MHz, CDCl3) of *E*-**2g**

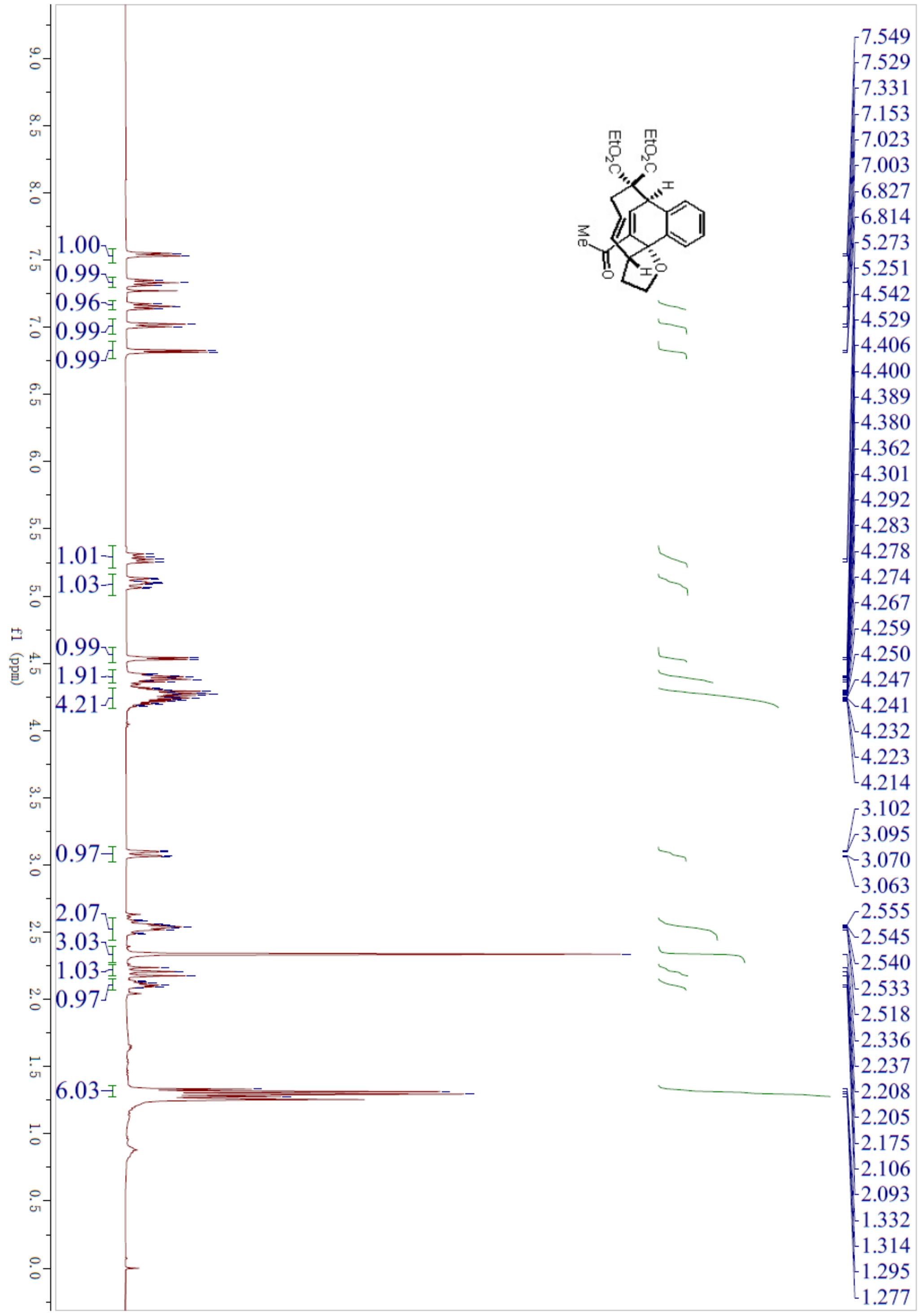

^{13}C NMR Spectrum (100 MHz, CDCl3) of *E*-**2g**

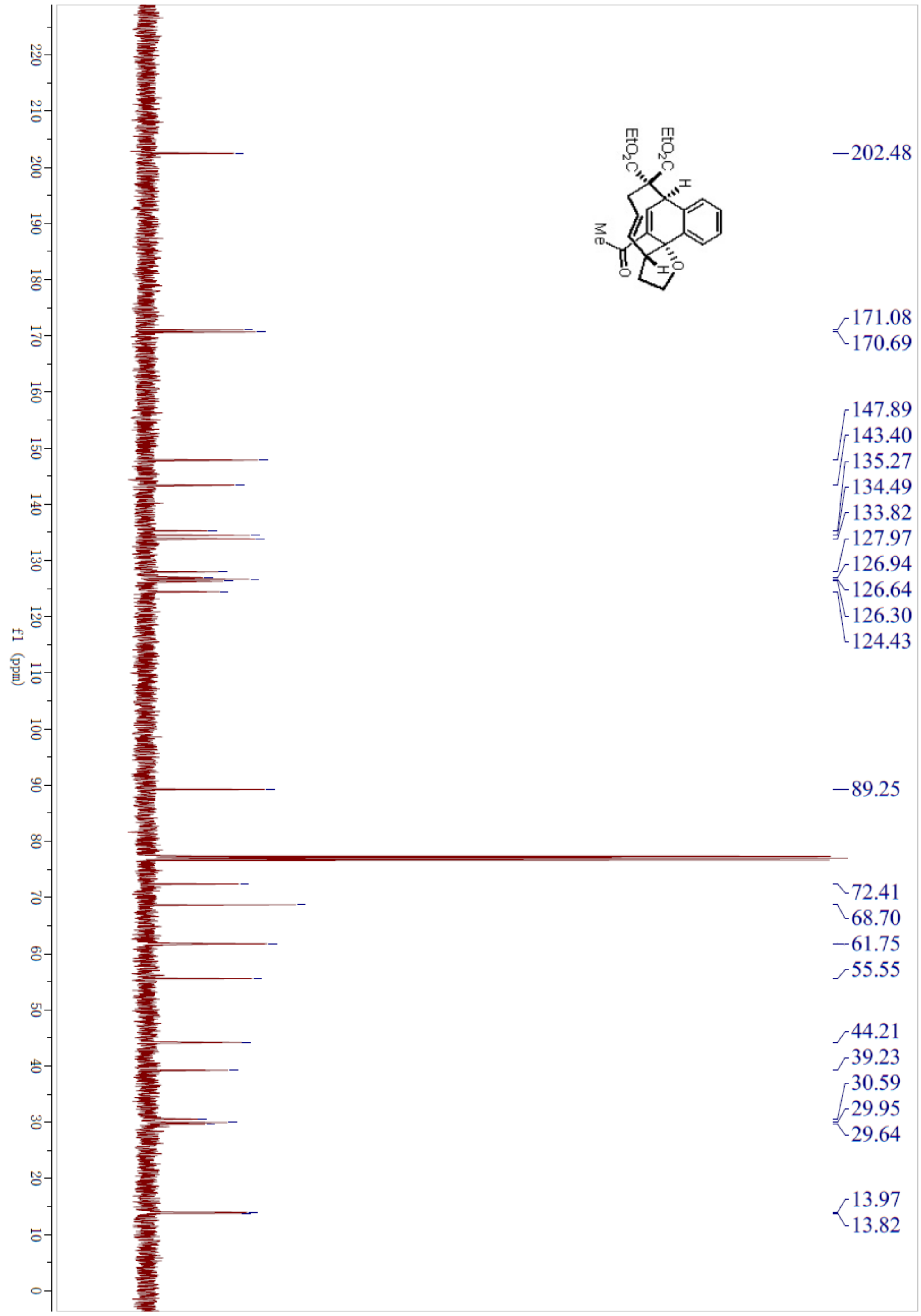

^{1}H NMR Spectrum (400 MHz, CDCl3) of *E*-**2h**

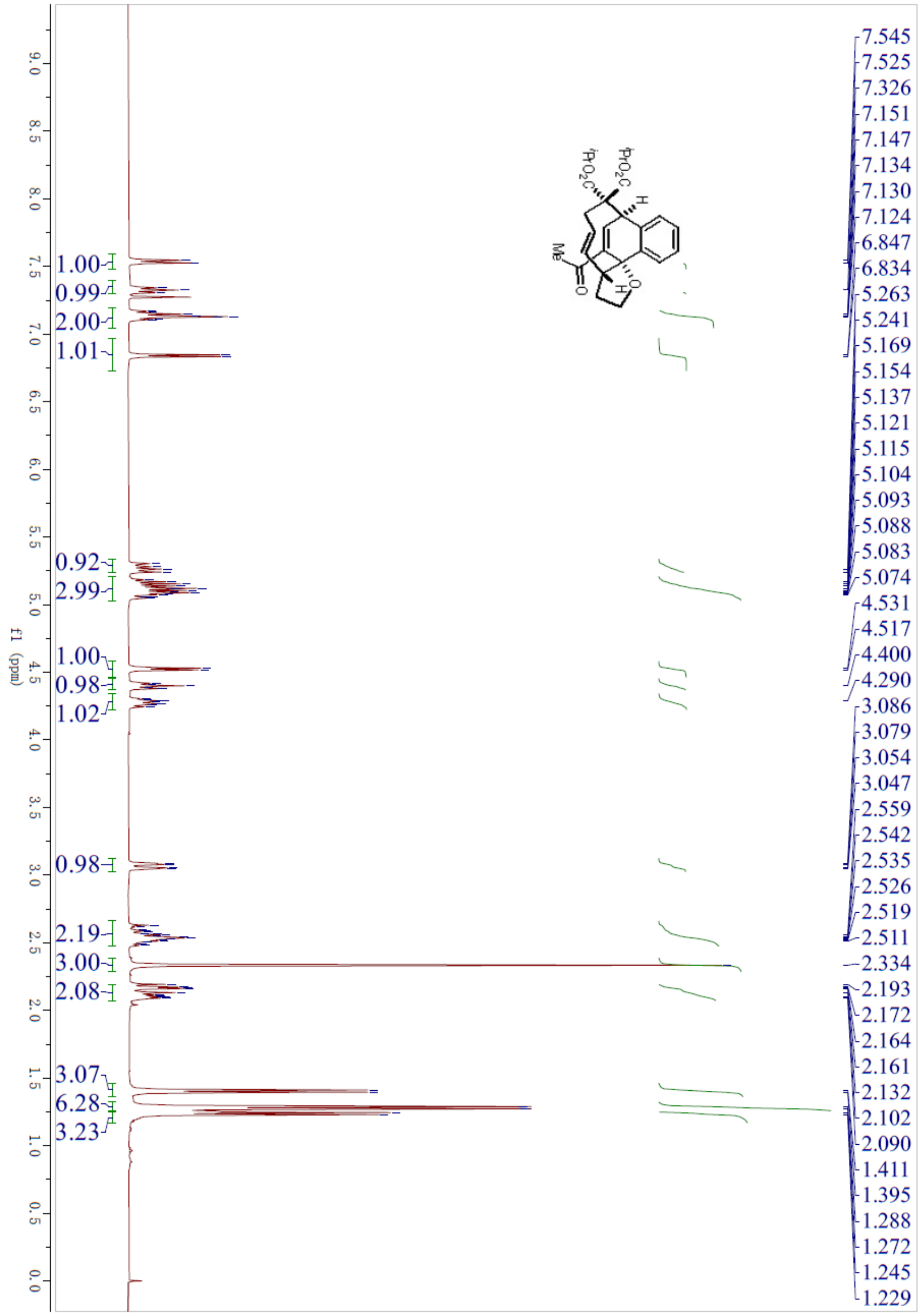

^{13}C NMR Spectrum (100 MHz, CDCl3) of *E*-**2h**

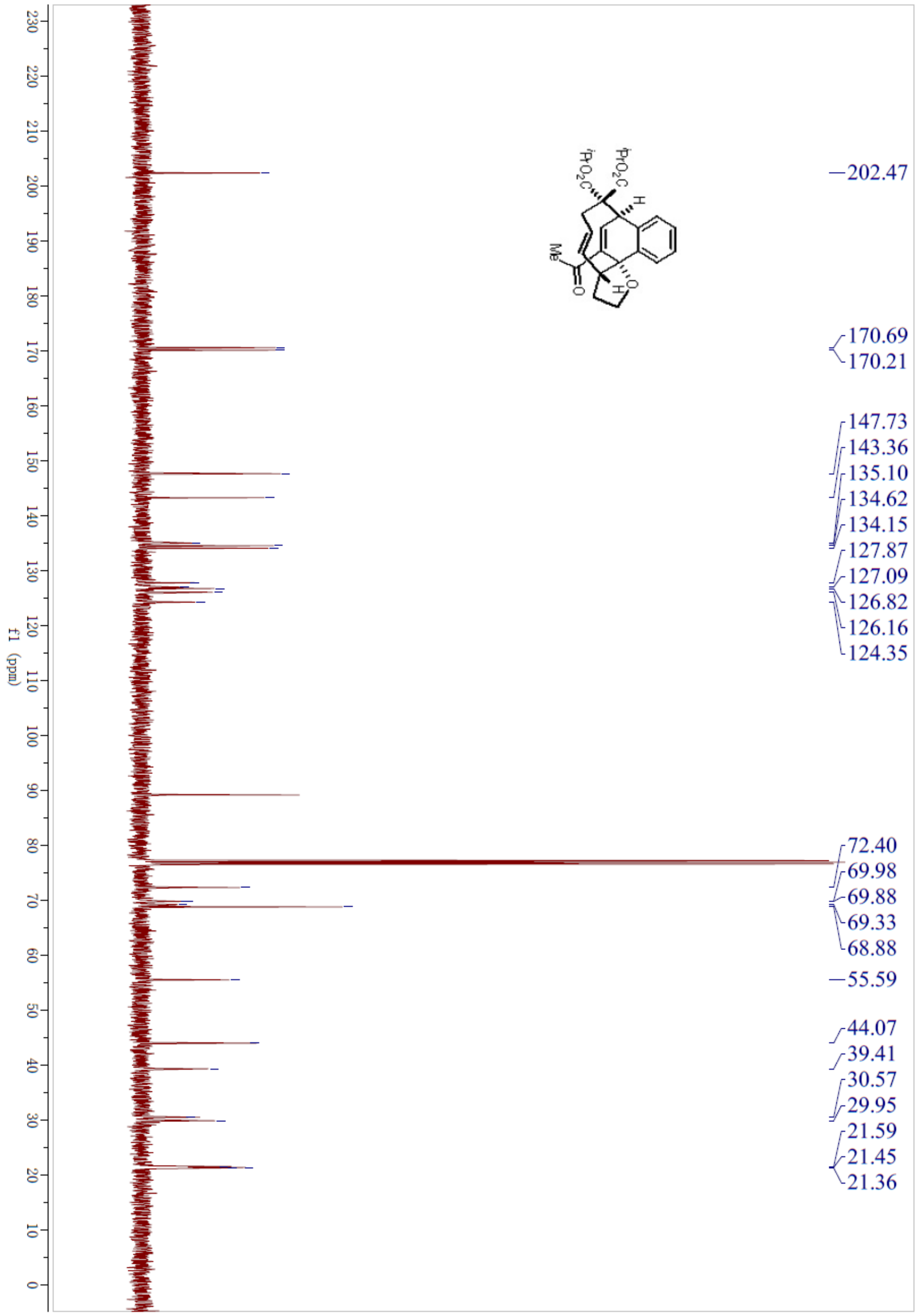

^{1}H NMR Spectrum (400 MHz, $CDCl_3$) of *E*-**2i**

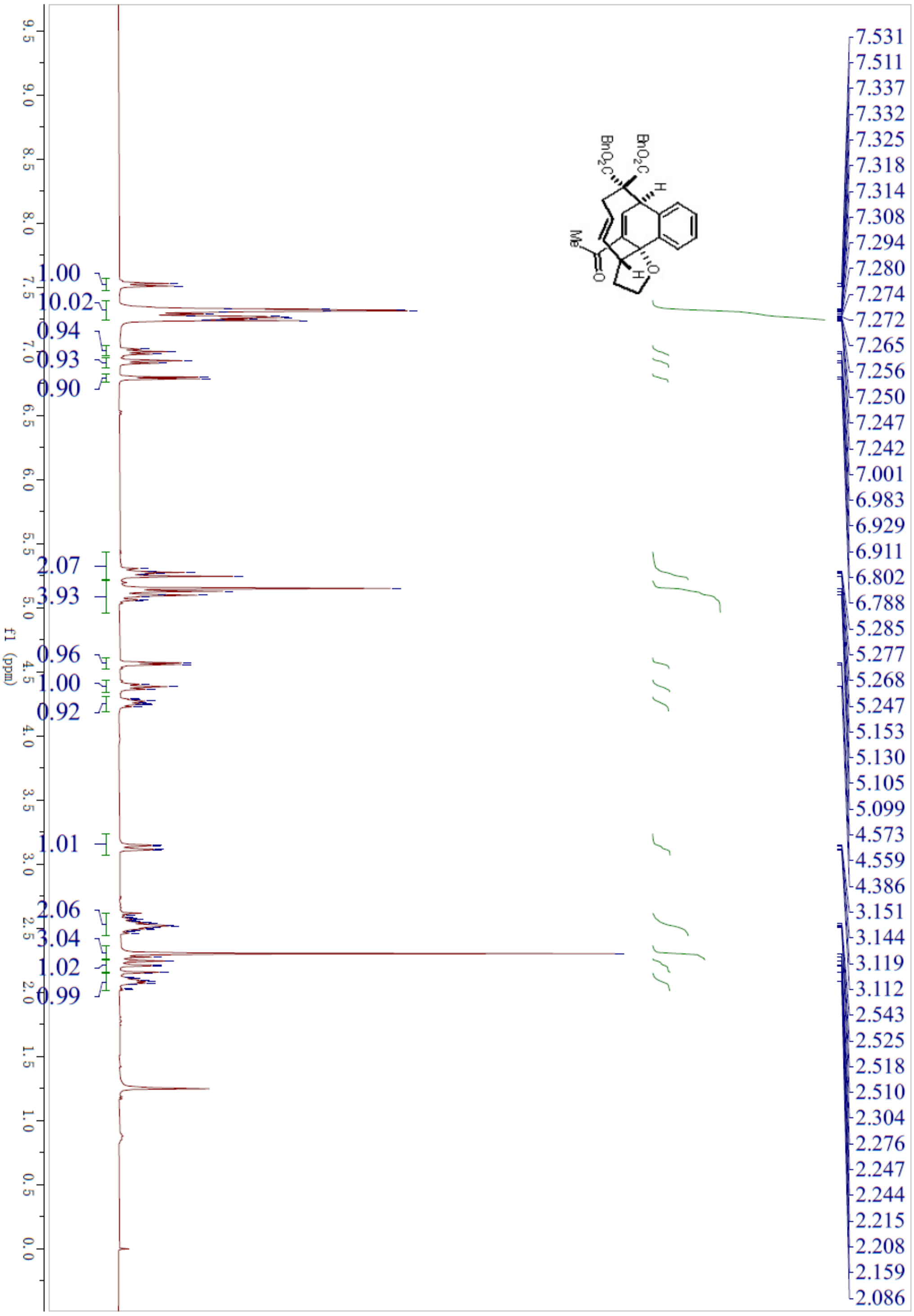

^{13}C NMR Spectrum (100 MHz, $CDCl_3$) of *E*-**2i**

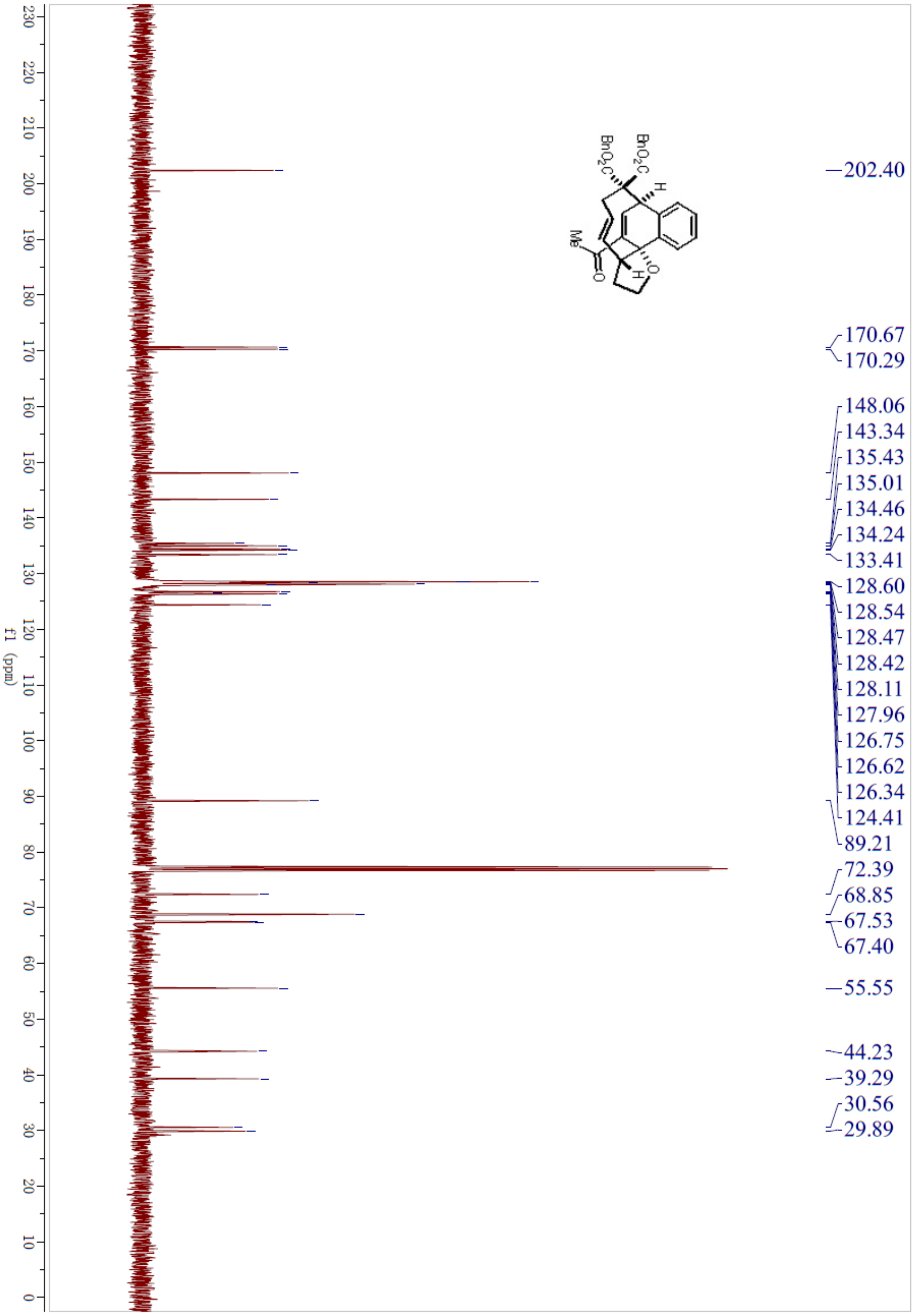

^{1}H NMR Spectrum (400 MHz, $CDCl_3$) of *E*-**2j**

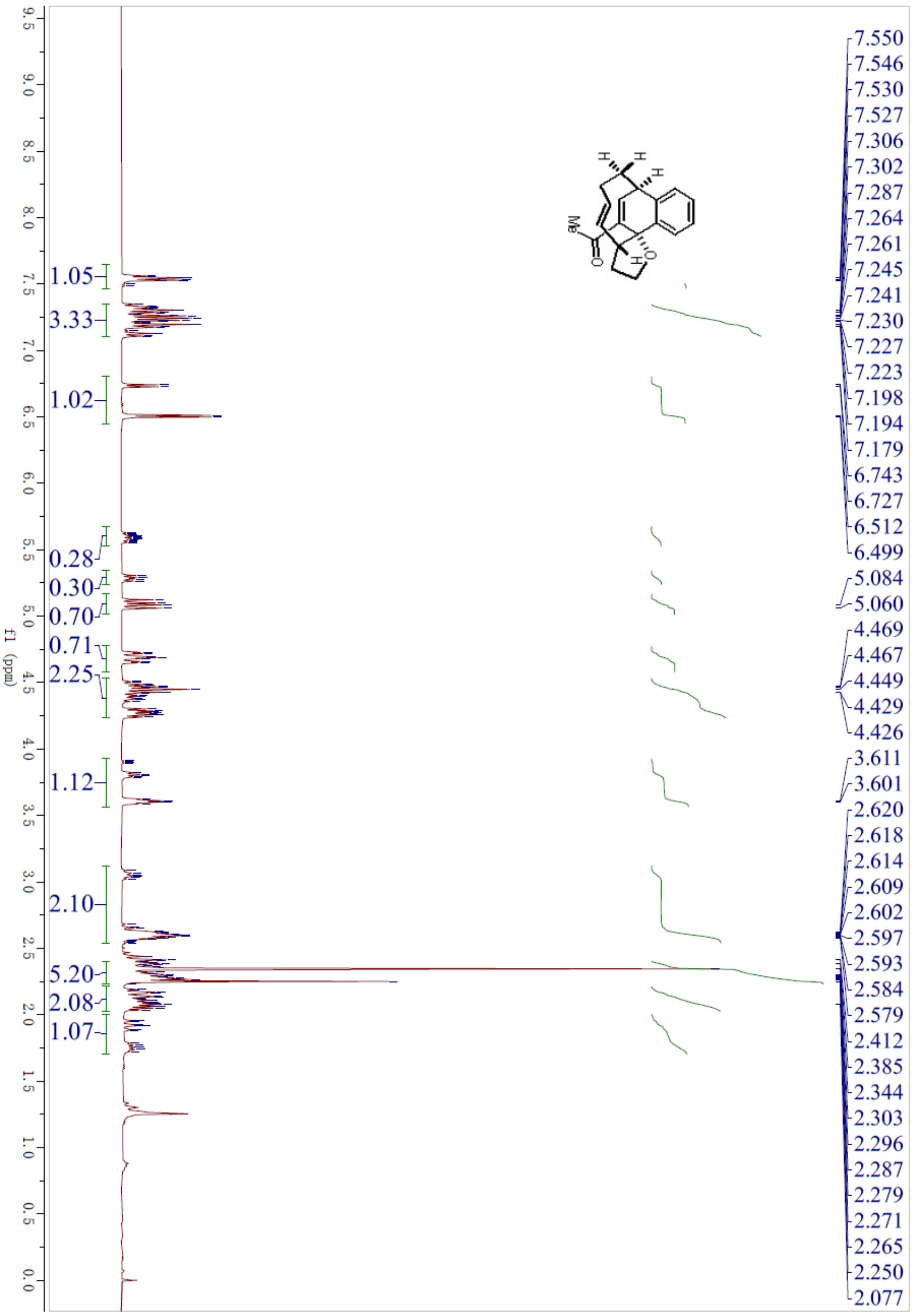

^{13}C NMR Spectrum (100 MHz, $CDCl_3$) of *E*-**2j**

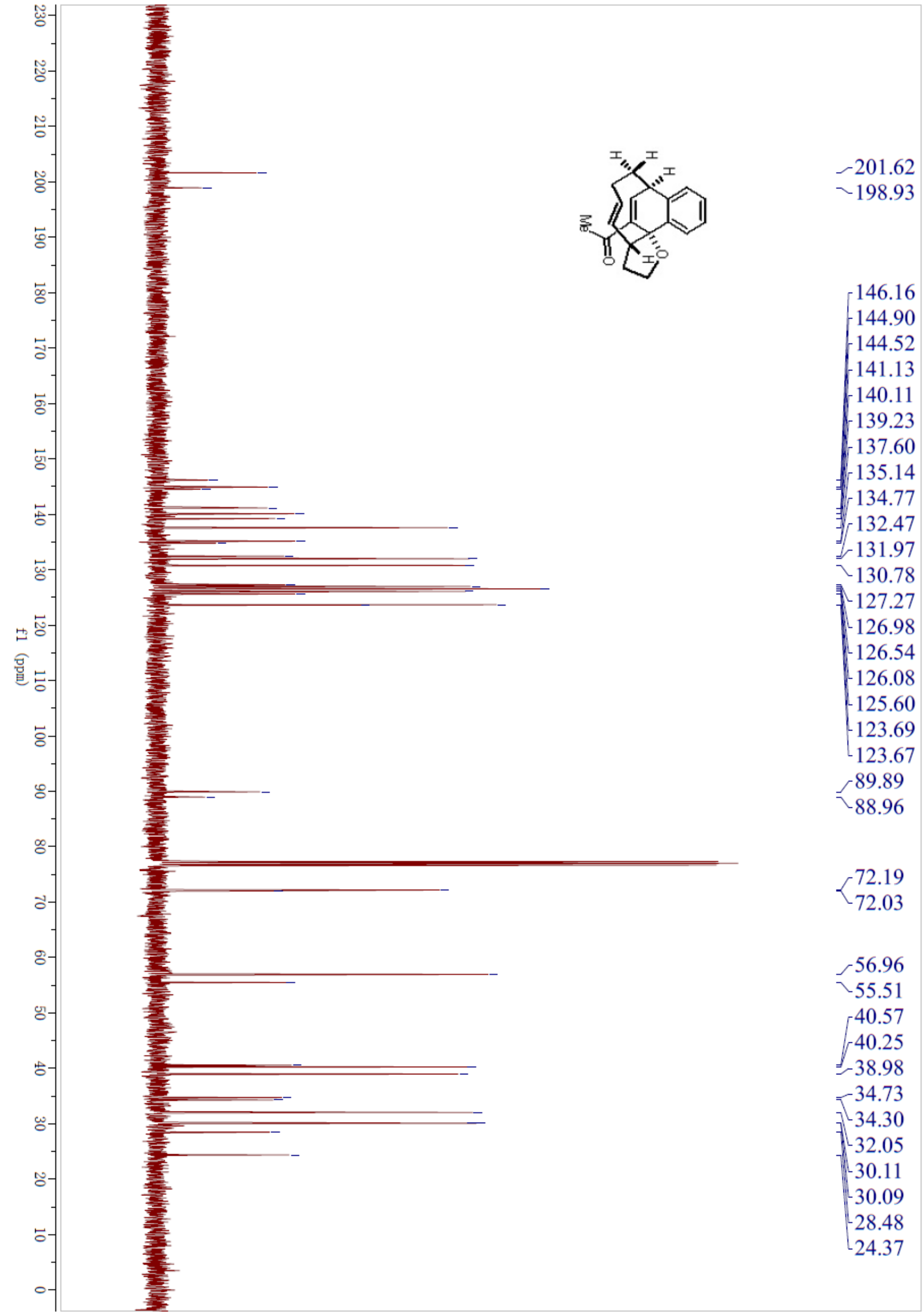

^{1}H NMR Spectrum (400 MHz, $CDCl_3$) of *E*-**2k**

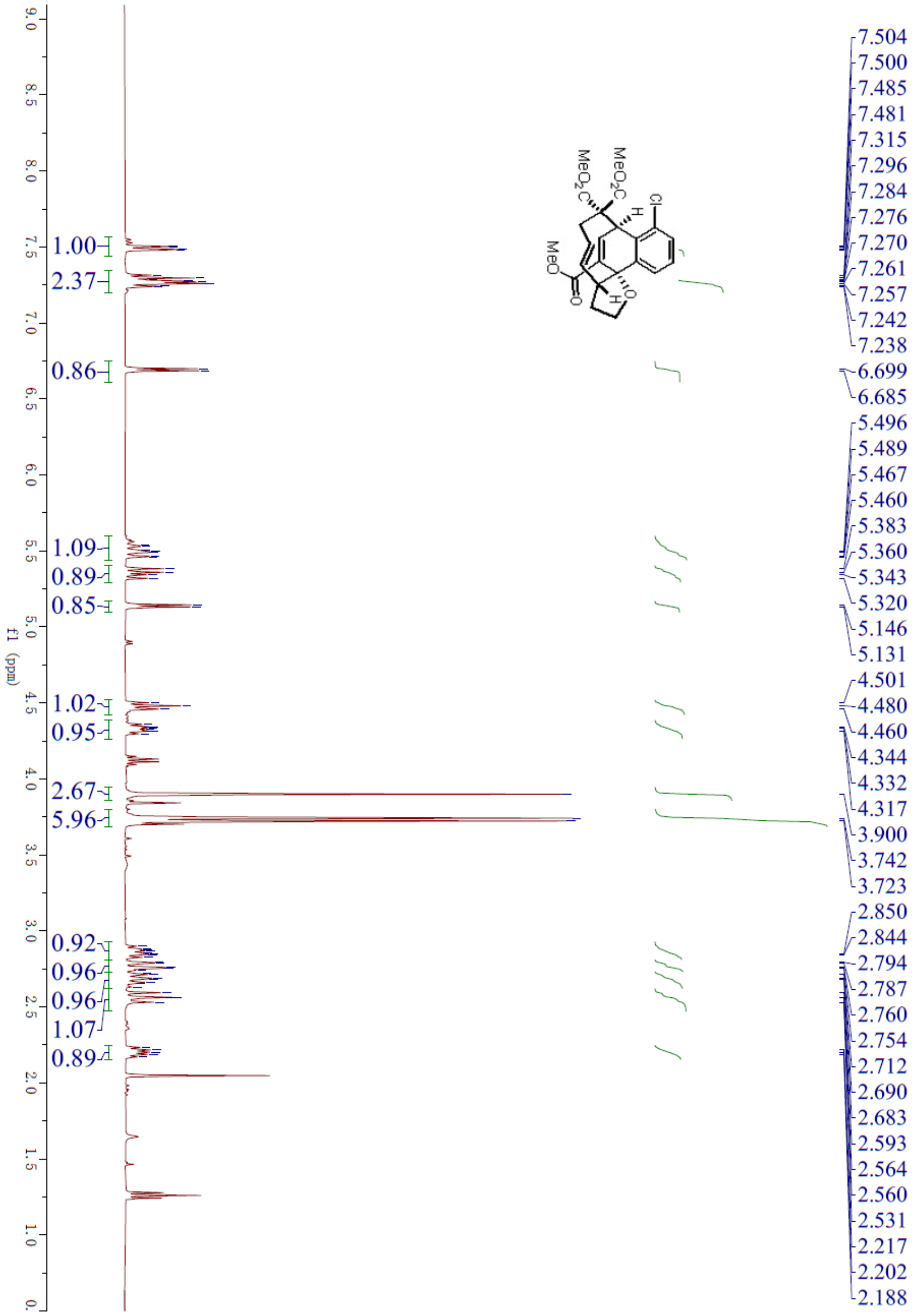

^{13}C NMR Spectrum (100 MHz, CDCl3) of *E*-**2k**

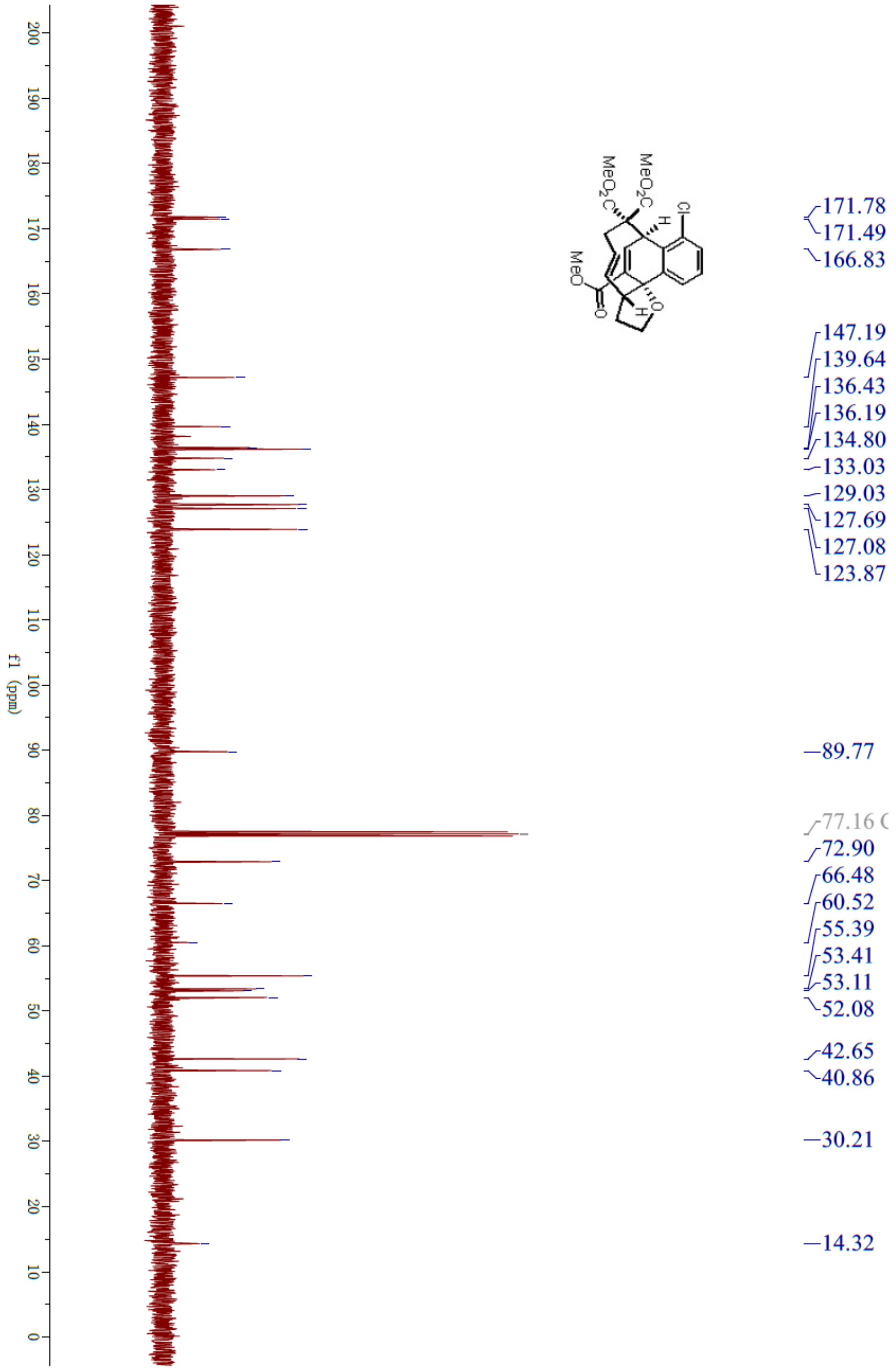

^{1}H NMR Spectrum (400 MHz, $CDCl_3$) of *E*-**2l**

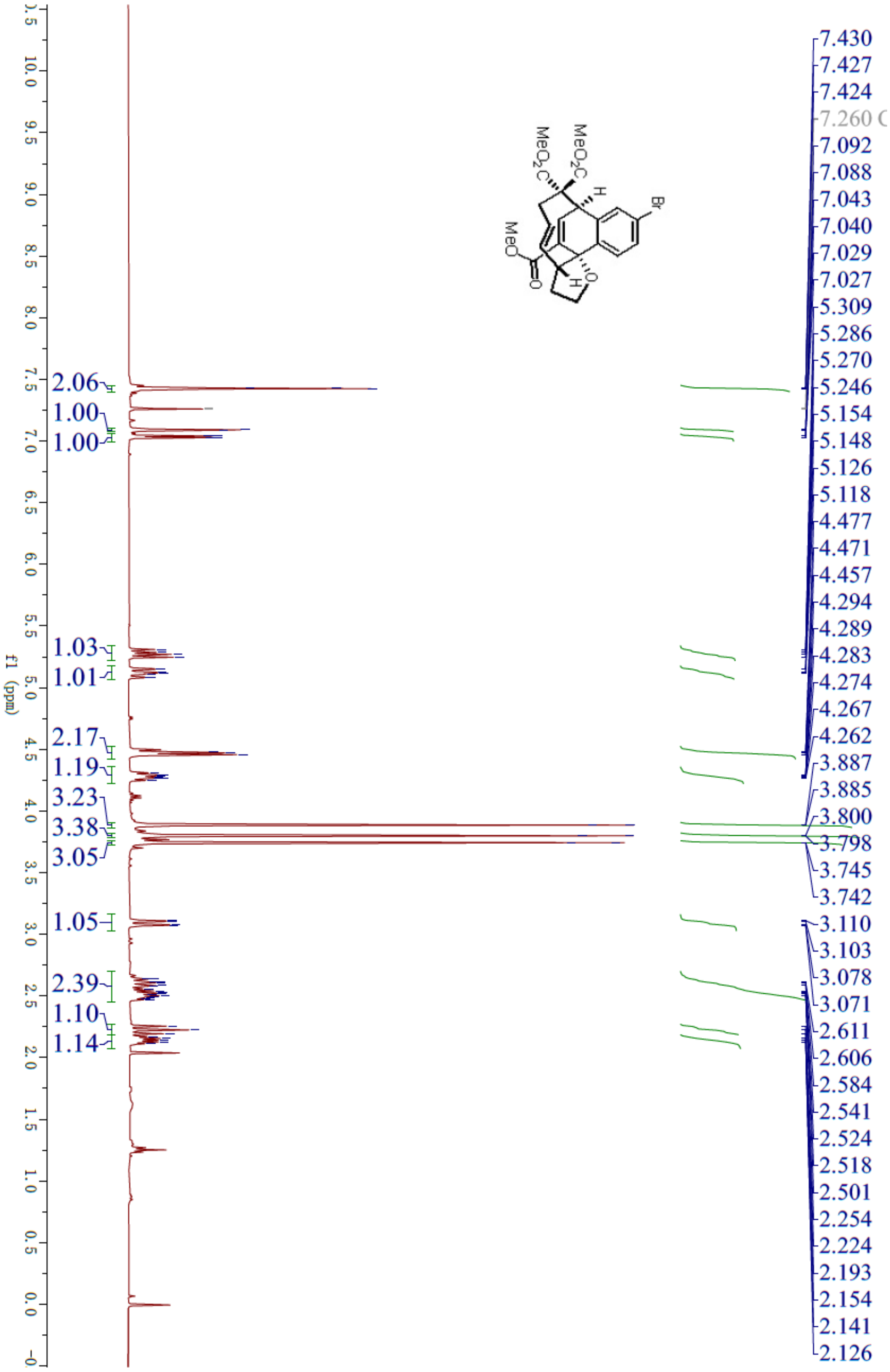

^{13}C NMR Spectrum (100 MHz, CDCl3) of *E*-**2l**

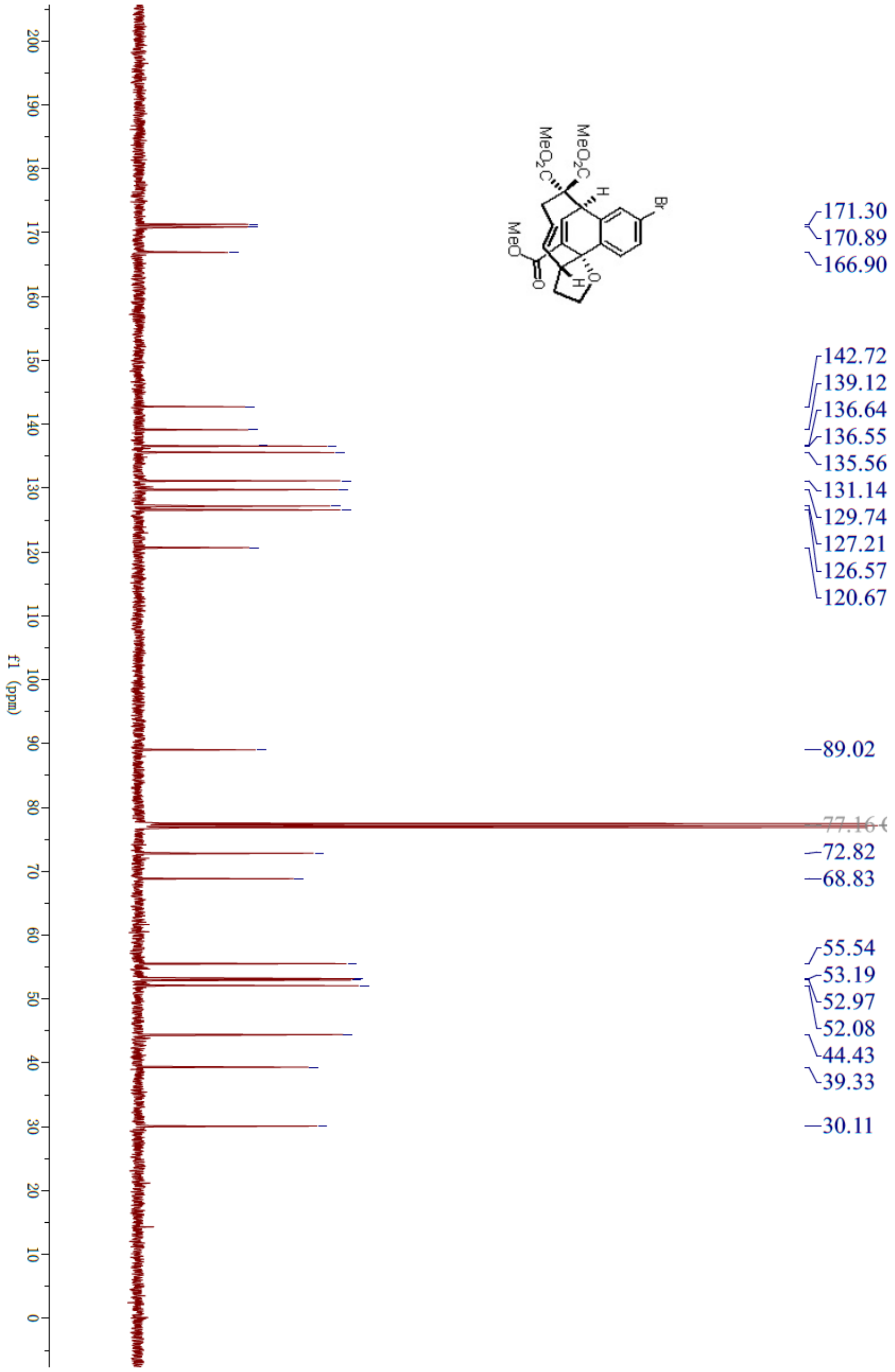

^{1}H NMR Spectrum (400 MHz, CDCl3) of *E*-**2m**

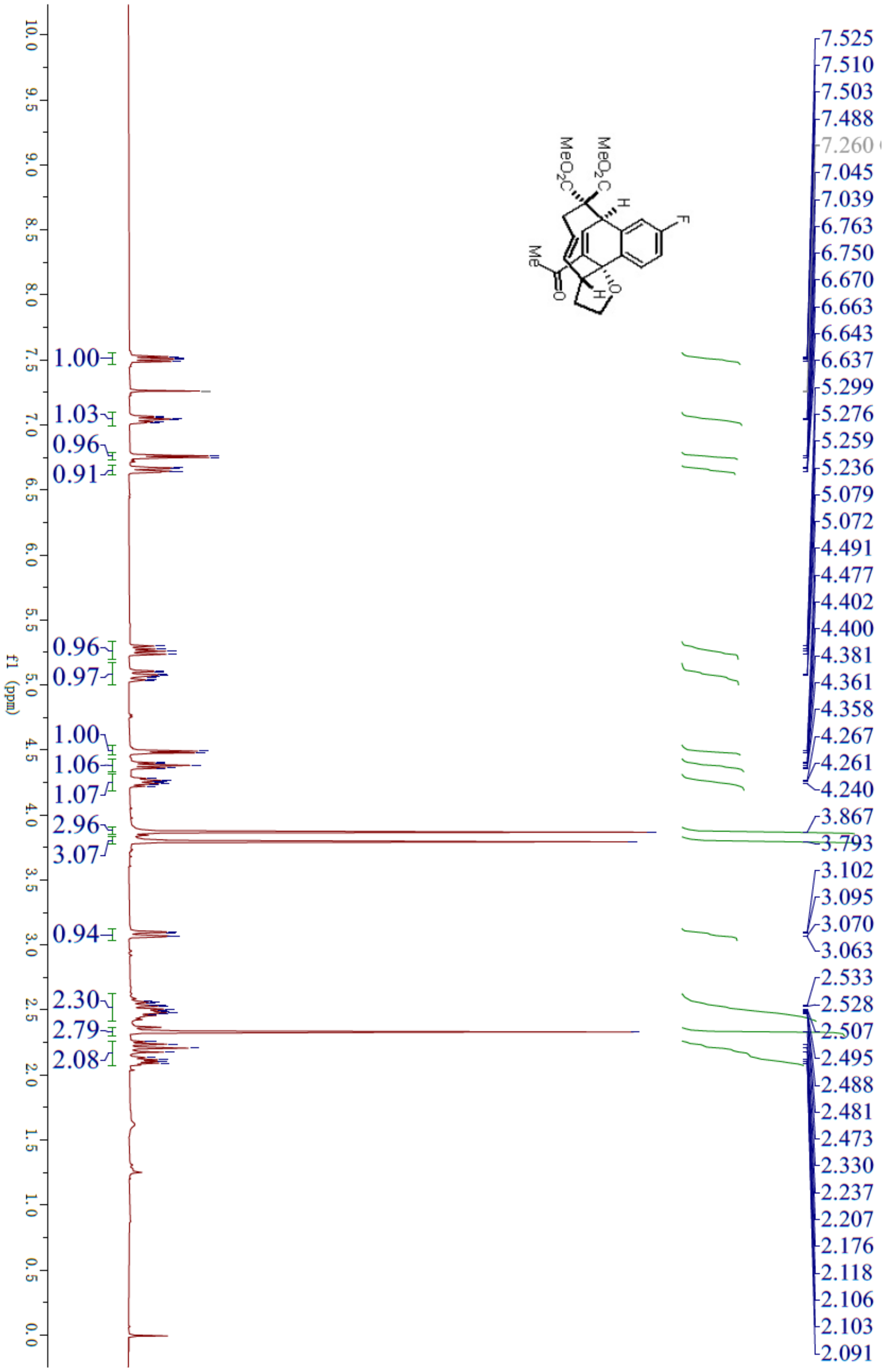

^{13}C NMR Spectrum (100 MHz, $CDCl_3$) of *E*-**2m**

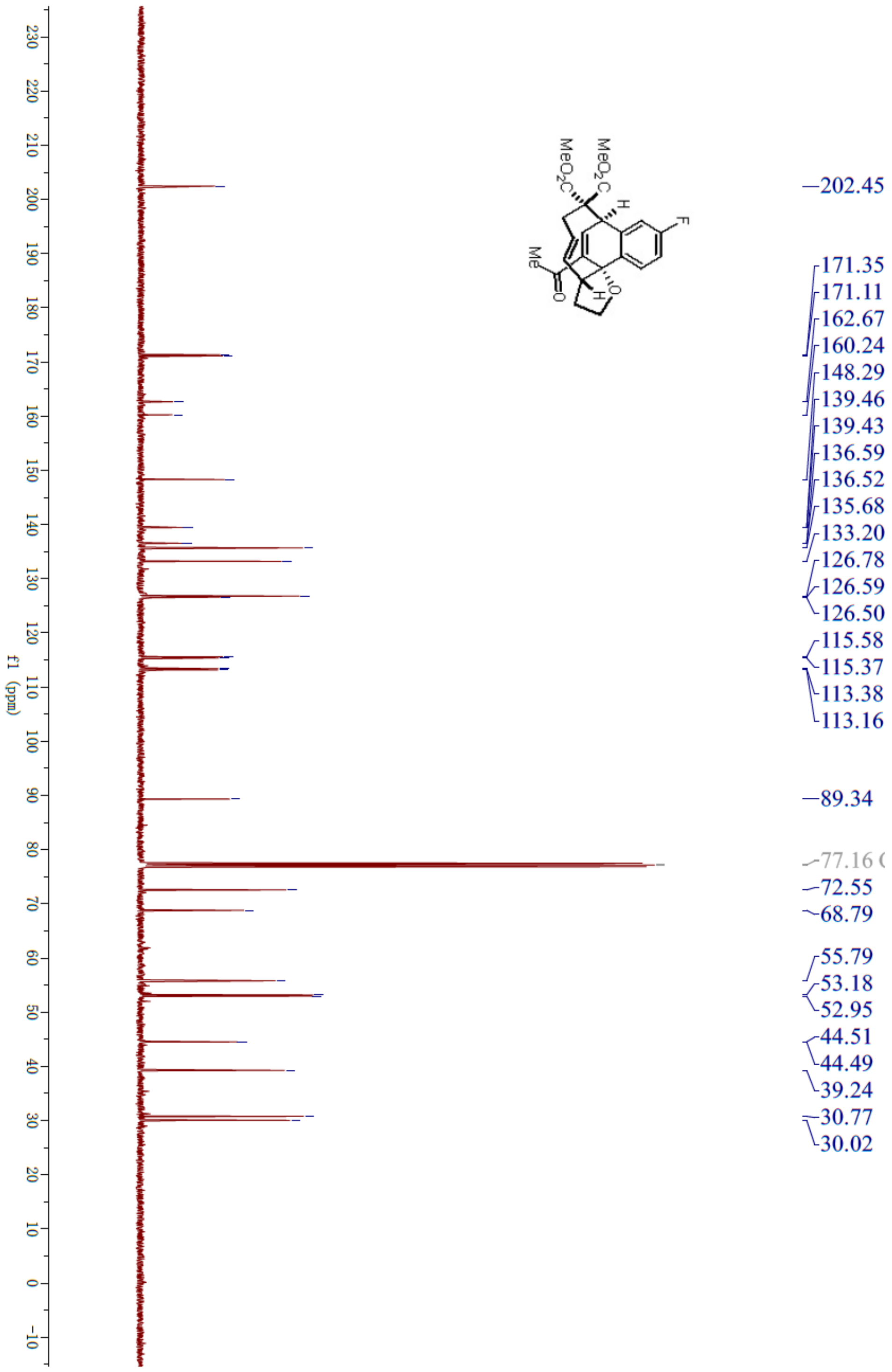

19F NMR Spectrum (376 MHz, $CDCl_3$) of *E*-**2m**

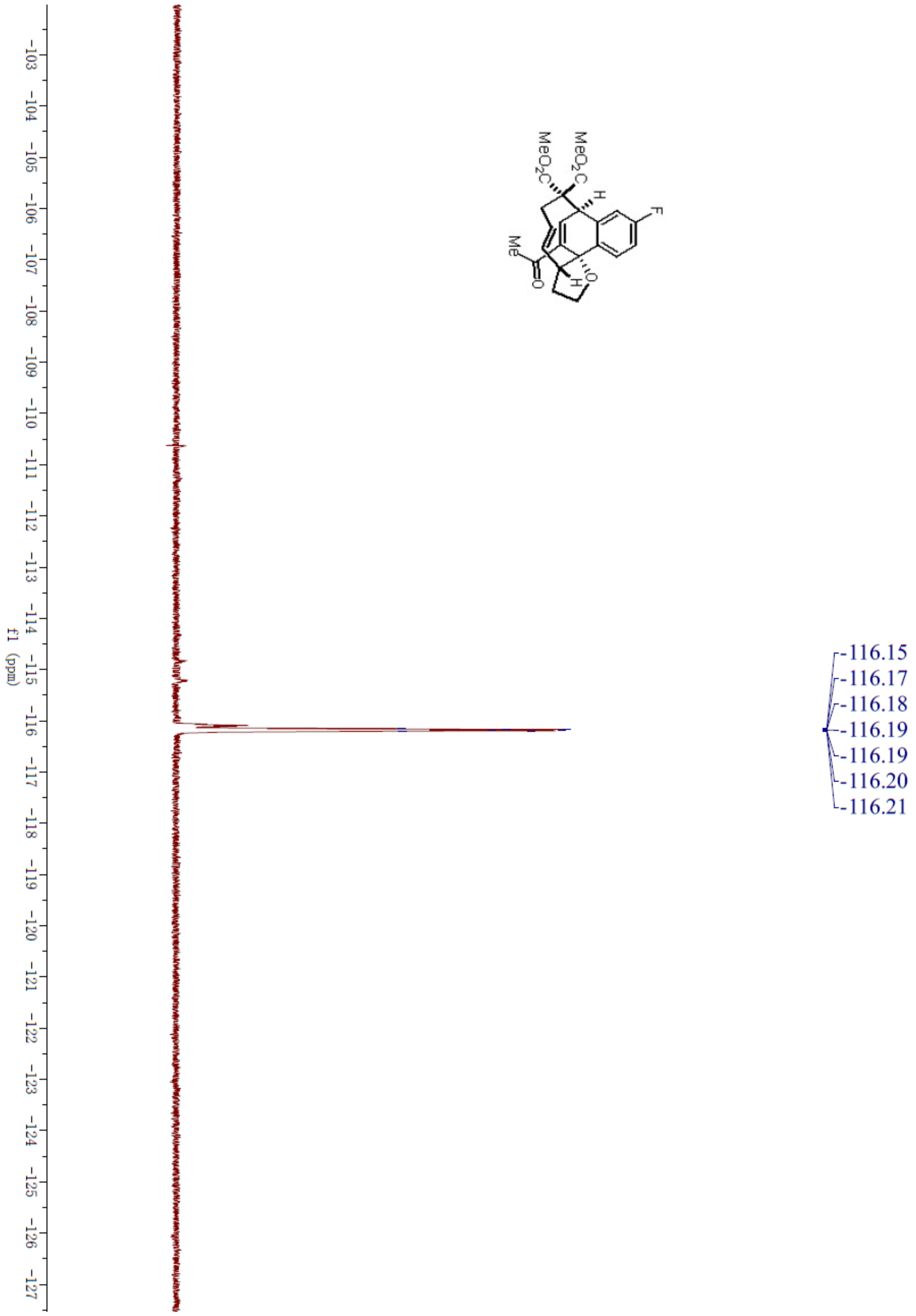

^{1}H NMR Spectrum (400 MHz, CDCl3) of *E*-**2n**

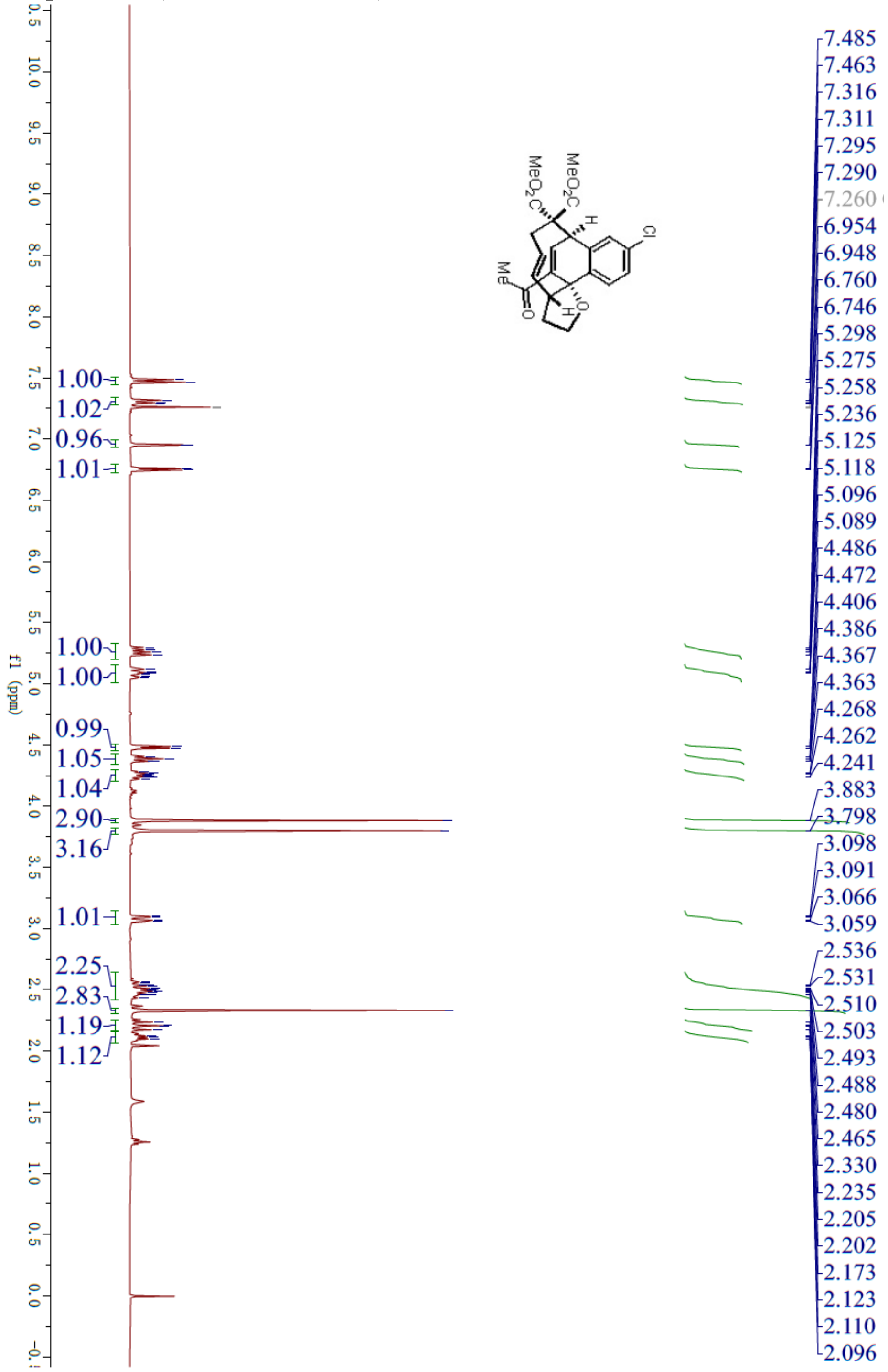

^{13}C NMR Spectrum (100 MHz, $CDCl_3$) of *E*-**2n**

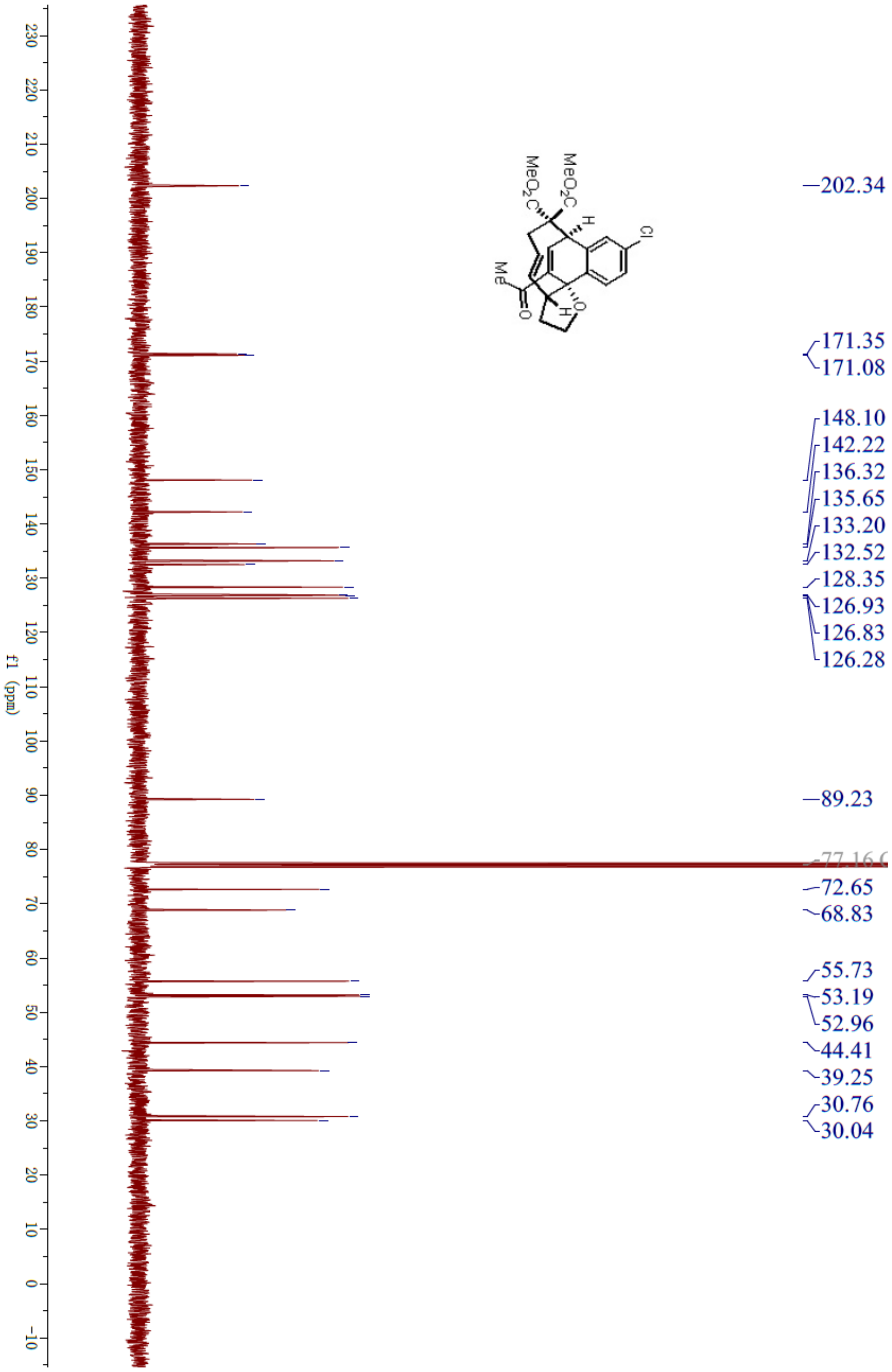

^{1}H NMR Spectrum (400 MHz, $CDCl_3$) of *E*-**2o**

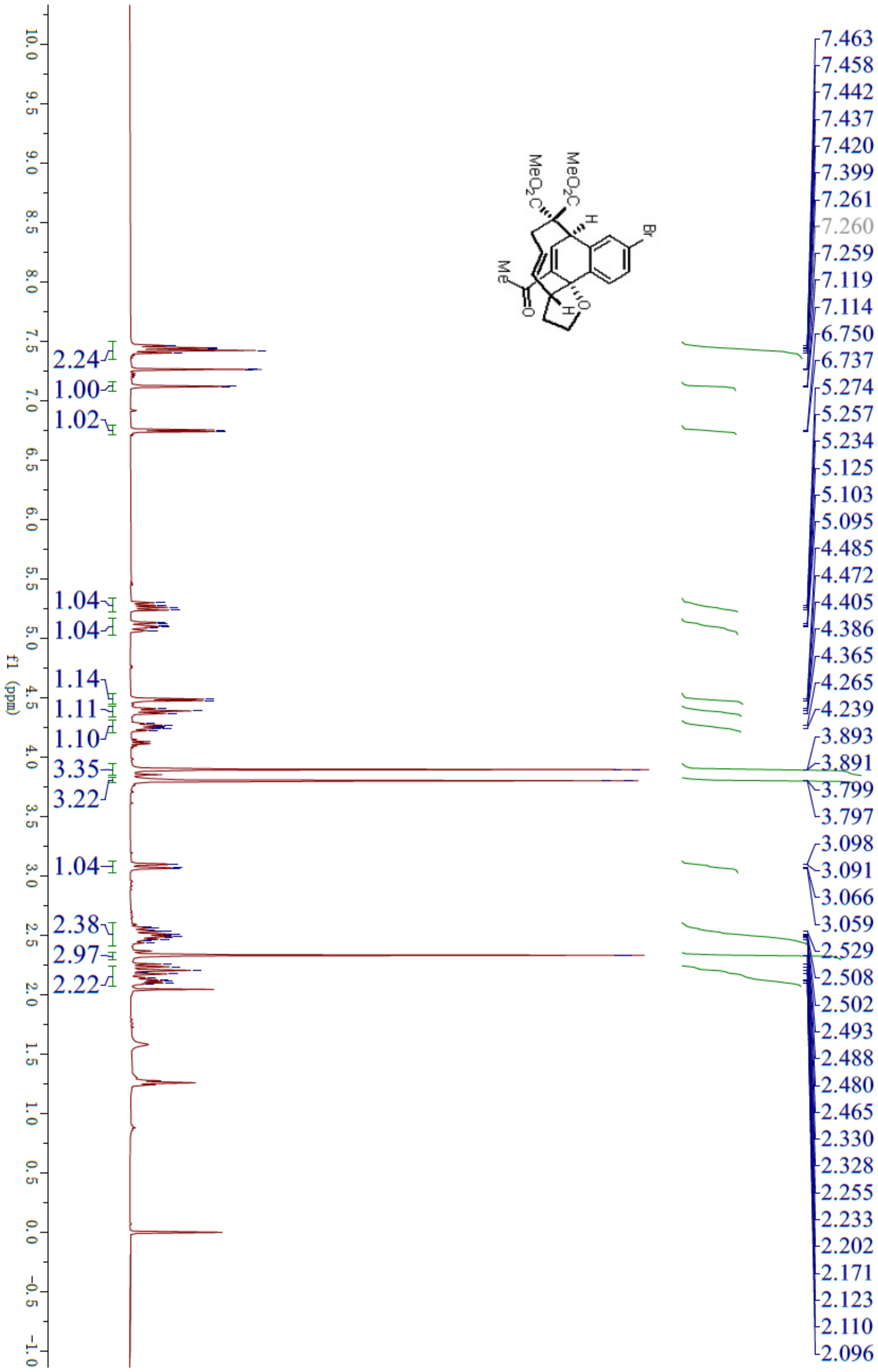

^{13}C NMR Spectrum (100 MHz, $CDCl_3$) of *E*-**2o**

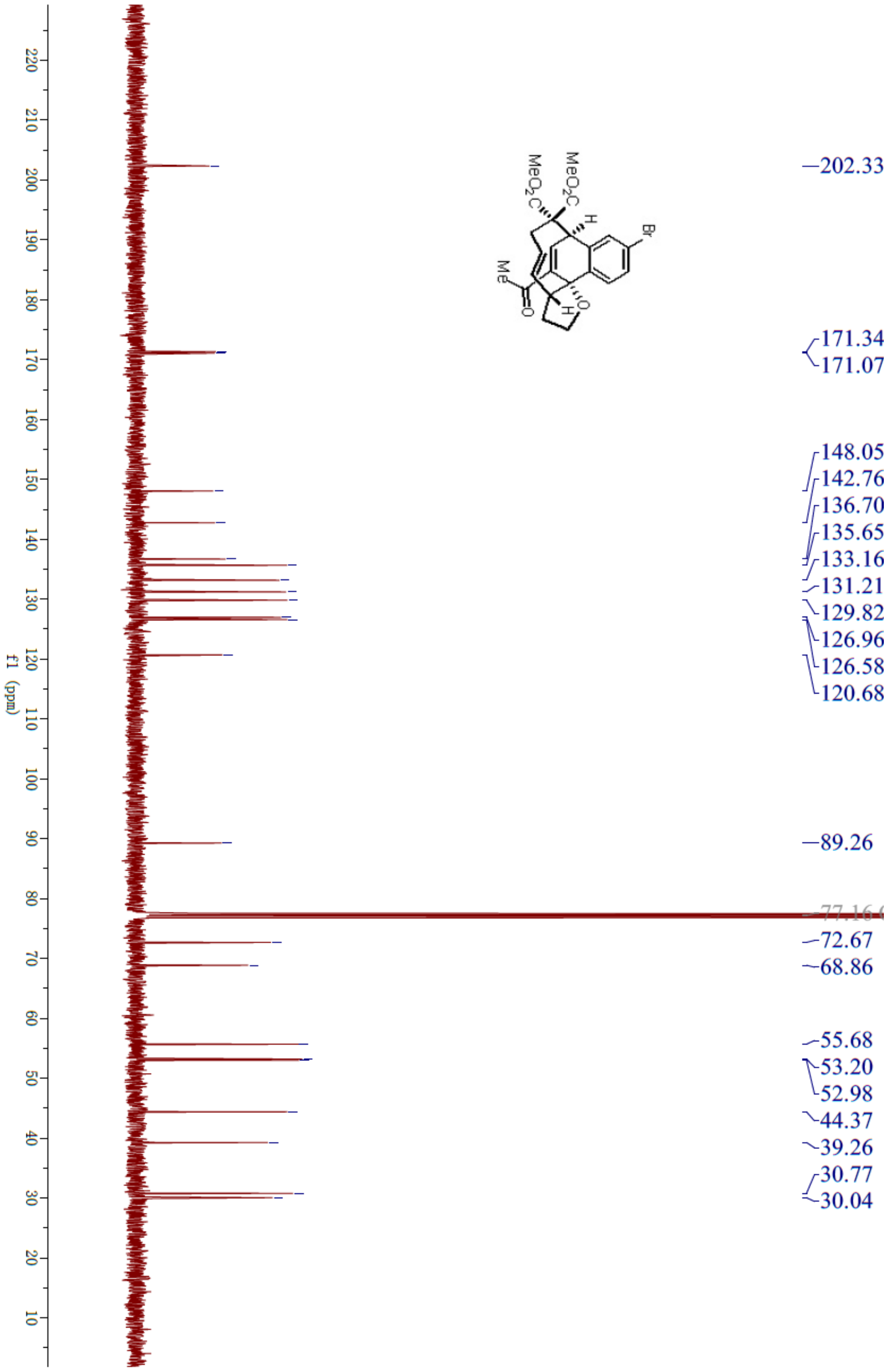

^{1}H NMR Spectrum (400 MHz, $CDCl_3$) of *E*-**2p**

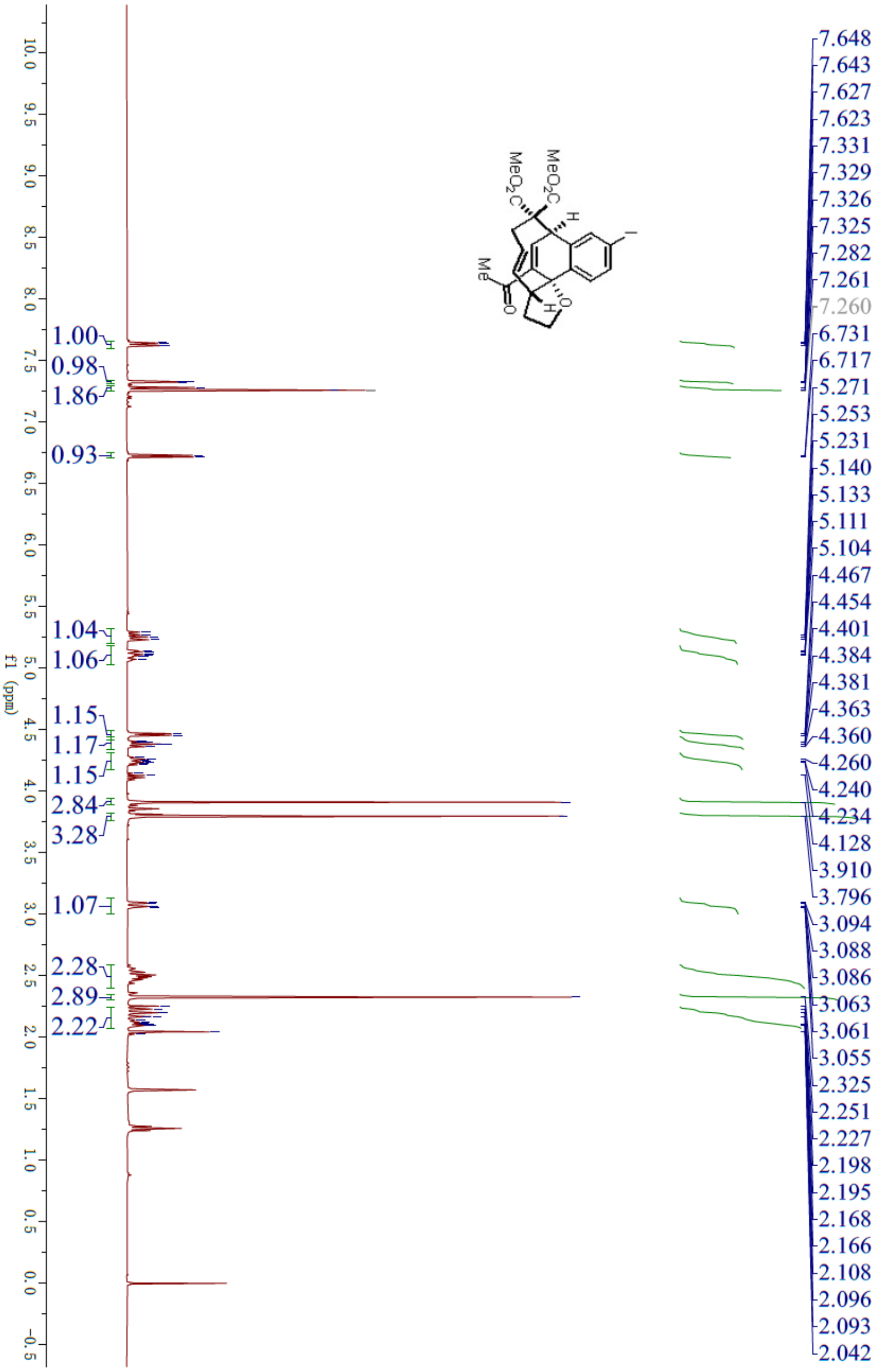

^{13}C NMR Spectrum (100 MHz, $CDCl_3$) of *E*-**2p**

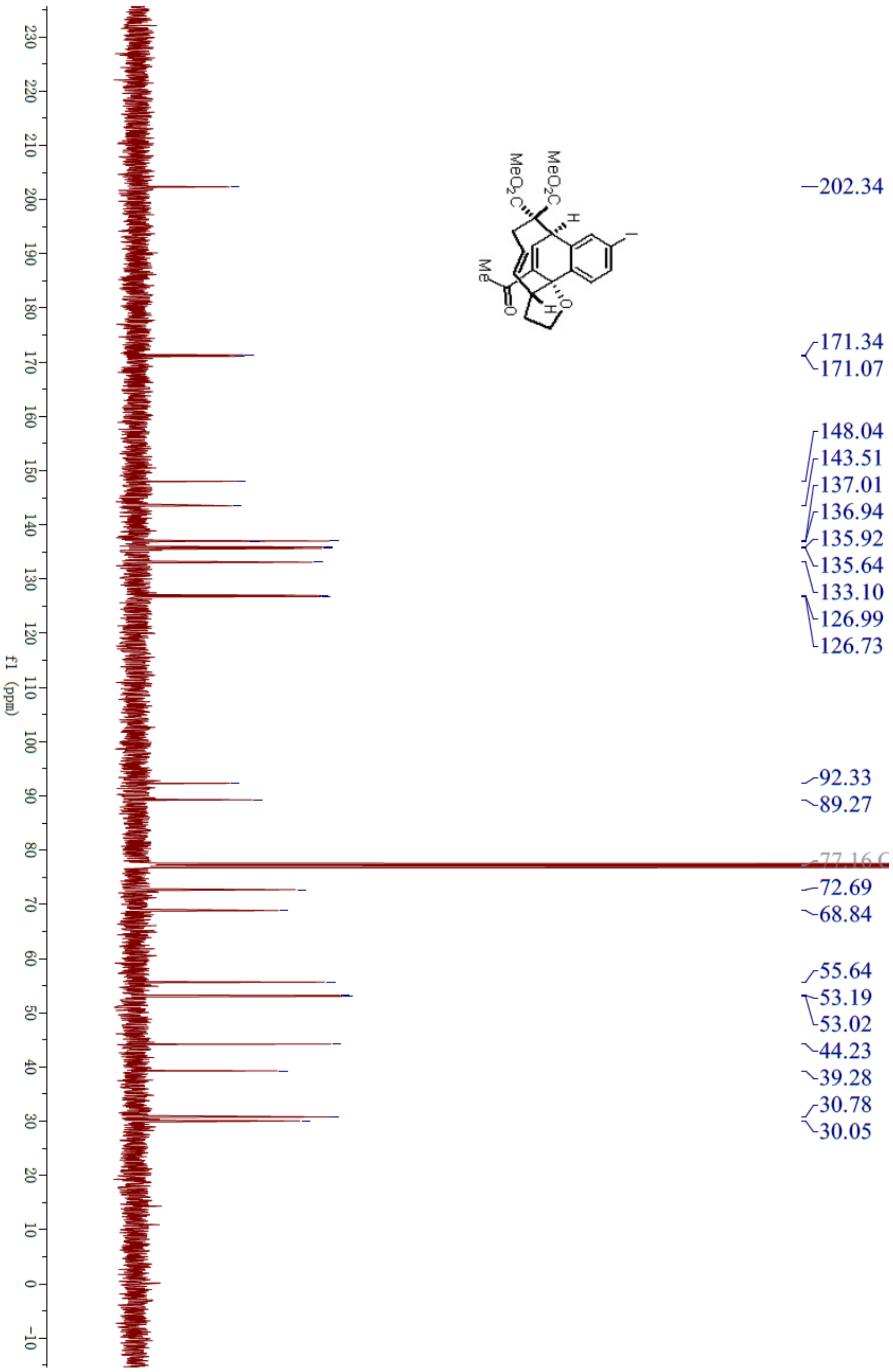

^{1}H NMR Spectrum (400 MHz, CDCl3) of *E*-**2q**

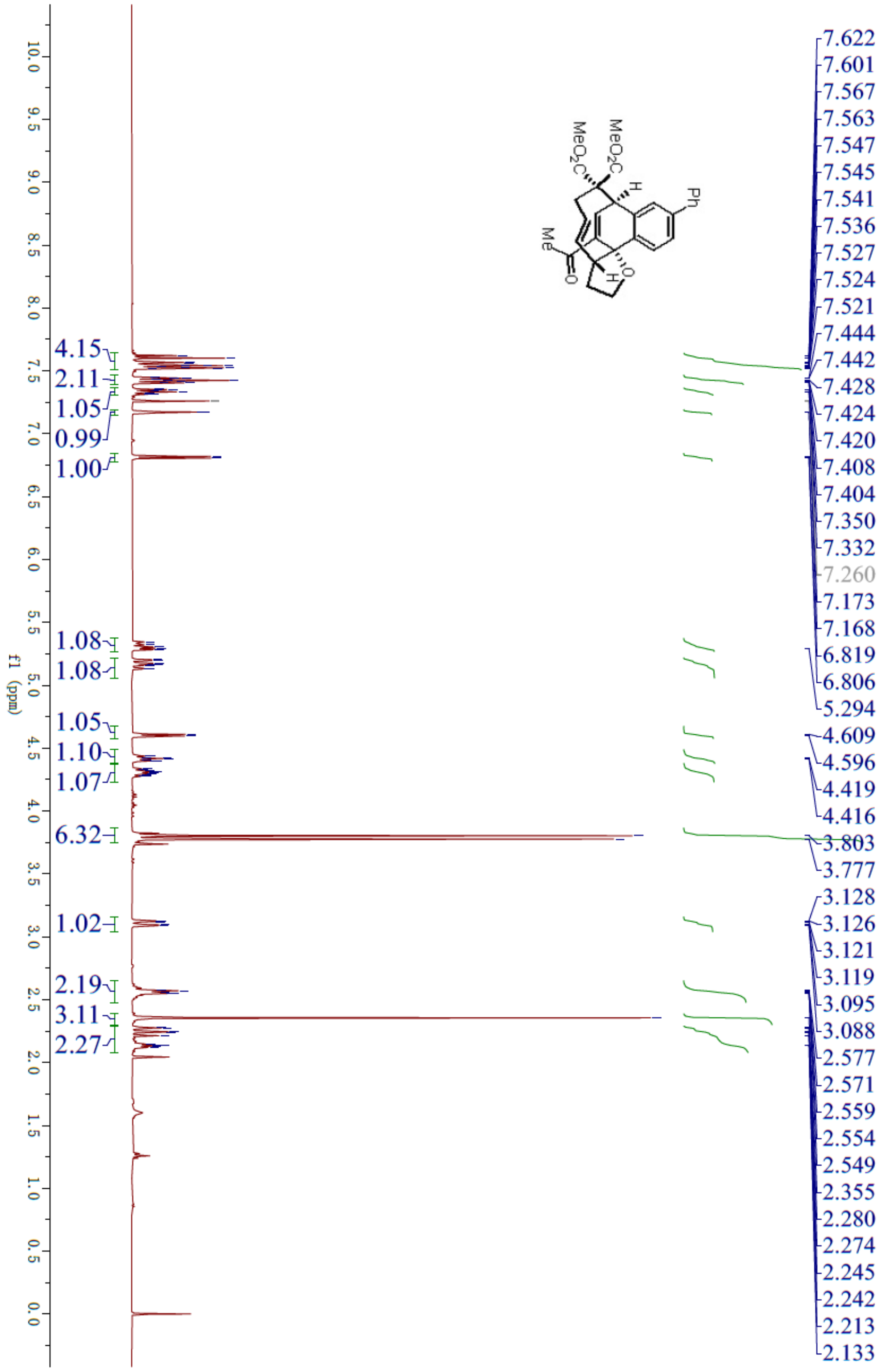

^{13}C NMR Spectrum (100 MHz, $CDCl_3$) of *E*-**2q**

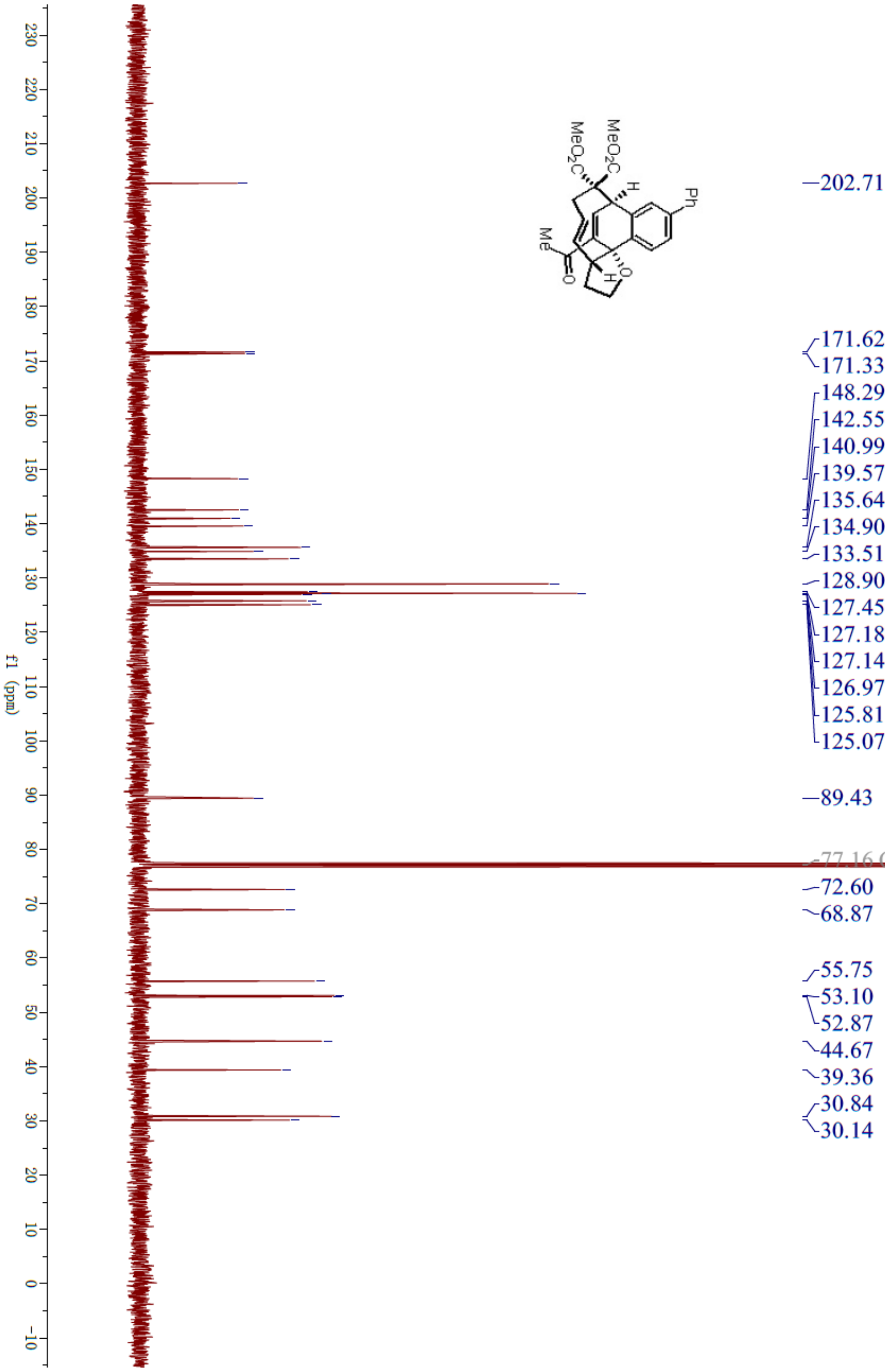

^{1}H NMR Spectrum (400 MHz, $CDCl_3$) of *E*-**2r**

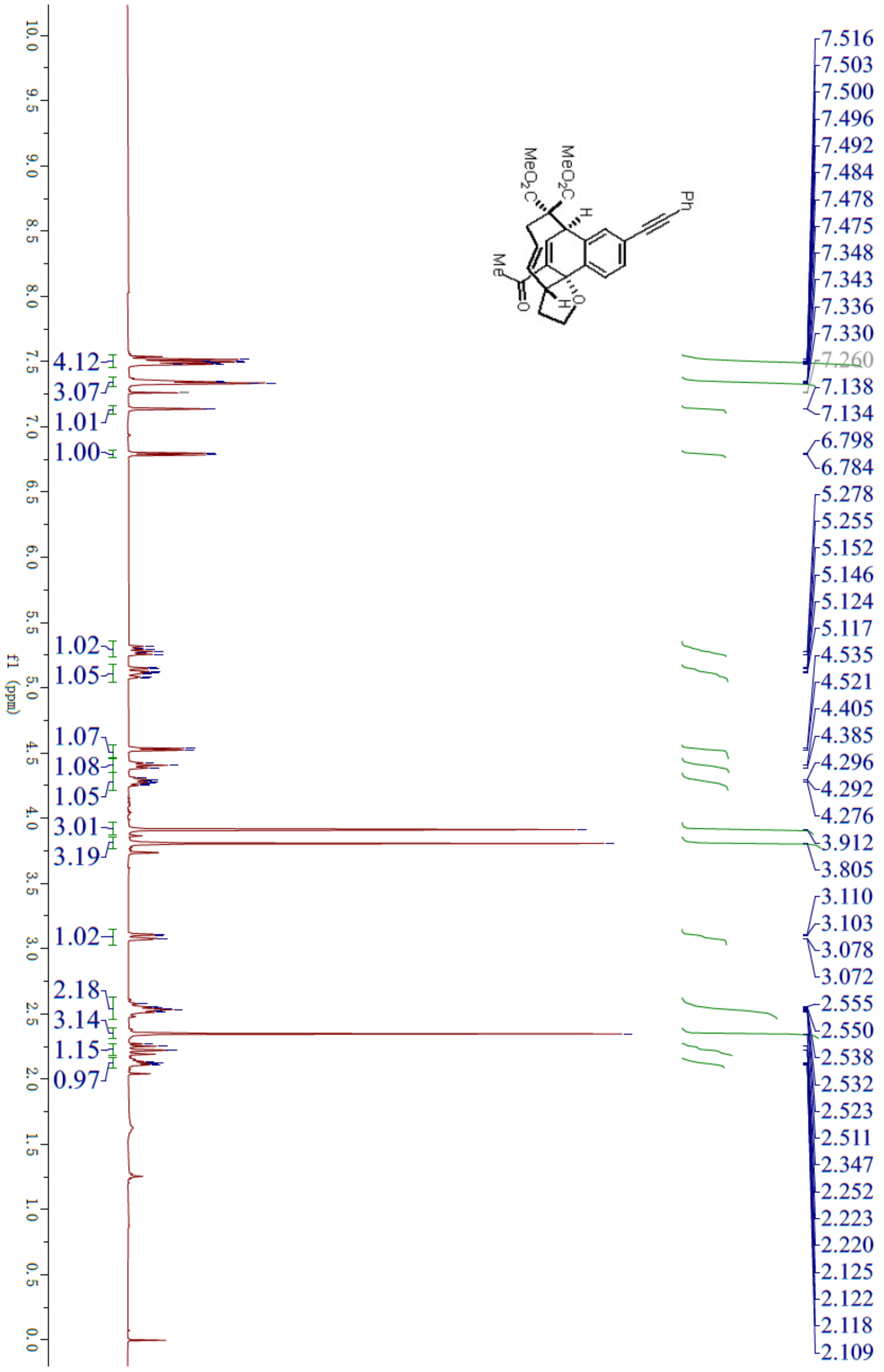

^{13}C NMR Spectrum (100 MHz, $CDCl_3$) of *E*-**2r**

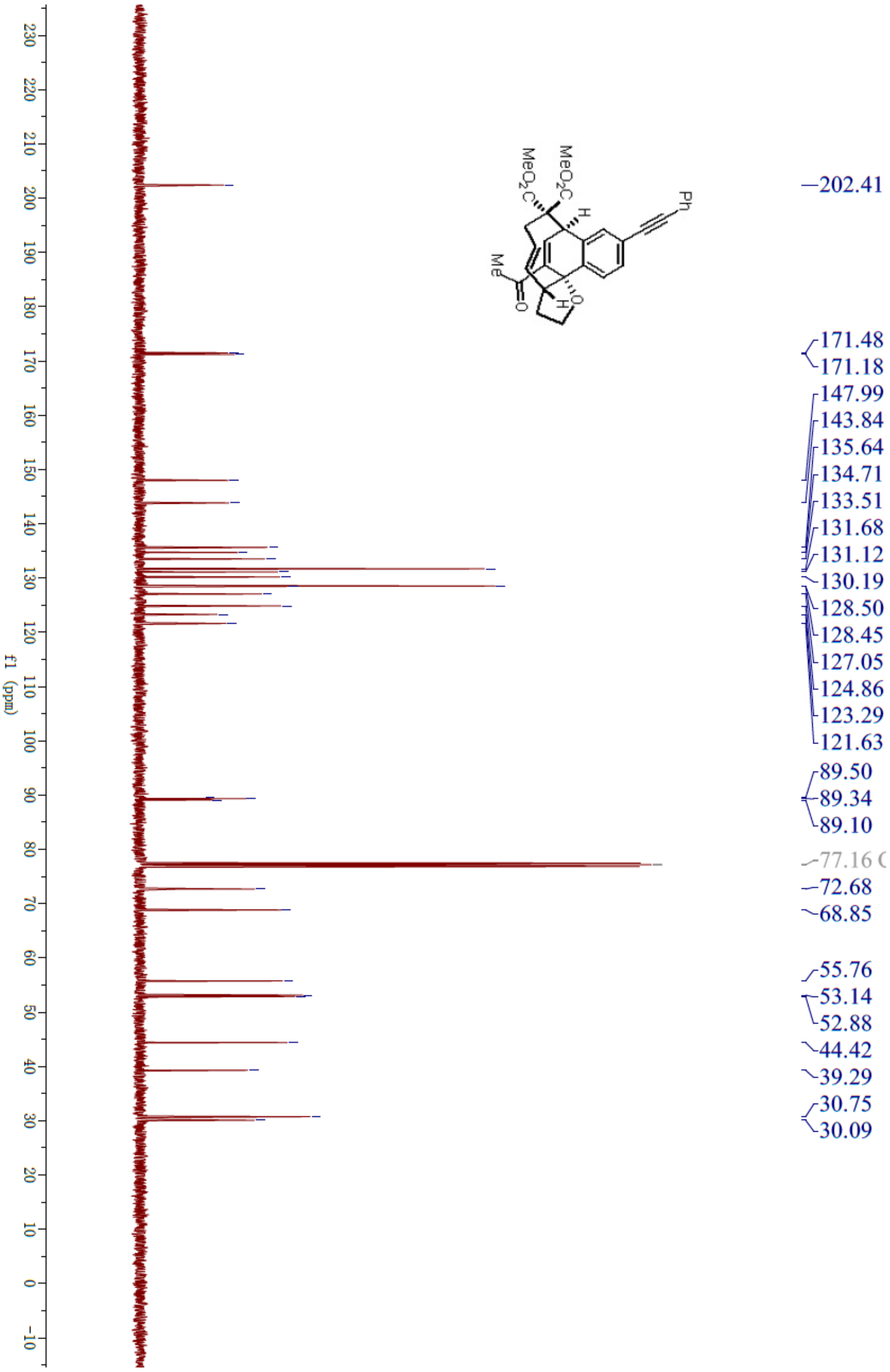

^{1}H NMR Spectrum (400 MHz, $CDCl_3$) of *E*-**2s**

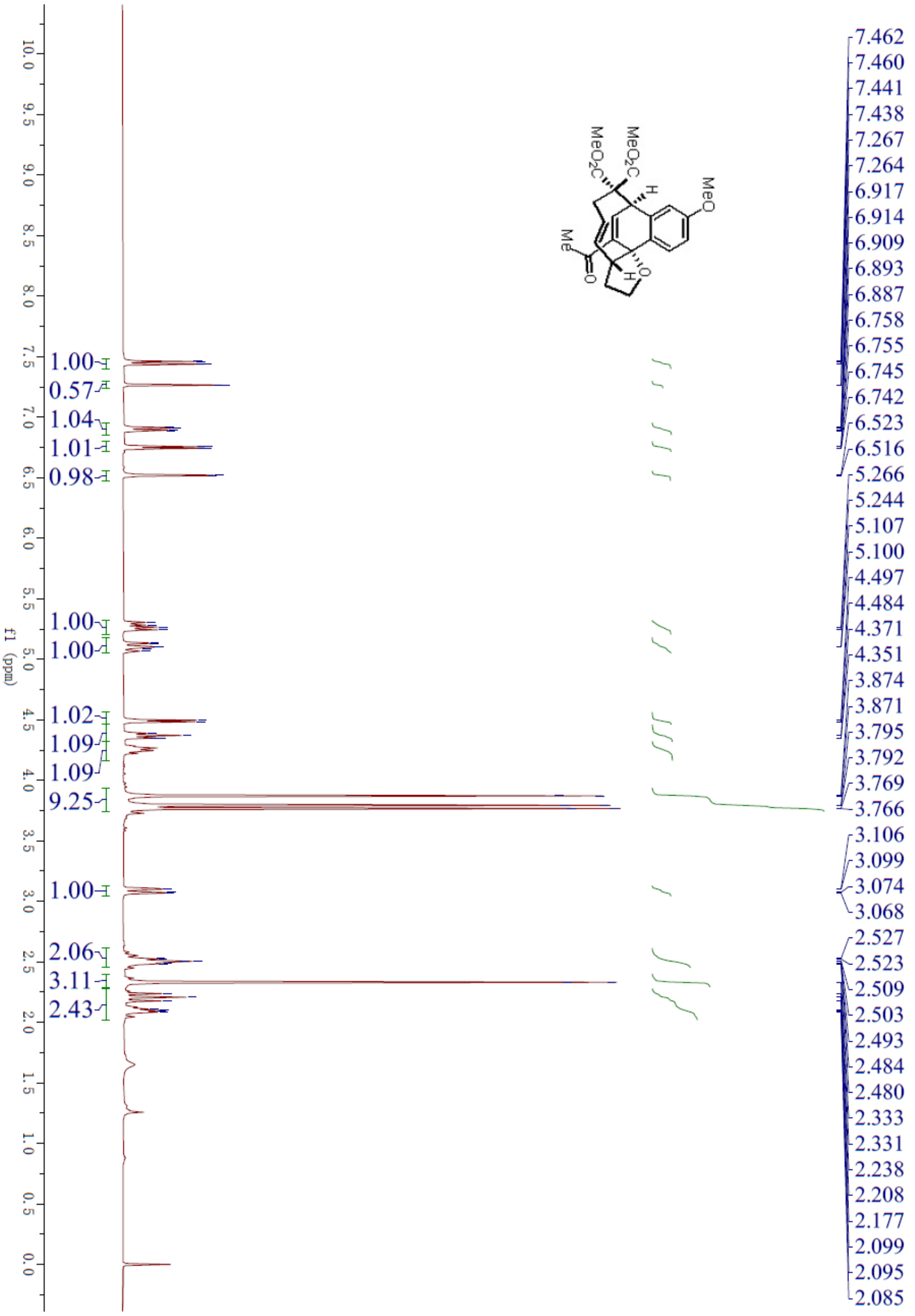

^{13}C NMR Spectrum (100 MHz, $CDCl_3$) of *E*-**2s**

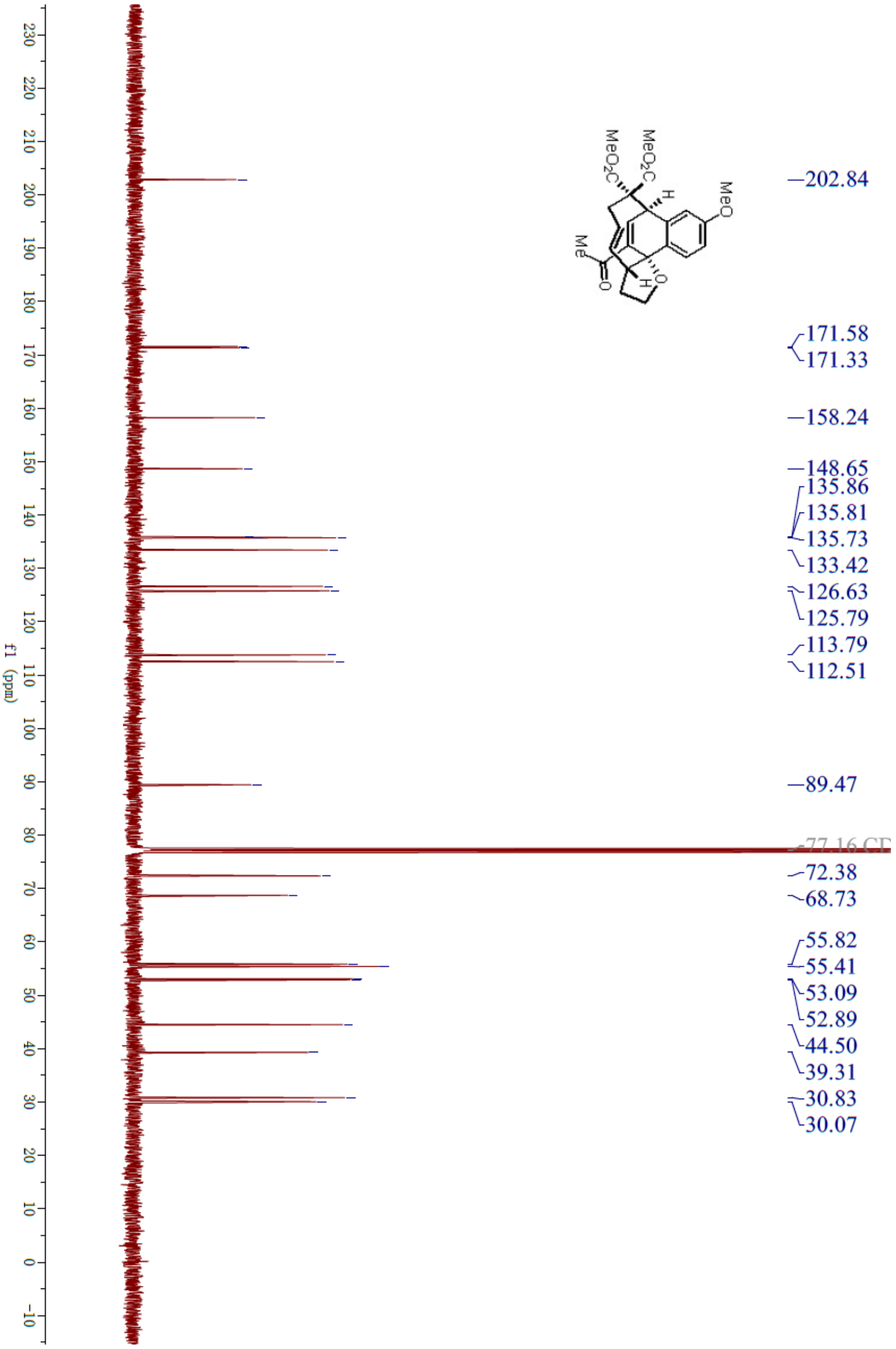

^{1}H NMR Spectrum (400 MHz, $CDCl_3$) of *E*-**2t**

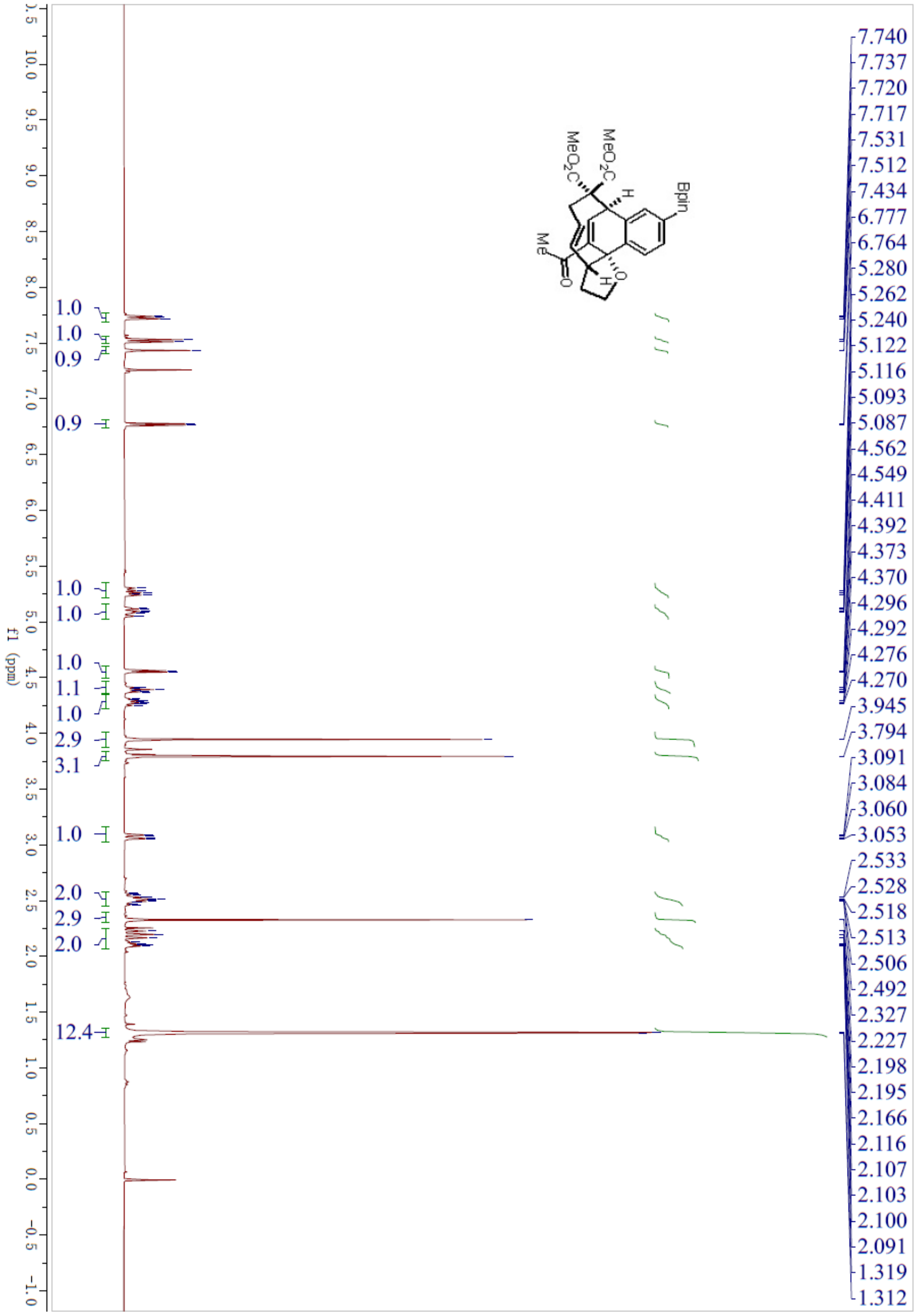

^{13}C NMR Spectrum (100 MHz, $CDCl_3$) of *E*-**2t**

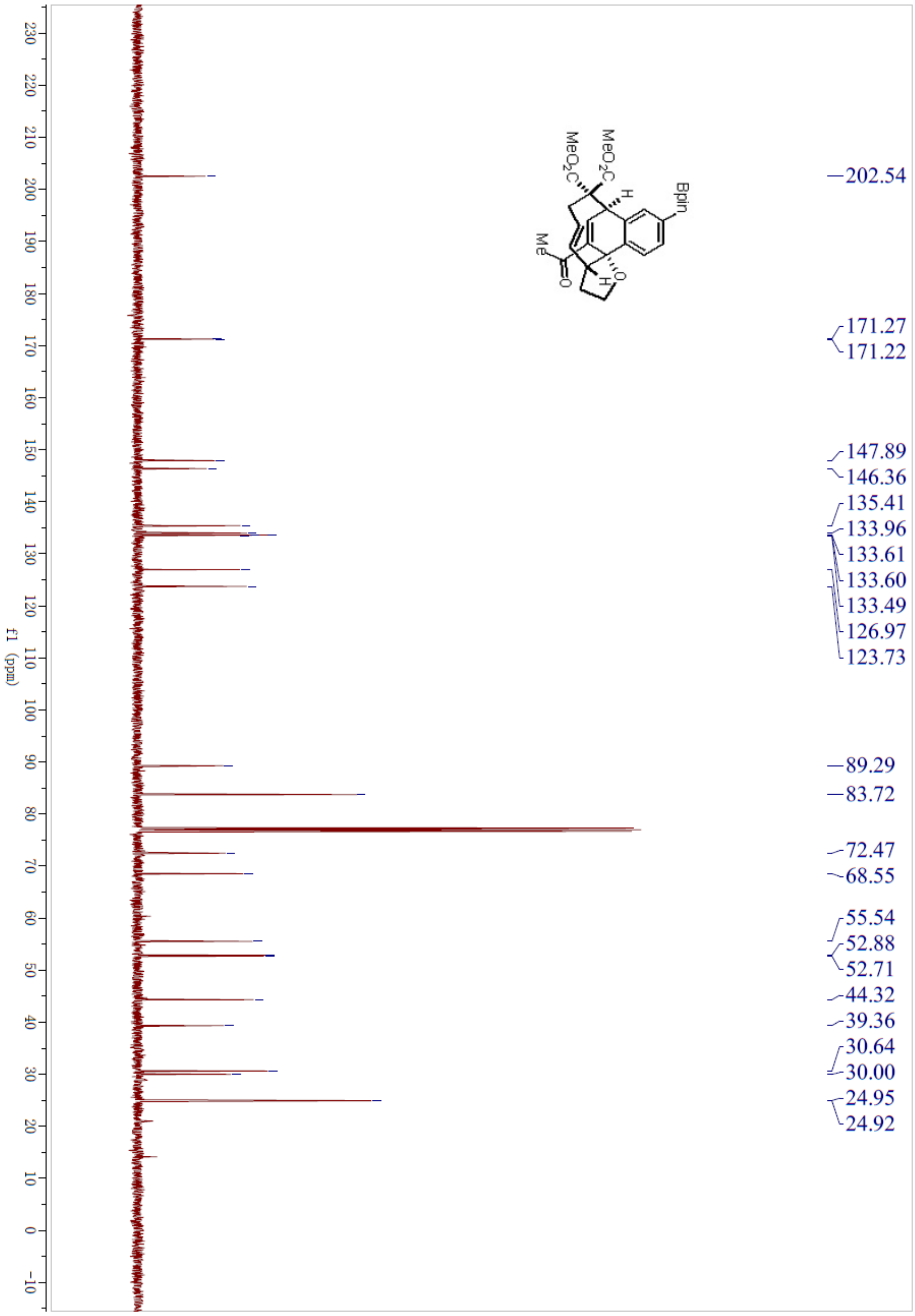

^{1}H NMR Spectrum (400 MHz, $CDCl_3$) of **10a**

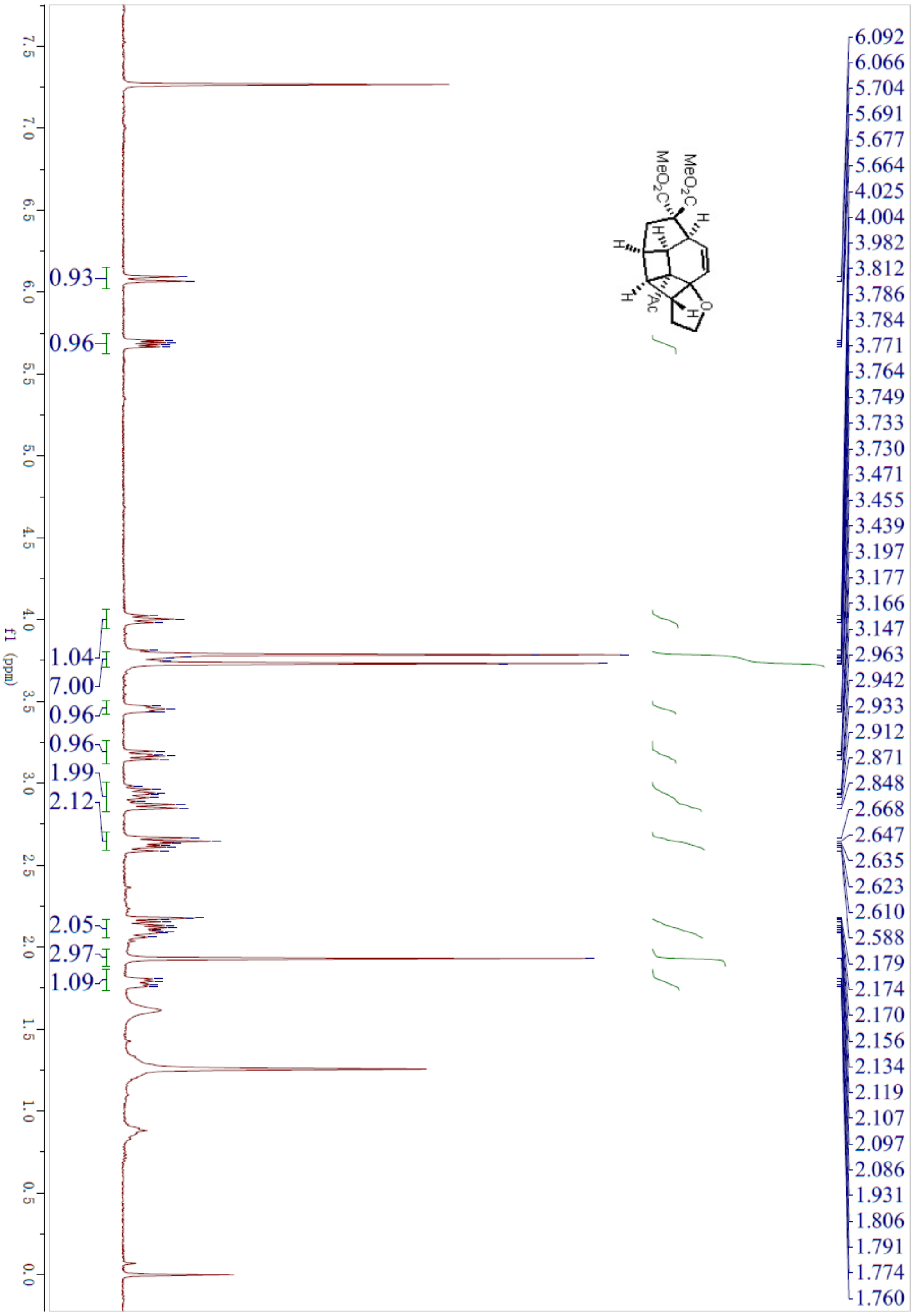

^{13}C NMR Spectrum (100 MHz, CDCl3) of **10a**

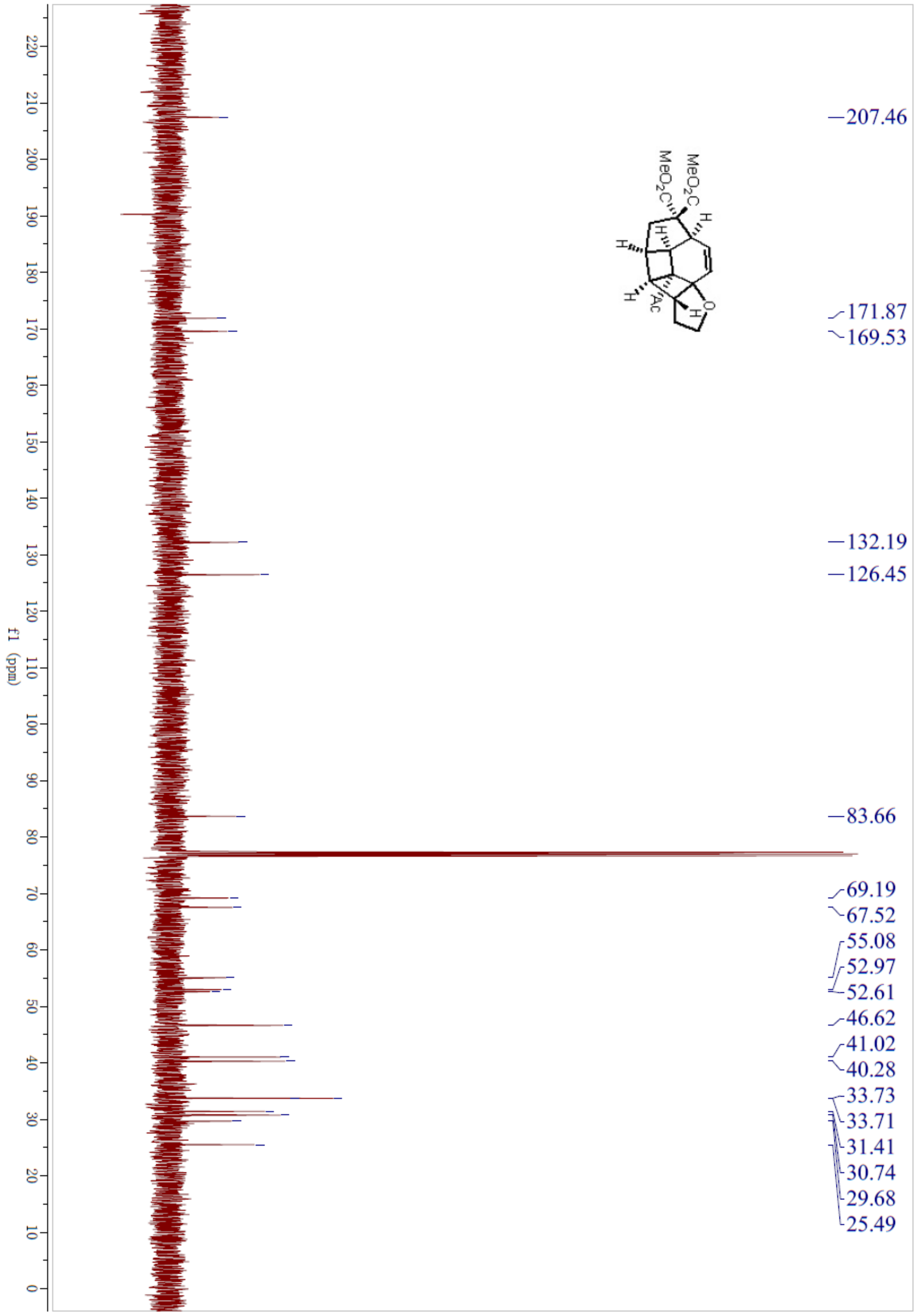

^{1}H NMR Spectrum (400 MHz, $CDCl_3$) of **10b**

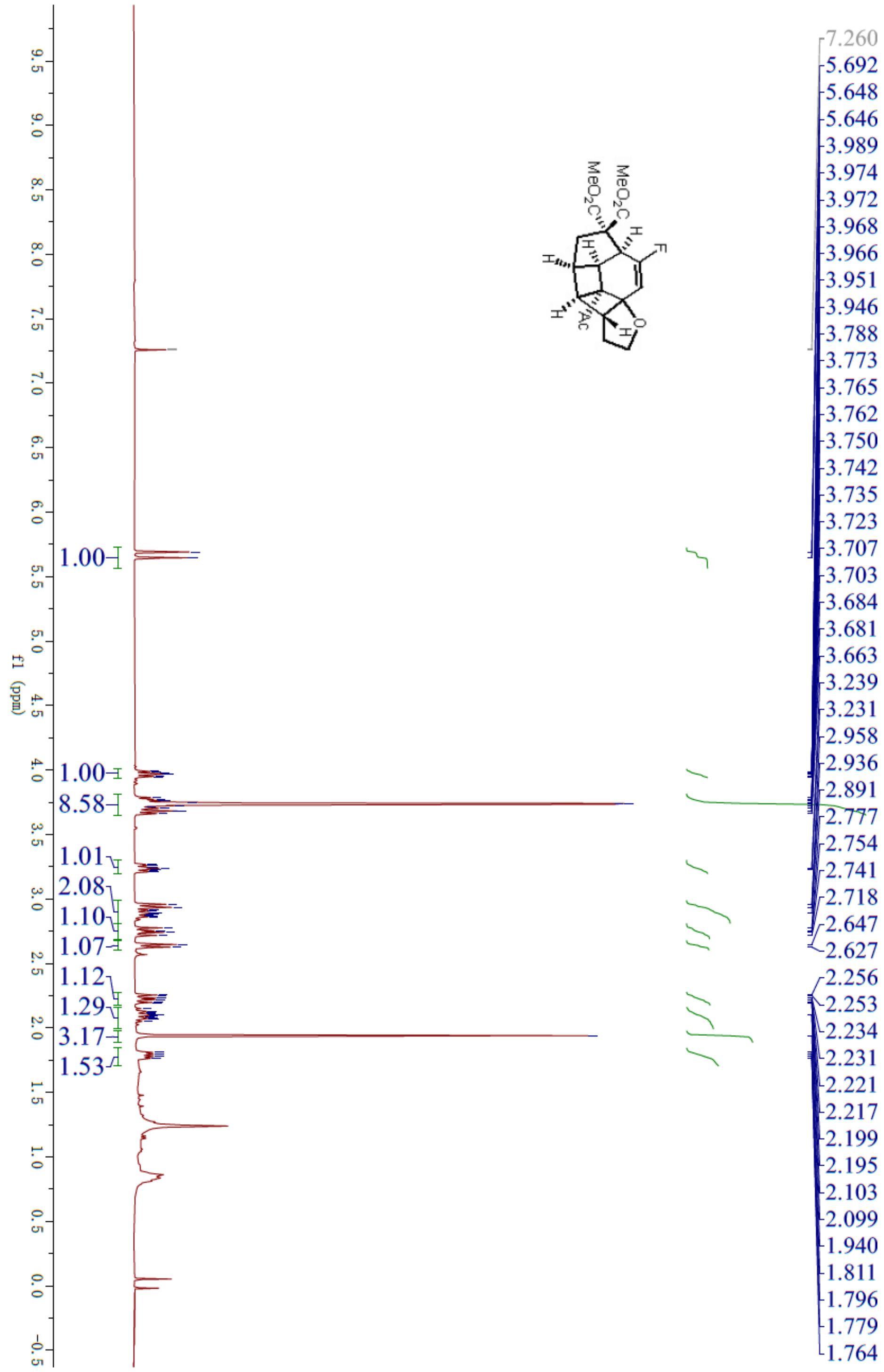

^{13}C NMR Spectrum (100 MHz, $CDCl_3$) of **10b**

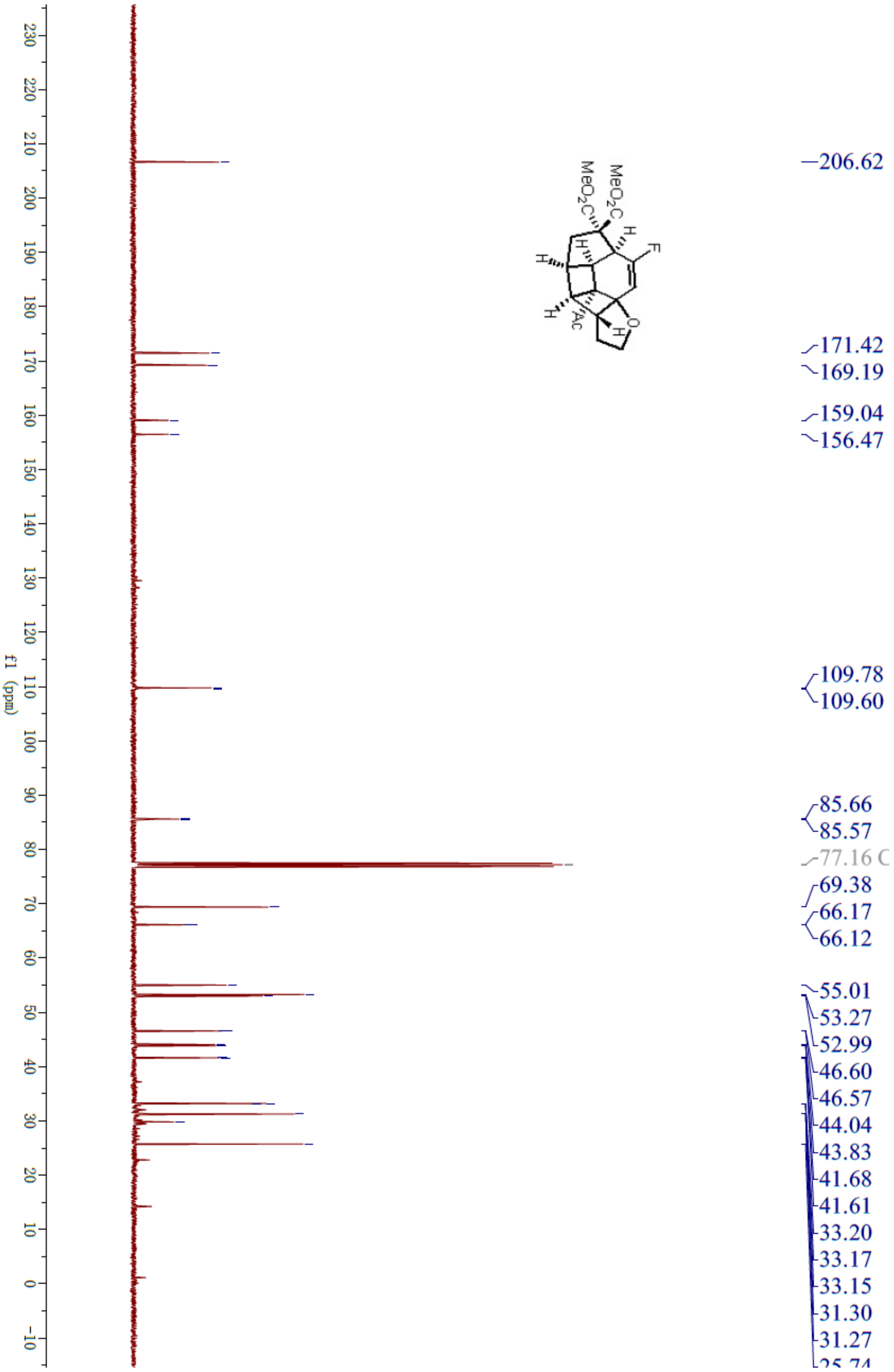

^{19}F NMR Spectrum (396 MHz, CDCl$_3$) of **10b**

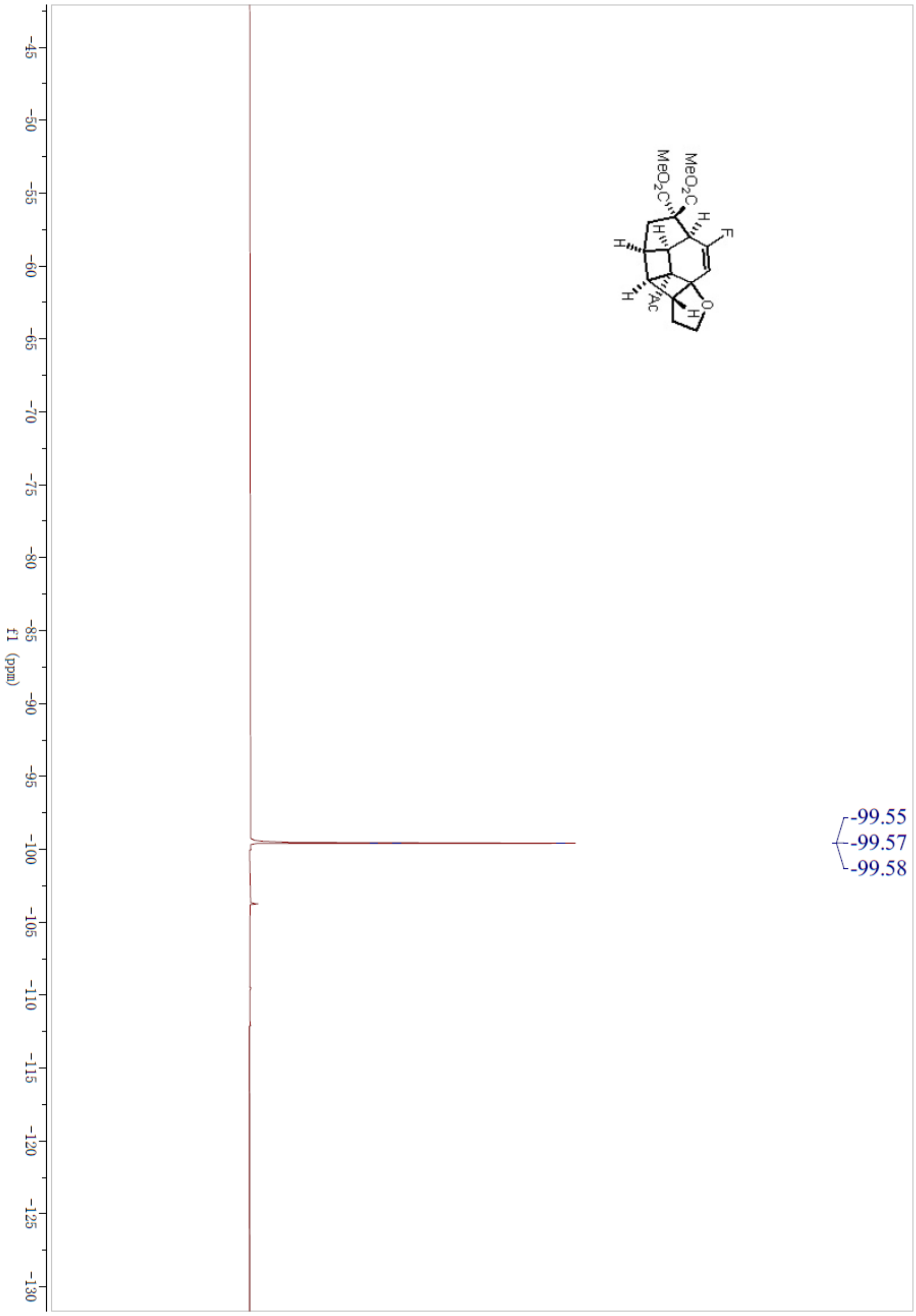

^{1}H NMR Spectrum (400 MHz, $CDCl_3$) of **3**

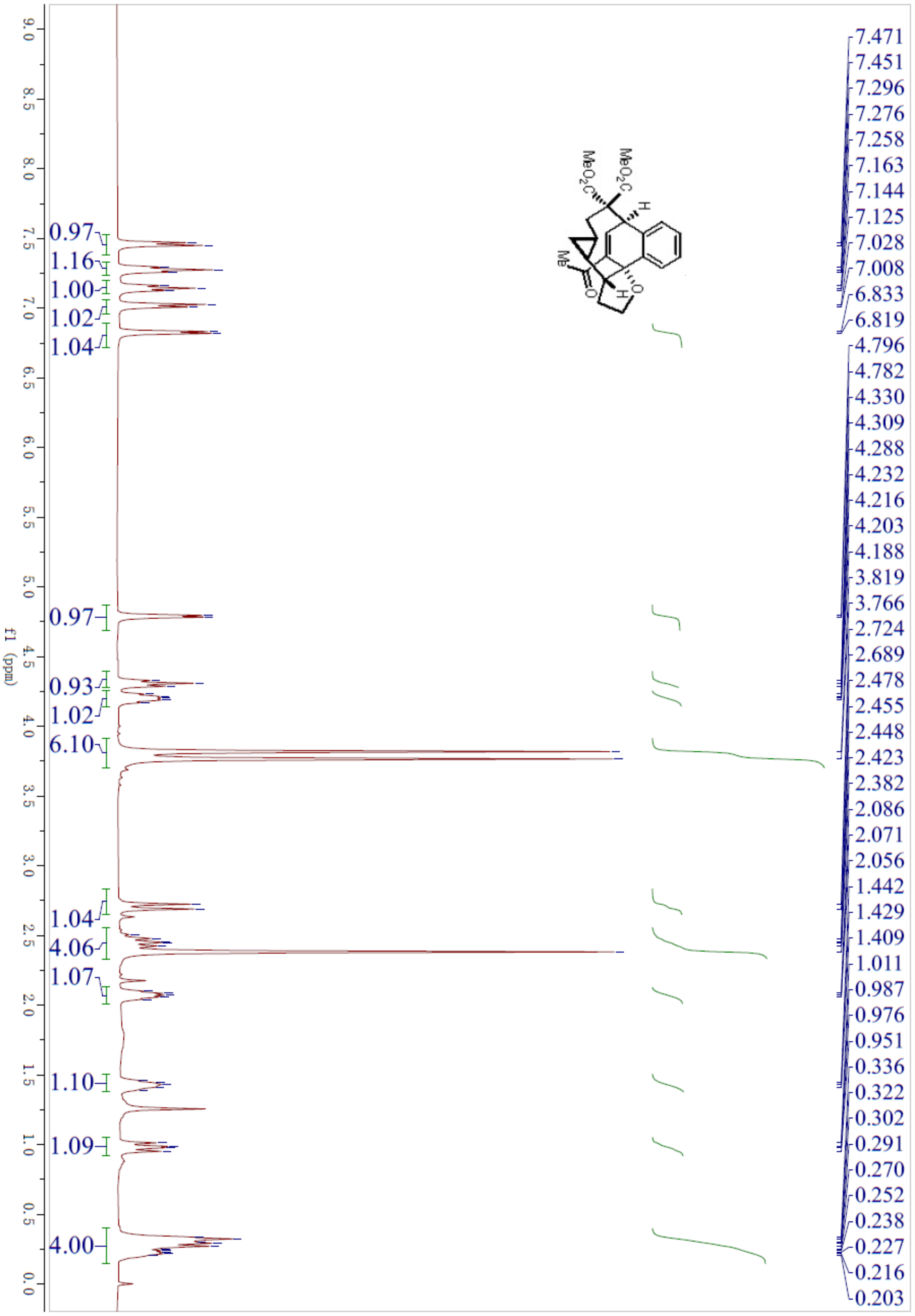

^{13}C NMR Spectrum (100 MHz, CDCl3) of **3**

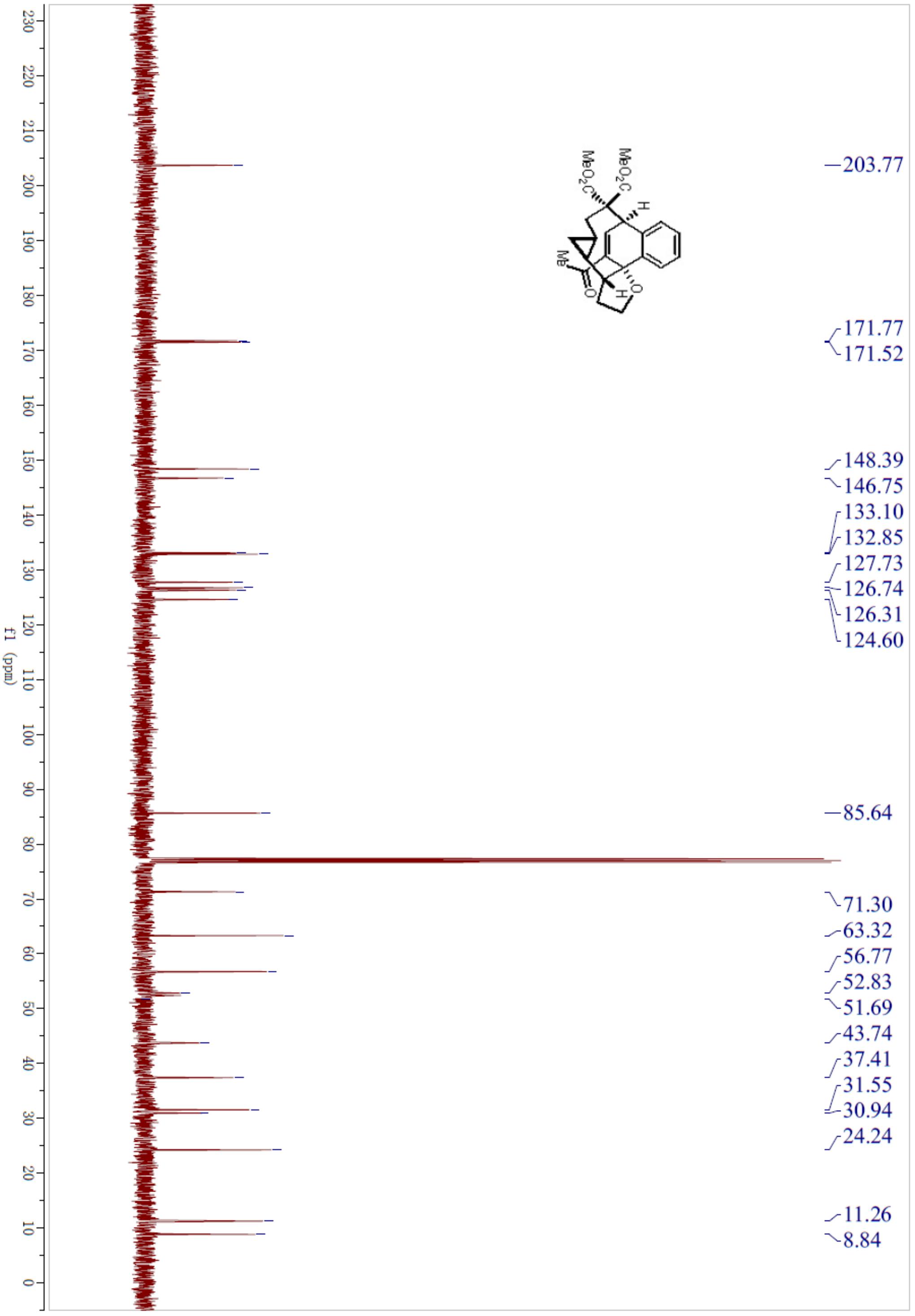

^{1}H NMR Spectrum (400 MHz, $CDCl_3$) of **4**

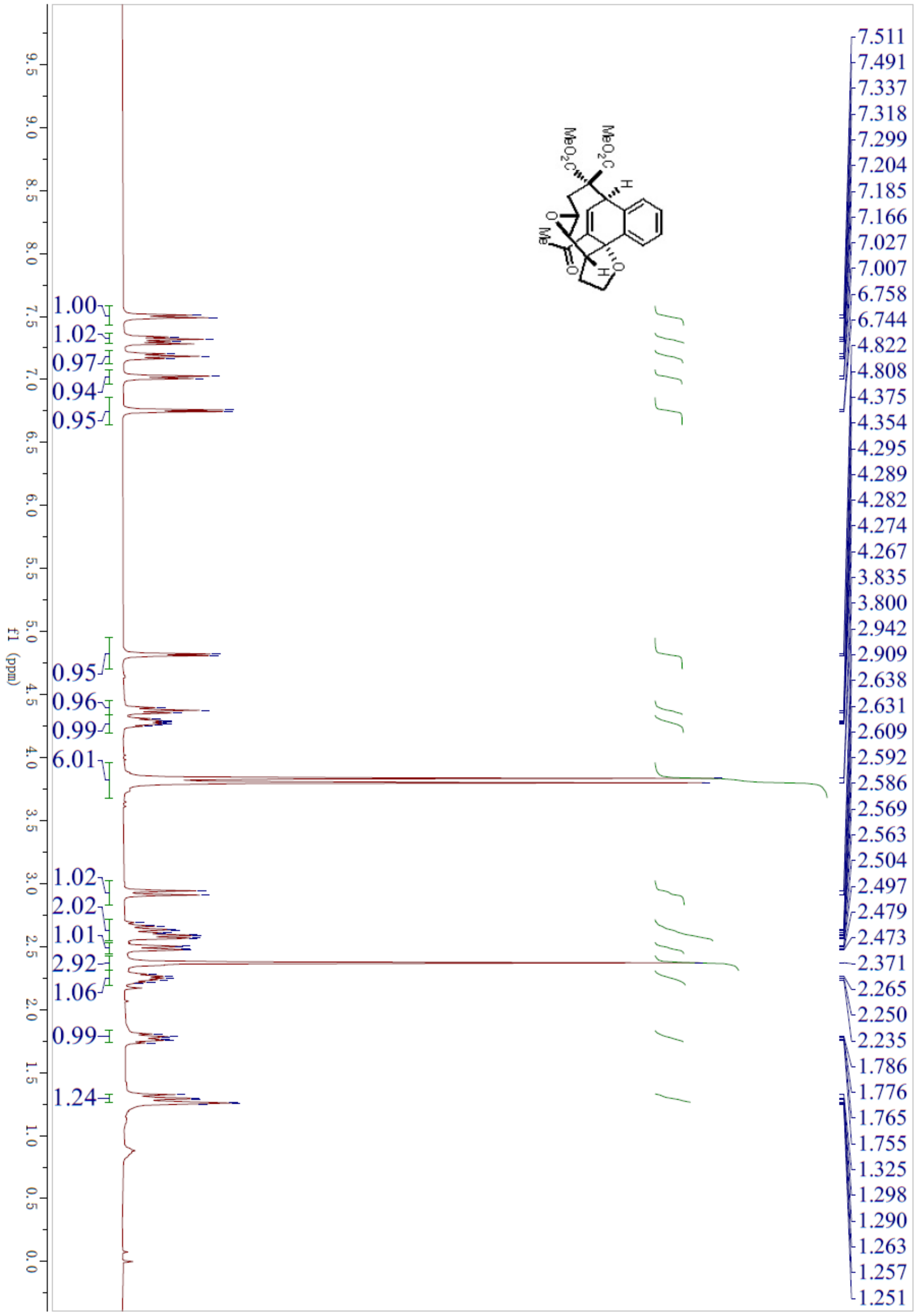

^{13}C NMR Spectrum (100 MHz, $CDCl_3$) of **4**

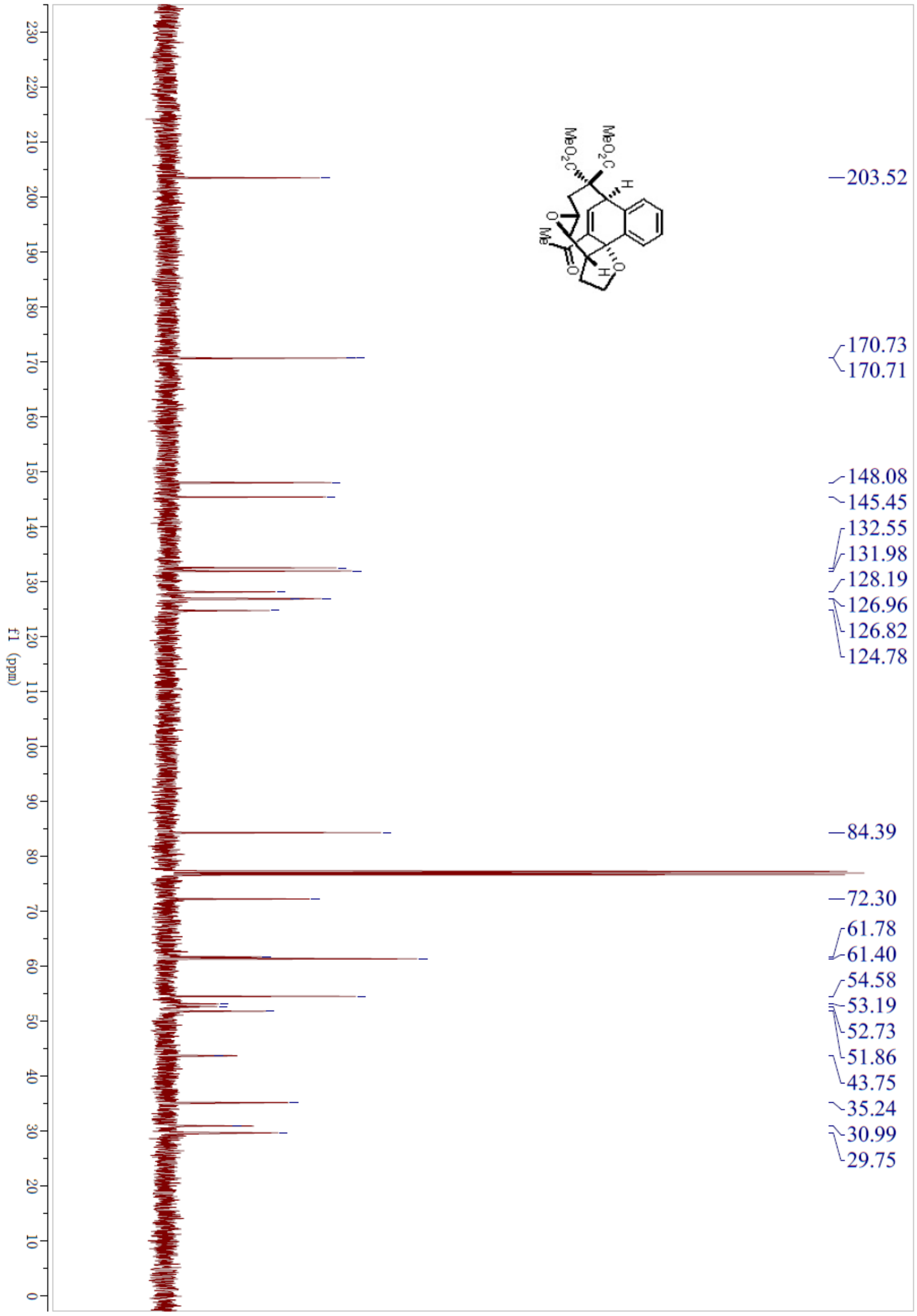

^{1}H NMR Spectrum (400 MHz, $CDCl_3$) of **5**

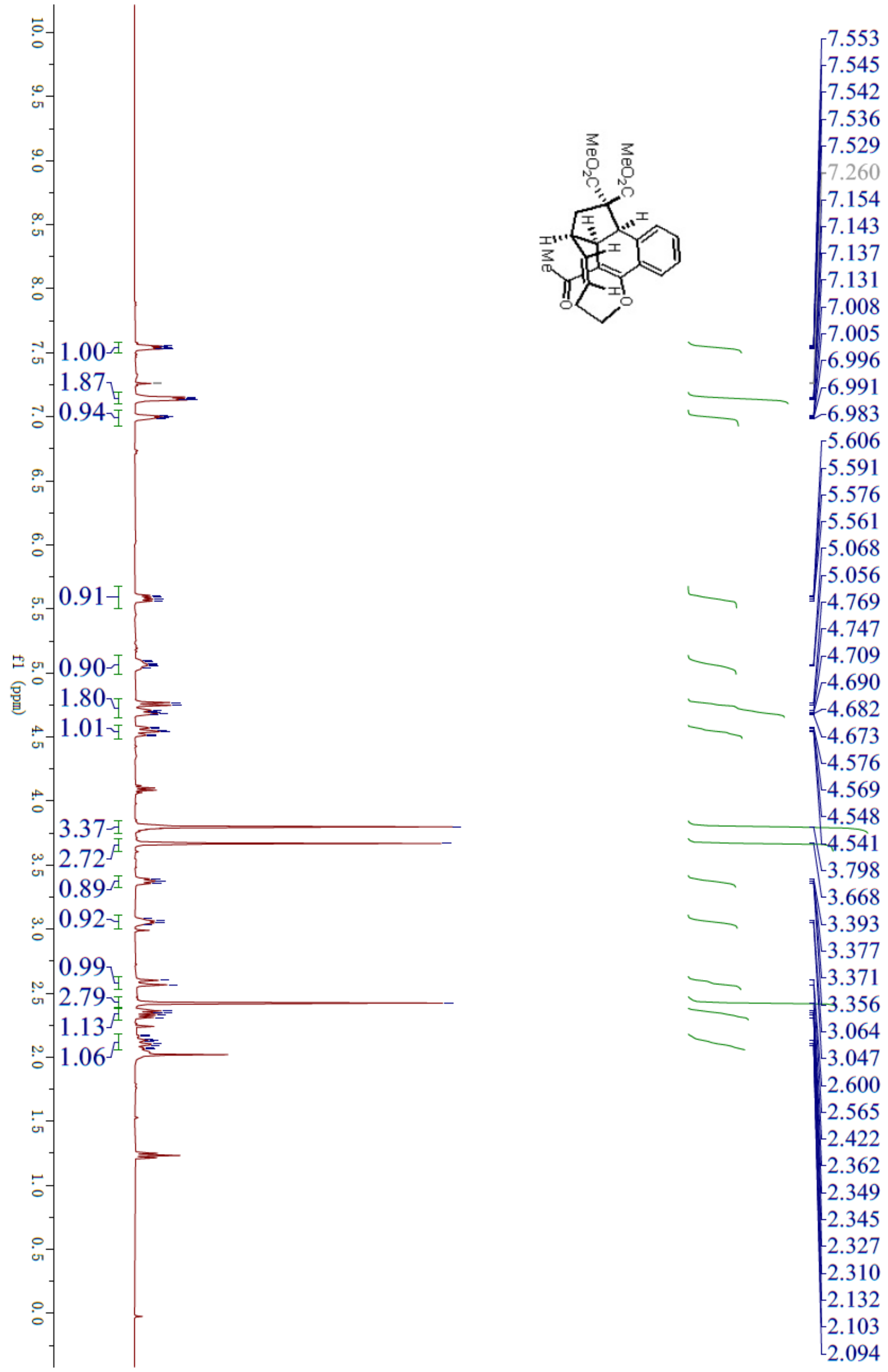

^{13}C NMR Spectrum (100 MHz, $CDCl_3$) of **5**

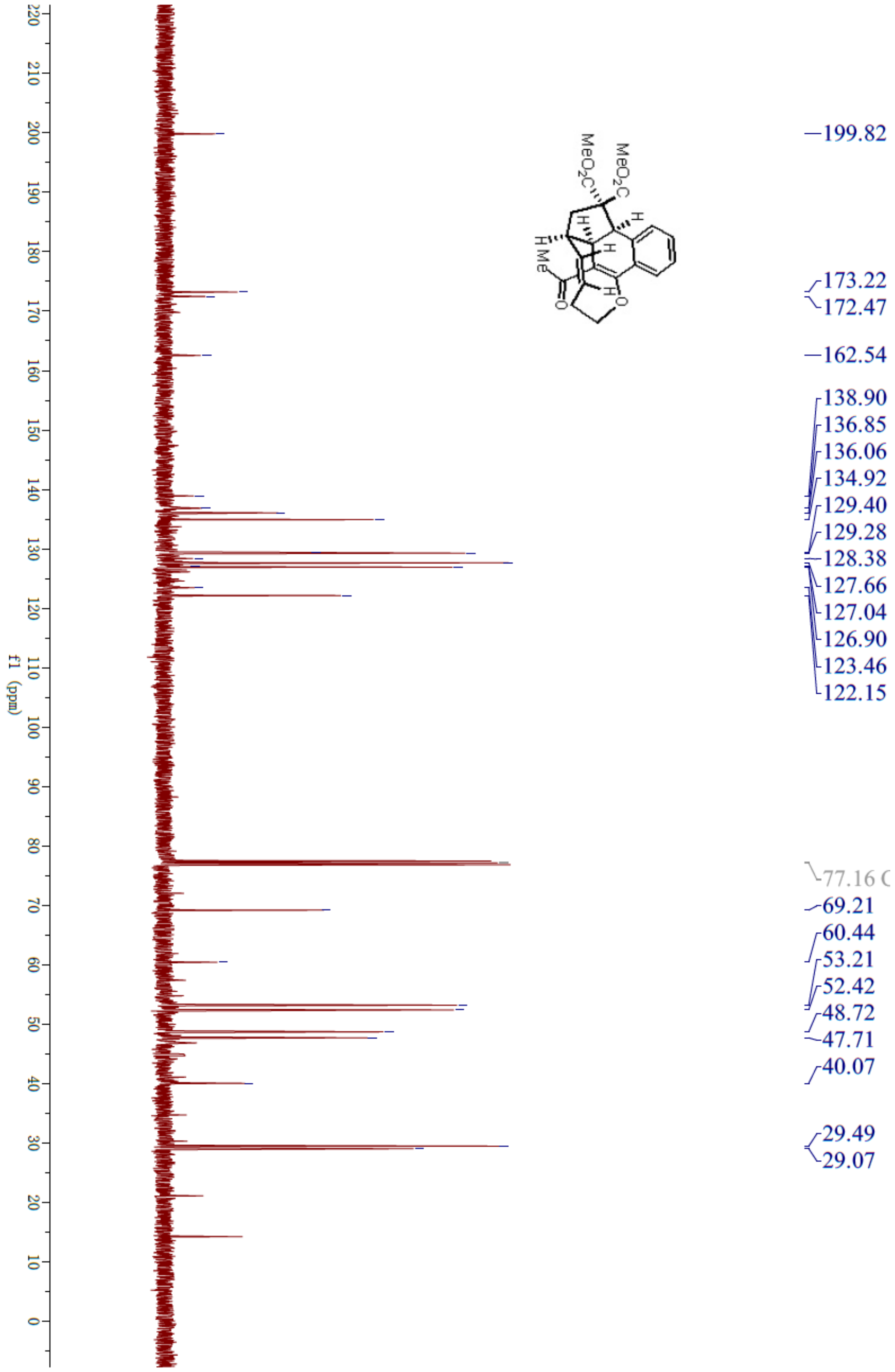

^{1}H NMR Spectrum (400 MHz, $CDCl_3$) of **6**

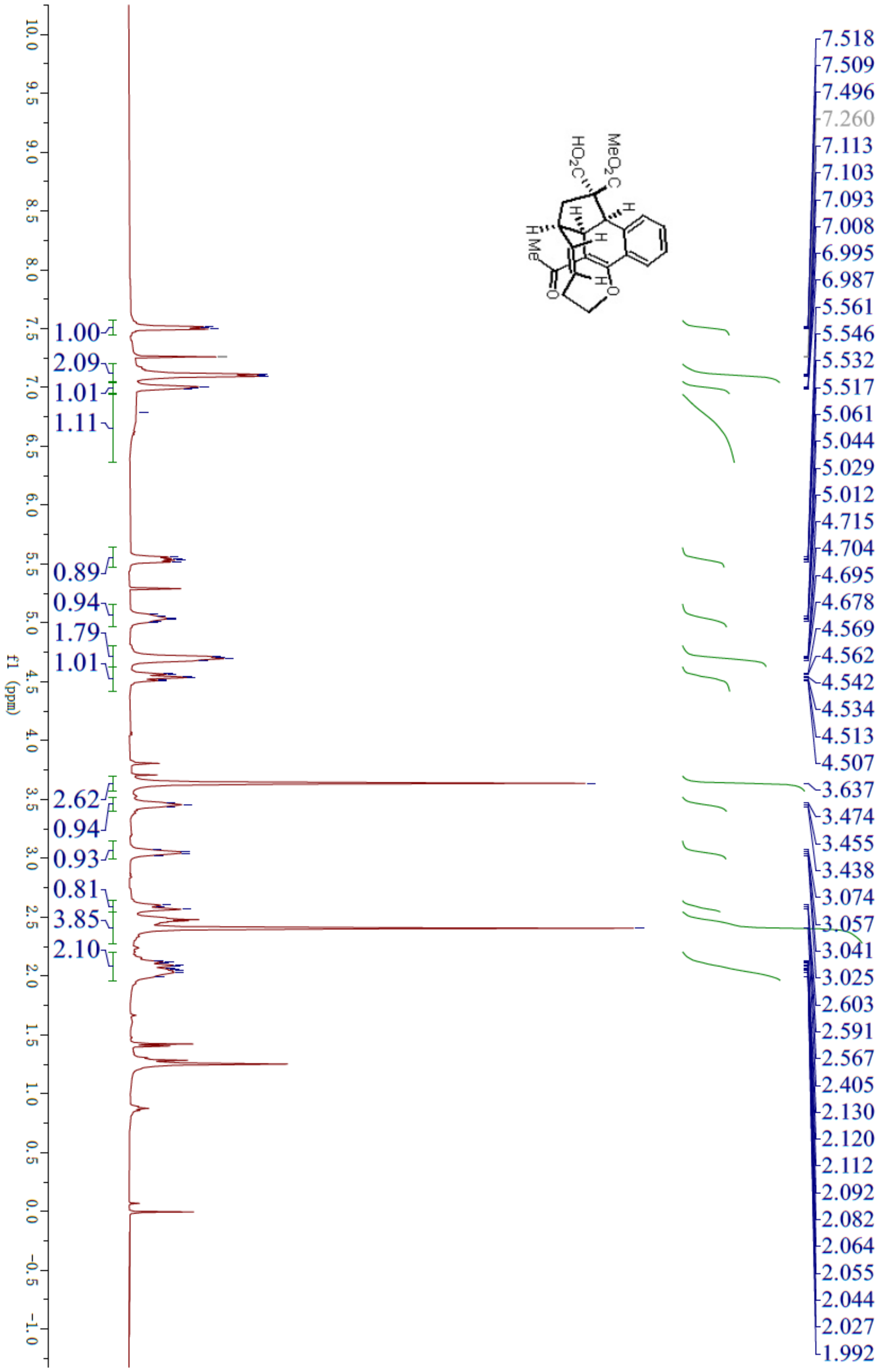

^{13}C NMR Spectrum (100 MHz, $CDCl_3$) of **6**

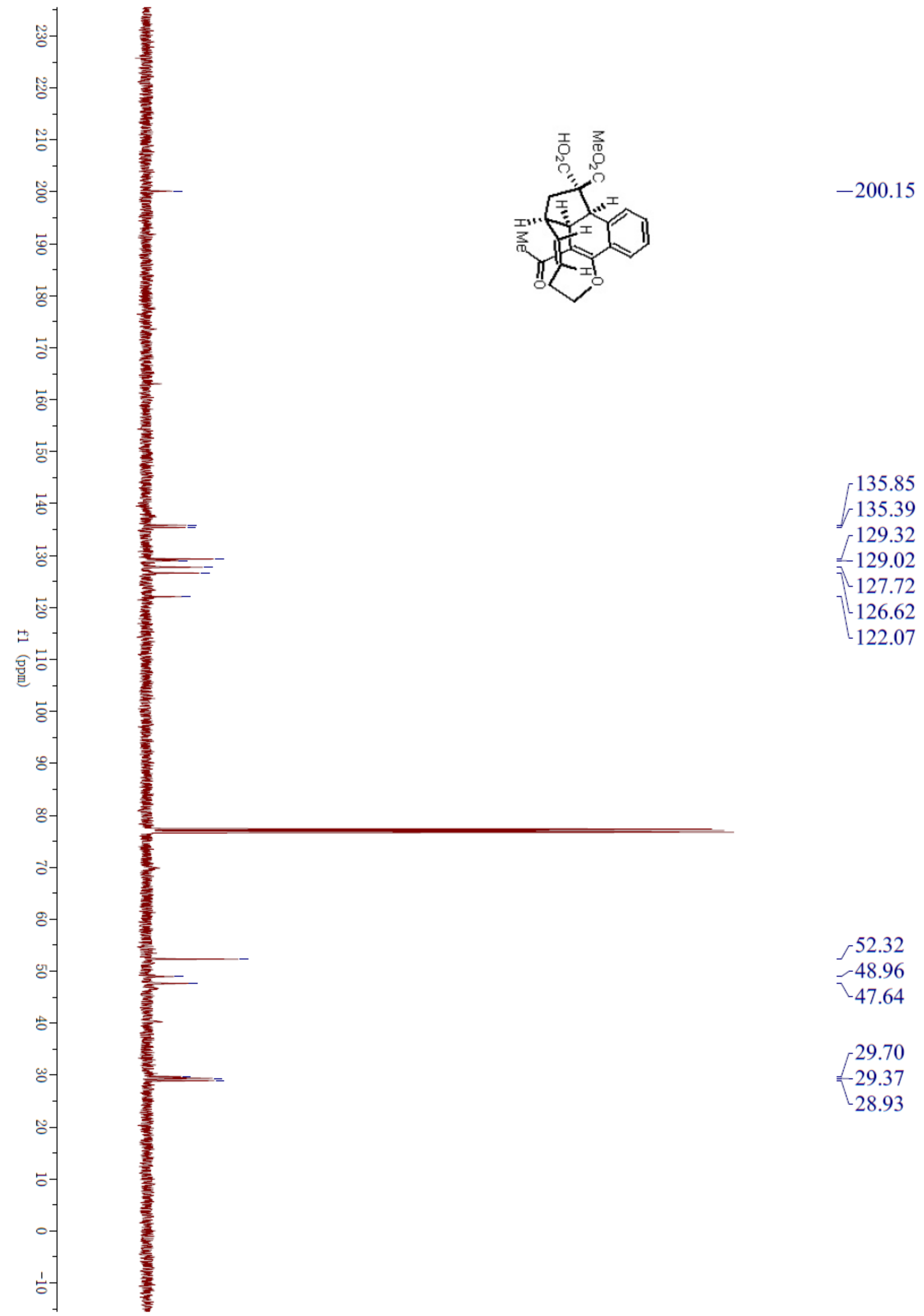

^{1}H NMR Spectrum (400 MHz, $CDCl_3$) of **7**

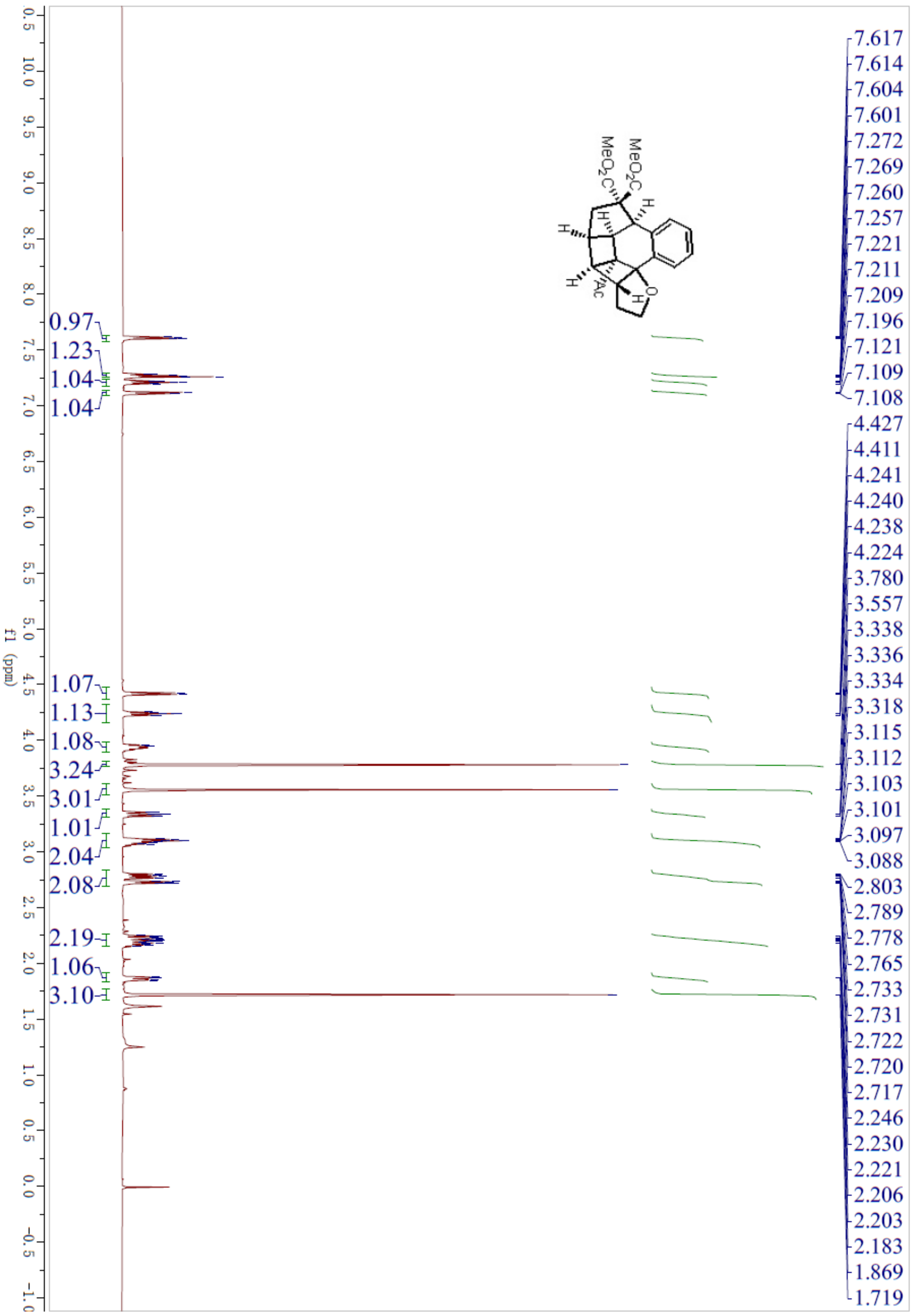

^{13}C NMR Spectrum (100 MHz, $CDCl_3$) of **7**

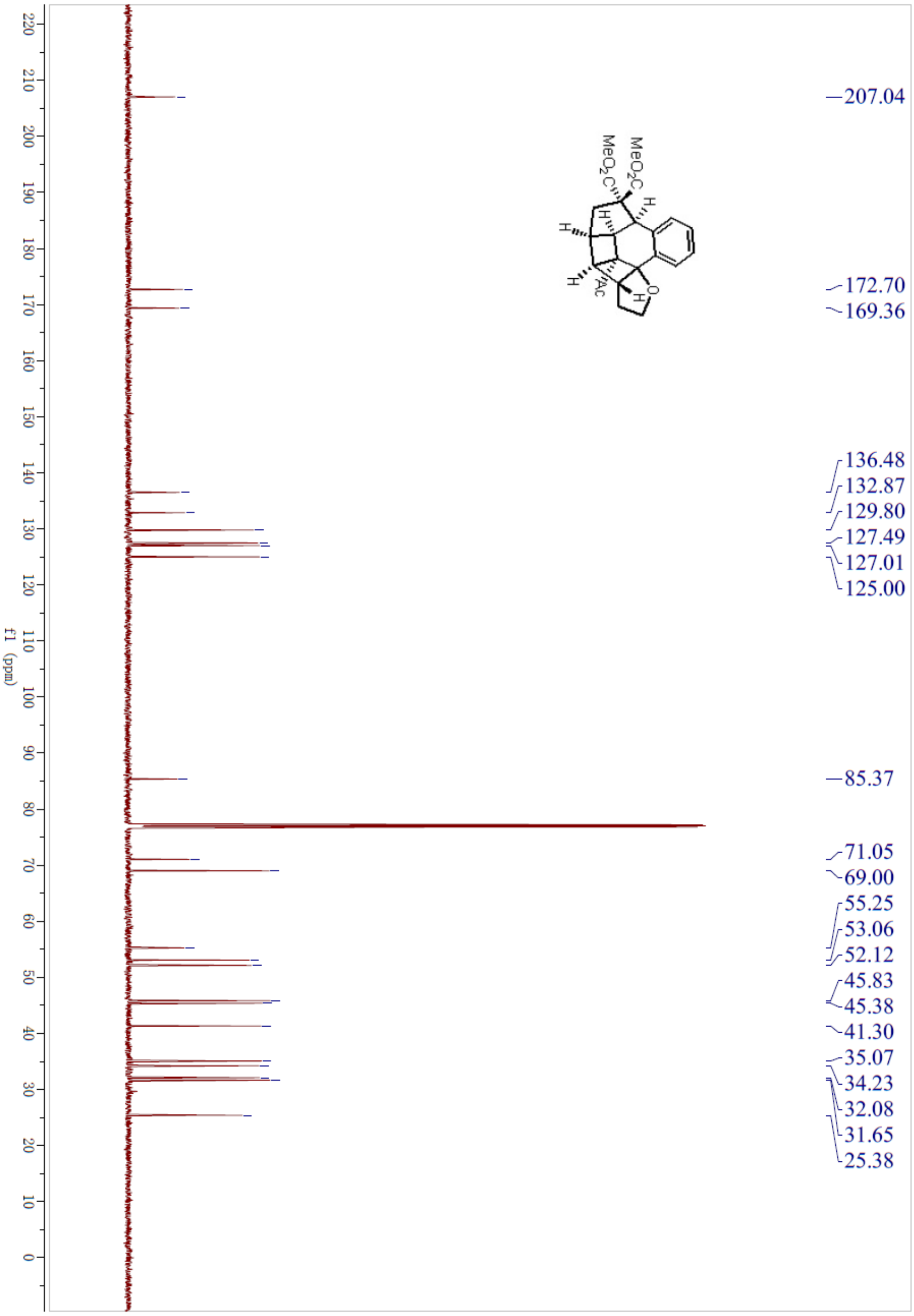

^{1}H NMR Spectrum (400 MHz, $CDCl_3$) of **8**

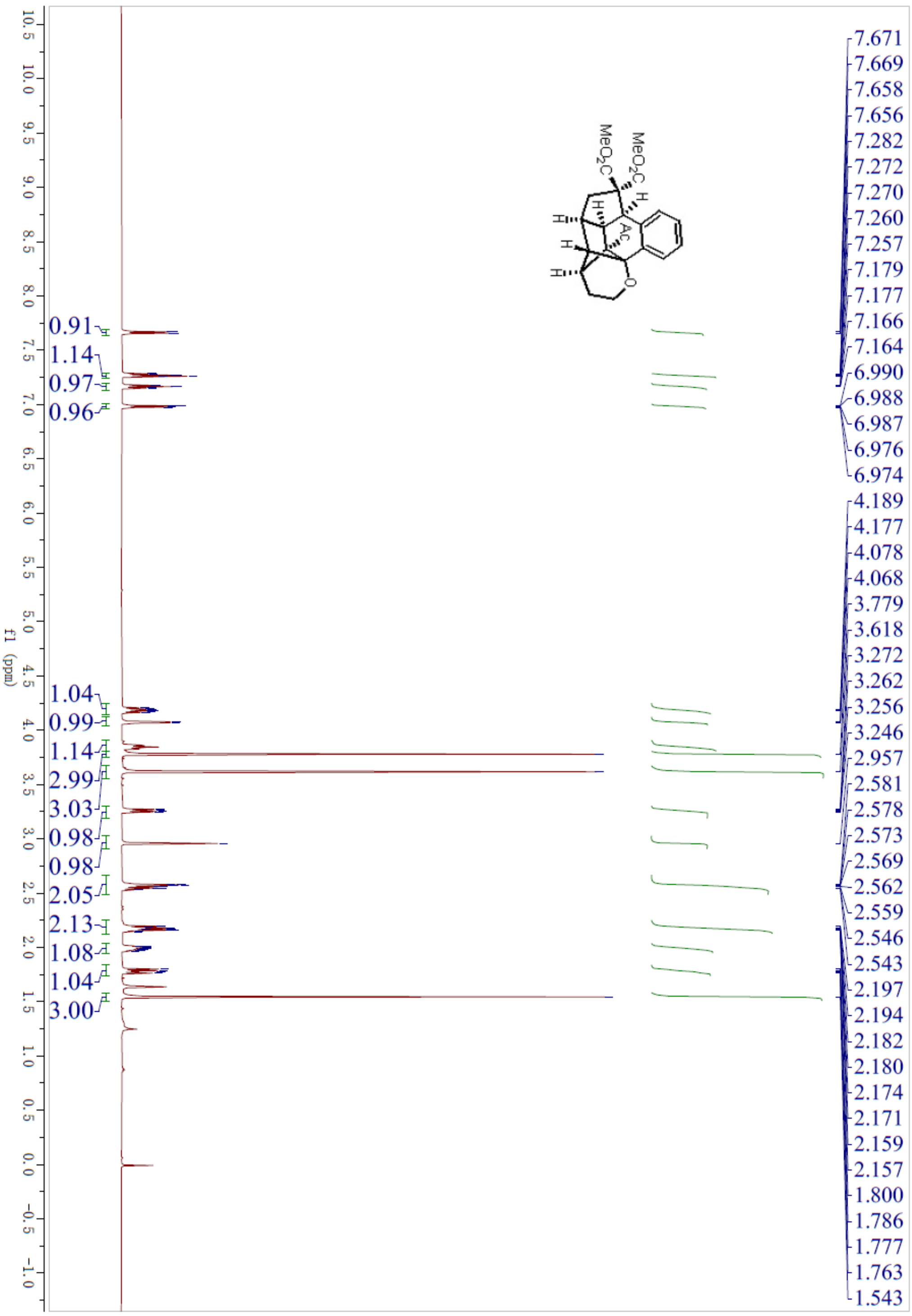

^{13}C NMR Spectrum (100 MHz, $CDCl_3$) of **8**

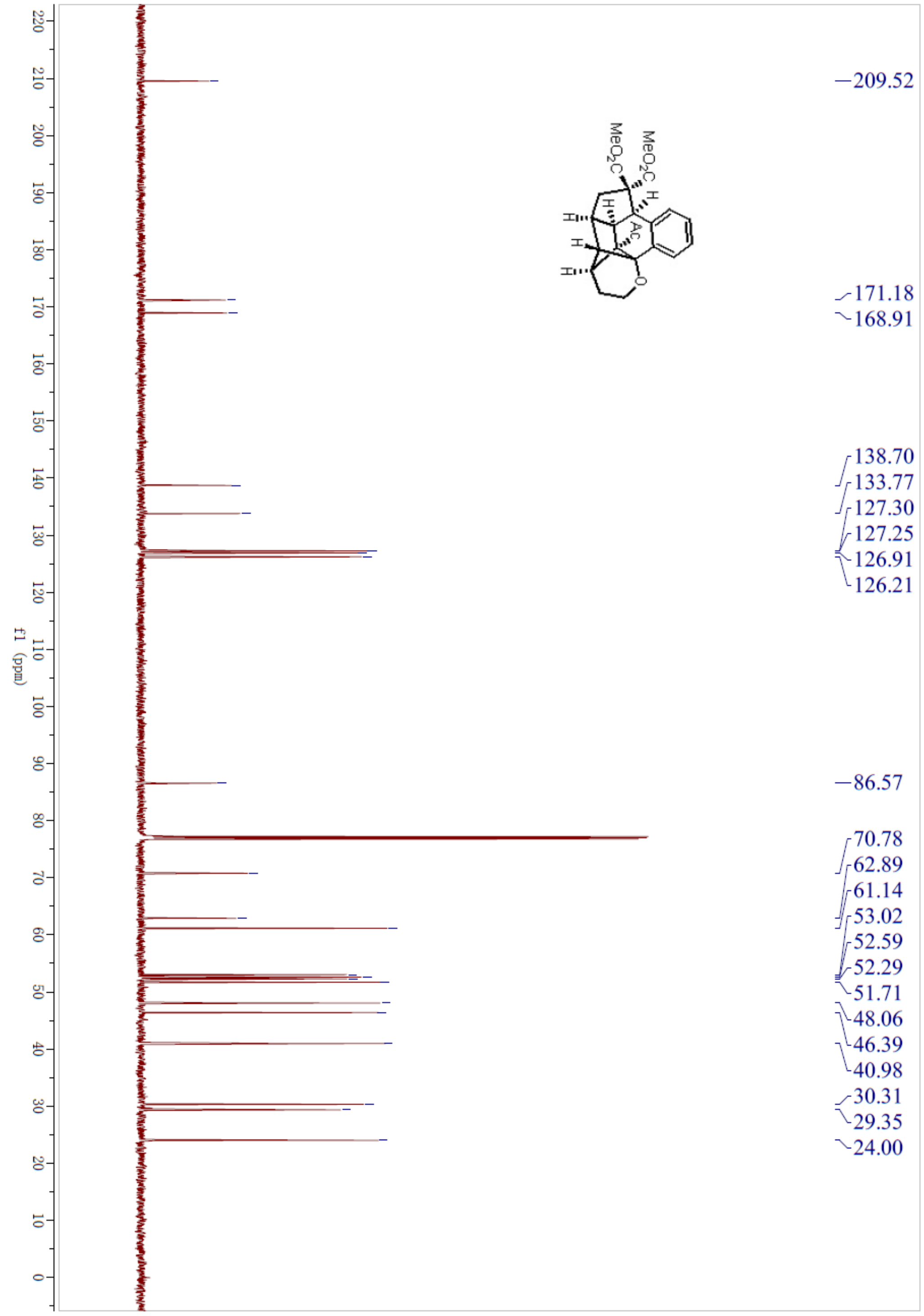

7. Unsuccessful Substrates

The unsuccessful substrates for the desired intramolecular dearomatization reactions are listed in Supplementary Figure 9.

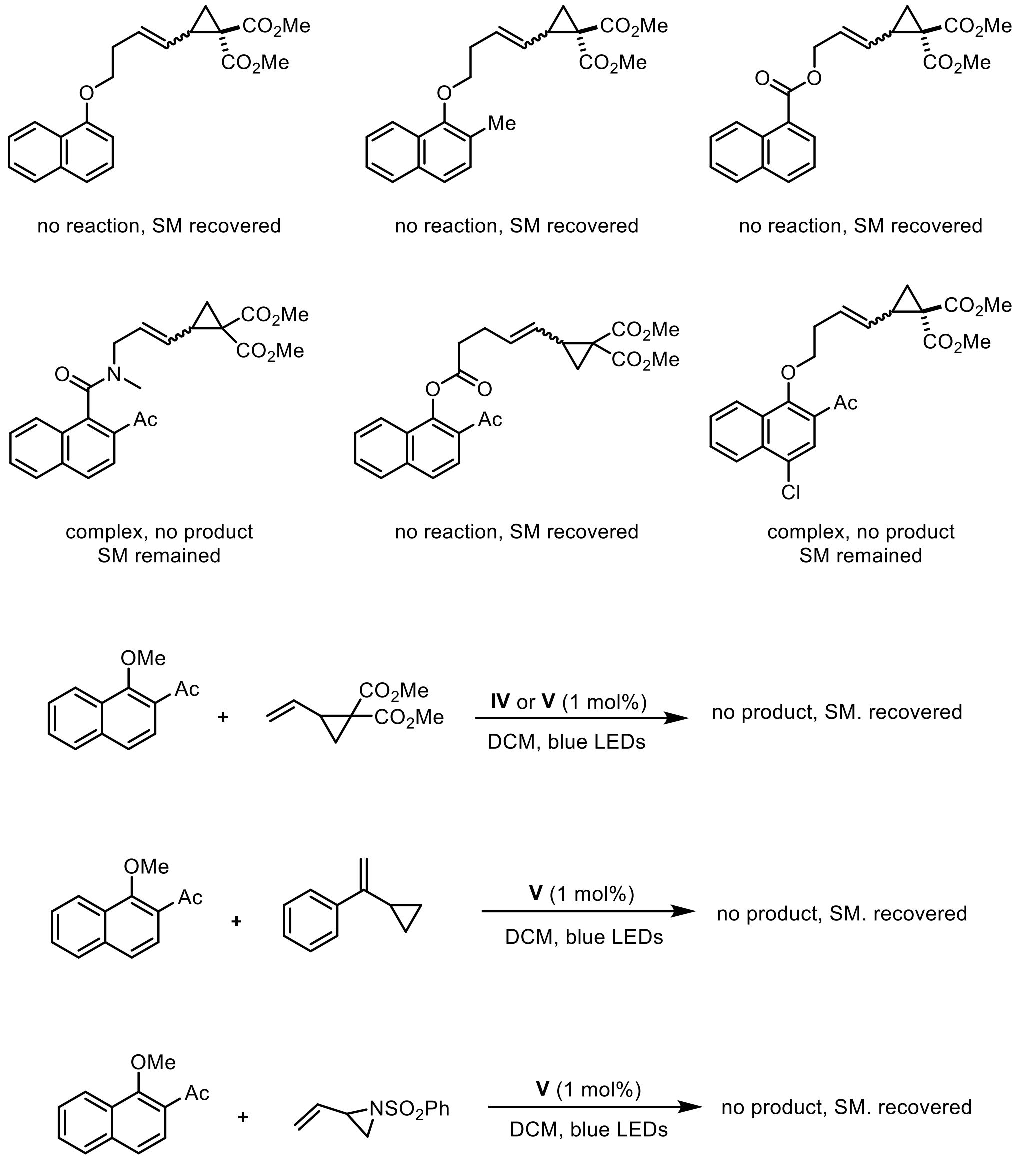

Supplementary Figure 9. Unsuccessful substrates for the desired intramolecular dearomatization reactions.

8. References

[1] Feng, Y. *et al.* Synthesis of antitricyclic morpholine derivatives through iodine(III)-mediated intramolecular umpolung cycloaddition of olefins. *J. Org. Chem.* **85**, 4500–4506 (2020).

[2] James, M. J., Schwarz, J. L., Strieth-Kalthoff, F., Wibbeling, B. & Glorius, F. Dearomative cascade photocatalysis: divergent synthesis through catalyst selective energy transfer. *J. Am. Chem. Soc.* **140**, 8624–8628 (2018).

[3] Montalti, M., Credi, A., Prodi, L. & Gandolfi, M. T. *Handbook of Photochemistry*, 3rd ed, Ed.; CRC, Taylor & Francis Group, Boca Raton, FL, (2006).

[4] Frisch, M. J. *et al.* Gaussian 16, Revision A.03, Gaussian, Inc., Wallingford CT, 2016.

[5] Becke, A. D. Density–functional thermochemistry. III. The role of exact exchange. *J. Chem. Phys.* **98**, 5648–5652 (1993).

[6] Lee, C., Yang, W. & Parr, R. G. Development of the Colle–Salvetti correlation-energy formula into a functional of the electron density. *Phys. Rev. B* **37**, 785–789 (1988).

[7] Stephens, P. J., Devlin, F. J., Chabalowski, C. F. & Frisch, M. J. *Ab Initio* calculation of vibrational absorption and circular dichroism spectra using density functional force fields. *J. Phys. Chem.* **98**, 11623–11627 (1994).

[8] Grimme, S., Ehrlich, S. & Goerigk, L. Effect of the damping function in dispersion corrected density functional theory. *J. Comp. Chem.* **32**, 1456–1465 (2011).

[9] Grimme, S., Antony, J., Ehrlich, S. & Krieg, H. A consistent and accurate *ab initio* parametrization of density functional dispersion correction (DFT-D) for the 94 elements H–Pu. *J. Chem. Phys.* **132**, 154104 (2010).

[10] Weigend, F. & Ahlrichs, R. Balanced basis sets of split valence, triple zeta valence and quadruple zeta valence quality for H to Rn: Design and assessment of accuracy. *Phys. Chem. Chem. Phys.* **7**, 3297–3305 (2005).

[11] Weigend, F. Accurate Coulomb-fitting basis sets for H to Rn. *Phys. Chem. Chem. Phys.* **8**, 1057–1065 (2006).

[12] Marenich, A. V., Cramer, C. J. & Truhlar, D. G. Universal solvation model based on solute electron density and on a continuum model of the solvent defined by the

bulk dielectric constant and atomic surface tensions. *J. Phys. Chem. B* **113**, 6378–6396 (2009).

[13] Harvey, J. N., Aschi, M., Schwarz, H. & Koch, W. The singlet and triplet states of phenyl cation. A hybrid approach for locating minimum energy crossing points between non-interacting potential energy surfaces. *Theor. Chem. Acc.* **99**, 95–99 (1998).

[14] Lu, T. sobMECP program; http://sobereva.com/286.
